# Stable Monoareno-pentalenes with Two Olefinic Protons

**DOI:** 10.1021/acs.orglett.2c03752

**Published:** 2022-12-28

**Authors:** Péter J. Mayer, Gábor London

**Affiliations:** † MTA TTK Lendület Functional Organic Materials Research Group, Institute of Organic Chemistry, Research Centre for Natural Sciences, Magyar tudósok krt 2., Budapest, 1117, Hungary; ‡ Institute of Chemistry, University of Szeged, Rerrich tér 1., Szeged, 6720, Hungary

## Abstract

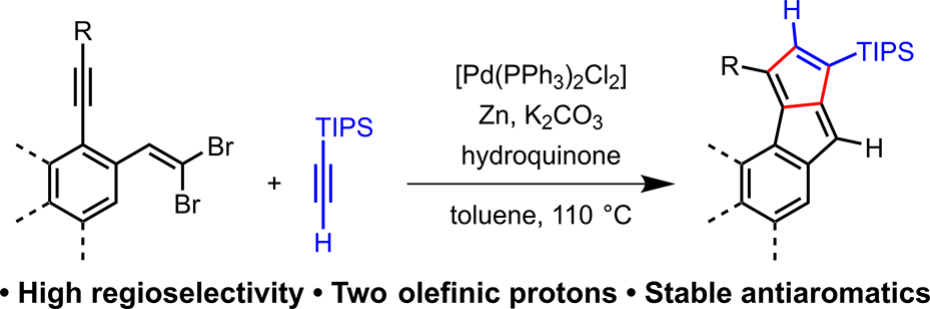

A novel class of stable monoareno-pentalenes is introduced that have an olefinic proton on each five-membered ring of the pentalene subunit. Their synthesis was accomplished via a regioselective carbopalladation cascade reaction between *ortho*-arylacetyleno *gem*-dibromoolefins and TIPS-acetylene. These molecules could be experimental probes of magnetic (anti)aromaticity effects.

Pentalene, an 8π antiaromatic hydrocarbon, holds interest in terms of both its electronic structure and as a functional component of organic semiconducting materials.^1^ Since pentalene itself is only stable below −196 °C,^2^ its properties are most conveniently studied experimentally through its stabilized derivatives.^3^ A general approach to prepare stable derivatives of pentalene is its π-extension. However, this modification can considerably alter the properties of pentalene depending on the number and the nature of the fused rings or ring systems.^4,5^ Diareno[*a*,*e*]pentalenes have been prepared in great diversity^3a,6^ and proved to be stable compounds, along with generally alleviated antiaromaticity of the pentalene core.^7^ On the other hand, diareno[*a*,*f*]pentalenes exhibit decreased stability due to their strong antiaromaticity combined with an open-shell character.^8^ Stable monoareno-pentalenes have been reported^5,9^ and showed more preserved antiaromaticity than diareno[*a*,*e*]pentalenes.^5,9c−9f^ Furthermore, the antiaromaticity of pentalene in its arene-fused derivatives is influenced by the bond order of the fused bond, as increased double-bond character leads to increased antiaromaticity.^10^ Finding a balance between stability and well-preserved antiaromaticity, which is interesting for both fundamental understanding and applications, is a goal of most structure–property relationship studies.

It is clear that benzannulation and ring substitution are vital to obtain stable pentalene derivatives. This, however, leads to molecules that lack free olefinic (pentalenic) protons. In the characterization of pentalenes, protons attached to the pentalene core could be informative through their ^1^H NMR shifts. These olefinic proton shifts reflect paratropic ring current effects associated with antiaromaticity.^11^ Most of the stable pentalene derivatives have no such protons, and those that have a single are usually diarenopentalenes with already low levels of antiaromaticity.

Regarding experimental and theoretical aspects of magnetic antiaromaticity, monoareno-pentalenes are an interesting class of compounds.^9^ When having at least three pendant aryl substituents on the pentalene core (Figure 1, **TPBP**), these molecules are stable and exhibit strongly preserved antiaromaticity.^5,9^ Furthermore, the introduction of an unsubstituted olefinic proton is possible^5,9c−9e^ and, importantly, their structure is asymmetric, which provides the possibility to study the spatial distribution of ring-currents in the molecules.^12^

Differences in the strength of paratropicity within the different five-membered rings in monobenzopentalenes have been shown via the calculation of current density maps and NICS values.^11b,13^ However, theoretical treatments of such systems often disregard substituents for convenience, which is usually not an option for organic synthesis. Hence, many of the subjects of computations are experimentally inaccessible. Nevertheless, unsubstituted monobenzopenalene (Figure 1, **BP**) has been reported to form upon flash-vacuum pyrolysis of 3-phenylphthalic anhydride but found stable only below −70 °C, otherwise dimerized.^14^ Stable monoareno-pentalenes having olefinic Hs on each 5-membered ring are unknown, which prompted us to explore their synthesis and stability. Such molecules would not only provide new information regarding structure–stability relationships among pentalenes but also allow for gaining experimental insight into antiaromaticity effects.

**Figure 1:**
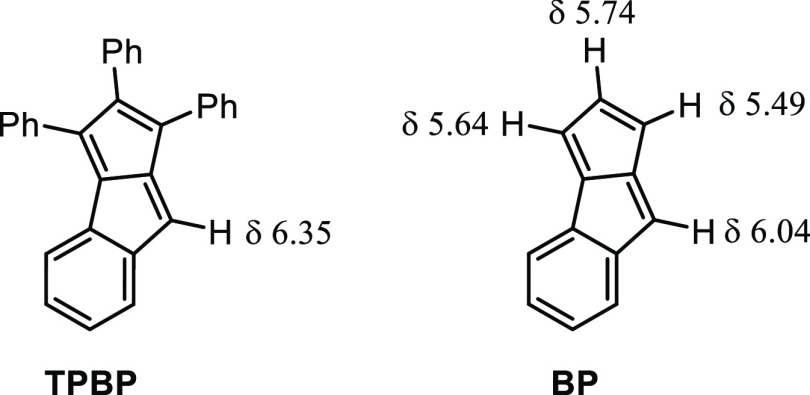
^1^H NMR chemical shifts of the olefinic protons in triphenylbenzopentalene (**TPBP**) (500 MHz, CD_2_Cl_2_) and benzopentalene (**BP**) (300 MHz, CD_2_Cl_2_).

The most straightforward way to access stable monoareno-pentalenes is the carbopalladation cascade between acetylenes and *gem*-dibromoolefins.^9c^ In this transformation, a *gem*-dibromoolefin reacts with an internal alkyne, which so far has been exclusively diphenylacetylene derivatives.^5,9c−9e^ Likely, the application of an unsymmetric alkyne would lead to a nonselective reaction complicating purification. Yet, based on the proposed reaction mechanism^9b^ (Figure 2), the selectivity of the alkyne attachment could possibly be controlled by varying the size of the substituents (R^1^ and R^2^) of the reagent alkyne. Considering our goal to access monoareno-pentalenes that have an olefinic H on each 5-membered ring, terminal acetylenes were necessary for the reaction. Although possible side-reactions could be envisioned in the presence of terminal alkynes, the reaction between *gem*-dibromoolefins and TIPS-acetylene (R^1^ = H, R^2^ =TIPS) yielded the desired pentalene derivatives (Figure 3). Importantly, these compounds could be isolated by column chromatography and found stable under ambient conditions. In terms of the reaction mechanism, the results suggest that the first, intramolecular carbopalladation precedes all other steps. Furthermore, as no regioisomers were isolated or observed in the crude reaction mixture, the size of the alkyne substituents indeed controlled the selectivity. Apart from the parent benzopentalene derivative **1**, we synthesized both its π-extended (**2**–**4**) and substituted (**5**–**8**) derivatives to explore their properties (Figure 3). Molecules with phenyl (**1**–**8**) and 4-methoxyphenyl (**1′**–**7′**) substituents were prepared to check possible effects on ^1^H NMR shifts. Protons H_A_ and H_B_ were identified based on NOESY spectra of each compound (Table 1). An exception is compound **8**, which was found to degrade during the 2D measurements. Here, the proton assignment was based on the pattern found for the stable derivatives.

**Figure 2:**
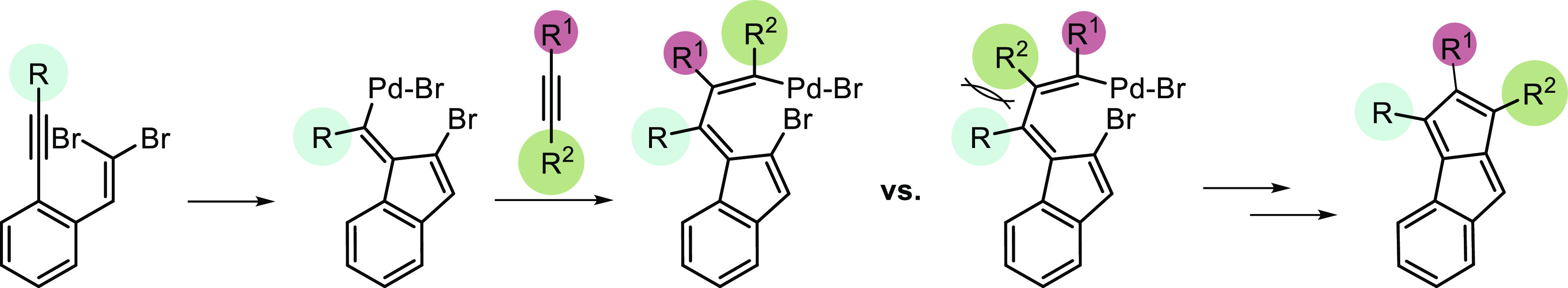
Suggested regioselectivity based on the steric hindrance in the proposed intermediates. R^1^ represents a less bulky group compared to R^2^.

**Figure 3:**
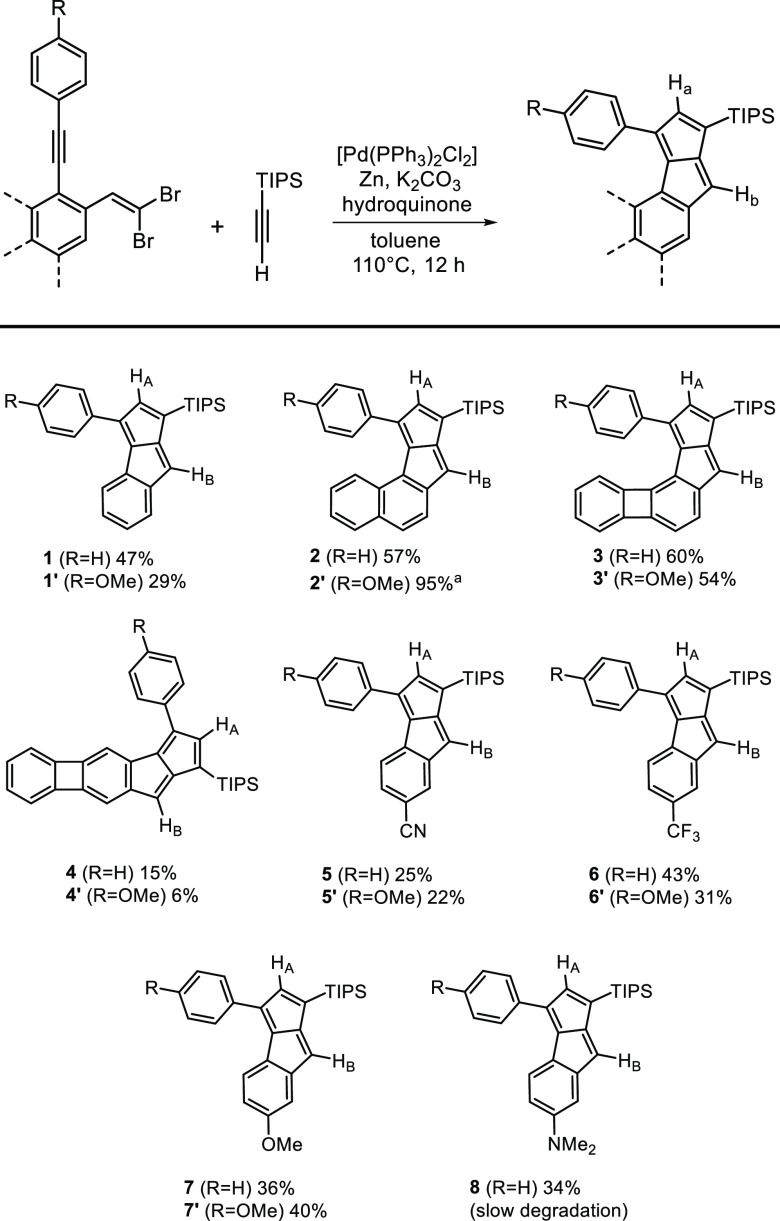
Prepared monobenzopentalene derivatives with two olefinic protons. ^a^ High yield might be due to inseparable impurity (∼5% based on ^1^H NMR).

**Table 1:** ^1^H NMR Shifts (500 MHz, CD_2_Cl_2_) of the Pentalenic Protons in **1**–**8** Identified via 2D-NOESY Measurements^a^

Entry	Compd	δ H_A_/ppm	δ H_B_/ppm
1	**1** (**1′**)	6.36 (6.35)	6.12 (6.10)
2	**2** (**2′**)	5.94 (5.95)	5.90 (5.90)
3	**3** (**3′**)	6.73 (6.72)	6.47 (6.44)
4	**4** (**4′**)	6.15 (6.14)	5.82 (5.81)
5	**5** (**5′**)	6.42 (6.42)	6.18 (6.17)
6	**6** (**6′**)	6.41 (6.41)	6.20 (6.18)
7	**7** (**7′**)	6.38 (6.37)	6.04 (6.03)
8	**8**	6.39	6.01

a In parentheses, the chemical shifts for compounds **1′–7′** (R = OMe) are shown.

The ^1^H NMR shift of H_B_ in compound **1** (6.12 ppm) was found to be closer to the shift of the corresponding H in **BP** (6.04 ppm) than to that in **TPBP** (6.35 ppm), which reflects the shielding effect of the proximal phenyl group in **TPBP**. Within the π-extended series **2** (**2′**)–**4** (**4′**), the chemical shifts follow the trend of the bond orders of the fused ring systems. It is now generally accepted that the paratropicity strength in an antiaromatic subunit increases if this subunit is fused to a bond with higher bond order.^10^ In compounds **2** and **4** the pentalene subunit is annelated with naphthalene and biphenylene, respectively, through their bonds of higher bond order. Accordingly, in these compounds H_A_ (5.94 ppm in **2**, 6.15 ppm in **4**) and H_B_ (5.90 ppm in **2**, 5.82 ppm in **4**) are shifted upfield compared to those in structure **3** (H_A_: 6.73 ppm, H_B_: 6.47 ppm) where the pentalene unit is fused to the bond of biphenylene with lower bond order.

A similar tendency was observed among the substituted derivatives **5**–**8**. The presence of electron-donating substituents OMe and NMe_2_ (**7**, **8**) on the fused benzene ring led to an upfield shift of H_A_ and H_B_ (**7**: 6.38/6.04 ppm, **8**: 6.39/6.01 ppm) compared to electron-withdrawing substituents CN (**5**, 6.42/6.18 ppm) and CF_3_ (**6**, 6.41/6.20 ppm). Replacing the phenyl substituent that was present in the series **1**–**7** to 4-methoxyphenyl in **1′**–**7′** did not influence the measured chemical shifts considerably.

To gain more insights into shielding effects, we attempted further variations of substituents on the pentalene subunit. First, we explored the effect of replacing the TIPS group with less bulky silyl (TMS, TES, TBDMS), alkyl (*t*Bu, *n*Bu, *n*Hex), or aryl (Ph, PMP) groups by changing the terminal acetylene reagent in the reaction. Unfortunately, alkyl-, aryl-, and TMS-acetylenes were not tolerated by the transformation. However, the reaction worked with TES-acetylene (**S52**, 15%) and TBDMS-acetylene (**S53**, 54%), although the products were found to degrade over time. Second, we investigated the effect of the R substituent in the starting material. Upon replacing Ph (or PMP) with TIPS, TMS, H, *t*Bu, *n*Bu, or *n*Hex in the dibromoolefin, no product formation was observed with TIPS-acetylene. Notably, changing the fused benzene ring to benzothiophene^5b^ did not lead to product formation either.

Next, optoelectronic properties of **1** and **TPBP** were compared (for further details, see section S1.4, Supporting Information). Molecule **1** had a similar UV–vis absorption spectrum to **TPBP**. However, its long-wavelength, low-intensity absorption maximum, indicative of the symmetry-forbidden HOMO–LUMO transition, is hypsochromically shifted and somewhat merged into the band between 350 and 450 nm (Figure 4a, inset). A similar tendency was found in the electrochemical measurements (CV) of the compounds (Figure 4b); while the electrochemically determined HOMO–LUMO gap of **1** was 2.2 eV, it was a lower value, 1.92 eV, for **TPBP**. Based on these experimental findings one might conclude that antiaromaticity is comparably lower in **1** than in **TPBP**. However, it is most likely that the pendant Ph-substituents are responsible for the measured differences. In compound **1** there is only one such substituent, while in **TPBP** there are three. Although these Ph-groups are noncoplanar with the conjugated benzopentalene cores, they have a non-negligible contribution to the frontier orbitals (Figure 4c). Hence, their presence led to an overall extension of the π-systems, which is reflected in the measurements. Furthermore, such π-extension could also affect antiaromaticity features.

**Figure 4:**
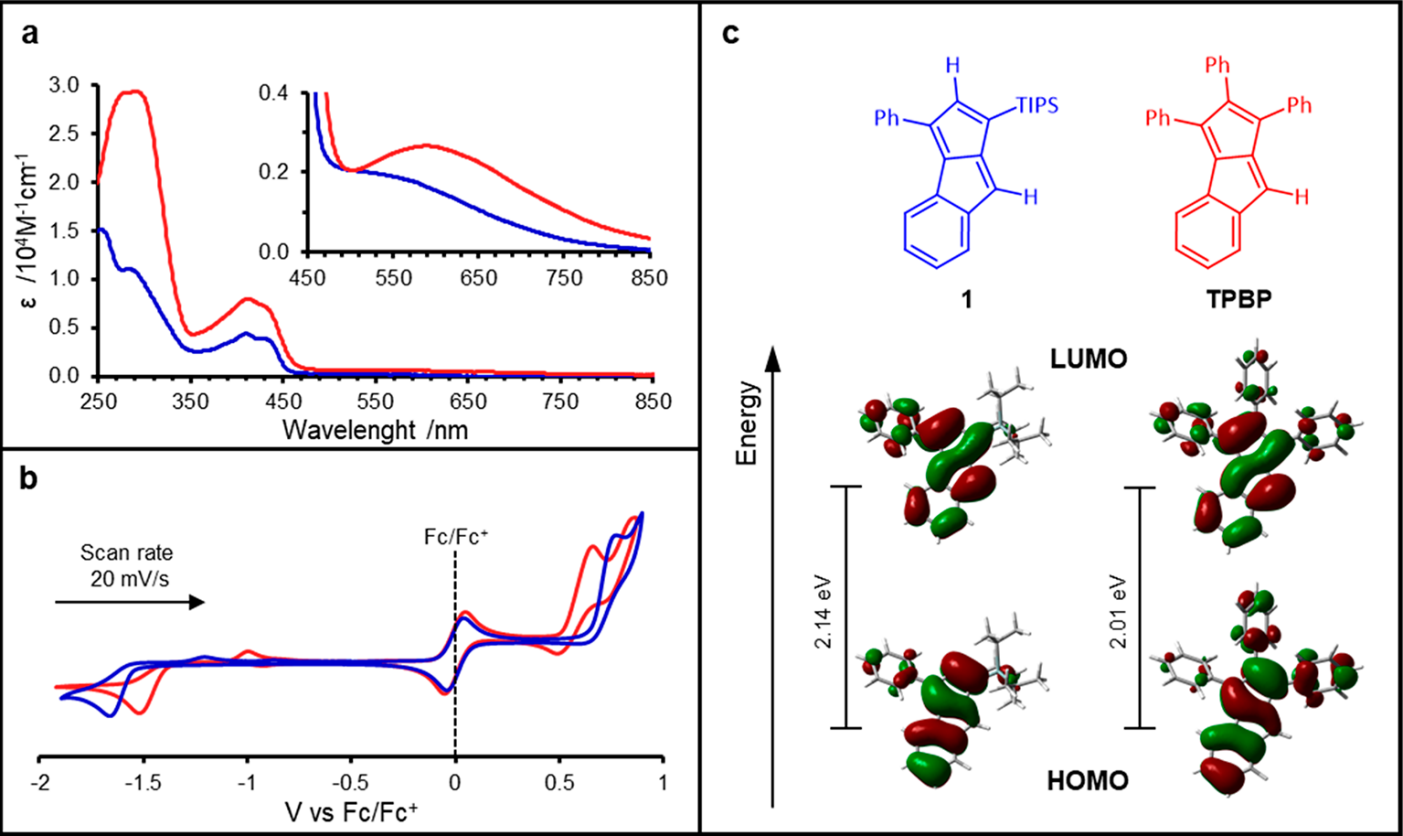
Optoelectronic measurements and calculated HOMOs and LUMOs of **1** (blue) and **TPBP** (red). (a) UV–vis spectra of the compounds in CH_2_Cl_2_ with the inset of the low-intensity absorptions. (b) Normalized cyclic voltammograms of **1** and **TPBP**. (c) Calculated HOMOs and LUMOs at 0.02 isosurface value, calculated HOMO–LUMO gap is shown in eV (for further details, see sections S1.4 and S2, Supporting Information).

To better understand the antiaromaticity of these compounds, NICS^15^ and ACID^16^ calculations were performed. For all geometry optimizations the B3LYP hybrid functional and the 6-311+G(d,p) basis set were used within the Gaussian 09 package^17^ (for further details, see section S2, Supporting Information). Decreasing NICS values were found with increasing number of Ph-substituents on the pentalene unit, which reflects the decrease of antiaromaticity in the order **BP** > **1** > **TPBP** (Figure 5). This is in agreement with the measured ^1^H NMR shifts of the Hs adjacent to the fused benzene rings. Moreover, within the studied series, the outer ring of pentalene was more antiaromatic than the inner ring based on NICS(1.0)_π,zz_ values, the asymmetry of which is documented in the literature^13^ possibly originating from the stronger stabilizing effect of the adjacent benzene ring.

**Figure 5:**
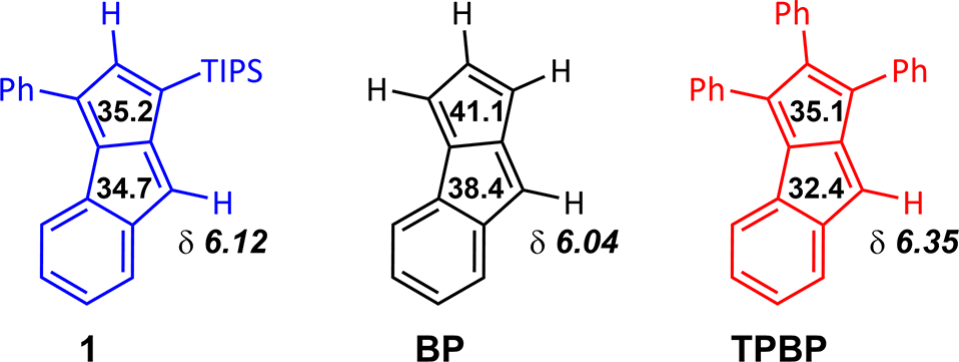
Comparison of ^1^H NMR shifts (in italics) and NICS(1.0)_π,zz_ (in the middle of the rings) values in **1**, **BP**, and **TPBP**.

In conclusion, a novel class of monoareno-pentalenes with two olefinic protons was described. These new derivatives were studied with experimental (NMR, UV–vis, CV) and theoretical (NICS, ACID) methods and were found to be stable antiaromatic compounds. Overall, these structures could be useful to explore magnetic (anti)aromaticity effects further on an experimental ground.

## Data Availability

The data underlying this study are available in the published article and its Supporting Information.

## Supporting Information Available

Detailed synthetic procedures, gram-scale synthesis of **1**, characterization, images of HRMS spectra, and images of ^1^H, ^13^C, and NOESY NMR spectra for all new compounds. Detailed procedures and results for UV–vis and CV measurements. Detailed procedures for theoretical characterization and Cartesian coordinates of calculated structures. (PDF)
