# Supplementary material for: Stable Monoareno-pentalenes with Two Olefinic Protons
Source: Org Lett. 2022 Dec 28;25(1):42–6. doi: 10.1021/acs.orglett.2c03752 (PMC9841605; doi:10.1021/acs.orglett.2c03752)
Supplement: Supplementary file 1 — ol2c03752_si_001.pdf [file ol2c03752_si_001.pdf]

Supporting Information

Stable monoareno-pentalenes with two olefinic protons

Péter J Mayer[1,2] and Gábor London[1]*

[1] MTA TTK Lendület Functional Organic Materials Research Group, Institute of Organic Chemistry, Research Centre for Natural Sciences, Magyar tudósok krt. 2., Budapest 1117, Hungary

[2] Institute of Chemistry, University of Szeged, Rerrich tér 1, Szeged 6720, Hungary

* london.gabor@ttk.hu

Table of Content

S1 Synthesis and characterization S3

S1.1 Preparation of starting materials and monoarenopentalenes S4

S1.1.1 Sonogashira products (**S9-S24**) S6

S1.1.2 Dibromoolefin products (**S25-40**) S12

S1.1.3 Monoareno-pentalenes (**1-8**) S18

S1.1.4 Gram scale synthesis of **1** S25

S1.2 Test reactions to access different substitution patterns S27

S1.2.1 Preparation and characterization of starting materials S27

S1.3 Assignation of olefinic protons from NOESY spectra S32

S1.4 Opto-electronic characterization and comparison S33

S1.4.1 UV-Vis measurements S33

S1.4.2 Cyclic voltammetry measurements S34

S2 Theoretical characterization S37

S2.1 Calculated transition energies S37

S2.2 Aromaticity indices S40

S3 NMR spectra S42

S4 HRMS spectra S117

S5 Cartesian coordinates and absolute electronic energies S144

References S146

S1 Synthesis and characterization

General Information

Commercial reagents, solvents, and catalysts (Aldrich, Fluorochem, VWR) were purchased as reagent grade and used without further purification. Solvents for extraction or column chromatography were of technical quality. Organic solutions were concentrated by rotary evaporation at 25−40 °C. Thin-layer chromatography was carried out on SiO_2-layered aluminium plates (60778-25EA, Fluka). Column chromatography was performed using SiO_2−60 (230−400-mesh ASTM, 0.040−0.063 mm from Merck) at 25 °C or Teledyne Isco CombiFlash Rf^{+} automated flash chromatographer with silica gel (25−40 µm, Zeochem). Room temperature refers to 25(±1) °C. NMR spectra were acquired on a Varian 500 NMR spectrometer, running at 500 and 126 MHz for ^{1}H and ^{13}C, respectively, and on a Varian 300 NMR spectrometer, running at 300 and 75 MHz for ^{1}H and ^{13}C, respectively. The residual solvent peaks were used as the internal reference. Chemical shifts (δ) are reported in ppm. The following abbreviations indicate the multiplicity in ^{1}H NMR spectra: s, singlet; d, doublet; t, triplet; q, quartet; and m, multiplet. ^{13}C NMR spectra were acquired on a broad-band decoupled mode. Structural assignments were made with additional information from NOESY, gHSQC, and gHMBC experiments.

Mass spectrometric measurements were performed using a Q-TOF Premier mass spectrometer (Waters Corporation, Milford, MA) in the positive electrospray ionisation (ESI) or atmospheric pressure chemical ionisation (APCI) mode.

IR spectra were recorded on a PerkinElmer Spectrum 3 FT-IR / FIR spectrometer in an ATR cell. UV spectra were recorded on a PerkinElmer Lambda 465 spectrophotometer. CV spectra were recorded with a Pine Research Wavenow^{xv} potentiostat.

S1.1 Preparation of starting materials and monoarenopentalenes

Starting materials were prepared in a similar manner as previously.[1,2] Acetylene moieties were introduced using Sonogashira reactions according to General Procedure 1 (**GP1**). *gem*-Dibromoolefins were prepared with Ramirez dibroomoolefination according to General Procedure 2 (**GP2**). The cascade reaction to prepare the target pentalene derivatives was developed based on the Pd-catalyzed pentalene formation reactions pioneered by Diederich and co-workers[3,4] and was carried out according to General Procedure 3 (**GP3**).

General Procedure for Sonogashira couplings (**GP1**):

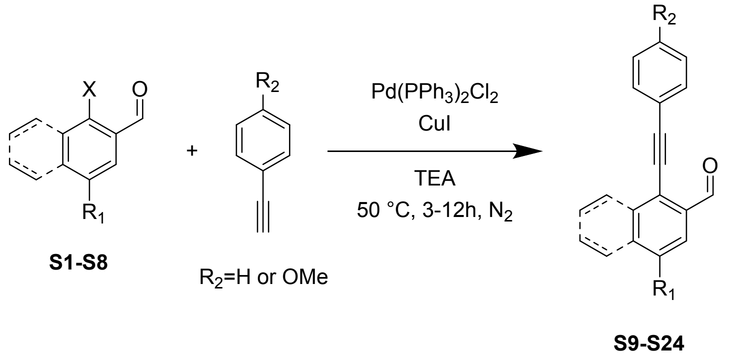

Figure S1. Scheme of Sonogashira reaction.

The ortohalo-formyl compound (1 equiv.) and the acetylene (1.05 equiv.) were dissolved in triethylamine (10 mL/mmol). The solution was added to a vial that contained $Pd(PPh_3)_2Cl_2$ (0.03 equiv.) and CuI (0.02 equiv.) under an inert atmosphere (N_2). The mixture was heated to 50 °C in an aluminium heating block and stirred for 3−12 h. After the reaction was completed (followed by TLC analysis), the mixture was diluted with EtOAc and filtered through a Celite plug. The solvent was evaporated *in vacuo*, and the crude product was further purified with column chromatography (SiO_2, hexanes/EtOAc).

General Procedure for *gem*-dibromoolefination (**GP2**):

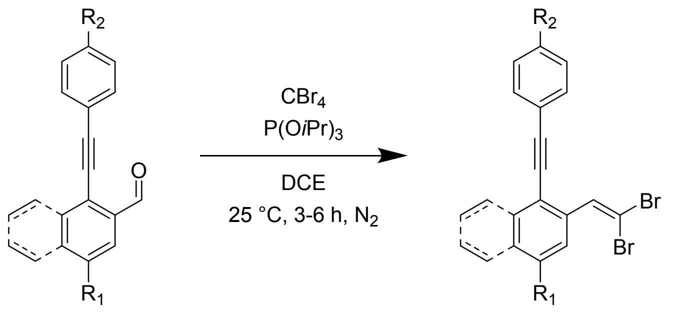

Figure S2. Scheme of Ramirez *gem*-dibromoolefination.

The product of **GP1** (1 equiv.) and CBr_4 (1.5 equiv.) were dissolved in 1,2-dichloroethane (DCE, 10 mL/mmol). After the solution was purged with N_2 for 10 min, P(O*i*Pr)$_3$ (3 equiv.) was added. The reaction was stirred at room temperature and was monitored with TLC, and upon completion (generally 3−6 h), the solvent was evaporated *in vacuo*. Subsequently, the crude product was further purified with column chromatography (SiO_2, hexanes/EtOAc).

General Procedure for carbopalladation cascade reaction (**GP3**):

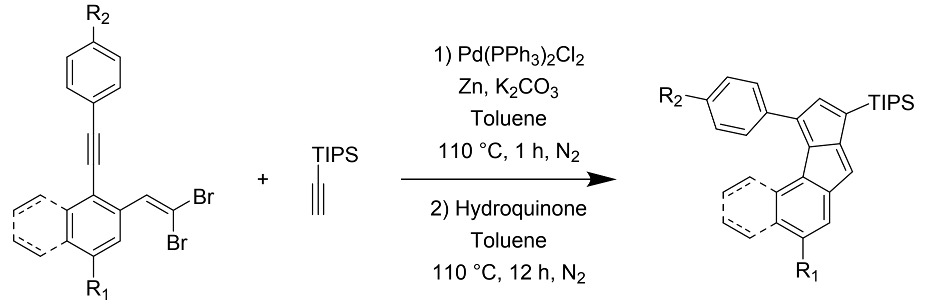

Figure S3. Scheme of carbopalladation cascade reaction for the formation of pentalene.

A vial was charged with the product of **GP2** (1 equiv.), $Pd(PPh_3)_2Cl_2$ (0.1 equiv.), Zn (1.5 equiv.), and K_2CO_3 (2 equiv.) and purged with N_2. After 10 min of purging with N_2, a solution of TIPS-acetylene (5 equiv.) in toluene (20 mL/mmol) was added to the vial. The mixture was heated to 110 °C in an aluminium heating block and stirred for 1 h. After the initial stirring, the mixture was cooled to room temperature, and hydroquinone (1.5 equiv.) and a further portion of TIPS-acetylene (5 equiv.) were added to the reaction. Subsequently, the vial was purged with N_2, and the solution was heated up to 110 °C and stirred for 12 h. The reaction was monitored with TLC, and upon completion, the mixture was filtered through a Celite plug.

This was followed by the removal of the solvent under reduced pressure and the further purification of the product with column chromatography (SiO_2, hexanes/EtOAc).

S1.1.1 Sonogashira products (**S9-S24**)

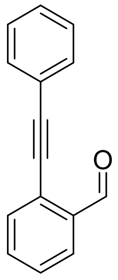

2-(Phenylethynyl)benzaldehyde (**S9**) was prepared according to **GP1** from 1.00 g (5.4 mmol) of 2-bromobenzaldehyde (**S1**). The column chromatography was performed on SiO_2 with a gradient from hexanes to hexanes/ethyl acetate = 9/1. After purification, 963 mg product (86%) was obtained as a yellowish solid.

^{1}H NMR (500 MHz, $CDCl_3$) δ = 10.66 (s, 1H), 7.96 (d, J = 7.7 Hz, 1H), 7.65 (d, J = 7.6 Hz, 1H), 7.62 – 7.54 (m, 3H), 7.46 (t, J = 7.6 Hz, 1H), 7.42 – 7.36 ppm (m, 3H).

$^{13}C\{^{1}H\}$ NMR (126 MHz, $CDCl_3$) δ = 191.7, 136.0, 133.9, 133.3, 131.8 (2), 129.2, 128.7, 128.6 (2), 127.4, 127.0, 122.5, 96.5, 85.0 ppm.

HRMS (ESI): *m/z*: $[M+H]^+$ calcd for $C_{15}H_{11}O^+$: 207.0809; found 207.0812

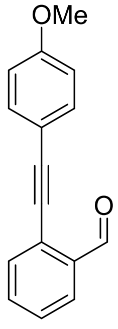

2-((4-Methoxyphenyl)ethynyl)benzaldehyde (**S10**) was prepared according to **GP1** from 2.00 g (10.8 mmol) of 2-bromobenzaldehyde (**S1**). The column chromatography was performed on SiO_2 with a gradient from hexanes to hexanes/ethyl acetate = 7/1. After purification, 2.53 g product (95%) was obtained as a yellow solid.

^{1}H NMR (500 MHz, $CDCl_3$) δ = 10.65 (s, 1H), 7.94 (ddd, J = 8.0, 1.5, 0.7 Hz, 1H), 7.62 (ddd, J = 7.8, 1.4, 0.7 Hz, 1H), 7.57 (td, J = 7.5, 1.4 Hz, 1H), 7.53 – 7.49 (m, 2H), 7.45 – 7.41 (m, 1H), 6.93 – 6.89 (m, 2H), 3.85 ppm (s, 3H).

$^{13}C\{^{1}H\}$ NMR (126 MHz, $CDCl_3$) δ = 192.0, 160.4, 135.9, 133.9, 133.4 (2), 133.2, 128.4, 127.5, 127.4, 114.6, 114.4 (2), 96.7, 83.9, 55.5 ppm.

HRMS (ESI): *m/z*: $[M+H]^+$ calcd for $C_{16}H_{13}O_2^+$: 237.0915; found 237.0919.

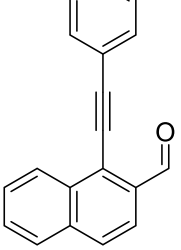

1-(Phenylethynyl)-2-naphthaldehyde (**S11**)[3] was prepared according to **GP1** from 500 mg (2.1 mmol) of 1-bromo-2-naphthaldehyde (**S2**). The column chromatography was performed on SiO_2 with a gradient from hexanes to hexanes/ethyl acetate = 9/1. After purification, 415 mg product (76%) was obtained as a yellowish solid.

^{1}H NMR (500 MHz, $CDCl_3$) δ = 10.89 (s, 1H), 8.63 – 8.58 (m, 1H), 7.99 (d, J = 8.6 Hz, 1H), 7.92 – 7.84 (m, 2H), 7.73 – 7.64 (m, 4H), 7.49 – 7.41 ppm (m, 3H).

$^{13}C\{^{1}H\}$ NMR (126 MHz, $CDCl_3$) δ = 192.2, 135.9, 134.4, 133.3, 131.9 (2), 129.5 (2), 129.0, 128.8 (2), 128.6, 127.8, 127.6, 127.4, 122.5, 122.2, 102.5, 83.1 ppm.

HRMS (ESI): *m/z*: $[M+H]^+$ calcd for $C_{19}H_{13}O^+$: 257.0966; found 257.0970.

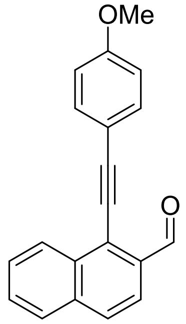

1-((4-Methoxyphenyl)ethynyl)-2-naphthaldehyde (**S12**)[3] was prepared according to **GP1** from 500 mg (2.1 mmol) of 1-bromo-2-naphthaldehyde (**S2**). The column chromatography was performed on SiO_2 with a gradient from hexanes to hexanes/ethyl acetate = 7/1. After purification, 492 mg product (81%) was obtained as a yellow solid.

^{1}H NMR (500 MHz, $CDCl_3$) δ =10.87 (s, 1H), 8.62 – 8.56 (m, 1H), 7.96 (d, J = 8.5 Hz, 1H), 7.90 – 7.85 (m, 1H), 7.82 (d, J = 8.6 Hz, 1H), 7.69 – 7.64 (m, 2H), 7.62 (d, J = 8.8 Hz, 2H), 6.96 (d, J = 6.8 Hz, 2H), 3.86 ppm (s, 3H).

$^{13}C\{^{1}H\}$ NMR (126 MHz, $CDCl_3$) δ = 192.3, 160.7, 135.9, 134.0, 133.5 (2), 133.2, 129.4, 128.6 (2), 128.1, 127.7, 127.4, 122.2, 114.55, 114.47 (2), 102.9, 82.1, 55.5 ppm.

HRMS (ESI): *m/z*: $[M+H]^+$ calcd for $C_{20}H_{15}O_2^+$: 287.1072; found 287.1079.

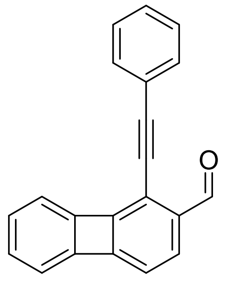

1-(Phenylethynyl)biphenylene-2-carbaldehyde (**S13**)[1] was prepared according to **GP1** from 200 mg (0.65 mmol) of 1-iodobiphenylene-2-carbaldehyde (**S3**, prepared according to literature[1]). The column chromatography was performed on SiO_2 with a gradient from hexanes to hexanes/ethyl acetate = 9/1. After purification, 128 mg product (70%) was obtained as a yellowish solid.

^{1}H NMR (300 MHz, $CDCl_3$) δ = 10.26 (s, 1H), 7.60 – 7.51 (m, 2H), 7.48 (d, J = 7.2 Hz, 1H), 7.44 – 7.33 (m, 3H), 6.89 (d, J = 9.5 Hz, 3H), 6.78 (d, J = 6.5 Hz, 1H), 6.71 ppm (d, J = 7.2 Hz, 1H).

$^{13}C\{^{1}H\}$ NMR (126 MHz, $CDCl_3$) δ = 190.1, 157.4, 154.8, 149.5, 149.1, 135.2, 132.3, 131.9 (2), 130.5, 129.8, 129.2, 128.7 (2), 122.7, 119.3, 119.0, 116.7, 97.7, 82.3 ppm.

HRMS (ESI): *m/z*: $[M+H]^+$ calcd for $C_{21}H_{13}O^+$: 281.0966; found 281.0970.

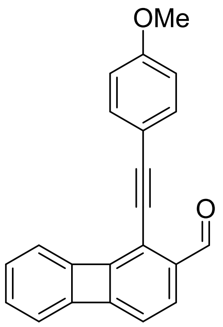

1-((4-Methoxyphenyl)ethynyl)biphenylene-2-carbaldehyde (**S14**)[1] was prepared according to **GP1** from 200 mg (0.65 mmol) of 1-iodobiphenylene-2-carbaldehyde (**S3**, prepared according to literature[1]). The column chromatography was performed on SiO_2 with a gradient from hexanes to hexanes/ethyl acetate = 7/1. After purification, 157 mg product (77%) was obtained as an orangish yellow solid.

^{1}H NMR (300 MHz, $CDCl_3$) δ = 10.26 (s, 1H), 7.52 – 7.42 (m, 3H), 6.96 – 6.82 (m, 5H), 6.76 (dt, J = 6.3, 1.2 Hz, 1H), 6.69 (d, J = 7.2 Hz, 1H), 3.84 ppm (s, 3H).

^{13}C{^{1}H} NMR (126 MHz, $CDCl_3$) δ = 190.3, 160.4, 157.4, 154.3, 149.6, 149.1, 135.1, 133.4 (2), 132.2, 130.4, 129.7, 119.2, 118.9, 116.4, 114.7, 114.3 (2), 114.1, 98.1, 81.2, 55.5 ppm.

HRMS (ESI): *m/z*: $[M+H]^+$ calcd for $C_{22}H_{15}O_2^+$: 311.1072; found 311.1082.

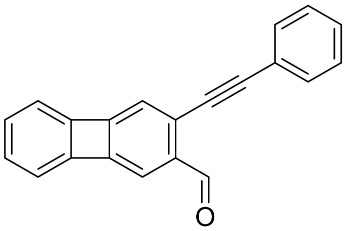

3-(Phenylethynyl)biphenylene-2-carbaldehyde (**S15**)[1] was prepared according to **GP1** from 100 mg (0.39 mmol) of 3-bromobiphenylene-2-carbaldehyde (**S4**, prepared according to literature[1]). The column chromatography was performed on SiO_2 with a gradient from hexanes to hexanes/ethyl acetate = 9/1. After purification, 59 mg product (55%) was obtained as a yellow solid.

^{1}H NMR (500 MHz, $CDCl_3$) δ = 10.46 (s, 1H), 7.56 – 7.51 (m, 2H), 7.38 (dd, J = 5.1, 1.9 Hz, 3H), 7.15 (s, 1H), 6.97 – 6.89 (m, 2H), 6.86 – 6.81 ppm (m, 3H).

^{13}C{^{1}H} NMR (126 MHz, $CDCl_3$) δ = 190.7, 156.9, 151.2, 149.9, 149.1, 137.5, 131.8 (2), 130.7, 130.6, 129.6, 129.2, 128.6 (2), 122.4, 120.3, 119.6, 119.2, 113.8, 97.5, 86.0 ppm.

HRMS (ESI): *m/z*: $[M+H]^+$ calcd for $C_{21}H_{13}O^+$: 281.0966; found 281.0975.

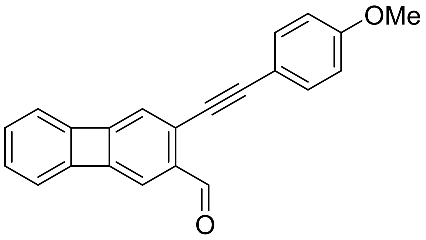

3-((4-methoxyphenyl)ethynyl)biphenylene-2-carbaldehyde (**S16**)[1] was prepared according to **GP1** from 100 mg (0.39 mmol) of 3-bromobiphenylene-2-carbaldehyde (**S4**, prepared according to literature[1]). The column chromatography was performed on SiO_2 with a gradient from hexanes to hexanes/ethyl acetate = 7/1. After purification, 61 mg product (51%) was obtained as an orange solid.

^{1}H NMR (500 MHz, $CDCl_3$) δ =10.44 (s, 1H), 7.49 – 7.45 (m, 2H), 7.13 (s, 1H), 6.96 – 6.88 (m, 4H), 6.85 – 6.80 (m, 3H), 3.84 ppm (s, 3H).

$^{13}C\{^1H\}$ NMR (126 MHz, $CDCl_3$) δ = 190.9, 160.5, 156.9, 150.8, 150.0, 149.2, 137.2, 133.4 (2), 131.3, 130.6, 129.6, 120.3, 119.6, 119.2, 114.5, 114.4 (2), 113.8, 98.0, 85.1, 55.5 ppm.

HRMS (ESI): *m/z*: $[M+H]^+$ calcd for $C_{22}H_{15}O_2^+$: 311.1072; found 311.1082.

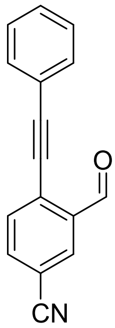

3-Formyl-4-(phenylethynyl)benzonitrile (**S17**) was prepared according to **GP1** from 200 mg (0.95 mmol) of 4-bromo-3-formylbenzonitrile (**S5**). The column chromatography was performed on SiO_2 with a gradient from hexanes to hexanes/ethyl acetate = 9/1. After purification, 135 mg product (61%) was obtained as a yellow solid.

^{1}H NMR (500 MHz, $CDCl_3$) δ = 10.58 (s, 1H), 8.16 (d, J = 1.3 Hz, 1H), 7.79 (dd, J = 8.1, 1.7 Hz, 1H), 7.72 (d, J = 8.1 Hz, 1H), 7.56 (d, J = 6.4 Hz, 2H), 7.47 – 7.35 ppm (m, 3H).

$^{13}C\{^1H\}$ NMR (126 MHz, $CDCl_3$) δ = 189.5, 136.3, 136.1, 134.1, 132.1 (2), 131.4, 130.8, 130.1, 128.8 (2), 121.5, 117.5, 112.5, 100.9, 83.8 ppm.

HRMS (ESI): *m/z*: $[M+H]^+$ calcd for $C_{16}H_{10}NO^+$: 232.0762; found 232.0767.

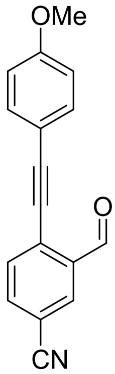

3-Formyl-4-((4-methoxyphenyl)ethynyl)benzonitrile (**S18**) was prepared according to **GP1** from 200 mg (0.95 mmol) of 4-bromo-3-formylbenzonitrile (**S5**). The column chromatography was performed on SiO_2 with a gradient from hexanes to hexanes/ethyl acetate = 7/1. After purification, 106 mg product (42%) was obtained as an orange solid.

^{1}H NMR (500 MHz, $CDCl_3$) δ = 10.58 (s, 1H), 8.17 (d, J = 1.7 Hz, 1H), 7.78 (dd, J = 8.1, 1.8 Hz, 1H), 7.69 (d, J = 8.1 Hz, 1H), 7.51 (d, J = 8.8 Hz, 2H), 6.92 (d, J = 8.8 Hz, 2H), 3.85 ppm (s, 3H).

$^{13}C\{^1H\}$ NMR (126 MHz, $CDCl_3$) δ = 189.7, 161.2, 136.1, 136.0, 133.9, 133.8 (2), 131.4, 131.4, 117.7, 114.6 (2), 113.5, 112.0, 101.6, 83.0, 55.6 ppm.

HRMS (ESI): *m/z*: $[M+H]^+$ calcd for $C_{17}H_{12}NO_2^+$: 262.0868; found 262.0877.

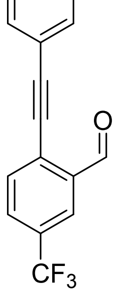

2-(Phenylethynyl)-5-(trifluoromethyl)benzaldehyde (**S19**) was prepared according to **GP1** from 200 mg (0.79 mmol) of 2-bromo-

5-(trifluoromethyl)benzaldehyde (**S6**). The column chromatography was performed on SiO_2 with a gradient from hexanes to hexanes/ethyl acetate = 9/1. After purification, 159 mg product (73%) was obtained as a yellow oil.

^{1}H NMR (500 MHz, $CDCl_3$) δ = 10.66 (s, 1H), 8.21 (s, 1H), 7.82 (d, J = 8.1 Hz, 1H), 7.77 (d, J = 8.2 Hz, 1H), 7.59 (dd, J = 7.6, 1.9 Hz, 2H), 7.47 – 7.38 ppm (m, 3H).

^{13}C{^{1}H} NMR (126 MHz, $CDCl_3$) δ = 190.3, 136.2, 133.9, 132.0 (2), 130.1 (q, J = 3.3 Hz), 129.8, 128.8 (2), 124.6 (q, J = 3.7 Hz), 121.8, 99.1, 83.9 ppm.

HRMS (ESI): *m/z*: $[M+H]^+$ calcd for $C_{16}H_{10}OF_3^+$: 275.0683; found 275.0689.

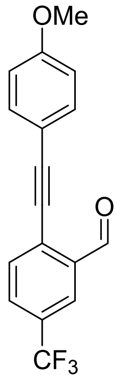

2-((4-Methoxyphenyl)ethynyl)-5-(trifluoromethyl)benzaldehyde (**S20**) was prepared according to **GP1** from 200 mg (0.79 mmol) of 2-bromo-5-(trifluoromethyl)benzaldehyde (**S6**). The column chromatography was performed on SiO_2 with a gradient from hexanes to hexanes/ethyl acetate = 9/1. After purification, 158 mg product (66%) was obtained as a yellow solid.

^{1}H NMR (500 MHz, $CDCl_3$) δ = 10.65 (s, 1H), 8.21 – 8.16 (m, 1H), 7.79 (dd, J = 8.2, 2.0 Hz, 1H), 7.72 (d, J = 8.2 Hz, 1H), 7.52 (d, J = 8.7 Hz, 2H), 6.92 (d, J = 8.8 Hz, 2H), 3.86 ppm (s, 3H).

^{13}C{^{1}H} NMR (126 MHz, $CDCl_3$) δ = 190.4, 160.9, 135.9, 133.7, 133.6 (2), 130.7, 130.5, 130.2, 130.0 (q, J = 3.6 Hz), 124.6, 124.5 (q, J = 3.9 Hz), 122.8, 114.5 (2), 113.8, 99.6, 83.0, 55.5 ppm.

HRMS (ESI): *m/z*: $[M+H]^+$ calcd for $C_{17}H_{12}O_2F_3^+$: 305.0789; found 305.0800.

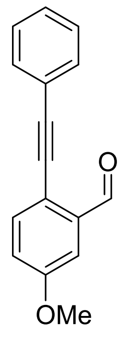

5-Methoxy-2-(phenylethynyl)benzaldehyde (**S21**) was prepared according to **GP1** from 500 mg (2.3 mmol) of 2-bromo-5-methoxybenzaldehyde (**S7**). The column chromatography was performed on SiO_2 with a gradient from hexanes to hexanes/ethyl acetate = 9/1. After purification, 506 mg product (92%) was obtained as a yellow solid.

^{1}H NMR (500 MHz, $CDCl_3$) δ = 10.62 (s, 1H), 7.57 (d, J = 8.6 Hz, 1H), 7.56 – 7.52 (m, 2H), 7.44 (d, J = 2.8 Hz, 1H), 7.37 (dd, J = 4.9, 1.9 Hz, 3H), 7.15 (dd, J = 8.5, 2.8 Hz, 1H), 3.89 ppm (s, 3H).

^{13}C{^{1}H} NMR (126 MHz, $CDCl_3$) δ = 191.7, 160.0, 137.4, 134.7, 131.6 (2), 128.9, 128.6 (2), 122.8, 121.8, 119.7, 110.0, 95.0, 85.0, 55.8 ppm.

HRMS (ESI): *m/z*: $[M+H]^+$ calcd for $C_{16}H_{13}O_2^+$: 237.0915; found 237.0914.

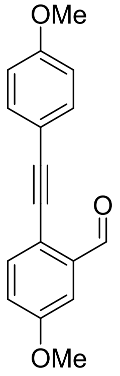

5-Methoxy-2-((4-methoxyphenyl)ethynyl)benzaldehyde (**S22**) was prepared according to **GP1** from 500 mg (2.3 mmol) of 2-bromo-5-methoxybenzaldehyde (**S7**). The column chromatography was performed on SiO_2 with a gradient from hexanes to hexanes/ethyl acetate = 7/1. After purification, 570 mg product (92%) was obtained as a reddish-orange solid.

^1H NMR (500 MHz, $CDCl_3$) δ = 10.61 (s, 1H), 7.54 (d, *J* = 8.6 Hz, 1H), 7.50 – 7.46 (m, 2H), 7.42 (d, *J* = 2.8 Hz, 1H), 7.13 (dd, *J* = 8.6, 2.8 Hz, 1H), 6.93 – 6.88 (m, 2H), 3.88 (s, 3H), 3.84 ppm (s, 3H).

$^{13}C\{^1H\}$ NMR (126 MHz, $CDCl_3$) δ = 191.9, 160.2, 159.7, 137.2, 134.5, 133.2 (2), 121.9, 120.3, 114.9, 114.3 (2), 109.9, 95.1, 83.7, 55.8, 55.5 ppm.

HRMS (ESI): *m/z*: $[M+H]^+$ calcd for $C_{17}H_{15}O_3^+$: 267.1021; found 267.1020.

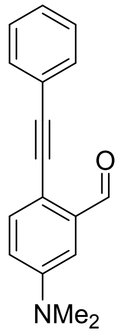

5-(Dimethylamino)-2-(phenylethynyl)benzaldehyde (**S23**) was prepared according to **GP1** from 200 mg (0.88 mmol) of 2-bromo-5-(dimethylamino)benzaldehyde (**S8**). The column chromatography was performed on SiO_2 with a gradient from hexanes (1% TEA) to hexanes (1% TEA)/ethyl acetate = 9/1. After purification, 181 mg product (83%) was obtained as a yellow solid.

^1H NMR (500 MHz, $CDCl_3$) δ = 10.62 (s, 1H), 7.53 (dd, *J* = 7.8, 1.7 Hz, 2H), 7.50 (d, *J* = 8.7 Hz, 1H), 7.38 – 7.32 (m, 3H), 7.20 (d, *J* = 2.9 Hz, 1H), 6.89 (dd, *J* = 8.7, 2.9 Hz, 1H), 3.04 ppm (s, 6H).

$^{13}C\{^1H\}$ NMR (126 MHz, $CDCl_3$) δ = 192.7, 150.1, 136.7, 134.4, 131.4 (2), 128.5, 128.3, 123.5 (2), 117.4, 114.0, 109.2, 93.8, 86.2, 40.2 ppm.

HRMS (ESI): *m/z*: $[M+H]^+$ calcd for $C_{17}H_{16}NO^+$: 250.1231; found 250.1238.

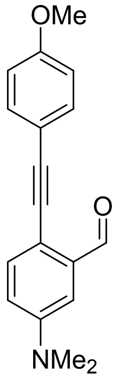

5-(Dimethylamino)-2-((4-methoxyphenyl)ethynyl)benzaldehyde (**S24**) was prepared according to **GP1** from 200 mg (0.88 mmol) of 2-bromo-5-(dimethylamino)benzaldehyde (**S8**). The column chromatography was performed on SiO_2 with a gradient from hexanes (1% TEA) to hexanes (1% TEA)/ethyl acetate = 7/1. After purification, 229 mg product (94%) was obtained as an orange solid.

^{1}H NMR (500 MHz, $CDCl_3$) δ = 10.60 (s, 1H), 7.46 (dd, J = 8.7, 6.5 Hz, 3H), 7.19 (d, J = 2.9 Hz, 1H), 6.91 – 6.86 (m, 3H), 3.82 (s, 3H), 3.03 ppm (s, 6H).

$^{13}C\{^1H\}$ NMR (126 MHz, $CDCl_3$) δ = 192.8, 159.8, 149.9, 136.5, 134.2, 132.9 (2), 117.5, 115.6, 114.6, 114.2 (2), 109.1, 93.8, 84.8, 55.4, 40.3 ppm.

HRMS (ESI): *m/z*: $[M+H]^+$ calcd for $C_{18}H_{18}NO_2^+$: 280.1337; found 280.1339.

S1.1.2 Dibromoolefin products (**S25-40**)

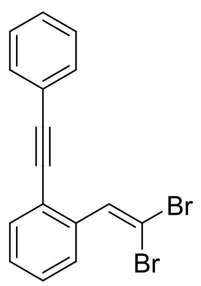

1-(2,2-Dibromovinyl)-2-(phenylethynyl)benzene (**S25**) was prepared according to **GP2** from 800 mg (3.9 mmol) of **S9**. The column chromatography was performed on SiO_2 with a gradient from hexanes to hexanes/ethyl acetate = 12/1. After purification, 1.13 g product (81%) was obtained as an orange oil (solidified in freezer).

^1H NMR (500 MHz, $CDCl_3$) δ = 7.87 (s, 1H), 7.80 – 7.75 (m, 1H), 7.59 – 7.53 (m, 3H), 7.41 – 7.31 ppm (m, 5H).

$^{13}C\{^1H\}$ NMR (126 MHz, $CDCl_3$) δ = 137.5, 136.1, 132.3, 131.8 (2), 128.8, 128.6, 128.4 (2), 128.23, 128.21, 123.1, 123.0, 95.3, 91.5, 87.4 ppm.

HRMS (APCI): *m/z*: $[M+H]^+$ calcd for $C_{16}H_{11}Br_2^+$: 360.9227; found 360.9212.

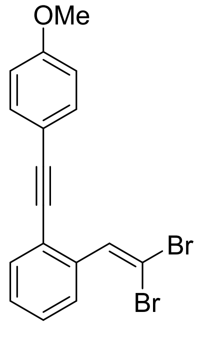

1-(2,2-Dibromovinyl)-2-((4-methoxyphenyl)ethynyl)benzene (**S26**) was prepared according to **GP2** from 2.00 g (8.5 mmol) of **S10**. The column chromatography was performed on SiO_2 with a gradient from hexanes to hexanes/ethyl acetate = 9/1. After purification, 1.88 g product (57%) was obtained as a red solid.

^1H NMR (500 MHz, $CDCl_3$) δ = 7.86 (s, 1H), 7.79 – 7.74 (m, 1H), 7.55 – 7.51 (m, 1H), 7.51 – 7.47 (m, 2H), 7.36 – 7.29 (m, 2H), 6.93 – 6.88 (m, 2H), 3.84 ppm (s, 3H).

$^{13}C\{^1H\}$ NMR (126 MHz, $CDCl_3$) δ = 160.1, 137.2, 136.2, 133.2 (2), 132.0, 128.4, 128.2, 127.8, 123.4, 115.2, 114.3 (2), 95.5, 86.2, 55.5 ppm.

HRMS (ESI): *m/z*: $[M+H]^+$ calcd for $C_{17}H_{13}OBr_2^+$: 390.9333; found 390.9351.

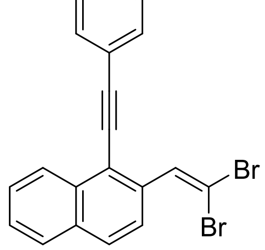

2-(2,2-Dibromovinyl)-1-(phenylethynyl)naphthalene (**S27**)[3] was prepared according to **GP2** from 300 mg (1.2 mmol) of **S11**. The column chromatography was performed on SiO_2 with a gradient from hexanes to

hexanes/ethyl acetate = 12/1. After purification, 400 mg product (83%) was obtained as an orangish yellow solid.

^{1}H NMR (500 MHz, $CDCl_3$) δ = 8.47 (d, J = 8.2 Hz, 1H), 8.10 (s, 1H), 7.88 (d, J = 8.7 Hz, 1H), 7.87 – 7.81 (m, 2H), 7.68 (dd, J = 7.8, 1.7 Hz, 2H), 7.61 (t, J = 6.9 Hz, 1H), 7.56 (t, J = 6.8 Hz, 1H), 7.46 – 7.38 ppm (m, 3H).

^{13}C{^{1}H} NMR (126 MHz, $CDCl_3$) δ = 136.8, 136.1, 133.3, 132.8, 131.8 (2), 129.0, 128.7 (2), 128.4, 128.2, 127.4, 127.2, 126.7, 125.2, 123.2, 120.6, 101.2, 91.9, 85.7 ppm.

HRMS (ESI): *m/z*: [M+H]$^+$ calcd for $C_{20}H_{13}Br_2^+$: 410.9383; found 410.9366.

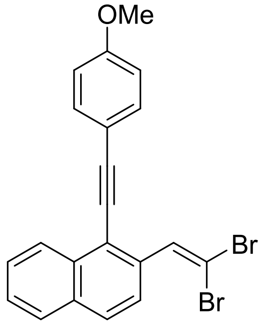

2-(2,2-Dibromovinyl)-1-((4-methoxyphenyl)ethynyl)naphthalene (**S28**)[3] was prepared according to **GP2** from 300 mg (1.1 mmol) of **S12**. The column chromatography was performed on SiO_2 with a gradient from hexanes to hexanes/ethyl acetate = 9/1. After purification, 329 mg product (71%) was obtained as an orangish yellow solid.

^{1}H NMR (500 MHz, $CDCl_3$) δ = 8.46 (d, J = 8.3 Hz, 1H), 8.10 (s, 1H), 7.87 (d, J = 8.7 Hz, 1H), 7.84 (d, J = 8.2 Hz, 1H), 7.80 (d, J = 8.7 Hz, 1H), 7.61 (d, J = 8.7 Hz, 2H), 7.60 – 7.52 (m, 2H), 6.96 (d, J = 8.7 Hz, 2H), 3.87 ppm (s, 3H).

^{13}C{^{1}H} NMR (126 MHz, $CDCl_3$) δ = 160.3, 136.9, 135.7, 133.3 (2), 133.2, 132.8, 128.3, 127.8, 127.3, 127.2, 126.8, 125.1, 121.0, 115.3, 114.4 (2), 101.5, 91.6, 84.5, 55.5 ppm.

HRMS (ESI): *m/z*: [M+H]$^+$ calcd for $C_{21}H_{15}OBr_2^+$: 440.9489; found 440.9510.

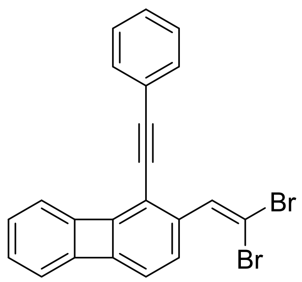

2-(2,2-Dibromovinyl)-1-(phenylethynyl)biphenylene (**S29**)[1] was prepared according to **GP2** from 50 mg (0.18 mmol) of **S13**. The column chromatography was performed on SiO_2 with a gradient from hexanes to hexanes/ethyl acetate = 12/1. After purification, 66 mg product (86%) was obtained as a yellow solid.

^{1}H NMR (300 MHz, $CDCl_3$) δ = 7.63 (s, 1H), 7.58 – 7.50 (m, 2H), 7.38 (t, J = 3.2 Hz, 3H), 7.21 (d, J = 7.3 Hz, 1H), 6.83 (s, 3H), 6.70 (d, J = 3.6 Hz, 1H), 6.61 ppm (d, J = 7.3 Hz, 1H).

^{13}C{^{1}H} NMR (126 MHz, $CDCl_3$) δ = 153.8, 151.2, 150.2, 150.0, 136.1, 135.6, 131.8 (2), 129.4, 129.2, 128.9, 128.7, 128.6 (2), 123.0, 118.4, 118.2, 116.5, 112.4, 97.1, 90.9, 83.9 ppm.

HRMS (APCI): *m/z*: [M]$^{\bullet+}$ calcd for $C_{22}H_{20}Br_2^{\bullet+}$: 433.9305; found 433.9311.

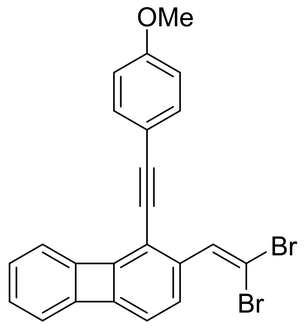

2-(2,2-Dibromovinyl)-1-((4-methoxyphenyl)ethynyl)biphenylene (**S30**)[1] was prepared according to **GP2** from 50 mg (0.16 mmol) of **S14**. The column chromatography was performed on SiO_2 with a gradient from hexanes to hexanes/ethyl acetate = 9/1. After purification, 47 mg product (63%) was obtained as an orangish yellow solid.

^{1}H NMR (300 MHz, $CDCl_3$) δ = 7.63 (s, 1H), 7.47 (d, J = 8.8 Hz, 2H), 7.20 (d, J = 7.2 Hz, 1H), 6.90 (d, J = 8.7 Hz, 2H), 6.81 (d, J = 2.4 Hz, 3H), 6.69 (d, J = 3.0 Hz, 1H), 6.59 (d, J = 7.3 Hz, 1H), 3.84 ppm (s, 3H).

$^{13}C\{^{1}H\}$ NMR (126 MHz, $CDCl_3$) δ = 160.2, 153.3, 151.2, 150.2, 150.1, 136.0, 135.7, 133.3 (2), 129.3, 129.1, 128.6, 118.3, 118.1, 116.3, 115.1, 114.3 (2), 112.8, 97.3, 90.7, 82.7, 55.5 ppm.

HRMS (APCI): *m/z*: $[M+H]^+$ calcd for $C_{23}H_{15}OBr_2^+$: 464.9489; found 464.9490.

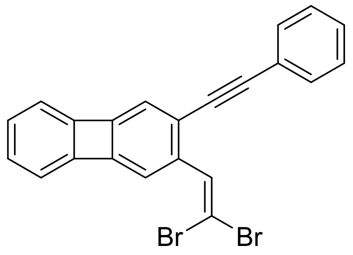

2-(2,2-Dibromovinyl)-3-(phenylethynyl)biphenylene (**S31**)[1] was prepared according to **GP2** from 59 mg (0.21 mmol) of **S15**. The column chromatography was performed on SiO_2 with a gradient from hexanes to hexanes/ethyl acetate = 12/1. After purification, 71 mg product (77%) was obtained as a yellow solid.

^{1}H NMR (500 MHz, $CDCl_3$) δ = 7.77 (s, 1H), 7.54 – 7.49 (m, 2H), 7.39 – 7.33 (m, 3H), 7.11 (s, 1H), 6.87 – 6.80 (m, 2H), 6.78 (s, 1H), 6.76 – 6.70 ppm (m, 2H).

$^{13}C\{^{1}H\}$ NMR (126 MHz, $CDCl_3$) δ = 150.9, 150.3, 150.2, 138.2, 136.4, 131.7 (2), 129.32, 129.27, 128.8, 128.6 (2), 123.1, 122.9, 120.0, 118.6, 118.4, 116.6, 96.5, 90.9, 88.3 ppm.

HRMS (APCI): *m/z*: $[M+H]^+$ calcd for $C_{22}H_{13}Br_2^+$: 434.9383; found 434.9380.

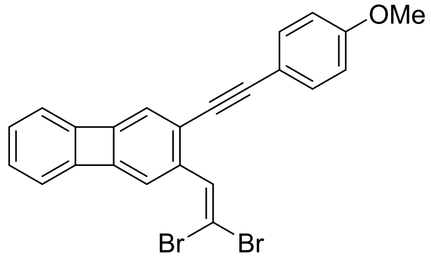

2-(2,2-Dibromovinyl)-3-((4-methoxyphenyl)ethynyl) biphenylene (**S32**)[1] was prepared according to **GP2** from 61 mg (0.20 mmol) of **S16**. The column chromatography was performed on SiO_2 with a gradient from hexanes to hexanes/ethyl acetate = 9/1. After purification, 70 mg product (76%) was obtained as a yellow solid.

^{1}H NMR (500 MHz, $CDCl_3$) δ = 7.77 (s, 1H), 7.45 (d, J = 8.7 Hz, 2H), 7.11 (s, 1H), 6.89 (d, J = 8.8 Hz, 2H), 6.86 – 6.79 (m, 2H), 6.75 (s, 1H), 6.74 – 6.69 (m, 2H), 3.84 ppm (s, 3H).

$^{13}C\{^{1}H\}$ NMR (126 MHz, $CDCl_3$) δ = 160.1, 150.9, 150.4, 150.3, 150.2, 137.8, 136.5, 133.2 (2), 129.3, 129.2, 123.3, 119.9, 118.5, 118.4, 116.6, 115.2, 114.3 (2), 96.8, 90.6, 87.2, 55.5 ppm.

HRMS (APCI): *m/z*: $[M+H]^+$ calcd for $C_{23}H_{15}OBr_2^+$: 464.9489; found 464.9511.

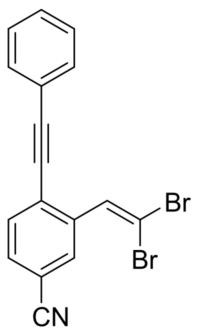

3-(2,2-Dibromovinyl)-4-(phenylethynyl)benzonitrile (S33) was prepared according to **GP2** from 100 mg (0.43 mmol) of **S17**. The column chromatography was performed on SiO_2 with a gradient from hexanes to hexanes/ethyl acetate = 9/1. After purification, 110 mg product (66%) was obtained as an orange solid.

^{1}H NMR (500 MHz, $CDCl_3$) δ = 8.04 (s, 1H), 7.79 (s, 1H), 7.63 (d, J = 8.0 Hz, 1H), 7.61 – 7.54 (m, 3H), 7.44 – 7.37 ppm (m, 3H).

$^{13}C\{^{1}H\}$ NMR (126 MHz, $CDCl_3$) δ = 138.3, 134.0, 132.8, 132.0 (2), 131.8, 131.4, 129.7, 128.8 (2), 127.6, 122.1, 118.3, 111.7, 99.6, 94.3, 86.0 ppm.

HRMS (ESI): *m/z*: $[M+H]^+$ calcd for $C_{17}H_{10}NBr_2^+$: 385.9179; found 385.9180.

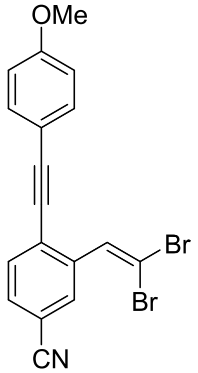

3-(2,2-Dibromovinyl)-4-((4-methoxyphenyl)ethynyl)benzonitrile (S34) was prepared according to **GP2** from 200 mg (0.77 mmol) of **S18**. The column chromatography was performed on SiO_2 with a gradient from hexanes to hexanes/ethyl acetate = 7/1. After purification, 51 mg product (16%) was obtained as a red solid.

Note: The reaction did not get complete in 16 h, so we terminated it and recovered 81 mg of starting material.

^{1}H NMR (500 MHz, $CDCl_3$) δ = 8.03 (s, 1H), 7.78 (s, 1H), 7.61 – 7.55 (m, 2H), 7.50 (d, J = 8.8 Hz, 2H), 6.92 (d, J = 8.8 Hz, 2H), 3.85 ppm (s, 3H).

$^{13}C\{^{1}H\}$ NMR (126 MHz, $CDCl_3$) δ = 160.8, 138.0, 134.2, 133.6 (2), 132.5, 131.8, 131.4, 128.0, 118.4, 114.5 (2), 114.1, 111.2, 100.1, 94.0, 85.2, 55.5 ppm.

HRMS (APCI): *m/z*: $[M]^{\bullet+}$ calcd for $C_{18}H_{11}NOBr_2^{\bullet+}$: 414.9207; found 414.9204.

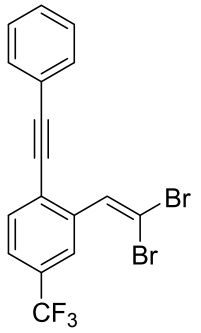

2-(2,2-Dibromovinyl)-1-(phenylethynyl)-4-(trifluoromethyl)benzene (**S35**) was prepared according to **GP2** from 100 mg (0.37 mmol) of **S19**. The column chromatography was performed on SiO_2 with a gradient from hexanes to hexanes/ethyl acetate = 12/1. After purification, 77 mg product (49%) was obtained as a yellow oil.

^{1}H NMR (500 MHz, $CDCl_3$) δ = 8.04 (s, 1H), 7.85 (s, 1H), 7.65 (d, J = 8.1 Hz, 1H), 7.61 – 7.55 (m, 3H), 7.43 – 7.37 ppm (m, 3H).

$^{13}C\{^1H\}$ NMR (126 MHz, $CDCl_3$) δ = 138.0, 134.8, 132.6, 131.9 (2), 130.2, 129.9, 129.4, 128.7 (2), 126.5, 125.3 (q, J = 4.0 Hz), 125.0 (q, J = 3.7 Hz), 122.4, 97.7, 93.6, 86.2 ppm.

HRMS (APCI): *m/z*: $[M+H]^+$ calcd for $C_{17}H_{10}F_3Br_2^+$: 428.9101; found 428.9114.

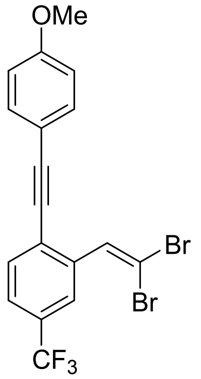

2-(2,2-Dibromovinyl)-1-((4-methoxyphenyl)ethynyl)-4-(trifluoromethyl) benzene (**S36**) was prepared according to **GP2** from 100 mg (0.33 mmol) of **S20**. The column chromatography was performed on SiO_2 with a gradient from hexanes to hexanes/ethyl acetate = 9/1. After purification, 83 mg product (55%) was obtained as a red solid.

^{1}H NMR (500 MHz, $CDCl_3$) δ = 8.02 (s, 1H), 7.84 (s, 1H), 7.62 (d, J = 8.2 Hz, 1H), 7.56 (d, J = 8.2 Hz, 1H), 7.51 (d, J = 8.8 Hz, 2H), 6.92 (d, J = 8.9 Hz, 2H), 3.85 ppm (s, 3H).

$^{13}C\{^1H\}$ NMR (126 MHz, $CDCl_3$) δ = 160.6, 137.7, 134.9, 133.5 (2), 132.3, 129.7, 126.9, 125.2 (q, J = 4.2 Hz), 125.0 (q, J = 3.7 Hz), 114.4 (2), 98.1, 93.3, 85.2, 55.5.

HRMS (APCI): *m/z*: $[M+H]^+$ calcd for $C_{18}H_{12}OF_3Br_2^+$: 458.9206; found 458.9201.

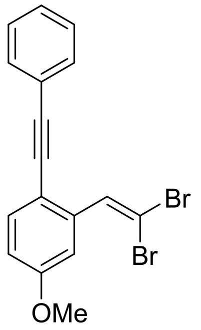

2-(2,2-Dibromovinyl)-4-methoxy-1-(phenylethynyl)benzene (**S37**) was prepared according to **GP2** from 300 mg (1.3 mmol) of **S21**. The column chromatography was performed on SiO_2 with a gradient from hexanes to hexanes/ethyl acetate = 9/1. After purification, 460 mg product (92%) was obtained as a pink solid.

^{1}H NMR (500 MHz, $CDCl_3$) δ = 7.85 (s, 1H), 7.55 – 7.51 (m, 2H), 7.48 (d, J = 8.6 Hz, 1H), 7.39 – 7.33 (m, 4H), 6.88 (dd, J = 8.6, 2.7 Hz, 1H), 3.85 ppm (s, 3H).

$^{13}C\{^1H\}$ NMR (126 MHz, $CDCl_3$) δ = 159.4, 138.7, 135.9, 133.5, 131.6 (2), 128.5 (2), 128.4, 115.3, 114.6, 113.6, 93.9, 91.6, 87.3, 55.6 ppm.

HRMS (APCI): *m/z*: $[M+H]^+$ calcd for $C_{17}H_{13}OBr_2^+$: 390.9333; found 390.9329.

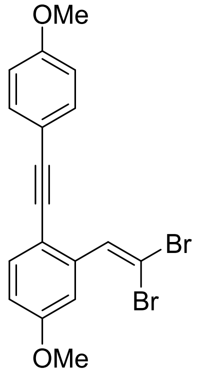

2-(2,2-Dibromovinyl)-4-methoxy-1-((4-methoxyphenyl)ethynyl)benzene (**S38**) was prepared according to **GP2** from 300 mg (1.1 mmol) of **S22**. The column chromatography was performed on SiO_2 with a gradient from hexanes to hexanes/ethyl acetate = 7/1. After purification, 340 mg product (71%) was obtained as a pink solid.

^{1}H NMR (500 MHz, $CDCl_3$) δ = 7.85 (s, 1H), 7.49 – 7.43 (m, 3H), 7.33 (d, *J* = 2.6 Hz, 1H), 6.91 – 6.85 (m, 3H), 3.84 (s, 3H), 3.84 ppm (s, 3H).

$^{13}C\{^1H\}$ NMR (126 MHz, $CDCl_3$) δ = 159.8, 159.1, 138.4, 136.0, 133.3, 133.0 (2), 115.7, 115.5, 114.6, 114.2 (2), 113.5, 93.9, 91.3, 86.0, 55.6, 55.4 ppm.

HRMS (ESI): *m/z*: $[M+H]^+$ calcd for $C_{18}H_{15}O_2Br_2^+$: 420.9438; found 420.9453.

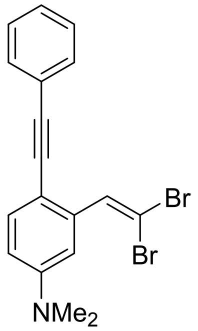

3-(2,2-Dibromovinyl)-N,N-dimethyl-4-(phenylethynyl)aniline (**S39**) was prepared according to **GP2** from 100 mg (0.40 mmol) of **S23**. The column chromatography was performed on SiO_2 with a gradient from hexanes (1% TEA) to hexanes (1% TEA)/ethyl acetate = 9/1. After purification, 71 mg product (44%) was obtained as a greenish yellow solid.

^{1}H NMR (500 MHz, $CDCl_3$) δ = 7.86 (s, 1H), 7.52 (d, *J* = 6.7 Hz, 2H), 7.41 (d, *J* = 8.7 Hz, 1H), 7.37 – 7.28 (m, 3H), 7.08 (d, *J* = 2.7 Hz, 1H), 6.66 (dd, *J* = 8.7, 2.7 Hz, 1H), 3.02 ppm (s, 6H).

$^{13}C\{^1H\}$ NMR (126 MHz, $CDCl_3$) δ = 149.9, 138.2, 136.9, 133.2, 131.4 (2), 128.5 (2), 127.9, 124.0, 112.2, 111.5, 92.9, 90.6, 88.5, 40.4 ppm.

HRMS (ESI): *m/z*: $[M+H]^+$ calcd for $C_{18}H_{16}NBr_2^+$: 403.9649; found 403.9659.

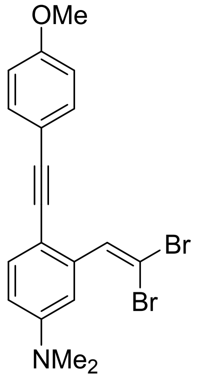

3-(2,2-Dibromovinyl)-4-((4-methoxyphenyl)ethynyl)-N,N-dimethyl aniline (**S40**) was prepared according to **GP2** from 100 mg (0.36 mmol) of **S24**. The column chromatography was performed on SiO_2 with a gradient from hexanes (1% TEA) to hexanes (1% TEA)/ethyl acetate = 7/1. After purification, 42 mg product (27%) was obtained as a greenish yellow solid.

^{1}H NMR (500 MHz, $CDCl_3$) δ = 7.85 (s, 1H), 7.45 (d, *J* = 8.8 Hz, 2H), 7.39 (d, *J* = 8.7 Hz, 1H), 7.08 (d, *J* = 2.7 Hz, 1H), 6.88 (d, *J* = 8.8 Hz, 2H), 6.65 (dd, *J* = 8.7, 2.7 Hz, 1H), 3.83 (s, 3H), 3.01 ppm (s, 6H).

$^{13}C\{^1H\}$ NMR (126 MHz, $CDCl_3$) δ = 159.5, 137.9, 137.0, 133.0, 132.8 (2), 116.2, 114.2 (2), 112.3, 111.6, 92.8, 90.4, 87.0, 55.5, 40.4 ppm.

HRMS (ESI): *m/z*: $[M+H]^+$ calcd for $C_{19}H_{18}NOBr_2^+$: 433.9755; found 433.9766.

S1.1.3 Monoareno-pentalenes (**1-8**)

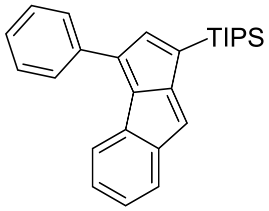

Triisopropyl(3-phenylcyclopenta[a]inden-1-yl)silane (**1**) was prepared according to **GP3** from 100 mg (0.28 mmol) of **S25**. The column chromatography was performed on SiO_2 with a gradient from hexanes to hexanes/ethyl acetate = 20/1. After purification, 60 mg product (47%) was obtained as a green solid.

Note: For the solvent effect on the NMR shifts, we measured this compound in CD_2Cl_2, C_6D_6, and CD_3CN. In CD_2Cl_2 it was very soluble, but less in the other two solvents; because of this, CD_2Cl_2 was chosen for further experiments. The proton attached to the five-membered ring adjacent to the annelated benzene ring was more sensitive for this solvent change.

^1H NMR (500 MHz, CD_2Cl_2) δ = 7.59 – 7.55 (m, 2H), 7.42 (t, *J* = 7.4 Hz, 2H), 7.35 (t, *J* = 7.4 Hz, 1H), 6.99 (dd, *J* = 6.3, 1.1 Hz, 1H), 6.71 – 6.64 (m, 3H), 6.36 (s, 1H), 6.12 (s, 1H), 1.23 – 1.10 ppm (m, 21H).

^1H NMR (500 MHz, C_6D_6) δ = 7.51 – 7.46 (m, 2H), 7.09 (t, *J* = 7.2 Hz, 2H), 7.07 – 7.02 (m, 2H), 6.55 – 6.50 (m, 2H), 6.50 – 6.46 (m, 1H), 6.41 (s, 1H), 6.04 (s, 1H), 1.15 ppm (d, *J* = 3.6 Hz, 21H).

^1H NMR (500 MHz, CD_3CN) δ = 7.62 – 7.58 (m, 2H), 7.45 (t, *J* = 7.4 Hz, 2H), 7.39 (t, *J* = 7.4 Hz, 1H), 7.01 (d, *J* = 6.7 Hz, 1H), 6.75 – 6.66 (m, 3H), 6.43 (s, 1H), 6.24 (s, 1H), 1.26 – 1.17 (m, 3H), 1.12 ppm (d, *J* = 7.1 Hz, 18H).

$^{13}C\{^1H\}$ NMR (126 MHz, CD_2Cl_2) δ = 160.5, 152.5, 150.8, 142.9, 142.8, 136.6, 135.0, 134.1, 133.1, 129.2, 128.9, 128.8 (2), 127.8 (2), 127.7, 124.3, 121.0, 18.9, 11.8 ppm.

$^{13}C\{^1H\}$ NMR (126 MHz, C_6D_6) δ = 158.3, 150.4, 148.6, 141.3, 140.8, 134.7, 132.9, 131.9, 130.6, 126.7, 126.6, 126.5 (2), 126.1 (2), 125.9, 125.7, 125.5, 125.4, 16.7, 9.6 ppm.

$^{13}C\{^1H\}$ NMR (126 MHz, CD_3CN) δ = 160.9, 153.0, 151.3, 143.5, 143.2, 136.9, 135.2, 133.8, 130.1, 129.72 (2), 129.71, 128.6, 128.4 (2), 125.3, 121.7, 19.1, 12.2.

HRMS (ESI): *m/z*: $[M+H]^+$ calcd for $C_{27}H_{33}Si^+$: 385.2351; found 385.2368.

IR (ATR) ν_{max} 2942, 2863, 1697, 1600, 1492, 1462, 1442, 1382, 1366, 1316, 1264, 1180, 1137, 1092, 1072, 1014, 996, 922, 882, 849, 805, 759, 743, 695, 680, 645, 603, 587, 568 cm^{-1}.

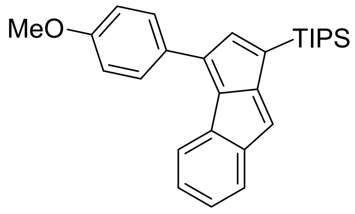

Triisopropyl(3-(4-methoxyphenyl)cyclopenta[a]inden-1-yl)silane (**1’**) was prepared according to **GP3** from 100 mg (0.27 mmol) of **S26**. The column chromatography was performed on SiO_2 with a gradient from hexanes to hexanes/ethyl acetate = 12/1. After purification, 32 mg product (29%) was obtained as a green solid.

^{1}H NMR (500 MHz, CD_2Cl_2) δ = 7.54 (d, J = 8.8 Hz, 2H), 7.00 (d, J = 6.8 Hz, 1H), 6.95 (d, J = 8.8 Hz, 2H), 6.73 – 6.63 (m, 3H), 6.35 (s, 1H), 6.10 (s, 1H), 3.85 (s, 3H), 1.22 – 1.10 ppm (m, 21H).

^{13}C{^{1}H} NMR (126 MHz, CD_2Cl_2) δ = 160.7, 160.5, 152.3, 150.6, 142.8, 141.0, 136.7, 133.3, 133.2, 129.5 (2), 128.7, 127.5, 127.3, 124.2, 120.8, 114.4 (2), 55.8, 18.9, 11.9 ppm.

HRMS (ESI): m/z: $[M+H]^+$ calcd for $C_{28}H_{35}OSi^+$: 415.2457; found 415.2474.

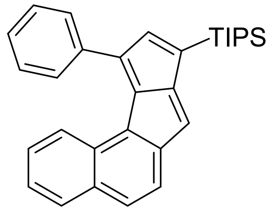

Triisopropyl(10-phenylpentaleno[1,2-a]naphthalen-8-yl)silane (**2**) was prepared according to **GP3** from 100 mg (0.24 mmol) of **S27**. The column chromatography was performed on SiO_2 with a gradient from hexanes to hexanes/ethyl acetate = 20/1. After purification, 60 mg product (57%) was obtained as a green solid.

^{1}H NMR (500 MHz, CD_2Cl_2) δ = 7.50 – 7.45 (m, 2H), 7.42 – 7.35 (m, 4H), 7.15 (d, J = 8.1 Hz, 1H), 7.00 (t, J = 8.1 Hz, 1H), 6.91 (d, J = 8.6 Hz, 1H), 6.80 (d, J = 8.0 Hz, 1H), 6.74 (t, J = 7.7 Hz, 1H), 5.94 (s, 1H), 5.90 (s, 1H), 1.15 ppm (s, 21H).

^{13}C{^{1}H} NMR (126 MHz, CD_2Cl_2) δ = 162.2, 154.7, 149.2, 146.1, 145.8, 136.2, 135.3, 134.6, 134.5, 134.4, 129.4, 129.2, 128.7 (2), 128.6 (2), 128.5, 128.2, 126.4, 126.1, 125.6, 122.6, 18.9, 11.8.

HRMS (ESI): m/z: $[M+H]^+$ calcd for $C_{31}H_{35}Si^+$: 435.2508; found 435.2498.

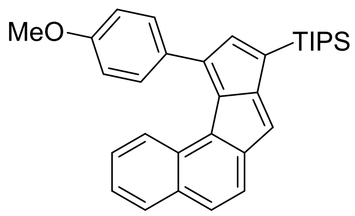

Triisopropyl(10-(4-methoxyphenyl)pentaleno[1,2-a] naphthalen-8-yl)silane (**2’**) was prepared according to **GP3** from 100 mg (0.23 mmol) of **S28**. The column chromatography was performed on SiO_2 with a gradient from hexanes to

hexanes/ethyl acetate = 12/1. After purification, 100 mg product (95%) was obtained as a green solid.

Note: On the NMR spectra, some TIPS-containing impurities can be observed, but we could not remove them with further purification. This impurity could contribute to the high yield. However, the material looked homogenous.

^{1}H NMR (500 MHz, CD_2Cl_2) δ = 7.44 (d, J = 8.7 Hz, 2H), 7.40 (d, J = 8.3 Hz, 1H), 7.15 (d, J = 8.1 Hz, 1H), 7.09 (d, J = 8.7 Hz, 1H), 7.03 (t, J = 7.5 Hz, 1H), 6.91 (d, J = 8.7 Hz, 2H), 6.84 – 6.79 (m, 2H), 5.95 (s, 1H), 5.90 (s, 1H), 3.85 (s, 3H), 1.15 ppm (apparent s, 21H).

^{13}C{^{1}H} NMR (126 MHz, CD_2Cl_2) δ = 162.3, 160.9, 154.3, 148.9, 145.7, 144.6, 135.2, 134.7, 134.6, 133.7, 130.4 (2), 129.2, 128.5, 128.2, 127.8, 126.3, 126.0, 125.6, 122.6, 114.1 (2), 55.8, 19.0, 11.9 ppm.

HRMS (ESI): *m/z*: $[M+H]^+$ calcd for $C_{32}H_{37}OSi^+$: 465.2613; found 465.2630.

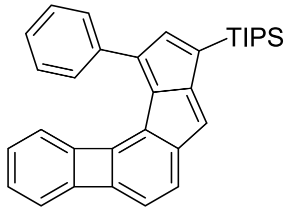

Triisopropyl(1-phenylpentaleno[1,2-a]biphenylen-3-yl)silane (**3**) was prepared according to **GP3** from 30 mg (0.07 mmol) of **S29**. The column chromatography was performed on SiO_2 with a gradient from hexanes to hexanes/ethyl acetate = 12/1. After purification, 19 mg product (60%) was obtained as a purple solid.

^{1}H NMR (500 MHz, CD_2Cl_2) δ = 7.52 (dd, J = 8.3, 1.4 Hz, 2H), 7.48 (t, J = 7.6 Hz, 2H), 7.43 – 7.37 (m, 1H), 6.95 – 6.91 (m, 1H), 6.90 – 6.85 (m, 2H), 6.73 (s, 1H), 6.47 (s, 1H), 6.43 (d, J = 6.7 Hz, 1H), 6.32 – 6.28 (m, 1H), 6.19 (d, J = 6.6 Hz, 1H), 1.29 – 1.22 (m, 3H), 1.12 ppm (d, J = 7.3 Hz, 18H).

^{13}C{^{1}H} NMR (126 MHz, CD_2Cl_2) δ = 160.2, 152.2, 151.2, 150.9, 150.8, 150.8, 140.8, 138.7, 135.6, 134.8, 131.1, 129.4 (2), 129.14, 129.11 (2), 128.9, 128.6, 125.2, 124.3, 121.9, 118.0, 113.7, 19.0, 12.0 ppm.

HRMS (ESI): *m/z*: $[M+H]^+$ calcd for $C_{33}H_{35}Si^+$: 459.2508; found 459.2523.

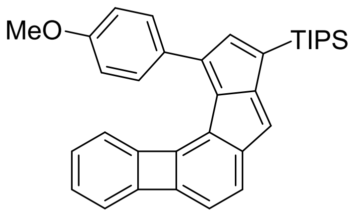

Triisopropyl(1-(4-methoxyphenyl)pentaleno[1,2-a] biphenylene-3-yl)silane (**3'**) was prepared according to **GP3** from 30 mg (0.06 mmol) of **S30**. The column chromatography was performed on SiO_2 with a gradient from hexanes to

hexanes/ethyl acetate = 9/1. After purification, 17 mg product (54%) was obtained as a purple solid.

^{1}H NMR (500 MHz, CD_2Cl_2) δ = 7.51 (d, J = 8.7 Hz, 2H), 7.02 (d, J = 8.7 Hz, 2H), 6.94 – 6.91 (m, 1H), 6.89 (d, J = 6.9 Hz, 1H), 6.85 (d, J = 6.7 Hz, 1H), 6.72 (s, 1H), 6.46 – 6.42 (m, 3H), 6.19 (d, J = 6.7 Hz, 1H), 3.87 (s, 3H), 1.12 ppm (d, J = 7.3 Hz, 21H).

^{13}C{^{1}H} NMR (126 MHz, CD_2Cl_2) δ = 160.4, 160.3, 152.0, 151.2, 151.0, 150.9, 150.8, 140.7, 140.6, 136.9, 133.9, 131.3, 131.0 (2), 129.1, 128.8, 128.2, 125.0, 124.6, 121.7, 117.9, 114.7 (2), 113.5, 55.9, 19.0, 12.0 ppm.

HRMS (ESI): *m/z*: $[M+H]^+$ calcd for $C_{34}H_{37}OSi^+$: 489.2613; found 489.2628.

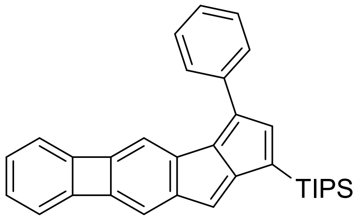

Triisopropyl(3-phenylpentaleno[1,2-b]biphenylen-1-yl)silane (**4**) was prepared according to **GP3** from 50 mg (0.12 mmol) of **S31**. The column chromatography was performed on SiO_2 with a gradient from hexanes to hexanes/ethyl acetate = 12/1. After purification, 8 mg product (15%) was obtained as an orangish brown film-like solid.

^{1}H NMR (500 MHz, CD_2Cl_2) δ = 7.53 – 7.47 (m, 2H), 7.42 (t, J = 7.4 Hz, 2H), 7.40 – 7.33 (m, 1H), 6.72 – 6.64 (m, 2H), 6.52 – 6.46 (m, 2H), 6.46 (t, J = 0.8 Hz, 1H), 6.17 (d, J = 1.0 Hz, 1H), 6.15 (s, 1H), 5.82 (s, 1H), 1.12 ppm (d, J = 4.2 Hz, 21H).

^{13}C{^{1}H} NMR (126 MHz, CD_2Cl_2) δ = 159.2, 151.9, 151.8, 150.6, 150.5, 150.0, 145.0, 143.3, 137.1, 134.8, 134.8, 134.4, 130.3, 129.5, 129.0 (2), 128.1, 128.0, 127.9 (2), 116.3, 116.1, 115.4, 112.5, 18.9, 11.8 ppm.

HRMS (ESI): *m/z*: $[M+H]^+$ calcd for $C_{33}H_{35}Si^+$: 459.2508; found 459.2490.

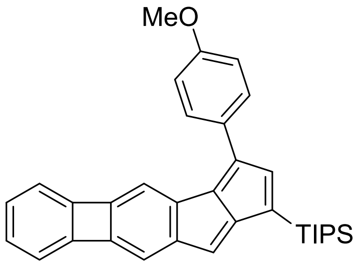

Triisopropyl(3-(4-methoxyphenyl)pentaleno[1,2-b] biphenylen-1-yl)silane (**4’**) was prepared according to **GP3** from 50 mg (0.11 mmol) of **S32**. The column chromatography was performed on SiO_2 with a gradient from hexanes to hexanes/ethyl acetate = 9/1. After purification, 3 mg product (6%) was obtained as an orangish brown film-like solid.

^{1}H NMR (500 MHz, CD_2Cl_2) δ = 7.47 (d, J = 8.8 Hz, 2H), 6.95 (d, J = 8.8 Hz, 2H), 6.71 – 6.65 (m, 2H), 6.49 (t, J = 3.8 Hz, 3H), 6.18 (s, 1H), 6.14 (s, 1H), 5.81 (s, 1H), 3.85 (s, 3H), 1.18 – 1.09 ppm (m, 21H).

$^{13}C\{^{1}H\}$ NMR (126 MHz, CD_2Cl_2) δ = 161.0, 159.1, 151.7, 151.5, 150.2, 150.1, 150.1, 149.9, 145.0, 141.5, 137.2, 135.0, 133.4, 129.7 (2), 128.1, 127.9, 127.2, 116.2, 116.0, 115.3, 114.4 (2), 112.3, 55.8, 18.9, 11.8 ppm.

HRMS (ESI): *m/z*: $[M+H]^+$ calcd for $C_{34}H_{37}OSi^+$: 489.2613; found 489.2596.

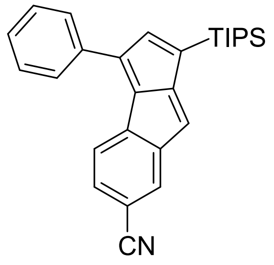

3-Phenyl-1-(triisopropylsilyl)cyclopenta[a]indene-6-carbonitrile (**5**) was prepared according to **GP3** from 89 mg (0.23 mmol) of **S33**. The column chromatography was performed on SiO_2 with a gradient from hexanes to hexanes/ethyl acetate = 9/1. After purification, 24 mg product (25%) was obtained as a green solid.

^{1}H NMR (500 MHz, CD_2Cl_2) δ = 7.59 – 7.55 (m, 2H), 7.46 (t, J = 7.3 Hz, 2H), 7.41 (t, J = 7.2 Hz, 1H), 7.05 (s, 2H), 6.90 (s, 1H), 6.42 (s, 1H), 6.18 (s, 1H), 1.24 – 1.10 ppm (m, 21H).

$^{13}C\{^{1}H\}$ NMR (126 MHz, CD_2Cl_2) δ = 161.8, 153.1, 151.3, 146.9, 141.1, 140.6, 135.7, 134.3, 134.0, 132.4, 130.1, 129.2 (2), 128.0 (2), 126.0, 120.6, 119.5, 110.3, 18.9, 11.8 ppm.

HRMS (ESI): *m/z*: $[M+H]^+$ calcd for $C_{28}H_{32}NSi^+$: 410.2304; found 410.2321.

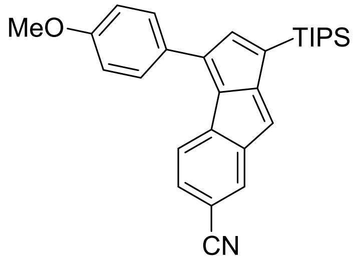

3-(4-Methoxyphenyl)-1-(triisopropylsilyl)cyclopenta[a] indene-6-carbonitrile (**5'**) was prepared according to **GP3** from 100 mg (0.24 mmol) of **S34**. The column chromatography was performed on SiO_2 with a gradient from hexanes to hexanes/ethyl acetate = 7/1. After purification, 23 mg product (22%) was obtained as a green solid.

^{1}H NMR (500 MHz, CD_2Cl_2) δ = 7.55 (d, J = 8.8 Hz, 2H), 7.08 (d, J = 7.7 Hz, 1H), 7.06 (dd, J = 7.7, 1.4 Hz, 1H), 6.98 (d, J = 8.8 Hz, 2H), 6.92 (d, J = 1.4 Hz, 1H), 6.42 (s, 1H), 6.17 (s, 1H), 3.86 (s, 3H), 1.23 – 1.11 ppm (m, 21H).

$^{13}C\{^{1}H\}$ NMR (126 MHz, CD_2Cl_2) δ = 161.8, 161.5, 152.8, 151.0, 146.9, 140.7, 139.1, 135.9, 133.7, 131.3, 130.0 (2), 126.7, 126.0, 120.3, 119.7, 114.6 (2), 109.7, 55.9, 18.9, 11.9 ppm.

HRMS (ESI): *m/z*: $[M+H]^+$ calcd for $C_{29}H_{34}NOSi^+$: 440.2409; found 440.2421.

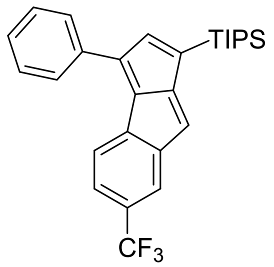

Triisopropyl(3-phenyl-6-(trifluoromethyl)cyclopenta[a]inden-1-yl)silane (**6**) was prepared according to **GP3** from 100 mg (0.23 mmol) of **S35**. The column chromatography was performed on SiO_2 with a

gradient from hexanes to hexanes/ethyl acetate = 12/1. After purification, 45 mg product (43%) was obtained as a green solid.

^{1}H NMR (500 MHz, CD_2Cl_2) δ = 7.59 (d, J = 7.0 Hz, 2H), 7.46 (t, J = 7.4 Hz, 2H), 7.40 (t, J = 7.3 Hz, 1H), 7.08 (d, J = 7.7 Hz, 1H), 7.00 (d, J = 7.8 Hz, 1H), 6.90 (s, 1H), 6.42 (s, 1H), 6.20 (s, 1H), 1.24 – 1.17 (m, 3H), 1.13 ppm (d, J = 6.9 Hz, 18H).

^{13}C{^{1}H} NMR (126 MHz, CD_2Cl_2) δ = 161.7, 152.9, 151.4, 145.6, 141.2, 139.96 (q, J = 1.7 Hz) 134.9, 134.5, 132.8, 129.9, 129.12 (2), 129.12 (q, J = 31.9 Hz), 128.0, 126.21 (q, J = 4.3 Hz), 124.72 (q, J = 271.6 Hz), 120.32 (q, J = 3.5 Hz). 120.2, 18.9, 11.9 ppm.

HRMS (APCI): *m/z*: $[M]^{\bullet+}$ calcd for $C_{28}H_{31}F_3Si^{\bullet+}$: 452.2147; found 452.2125.

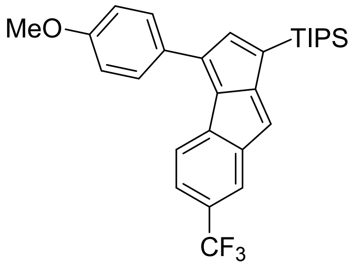

Triisopropyl(3-(4-methoxyphenyl)-6-(trifluoromethyl) cyclopenta[a]inden-1-yl)silane (**6'**) was prepared according to **GP3** from 100 mg (0.22 mmol) of **S36**. The column chromatography was performed on SiO_2 with a gradient from hexanes to hexanes/ethyl acetate = 9/1. After purification, 33 mg product (31%) was obtained as a green solid.

^{1}H NMR (500 MHz, CD_2Cl_2) δ = 7.57 (d, J = 8.8 Hz, 2H), 7.10 (d, J = 7.8 Hz, 1H), 7.01 (d, J = 8.6 Hz, 1H), 6.98 (d, J = 8.8 Hz, 2H), 6.92 (s, 1H), 6.41 (s, 1H), 6.18 (s, 1H), 3.86 (s, 3H), 1.23 – 1.16 (m, 3H), 1.13 ppm (d, J = 7.0 Hz, 18H).

^{13}C{^{1}H} NMR (126 MHz, CD_2Cl_2) δ = 161.7, 161.3, 152.6, 151.1, 145.6, 140.00 (q, J = 1.5 Hz), 139.3, 135.1, 131.8, 129.8 (2), 128.61 (q, J = 32.2 Hz), 128.1, 126.9, 125.96 (q, J = 4.2 Hz), 124.81 (q, J = 271.3 Hz), 120.22 (q, J = 3.6 Hz).119.9, 114.6 (2), 55.9, 18.9, 11.9 ppm.

HRMS (ESI): *m/z*: $[M+H]^+$ calcd for $C_{29}H_{34}OF_3Si^+$: 483.2331; found 483.2349.

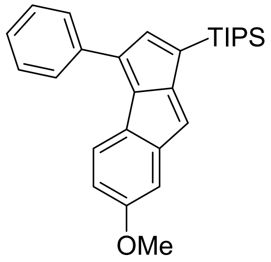

Triisopropyl(6-methoxy-3-phenylcyclopenta[a]inden-1-yl)silane (**7**) was prepared according to **GP3** from 100 mg (0.26 mmol) of **S37**. The column chromatography was performed on SiO_2 with a gradient from hexanes to hexanes/ethyl acetate = 9/1. After purification, 38 mg product (36%) was obtained as a green film-like solid.

^{1}H NMR (500 MHz, CD_2Cl_2) δ = 7.55 (dd, J = 8.2, 1.3 Hz, 2H), 7.41 (t, J = 7.7 Hz, 2H), 7.33 (t, J = 7.4 Hz, 1H), 6.92 (d, J = 8.1 Hz, 1H), 6.38 (s, 1H), 6.33 (d, J = 2.4 Hz, 1H), 6.15 (dd, J

= 8.1, 2.4 Hz, 1H), 6.04 (s, 1H), 3.70 (s, 3H), 1.22 – 1.14 (m, 3H), 1.12 ppm (d, J = 6.5 Hz, 18H).

$^{13}C\{^1H\}$ NMR (126 MHz, CD_2Cl_2) δ = 162.1, 160.3, 153.3, 152.7, 142.7, 140.2, 135.1, 132.8, 131.9, 128.95, 128.91 (2), 128.8, 127.6 (2), 121.7, 113.5, 110.2, 55.7, 18.9, 11.9 ppm.

HRMS (ESI): *m/z*: $[M+H]^+$ calcd for $C_{28}H_{35}OSi^+$: 415.2457; found 415.2472.

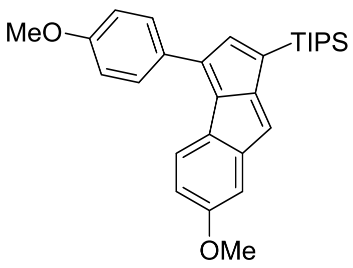

Triisopropyl(6-methoxy-3-(4-methoxyphenyl)cyclopenta[a]inden-1-yl)silane (**7’**) was prepared according to **GP3** from 100 mg (0.24 mmol) of **S38**. The column chromatography was performed on SiO_2 with a gradient from hexanes to hexanes/ethyl acetate = 7/1. After purification, 42 mg product (40%) was obtained as a green film-like solid.

Note: This compound could not be separated from residual starting material. The carbon peaks were separated via HSQC and HMBC assignations.

^1H NMR (500 MHz, CD_2Cl_2) δ = 7.52 (d, J = 8.8 Hz, 2H), 6.95 (d, J = 8.9 Hz, 2H), 6.92 (d, J = 8.3 Hz, 1H), 6.37 (s, 1H), 6.35 (d, J = 2.4 Hz, 1H), 6.17 (dd, J = 8.1, 2.5 Hz, 1H), 6.03 (s, 1H), 3.84 (s, 3H), 3.70 (s, 3H), 1.22 – 1.13 (m, 3H), 1.12 ppm (d, J = 6.6 Hz, 18H).

$^{13}C\{^1H\}$ NMR (126 MHz, CD_2Cl_2) δ = 162.1, 160.4, 160.0, 153.1, 152.5, 140.9, 140.1, 132.1, 131.9, 129.3 (2), 129.1, 127.6, 121.4, 114.4 (2), 113.2, 110.2, 55.8, 55.7, 18.9, 11.9 ppm.

HRMS (ESI): *m/z*: $[M+H]^+$ calcd for $C_{29}H_{37}O_2Si^+$: 445.2562; found 445.2580.

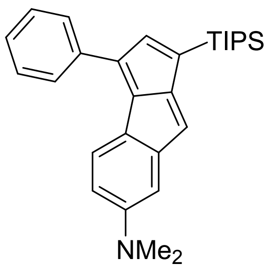

N,N-Dimethyl-3-phenyl-1-(triisopropylsilyl)cyclopenta[a]inden-6-amine (**8**) was prepared according to **GP3** from 103 mg (0.25 mmol) of **S39**. The column chromatography was performed on SiO_2 with a gradient from hexanes to hexanes/ethyl acetate = 9/1. After purification, 37 mg product (34%) was obtained as a green solid.

Note: During the overnight NMR measurements, the material decomposed. Hence, we could not obtain NOESY spectra from it.

^1H NMR (500 MHz, CD_2Cl_2) δ = 7.58 – 7.52 (m, 2H), 7.40 (t, J = 7.5 Hz, 2H), 7.31 (t, J = 7.4 Hz, 1H), 6.90 (d, J = 8.2 Hz, 1H), 6.39 (s, 1H), 6.29 (d, J = 2.5 Hz, 1H), 6.01 (s, 1H), 5.92 (dd, J = 8.2, 2.5 Hz, 1H), 2.89 (s, 6H), 1.22 – 1.17 (m, 3H), 1.14 ppm (d, J = 6.2 Hz, 18H).

$^{13}C\{^{1}H\}$ NMR (126 MHz, CD_2Cl_2) δ = 162.1, 153.5, 152.5, 150.9, 144.0, 137.9, 135.5, 133.2, 130.5, 128.8 (2), 128.3, 127.5 (2), 124.0, 122.4, 111.6, 109.0, 40.8, 19.0, 11.9 ppm.

S1.1.4 Gram scale synthesis of **1**

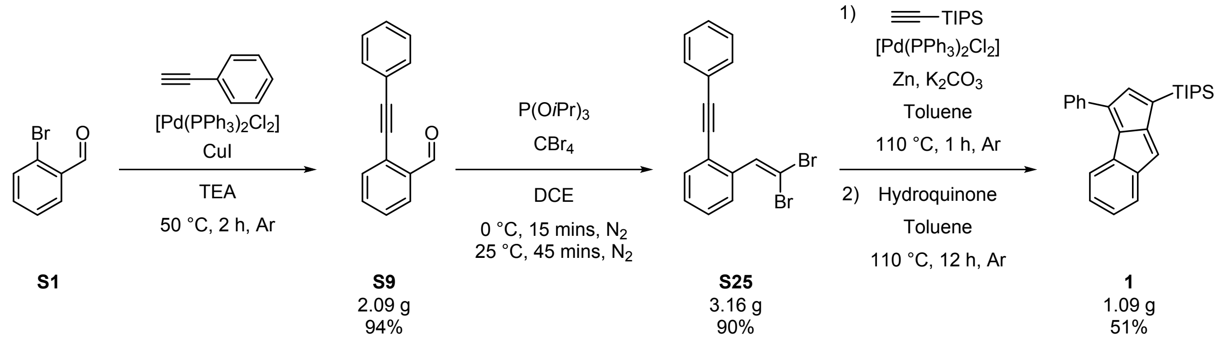

Figure S4. Gram scale synthesis of **1**. For each step 2.00 g starting material was used.

2-Bromobenzaldehyde (2.00 g, 10.8 mmol) and ethynylbenzene (1.16 g, 11.4 mmol) were dissolved in triethylamine (100 mL, 0.11 M). The solution was added to a 250 mL two-necked round-bottom flask that contained $Pd(PPh_3)_2Cl_2$ (76 mg, 108 μmol) and CuI (21 mg, 108 μmol) (*Note*: for larger scale reactions the catalyst loading was lower than for the general Sonogashira reactions.) under an inert atmosphere (Ar). The mixture was heated to 50 °C in an oil bath and stirred for 2 h. After the reaction was completed (followed by TLC analysis), the mixture was diluted with EtOAc and filtered through a Celite plug. The solvent was evaporated *in vacuo*, and the crude product was further purified with column chromatography (SiO_2, with a gradient from hexanes to hexanes/EtOAc = 19/1). After purification, 2.09 g 2-(phenylethynyl)benzaldehyde (**S9**, 94%) was obtained as an orange oil.

In a 250 mL round-bottom flask **S9** (2.00 g, 9.7 mmol) and CBr_4 (4.82 g, 14.5 mmol) were dissolved in DCE (100 mL, 0.09 M). After the solution was purged with N_2 for 10 min and cooled down to 0 °C with an ice-water bath, P(O*i*Pr)$_3$ (6.06 g, 29.1 mmol) was added to the solution. The reaction was stirred at 0 °C for 15 min, then at r.t. for 45 min. The solvent was evaporated *in vacuo*. Subsequently, the crude product was further purified with column chromatography (SiO_2, hexanes). After purification, 3.16 g 1-(2,2-dibromovinyl)-2-(phenylethynyl)benzene (**S25**, 90%) was obtained as a pale yellow solid.

A 250 mL two-necked round-bottom flask was charged with **S25** (2.00 g, 5.5 mmol), $Pd(PPh_3)_2Cl_2$ (390 mg, 550 μmol), Zn (540 mg, 8.3 mmol), and K_2CO_3 (1.53 g, 11 mmol) and purged with Ar. After 10 min of purging with Ar, first toluene (80 mL), then a solution of TIPS-acetylene (6.2 mL, 27.6 mmol) in toluene (20 mL, in total for **S25** 52 mM) was added to the

flask. The mixture was heated to 110 °C in an oil bath and stirred for 1 h. After the initial stirring, the mixture was cooled to rt, and hydroquinone (910 mg, 8.3 mmol) and a further portion of TIPS-acetylene (6.2 mL) were added to the reaction. Subsequently, the flask was purged with Ar, and the solution was heated up to 110 °C and stirred for 12 h. Upon completion, the mixture was filtered through a Celite plug. This was followed by the removal of the solvent under reduced pressure and the further purification of the product with column chromatography (SiO_2, hexanes). After purification, 1.09 g triisopropyl(3-phenylcyclopenta[*a*]inden-1-yl)silane (**1**, 51%) was obtained as a brownish-green solid.

S1.2 Test reactions to access different substitution patterns

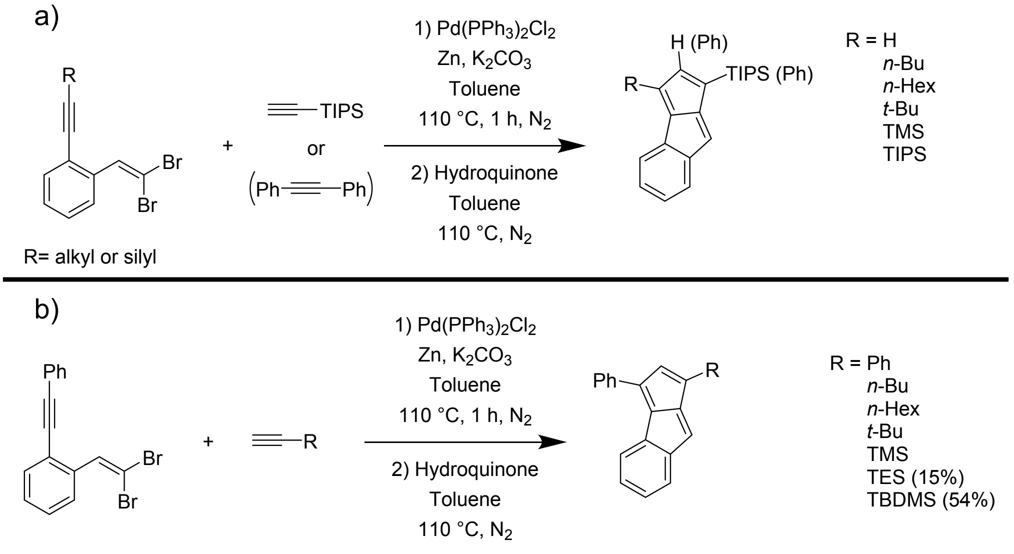

Figure S5. Scheme of test reactions to obtain different substitution patterns (if successful, the yield is in parenthesis).
a) Changes in the pendant 'R' group; b) Changes of the reagent acetylene.

Starting materials were prepared by Sonogashira reactions with alkyl or silyl acetylenes. The carbopalladation cascade reactions did not work in most of the cases (**GP3**). We tried the cascade reactions with diphenylacetylene, which were unsuccessful (Figure S5, a). When changing the TIPS acetylene reagent to less bulky acetylenes (Figure S5, b), the reaction did not occur, however, with similar bulky silyl groups (TES, TBDMS) the reaction worked well, suggesting that the bulkiness of the 'R' group stabilises the products, however these compounds (**S52**, **S53**) were found to decompose on the time scale of the 2D NMR measurements. These test reactions were monitored by TLC. When the starting materials were consumed, the mixtures were filtered through a Celite pad. After the evaporation of solvents, we obtained ^{1}H NMR spectra from the crude mixtures. In case we did not observe olefinic protons, we assumed that transformation did not occur.

S1.2.1 Preparation and characterization of starting materials

For the preparation of alkyl and silyl compounds (**S46-S51**), we used the same protocol as described above (**GP1** and **GP2**). First, Sonogashira reaction on 2-bromobenzaldehyde (**S1**), then Ramirez *gem*-dibromoolefination of the aldehyde. The R=H (**S51**) product was prepared via deprotecting the R=TMS (**S49**) molecule.

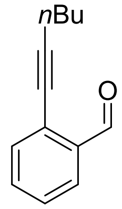

2-(Hex-1-yn-1-yl)benzaldehyde (**S41**)[5] was prepared according to **GP1** from 200 mg (1.1 mmol) of 2-bromobenzaldehyde (**S1**). The column chromatography was performed on SiO_2 with a gradient from hexanes to hexanes/ethyl acetate = 12/1. After purification, 191 mg product (95%) was obtained as a yellow oil.

^{1}H NMR (500 MHz, $CDCl_3$) δ = 10.54 (s, 1H), 7.88 (d, J = 7.5 Hz, 1H), 7.55 – 7.47 (m, 2H), 7.41 – 7.34 (m, 1H), 2.49 (t, J = 7.1 Hz, 2H), 1.68 – 1.59 (m, 2H), 1.56 – 1.45 (m, 2H), 0.97 ppm (t, J = 7.3 Hz, 3H).

^{13}C{^{1}H} NMR (126 MHz, $CDCl_3$) δ = 192.4, 136.2, 133.8, 133.5, 128.0, 127.1, 76.5, 30.8, 22.2, 19.4, 13.7 ppm.

HRMS (ESI): *m/z*: $[M+H]^+$ calcd for $C_{13}H_{15}O^+$: 187.1122; found 187.1120.

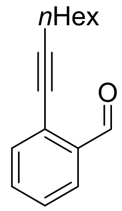

2-(Oct-1-yn-1-yl)benzaldehyde (**S42**)[6] was prepared according to **GP1** from 200 mg (1.1 mmol) of 2-bromobenzaldehyde (**S1**). The column chromatography was performed on SiO_2 with a gradient from hexanes to hexanes/ethyl acetate = 12/1. After purification, 197 mg product (85%) was obtained as a yellow oil.

^{1}H NMR (500 MHz, $CDCl_3$) δ = 10.54 (s, 1H), 7.89 (d, J = 8.3 Hz, 1H), 7.54 – 7.48 (m, 2H), 7.40 – 7.35 (m, 1H), 2.48 (t, J = 7.1 Hz, 2H), 1.64 (p, J = 7.2 Hz, 2H), 1.51 – 1.43 (m, 2H), 1.34 (tt, J = 7.6, 3.5 Hz, 4H), 0.94 – 0.88 ppm (apparent triplet, 3H).

^{13}C{^{1}H} NMR (126 MHz, $CDCl_3$) δ = 192.3, 136.2, 133.8, 133.4, 128.1, 128.0, 127.1, 98.4, 90.4, 76.5, 31.5, 28.8, 28.7, 22.7, 19.8, 14.2 ppm.

HRMS (ESI): *m/z*: $[M+H]^+$ calcd for $C_{15}H_{19}O^+$: 215.1435; found 215.1437.

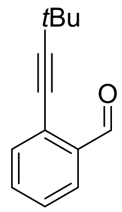

2-(3,3-Dimethylbut-1-yn-1-yl)benzaldehyde (**S43**)[7] was prepared according to **GP1** from 300 mg (1.6 mmol) of 2-bromobenzaldehyde (**S1**). The column chromatography was performed on SiO_2 with a gradient from hexanes to hexanes/ethyl acetate = 12/1. After purification, 267 mg product (88%) was obtained as a yellow oil.

^{1}H NMR (500 MHz, $CDCl_3$) δ = 10.54 (d, J = 0.9 Hz, 1H), 7.88 (dd, J = 7.8, 0.7 Hz, 1H), 7.54 – 7.46 (m, 2H), 7.41 – 7.34 (m, 1H), 1.35 ppm (s, 9H).

HRMS (ESI): *m/z*: $[M+H]^+$ calcd for $C_{13}H_{15}O^+$: 187.1122; found 187.1121.

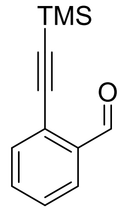

2-((Trimethylsilyl)ethynyl)benzaldehyde (**S44**)[8] was prepared according to **GP1** from 300 mg (1.6 mmol) of 2-bromobenzaldehyde (**S1**). The column chromatography was performed on SiO_2 with a gradient from hexanes to hexanes/ethyl acetate = 12/1. After purification, 204 mg product (62%) was obtained as a colourless oil.

^{1}H NMR (500 MHz, $CDCl_3$) δ = 10.56 (d, J = 0.9 Hz, 1H), 7.91 (dd, J = 7.8, 0.7 Hz, 1H), 7.57 (dd, J = 7.0, 0.8 Hz, 1H), 7.54 (td, J = 7.5, 1.4 Hz, 1H), 7.47 – 7.40 (m, 1H), 0.28 ppm (s, 9H).

HRMS (ESI): *m/z*: $[M+H]^+$ calcd for $C_{12}H_{15}OSi^+$: 203.0892; found 203.0888.

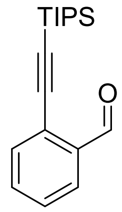

2-((Triisopropylsilyl)ethynyl)benzaldehyde (**S45**)[9] was prepared according to **GP1** from 300 mg (1.6 mmol) of 2-bromobenzaldehyde (**S1**). The column chromatography was performed on SiO_2 with a gradient from hexanes to hexanes/ethyl acetate = 12/1. After purification, 265 mg product (57%) was obtained as a yellow oil.

^{1}H NMR (500 MHz, $CDCl_3$) δ = 10.62 (d, J = 0.9 Hz, 1H), 7.92 (dd, J = 7.8, 1.4 Hz, 1H), 7.60 (dd, J = 7.7, 1.3 Hz, 1H), 7.54 (td, J = 7.5, 1.4 Hz, 1H), 7.46 – 7.41 (m, 1H), 1.15 ppm (d, J = 4.4 Hz, 21H).

HRMS (ESI): *m/z*: $[M+H]^+$ calcd for $C_{18}H_{27}OSi^+$: 287.1831; found 287.1827.

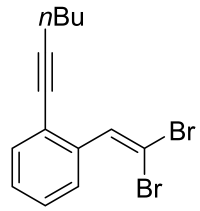

1-(2,2-dibromovinyl)-2-(hex-1-yn-1-yl)benzene (**S46**)[5] was prepared according to **GP2** from 100 mg (0.54 mmol) of **S41**. The column chromatography was performed on SiO_2 with a gradient from hexanes to hexanes/ethyl acetate = 12/1. After purification, 85 mg product (41%) was obtained as a yellow oil.

Note: Even after 24 h, the reaction mixture contained a small amount of starting material, which was inseparable from the product.

^{1}H NMR (500 MHz, $CDCl_3$) δ = 7.77 (s, 1H), 7.73 (dd, J = 7.4, 1.8 Hz, 1H), 7.41 (dd, J = 7.3, 1.8 Hz, 1H), 7.32 – 7.23 (m, 2H, overlaping with $CHCl_3$), 2.47 (t, J = 6.9 Hz, 2H), 1.67 – 1.58 (m, 2H), 1.59 – 1.48 (m, 2H), 0.98 ppm (t, J = 7.2 Hz, 3H).

$^{13}C\{^1H\}$ NMR (126 MHz, $CDCl_3$) δ = 137.3, 136.4, 132.2, 128.3, 128.0, 127.4, 123.9, 97.8, 90.8, 78.7, 30.9, 22.1, 19.4, 13.8 ppm.

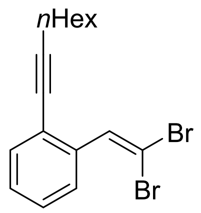

1-(2,2-Dibromovinyl)-2-(oct-1-yn-1-yl)benzene (**S47**)[6] was prepared according to **GP2** from 100 mg (0.47 mmol) of **S42**. The column chromatography was performed on SiO_2 with a gradient from hexanes to hexanes/ethyl acetate = 12/1. After purification, 102 mg product (59%) was obtained as a yellow oil.

Note: Even after 24 h, the reaction mixture contained a small amount of starting material, which was inseparable from the product.

^{1}H NMR (500 MHz, $CDCl_3$) δ = 7.77 (s, 1H), 7.73 (dd, J = 7.4, 1.8 Hz, 1H), 7.41 (dd, J = 7.3, 1.8 Hz, 1H), 7.31 – 7.23 (m, 2H, overlaping with $CHCl_3$), 2.46 (t, J = 7.0 Hz, 2H), 1.68 – 1.61 (m, 2H), 1.56 – 1.45 (m, 2H), 1.41 – 1.29 (m, 4H), 0.92 ppm (t, J = 6.9 Hz, 3H).

^{13}C{^{1}H} NMR (126 MHz, $CDCl_3$) δ = 137.3, 136.3, 132.2, 128.3, 128.0, 127.4, 123.9, 96.9, 90.8, 78.7, 31.6, 28.8, 28.7, 22.7, 19.8, 14.2 ppm.

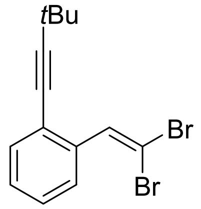

1-(2,2-Dibromovinyl)-2-(3,3-dimethylbut-1-yn-1-yl)benzene (**S48**)[10] was prepared according to **GP2** from 150 mg (0.81 mmol) of **S43**. The column chromatography was performed on SiO_2 with a gradient from hexanes to hexanes/ethyl acetate = 12/1. After purification, 206 mg product (75%) was obtained as a yellow oil.

^{1}H NMR (500 MHz, $CDCl_3$) δ = 7.75 (s, 1H), 7.73 – 7.68 (m, 1H), 7.41 – 7.37 (m, 1H), 7.30 – 7.22 (m, 2H, overlaping with $CHCl_3$), 1.35 ppm (s, 9H).

HRMS (ESI): *m/z*: $[M+H]^+$ calcd for $C_{14}H_{15}Br_2^+$: 340.9540; found 340.9541.

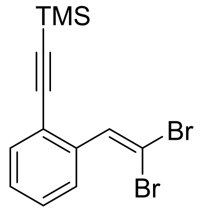

((2-(2,2-Dibromovinyl)phenyl)ethynyl)trimethylsilane (**S49**)[11] was prepared according to **GP2** from 150 mg (0.74 mmol) of **S44**. The column chromatography was performed on SiO_2 with a gradient from hexanes to hexanes/ethyl acetate = 12/1. After purification, 239 mg product (90%) was obtained as a yellow oil.

^{1}H NMR (500 MHz, $CDCl_3$) δ = 7.78 (s, 1H), 7.74 (dd, J = 8.0, 1.2 Hz, 1H), 7.47 (dd, J = 7.6, 1.5 Hz, 1H), 7.33 (td, J = 7.7, 1.5 Hz, 1H), 7.30 – 7.25 (m, 1H, overlaping with $CHCl_3$), 0.28 ppm (s, 9H).

HRMS (APCI): *m/z*: $[M]^{\bullet+}$ calcd for $C_{13}H_{14}SiBr_2^{\bullet+}$: 355.9231; found 355.9215.

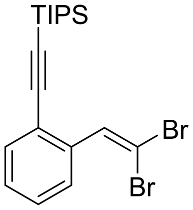

((2-(2,2-Dibromovinyl)phenyl)ethynyl)triisopropylsilane (**S50**)[12] was prepared according to **GP2** from 150 mg (0.52 mmol) of **S45**. The column chromatography was performed on SiO_2 with a gradient from hexanes to hexanes/ethyl acetate = 12/1. After purification, 219 mg product (93%) was obtained as a yellow oil.

^{1}H NMR (500 MHz, $CDCl_3$) δ = 7.84 (s, 1H), 7.76 (dd, J = 7.8, 1.3 Hz, 1H), 7.50 (dd, J = 7.6, 1.6 Hz, 1H), 7.33 (td, J = 7.7, 1.6 Hz, 1H), 7.31 – 7.26 (m, 1H, overlaping with $CHCl_3$), 1.16 ppm (s, 21H).

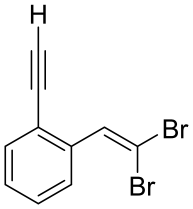

1-(2,2-Dibromovinyl)-2-ethynylbenzene (**S51**) was prepared from **S49** based on a similar reaction in the literature.[13] A vial was charged with 600 mg (1.68 mmol) **S49** and 255 mg (1.1 equiv., 1.84 mmol) K_2CO_3 and purged with N_2. To the solids, MeOH (6 mL) was added, and the reaction was monitored with TLC (SiO_2, hexanes/ethyl acetate = 20/1). After 30 min stirring at rt the starting material was consumed, and a single new spot was observed. To the reaction, water (20 mL) was added, and mixture was extracted with hexanes (3x20 mL), and the combined organic layer was dried over $MgSO_4$. After the removal of solvents *in vacuo* (*Note*: The solvents were removed at rt under reduced pressure because terminal acetylenes tend to decompose at 40 °C on the rotavap.), the crude product was purified with column chromatography (SiO_2, gradient from hexanes to hexanes/ethyl acetate = 20/1) and 349 mg product (73%) was obtained as a brown oil.

^{1}H NMR (500 MHz, $CDCl_3$) δ = 7.77 (d, J = 7.7 Hz, 2H), 7.52 (dd, J = 7.7, 1.5 Hz, 1H), 7.37 (td, J = 7.7, 1.4 Hz, 1H), 7.30 (t, J = 7.3 Hz, 1H), 3.37 ppm (s, 1H).

^{13}C{^{1}H} NMR (126 MHz, $CDCl_3$) δ = 138.0, 135.7, 133.1, 128.7, 128.4, 128.3, 121.8, 91.8, 82.9, 81.5 ppm.

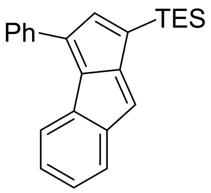

Triethyl(3-phenylcyclopenta[a]inden-1-yl)silane (**S52**) was prepared according to **GP3** from 100 mg (0.28 mmol) of **S25**. The column chromatography was performed on SiO_2 with hexanes. After purification, 14 mg product (15%) was obtained as a brownish-green solid.

^{1}H NMR (500 MHz, CD_2Cl_2) δ = 7.59 – 7.55 (m, 2H), 7.45 – 7.33 (m, 3H), 7.01 – 6.98 (m, 1H), 6.71 – 6.64 (m, 3H), 6.34 (s, 1H), 6.14 (s, 1H), 1.01 (t, J = 7.9 Hz, 9H), 0.68 ppm (q, J = 7.9 Hz, 6H).

$^{13}C\{^{1}H\}$ NMR (126 MHz, CD_2Cl_2) δ = 160.0, 151.3, 150.8, 143.0, 142.7, 136.7, 135.5, 135.0, 133.4, 129.2, 128.93 (2), 128.86, 127.8 (2), 127.7, 124.4, 121.1, 7.7 (3), 4.2 ppm (3).

Note: During the overnight NMR measurements the material decomposed. Hence, we could not obtain NOESY spectra from it.

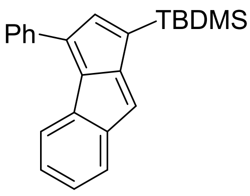

Tert-butyldimethyl(3-phenylcyclopenta[a]inden-1-yl)silane (S53) was prepared according to **GP3** from 100 mg (0.28 mmol) of **S25**. The column chromatography was performed on SiO_2 with hexanes. After purification, 51 mg product (54%) was obtained as a brownish-green solid.

^{1}H NMR (500 MHz, CD_2Cl_2) δ = 7.59 – 7.55 (m, 2H), 7.44 – 7.40 (m, 2H), 7.38 – 7.33 (m, 1H), 6.99 (d, J = 7.3 Hz, 1H), 6.71 – 6.63 (m, 3H), 6.34 (s, 1H), 6.14 (s, 1H), 0.96 (s, 9H), 0.14 ppm (s, 6H).

$^{13}C\{^{1}H\}$ NMR (126 MHz, CD_2Cl_2) δ = 160.1, 151.7, 150.8, 142.8, 142.8, 136.6, 136.0, 135.0, 134.1, 129.2, 128.94 (2), 128.89, 127.81 (2), 127.78, 124.4, 121.1, 26.8 (3), 16.7, -5.4 ppm (2).

Note: During the overnight NMR measurements the material decomposed. Hence, we could not obtain NOESY spectra from it.

S1.3 Assignation of olefinic protons from NOESY spectra

We were able to assign the olefinic protons in the pentalene moiety from the NOESY spectra of the compounds. The olefinic proton on the outer five-membered ring has cross-peaks with the protons on the pendant phenyl group and with the protons in the TIPS group (Figure S6, arrows 1 and 2, respectively). In contrast, the proton on the inner five-membered ring has cross-peaks with the TIPS group and the proton on the annulated benzene ring (Figure S6, arrows 3 and 4, respectively). Other NOESY spectra are at the NMR section of the supporting information (section S3).

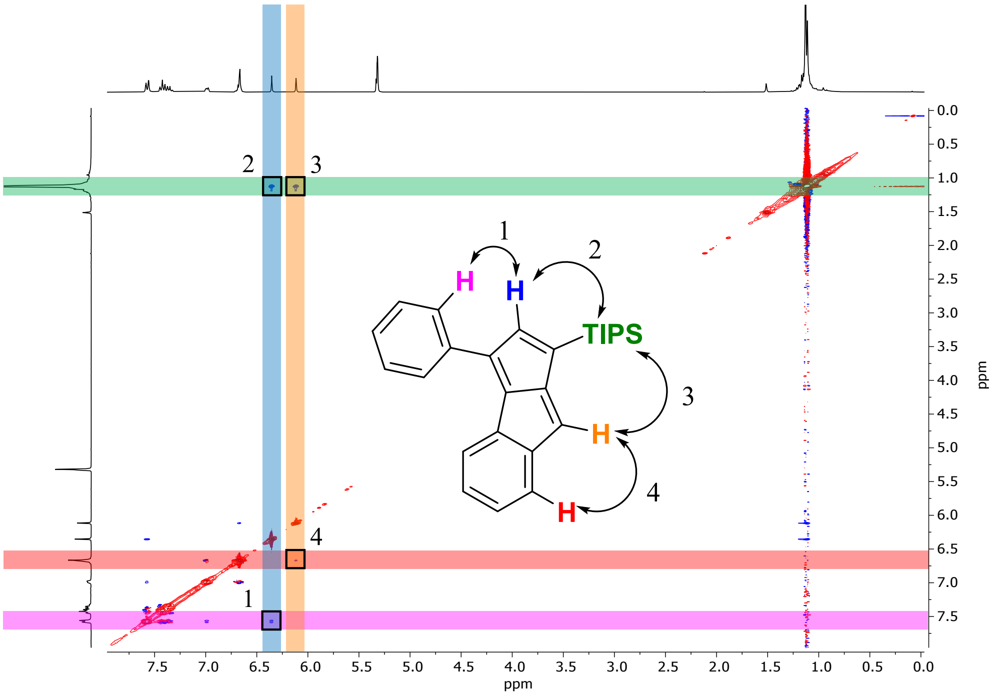

Figure S6. NOESY spectrum of **1**. The coloured columns and rows correspond to the protons of the same colour. The numbers correspond to the NOE interactions between the protons.

S1.4 Opto-electronic characterization and comparison

UV-Vis and cyclic voltammetry (CV) measurements were performed on **1** and **TPBP** (which was available in our group).

S1.4.1 UV-Vis measurements

PerkinElmer Lambda 465 spectrophotometer was used for the UV-Vis measurements with Hellma Analytics 10 mm quartz cuvettes in spectroscopic grade CH_2Cl_2. A stock solution of ~10^{-3} M was prepared from the materials, which was diluted further to ~10^{-5} M. Both solutions were measured, and the more concentrated solution gave more accurate ε values for the less intense absorptions.

In a 50 mL volumetric flask, 53.2 mg **1** was dissolved in CH_2Cl_2 (2.77x10^{-3} M, Figure S7, b, blue), 250 µL of this solution was diluted in a 25 mL volumetric flask (2.77x10^{-5} M, Figure S7, a, blue). For the **TPBP** solutions, in a 50 mL volumetric flask, 64.4 mg TPBP was dissolved in CH_2Cl_2 (3.39x10^{-3} M Figure S7, b, red), and 200 µL of this solution was diluted in a 25 mL volumetric flask (2.71x10^{-5} M, Figure S7, a, red). For comparing the HOMO-LUMO gaps obtained from different methods, see Table S1 in section **S2.1**.

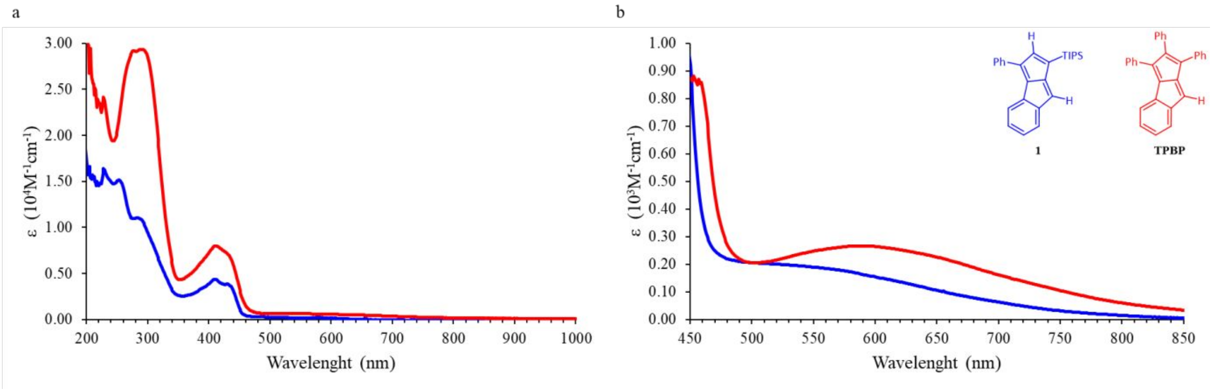

Figure S7. a) UV-Vis spectra of **1** ($2.77x10^{-5}$ M, blue) and **TPBP** ($2.71x10^{-5}$ M, red) in CH_2Cl_2.
b) UV-Vis spectra of **1** ($2.77x10^{-3}$ M, blue) and **TPBP** ($3.39x10^{-3}$ M, red) from concentrated solutions in CH_2Cl_2.

S1.4.2 Cyclic voltammetry measurements

General Pine Research Wavenow[xv] potentiostat was used for the CV measurements with a low-volume three-electrode cell, glassy carbon working electrode (disc OD 3.0 mm), platinum wire counter electrode, and Ag/AgCl reference electrode. For the CV measurements, dichloromethane, ferrocene, and tetrabutylammonium hexafluorophosphate (NBu_4PF_6) were used as the solvent, internal reference, and conducting salt, respectively. All materials were measured with and without ferrocene at the following scan rates: 20 mV/s, 100 mV/s, and 500 mV/s. After measurements, the potentials were corrected with the ferrocene halfway potential of appropriate scan rate. The on-set potentials for the first oxidation and reduction peaks were determined from the voltammogram, without ferrocene and with 20 mV/s scan rate.

Measurements In a 25 mL volumetric flask, 1010 mg NBu_4PF_6 was dissolved in CH_2Cl_2 (~0.1 M). This solution was used to dissolve 7,7 mg **1** in a 10 mL volumetric flask (~$2.00x10^{-3}$ M). 5 mL of this solution was transferred to the electrochemical cell and was purged with Ar stream and vigorously stirred for 5 min before measurement to remove O_2 from the solution. During the measurements, the Ar stream was slowed to a minimum, and the stirring was turned off. After initial measurements at different scan speeds (Figure S8, a), the cell was opened, and 2,0 mg ferrocene was added to the solution (~$2.15x10^{-3}$ M). It was re-purged with strong Ar stream, and then at different scan speeds, voltammograms were measured with ferrocene (Figure S8, b).

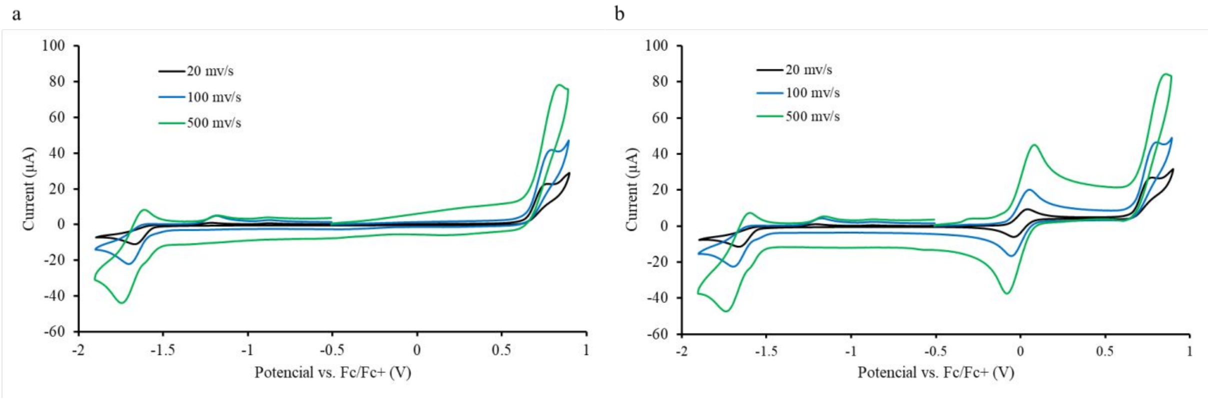

Figure S8. Voltammograms of **1** with different scan speeds a) without ferrocene; b) with ferrocene.

In a 25 mL volumetric flask, 1010 mg NBu_4PF_6 was dissolved in CH_2Cl_2 (~0.1 M). This solution was used to dissolve 8,9 mg **TPBP** in a 10 mL volumetric flask (~$2.34x10^{-3}$ M). 5 mL of this solution was transferred to the electrochemical cell and was purged with Ar stream and vigorously stirred for 5 minutes before measurement to remove O_2 from the solution. During the measurements, the Ar stream was slowed to a minimum, and the stirring was turned off. After initial measurements at different scan speeds (Figure S9, a), the cell was opened, and 1,2 mg ferrocene was added to the solution (~$1.29x10^{-3}$ M). It was re-purged with strong Ar stream, and then at different scan speeds voltammograms were measured with ferrocene (Figure S9, b).

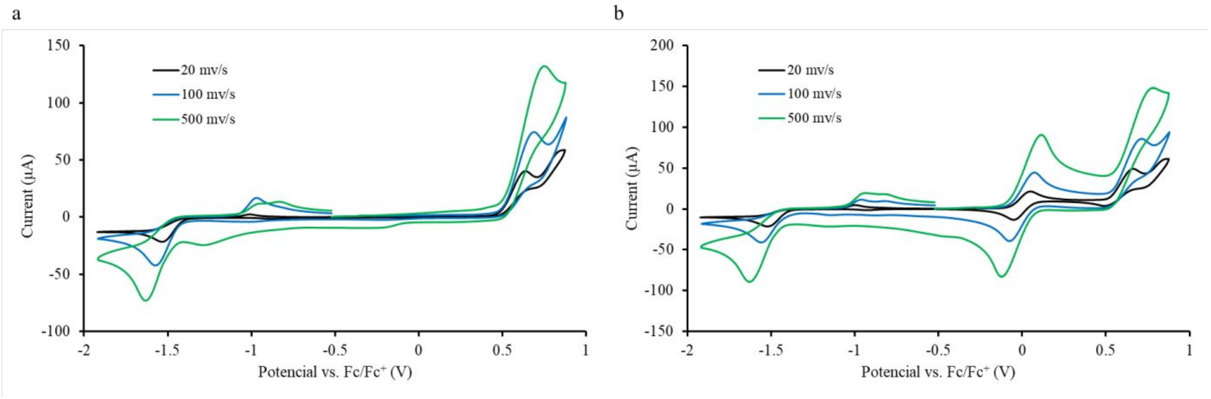

Figure S9. Voltammograms of **TPBP** with different scan speeds a) without ferrocene; b) with ferrocene.

For both monobenzopentalenes, we observed irreversible oxidation and reduction peaks at a low scan rate, however, for **1**, the reduction peak became quasi-reversible at higher scan rates. Onset potentials for **1** and **TPBP** were estimated, see Figure S10. The onset potential for the first reduction peak of **1** is -1.56 V. The onset potential for the first oxidation peak of **1** is 0.64 V. The onset potential for the first reduction peak of **TPBP** is -1.41 V. The onset potential for the first oxidation peak of **TPBP** is 0.49 V.

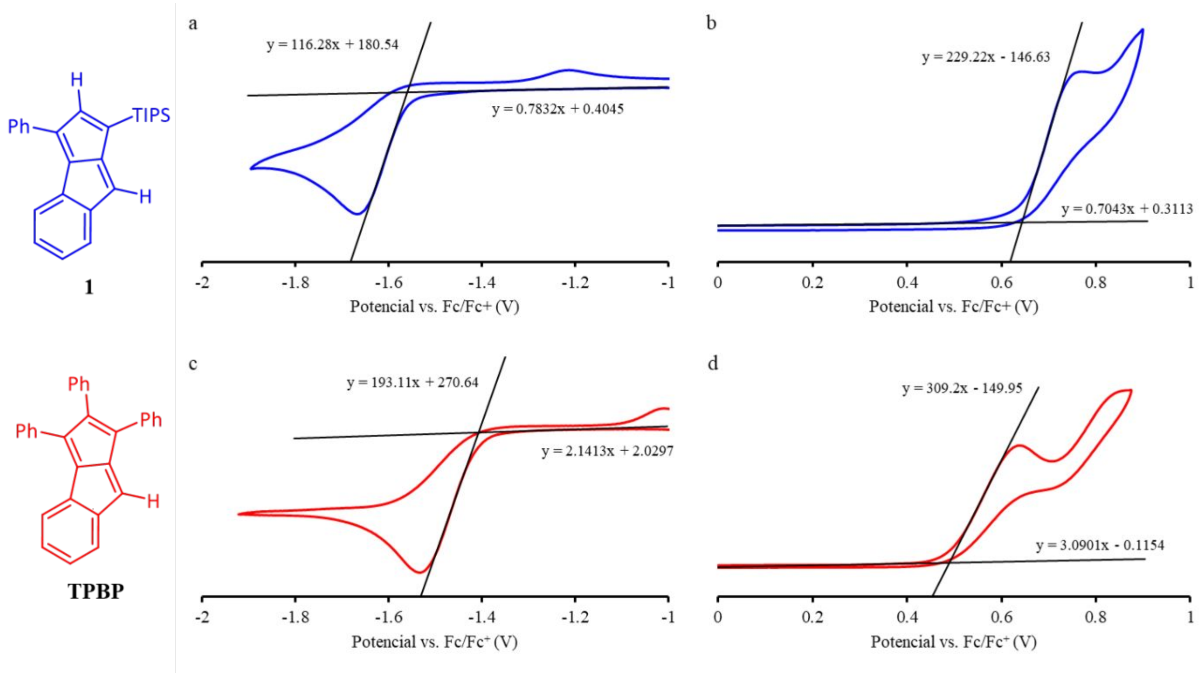

Figure S10. Estimation of onset potentials for a) first reduction peak of **1**; b) first oxidation peak of **1**; c) first reduction peak of **TPBP**; d) first oxidation peak of **TPBP**; the voltammograms were recorded with 20 mV/s scan rate.

S2 Theoretical characterization

General methods

All geometry optimisations were made with the Gaussian 09 rev E.01[14] package using the B3LYP[15] hybrid functional and the 6-311+G(d,p)[16] basis set. Analytical hessians were computed to confirm that the structures are minima. For the prepared compounds TD-DFT calculations were performed to obtain the HOMO-LUMO transition energies (with the following commands: td=(nstates=100) cam-b3lyp/6-311+g(d,p) scrf=(solvent=chloroform) scf=xqc). Aromaticity has been assessed using magnetic indices computed at the B3LYP/6-311+G(d,p) level. As magnetic indicators, the anisotropy of the induced current density (ACID) plots[17,18]and vertical and horizontal NICS scans[19-22] were used. ACID plots give a qualitative picture of the ring-current nature so that clockwise and anticlockwise ring-currents indicate aromatic and antiaromatic character, respectively. NICS scans, which consider only π-contributions by employing the σ-model, provide quantitative information about the diatropicity (negative values) and paratropicity (positive values) of the different circuits.

S2.1 Calculated transition energies

Transition energies for **1**, **BP**, and **TPBP** were calculated, and in Table S1, the calculated HOMO-LUMO gaps are compared with the measured data shown in section **S1.4**. Further information about the excitation states can be found in Table S2, and the frontier orbitals are shown in Figure S12.

Table S1. Comparison of the calculated and measured HOMO-LUMO energy gaps in molecules **1**, **BP**, and **TPBP**.

Molecule	CV measurement			UV measurement		Calculation
	$E_{onset,ox}$ (V)	$E_{onset,red}$ (V)	ΔE (eV)	nm	eV	eV
**1**	0.64	-1.56	2.20	– [a]	– [a]	2.14
**BP**	– [b]	– [b]	– [b]	– [b]	– [b]	2.19
**TPBP**	0.49	-1.41	1.90	588	2.11	2.01

[a] Highest energy peak cannot be resolved from other peaks.
[b] There is no available measured data for monobenzopentalene because it is unstable.

Table S2. Calculated excitation states, contributions, energies, and oscillator strengths (*f*) for molecules **1**, **BP**, and **TPBP**.

Molecule **1**

Excitation state	Contributing transitions	Coefficient	Excitatation energy		Oscillator strength
			eV	nm	
1	103 → 105	0.11945	2.135	581	0.0102
	104 (HOMO) → 105 (LUMO)	0.69105			
2	103 → 105	0.68015	3.173	391	0.3470
	104 →105	-0.11172			
3	98 → 105	0.10100	4.420	281	0.1573
	99 → 105	0.48122			
	102 → 105	-0.23084			
	104 → 106	0.32043			
	104 → 109	0.16074			
4	98 → 105	0.39226	4.514	275	0.2307
	99 → 105	0.12708			
	101 → 105	0.11011			
	102 → 105	0.51579			
	103 → 106	0.10457			
5	100 → 105	0.43050	4.647	267	0.0064
	100 → 106	0.11425			
	101 → 105	-0.42991			
	101 → 106	-0.10630			
	103 → 107	0.11571			
	103 → 108	-0.10373			
	104 → 107	-0.12018			
	104 → 108	0.10544			

Molecule **BP**

Excitation state	Contributing transitions	Coefficient	Excitatation energy		Oscillator strength
			eV	nm	
1	40 (HOMO) → 41 (LUMO)	0.70443	2.193	565	0.0013
2	39 → 41	0.67979	3.517	353	0.2558
	40 → 42	0.15373			
3	38 → 41	0.58465	4.578	271	0.0462
	39 → 42	-0.11575			
	40 → 42	-0.32662			
4	38 → 41	0.34648	5.135	241	0.8106
	39 → 41	-0.17781			
	40 → 42	0.57249			
5	37 → 41	0.61512	5.429	228	0.1116
	39 → 42	0.15076			
	40 → 42	-0.12068			
	40 → 46	-0.21604			

Molecule **TPBP**

Excitation state	Contributing transitions	Coefficient	Excitatation energy		Oscillator strength
			eV	nm	
1	100 (HOMO) → 101 (LUMO)	0.69316	2.014	616	0.0061
2	98 → 101	0.14013	3.168	391	0.3606
	99 → 101	0.67156			
3	98 → 101	0.65918	3.980	312	0.3544
	99 → 101	-0.15150			
4	92 → 101	-0.10718	4.378	283	0.2123
	93 → 101	0.37438			
	95 → 101	-0.26384			
	97 → 101	0.20588			
	100 → 102	0.35173			
	100 → 104	-0.16801			
5	94 → 101	0.11650	4.479	277	0.1323
	95 → 101	-0.17691			
	96 → 101	0.62827			
	100 → 102	-0.11422			

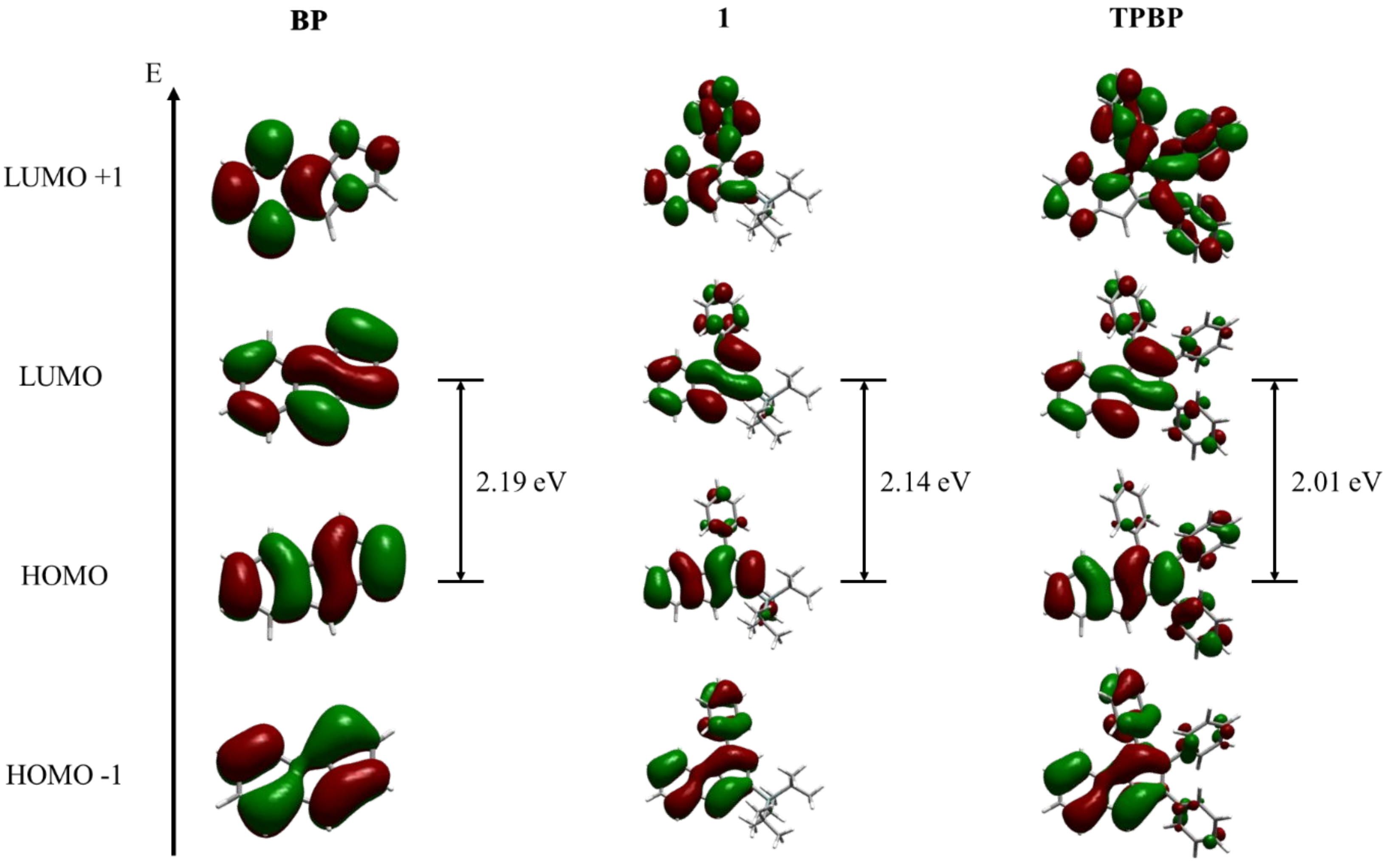

Figure S11. Frontier orbitals of **BP**, **1**, and **TPBP** (at the 0.02 a.u. isosurface) and calculated HOMO-LUMO energy differences in eV.

S2.2 Aromaticity indices

The NICS-*XY* scans of **1**, **BP**, and **TPBP** show similar aromatic characters (Figure S12). Both bench-stable molecules (**1** and **TPBP**) show slightly less antiaromatic character in the pentalene moiety than **BP**. Also, while over the *c* ring the NICS value is similar for both **1** and **TPBP**, over the *b* ring the value is slightly different. Because of this slight difference, we also performed vertical NICS scans in the middle of both five-membered rings of the pentalene moiety (Figure S13).

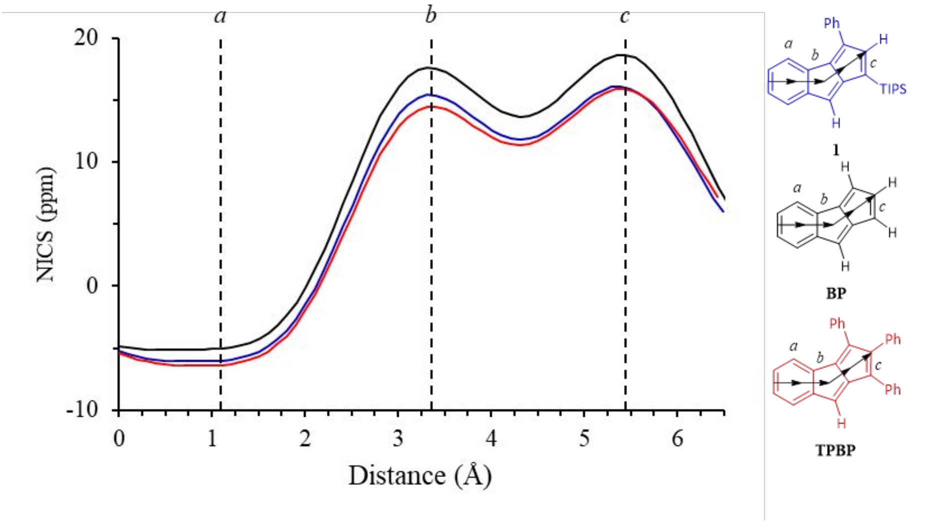

Figure S12. NICS-XY scans of **1**, **BP**, and **TPBP** with arrows showing the scan direction.

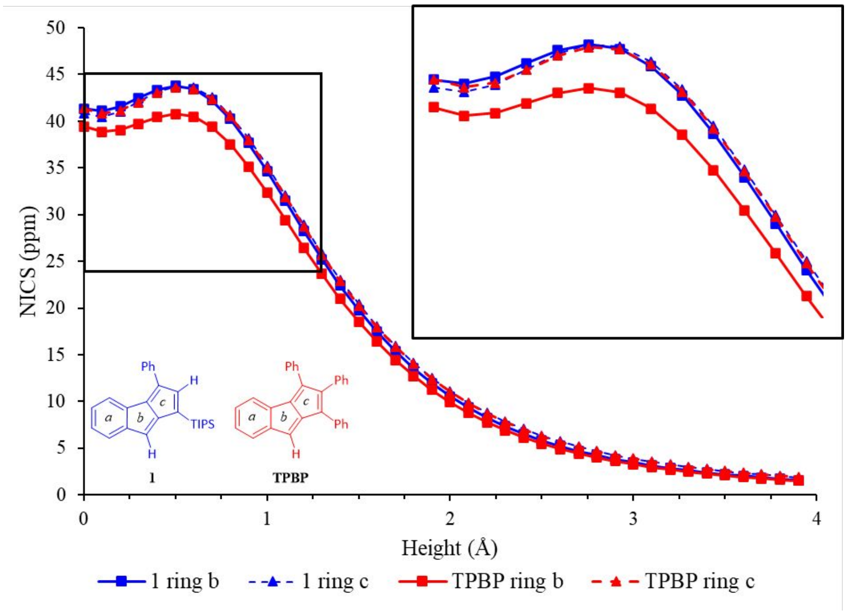

Figure S13. Vertical NICS scans of **1** and **TPBP** on the middle of the *b* and *c* rings.

Based on the vertical NICS scans (Figure S13), the aromatic character of the *c* ring is similar in both molecules. In molecule **1** both five-membered rings have the same aromatic characteristics, however, in **TPBP** the outer (*c*) ring is more antiaromatic than the inner (*b*) ring. For further investigation of the aromaticity in molecules **1**, **BP**, and **TPBP** the corresponding ACID plots were calculated and compared (Figure S14).

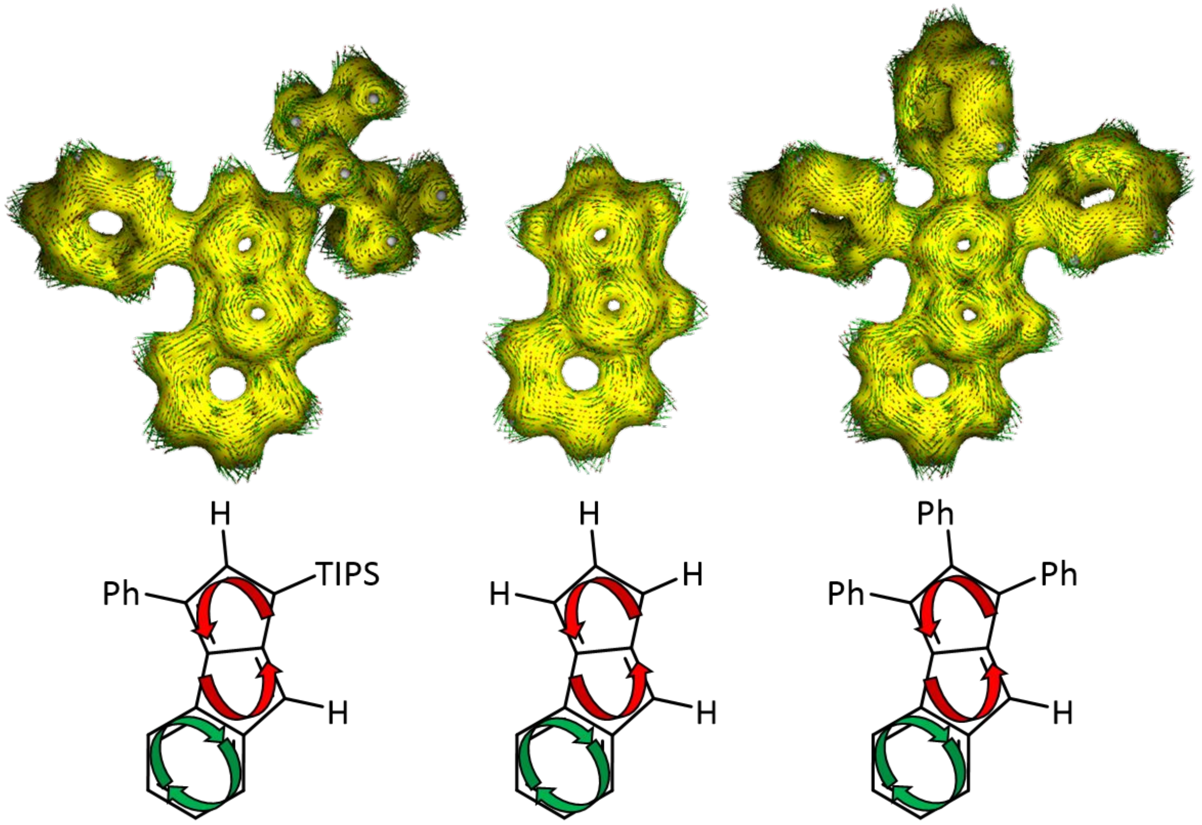

1 **BP** **TPBP**

Figure S14. ACID plots of **1**, **BP**, and **TPBP** with isosurface value 0.025. For clarity, the induced ring currents are represented by bigger arrows within the structures below the plots. The red arrow indicates antiaromatic ring currents, while the green arrows indicate aromatic ring currents.

The ACID plots are in agreement with the NICS results showing antiaromatic character in the pentalene moieties and aromatic character in the annelated benzene rings.

S3 NMR spectra

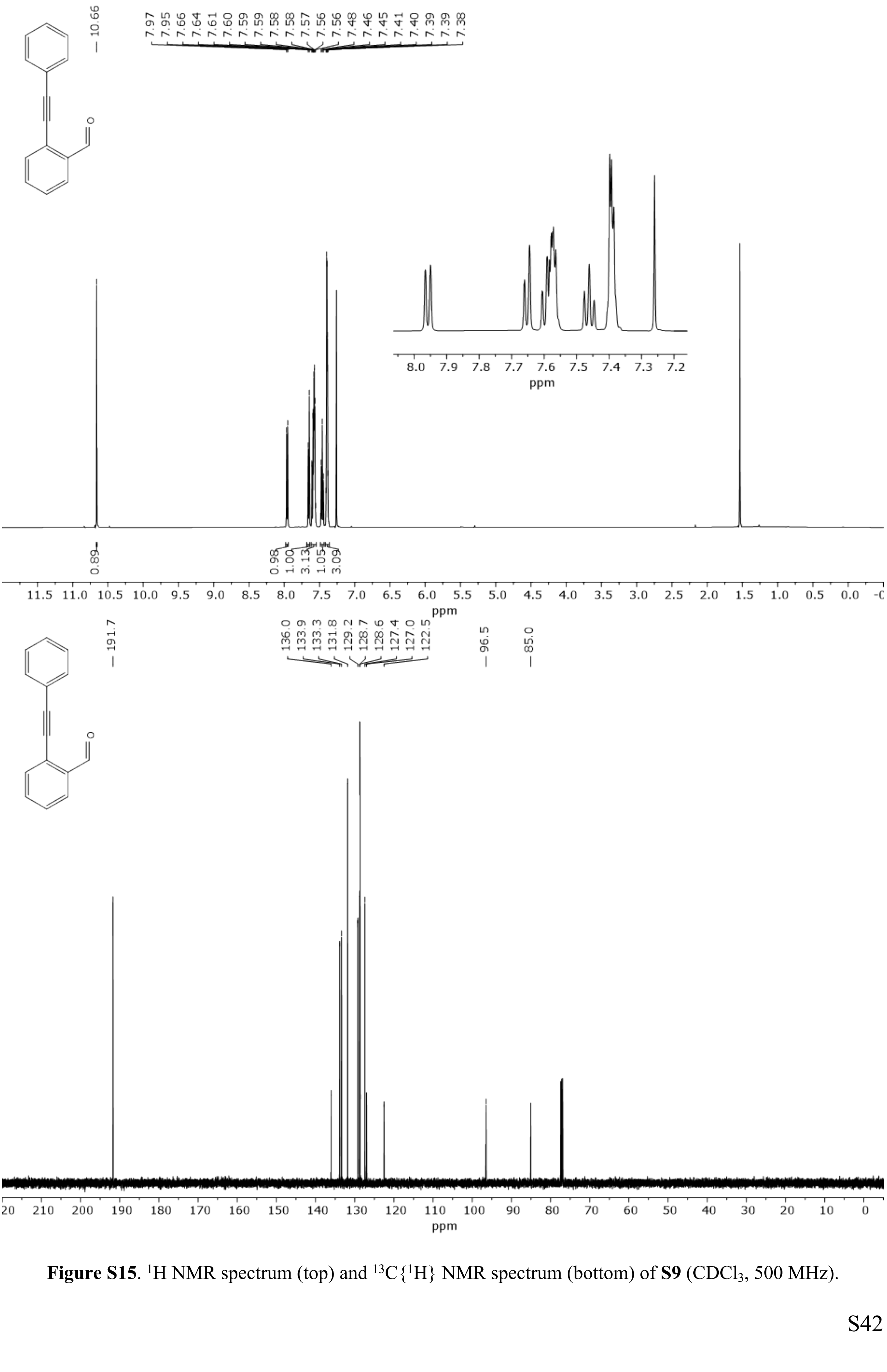

Figure S15. ^{1}H NMR spectrum (top) and $^{13}C\{^{1}H\}$ NMR spectrum (bottom) of **S9** ($CDCl_3$, 500 MHz).

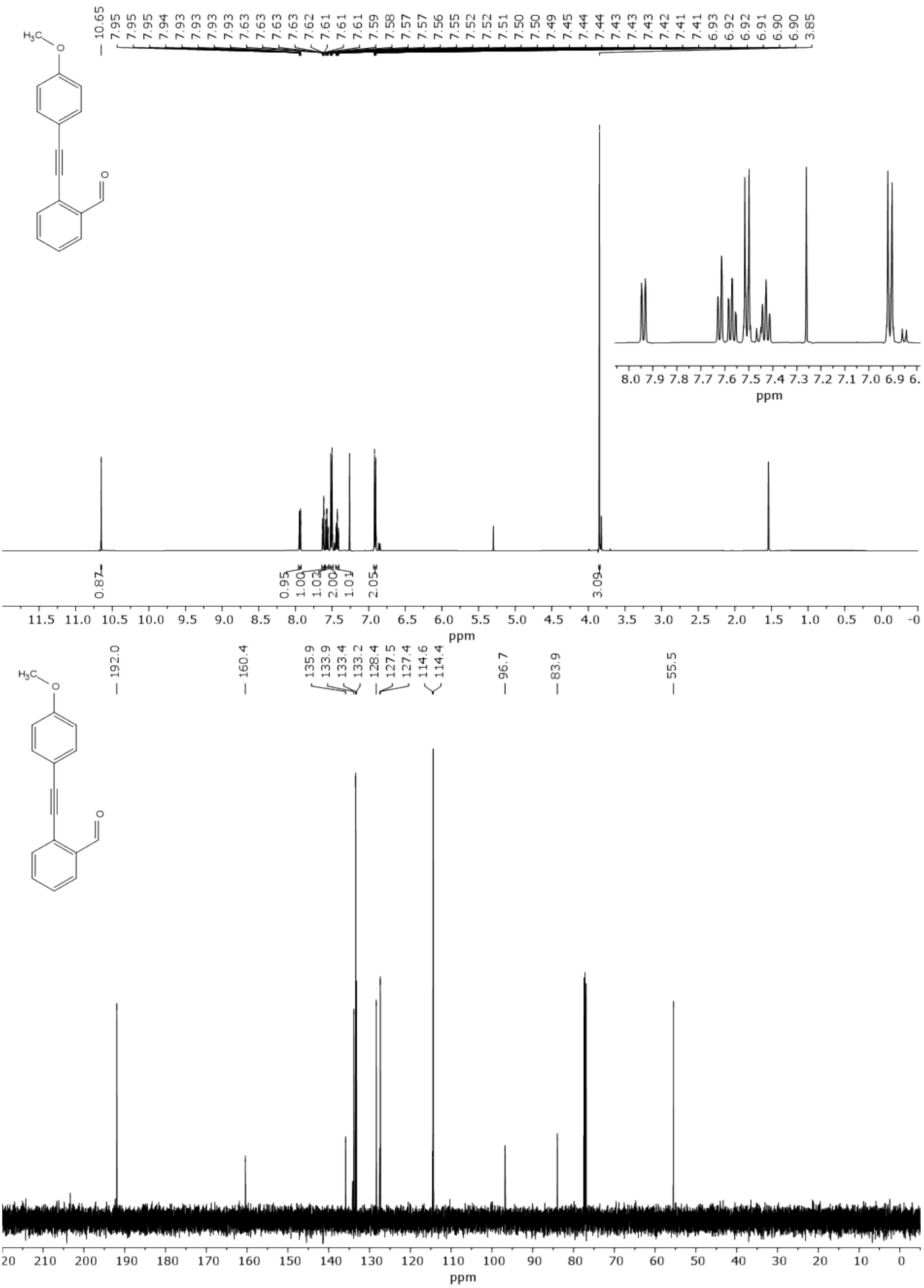

Figure S16. ^{1}H NMR spectrum (top) and $^{13}C\{^{1}H\}$ NMR spectrum (bottom) of **S10** ($CDCl_3$, 500 MHz).

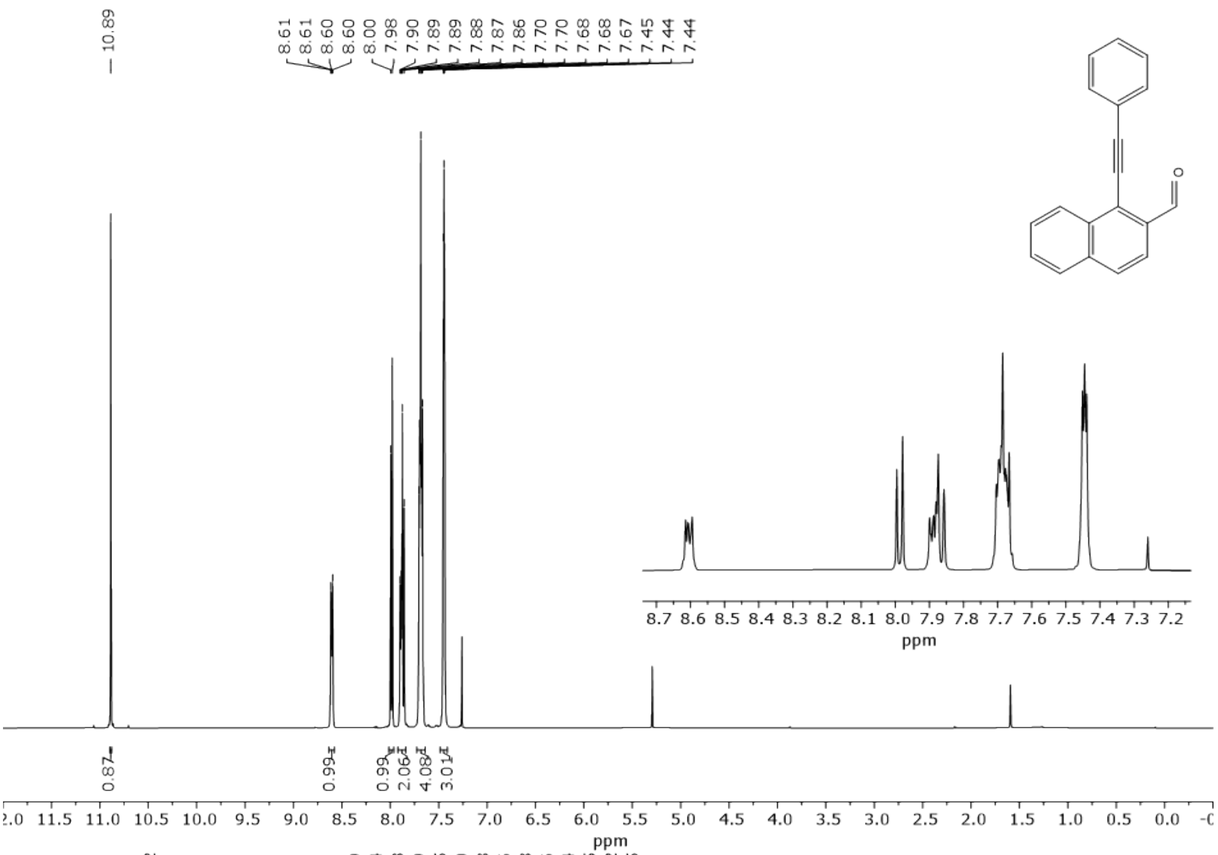

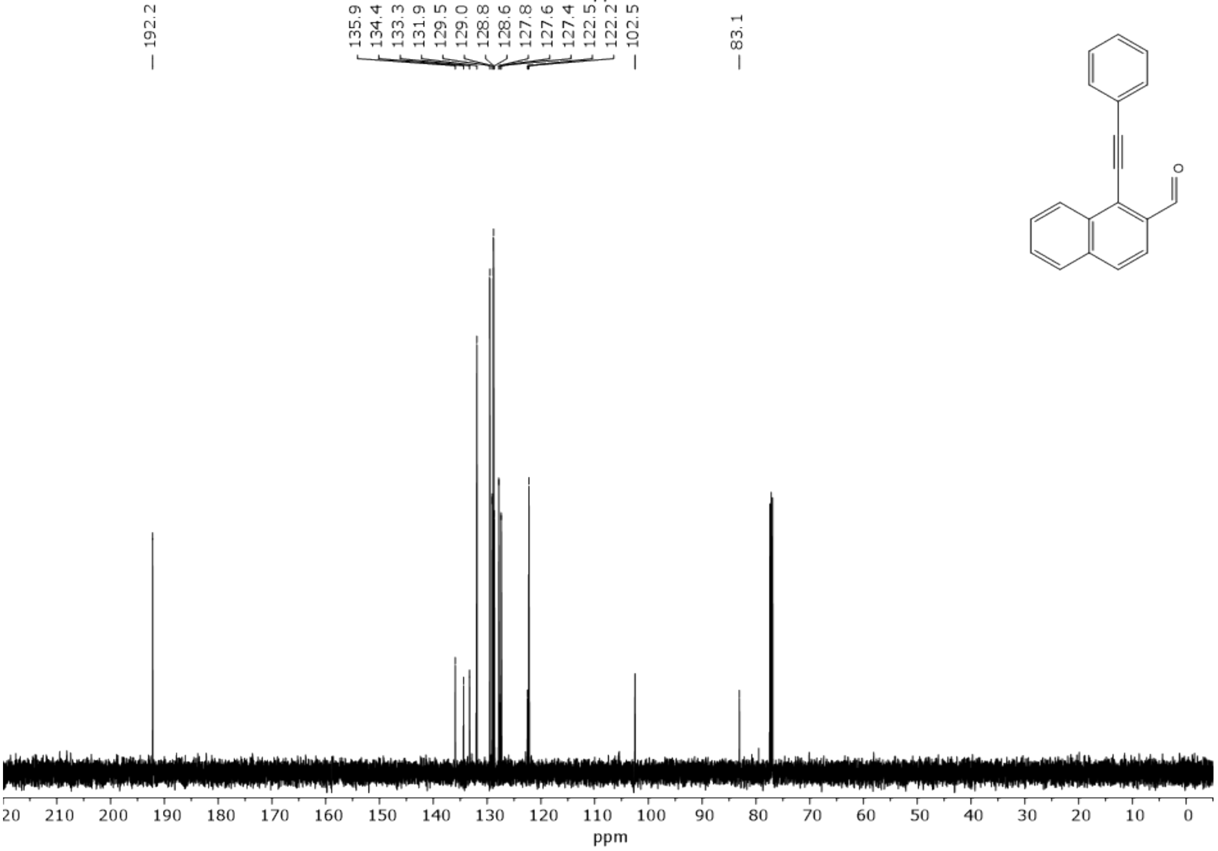

Figure S17. ^{1}H NMR spectrum (top) and ^{13}C{^{1}H} NMR spectrum (bottom) of **S11** ($CDCl_3$, 500 MHz).

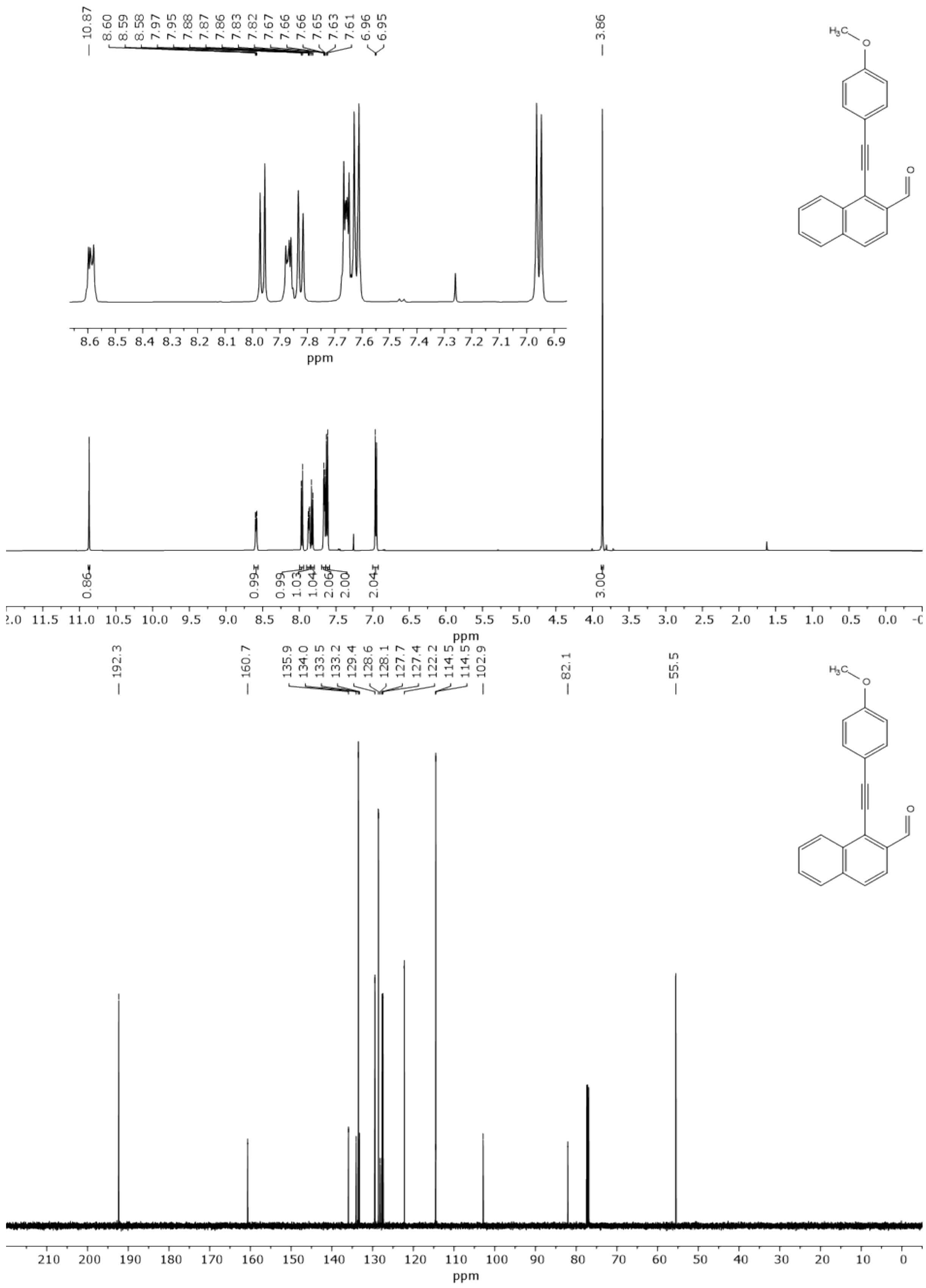

Figure S18. ^{1}H NMR spectrum (top) and $^{13}C\{^{1}H\}$ NMR spectrum (bottom) of **S12** ($CDCl_3$, 500 MHz).

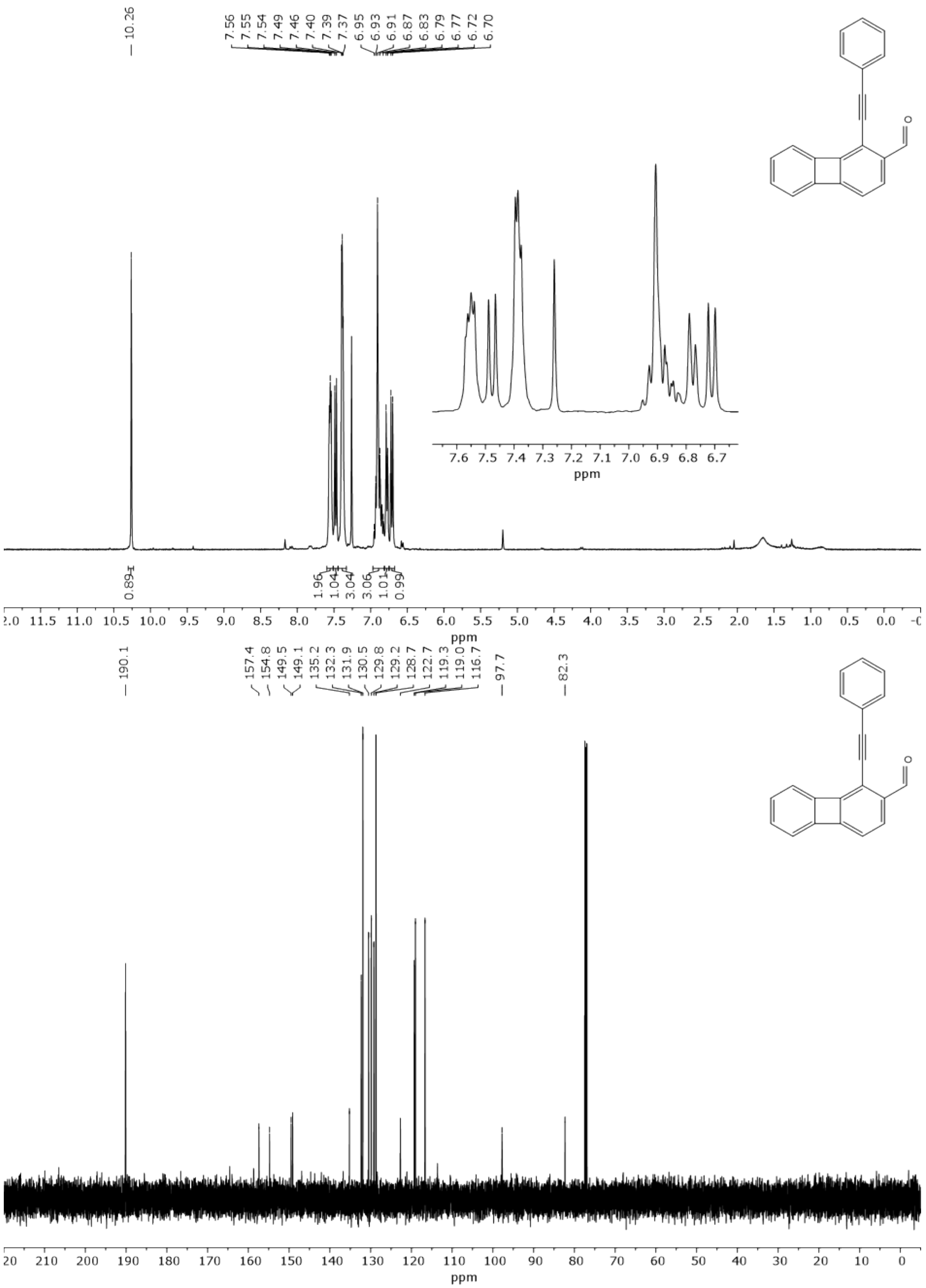

Figure S19. ^{1}H NMR spectrum (top) and $^{13}C\{^{1}H\}$ NMR spectrum (bottom) of **S13** ($CDCl_3$, 300 MHz).

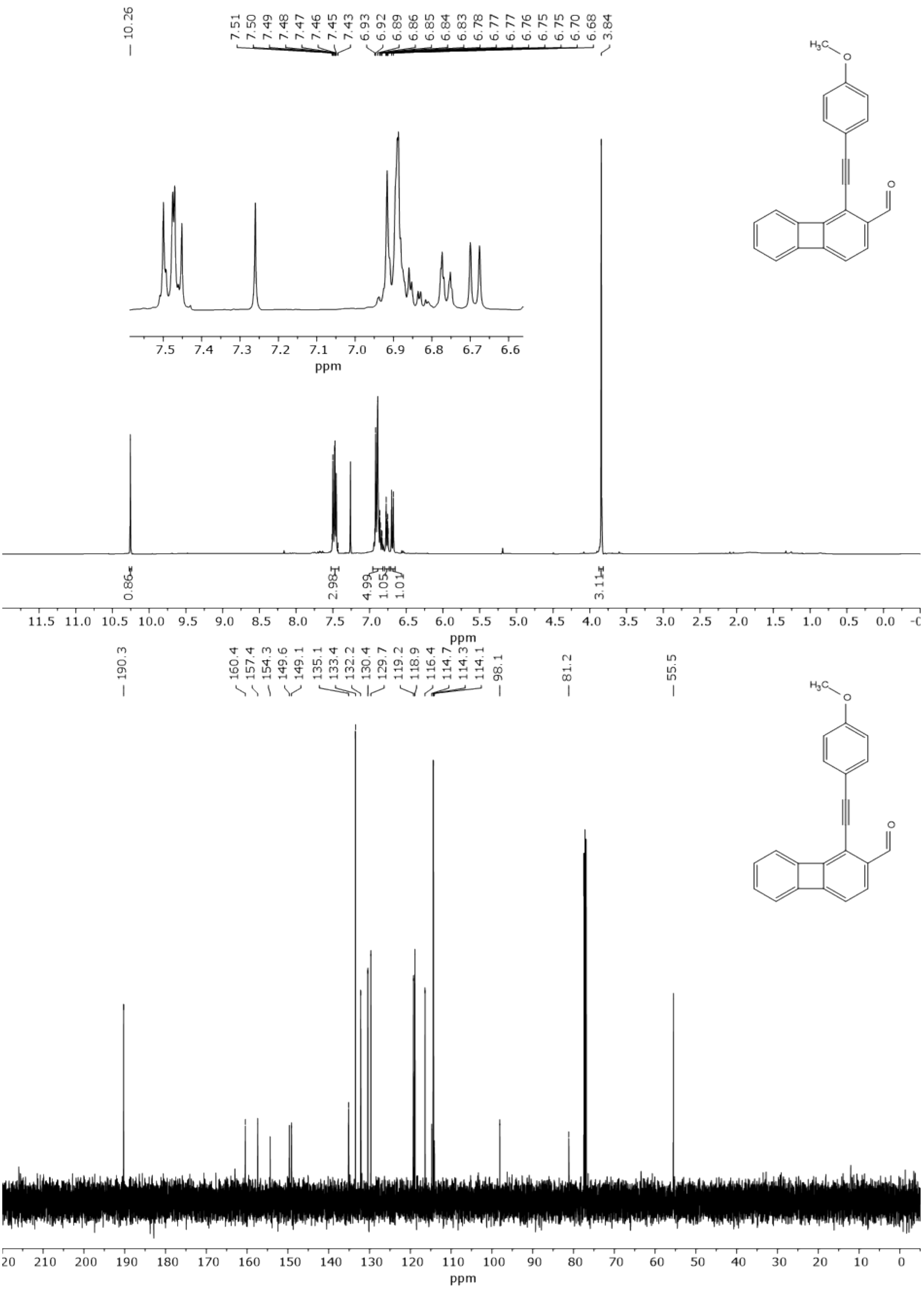

Figure S20. ^{1}H NMR spectrum (top) and $^{13}C\{^{1}H\}$ NMR spectrum (bottom) of **S14** ($CDCl_3$, 300 MHz).

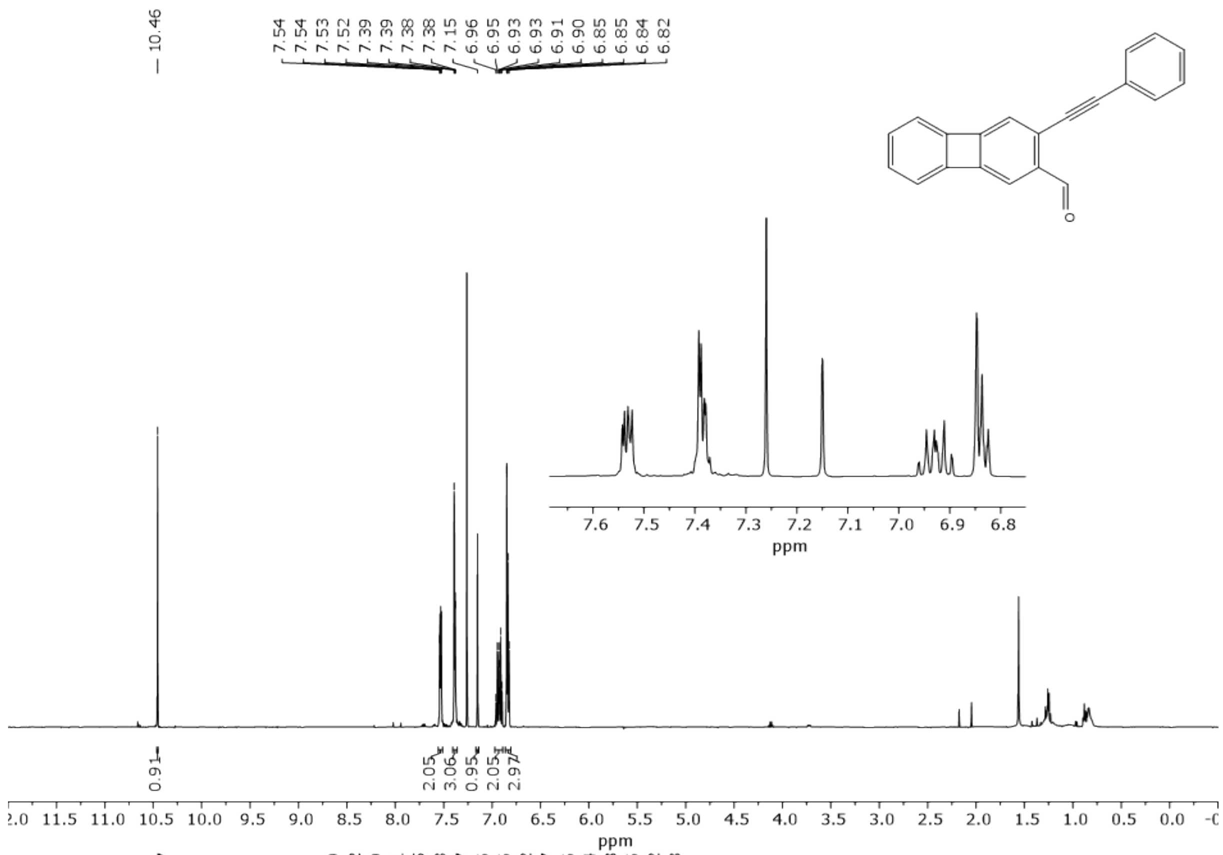

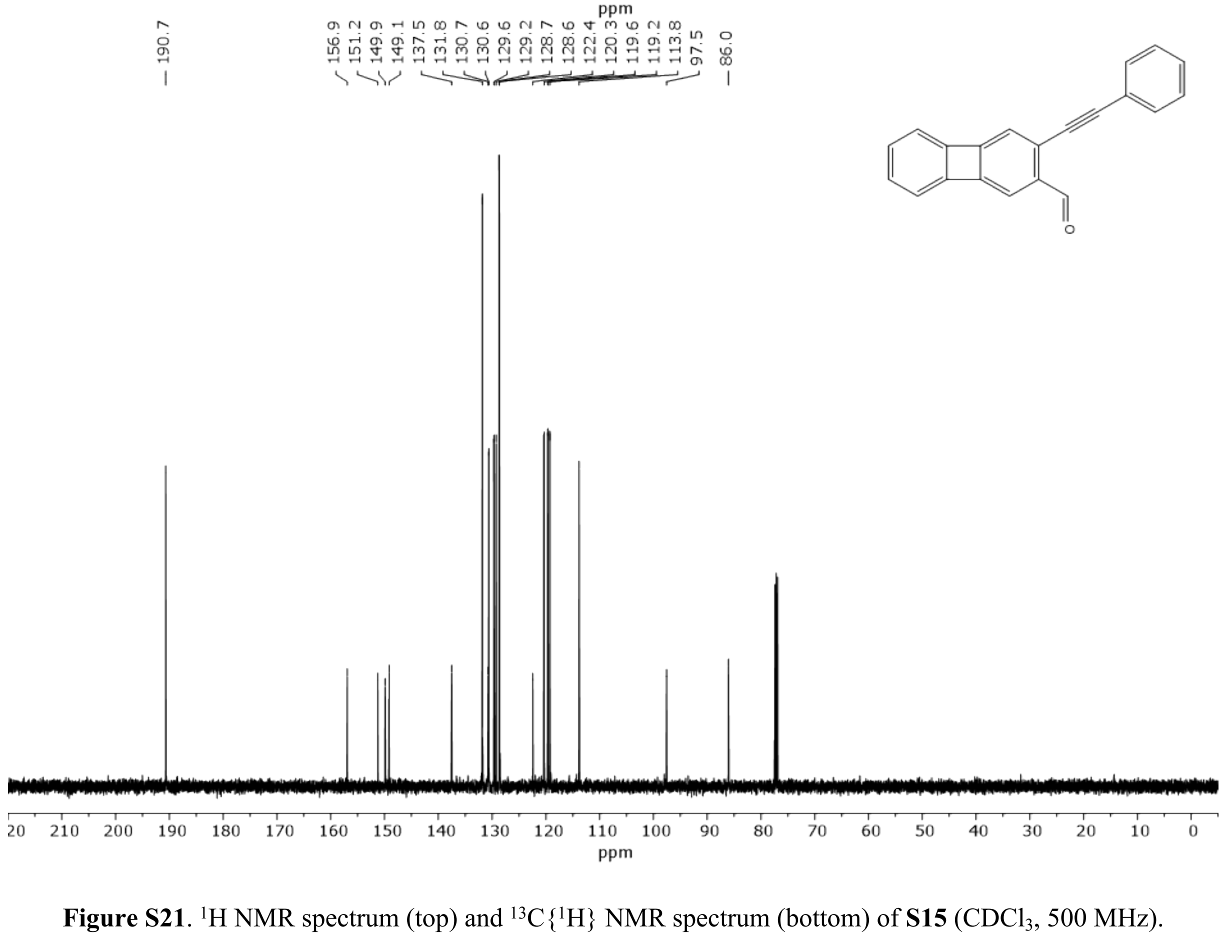

Figure S21. ^{1}H NMR spectrum (top) and $^{13}C\{^{1}H\}$ NMR spectrum (bottom) of **S15** ($CDCl_3$, 500 MHz).

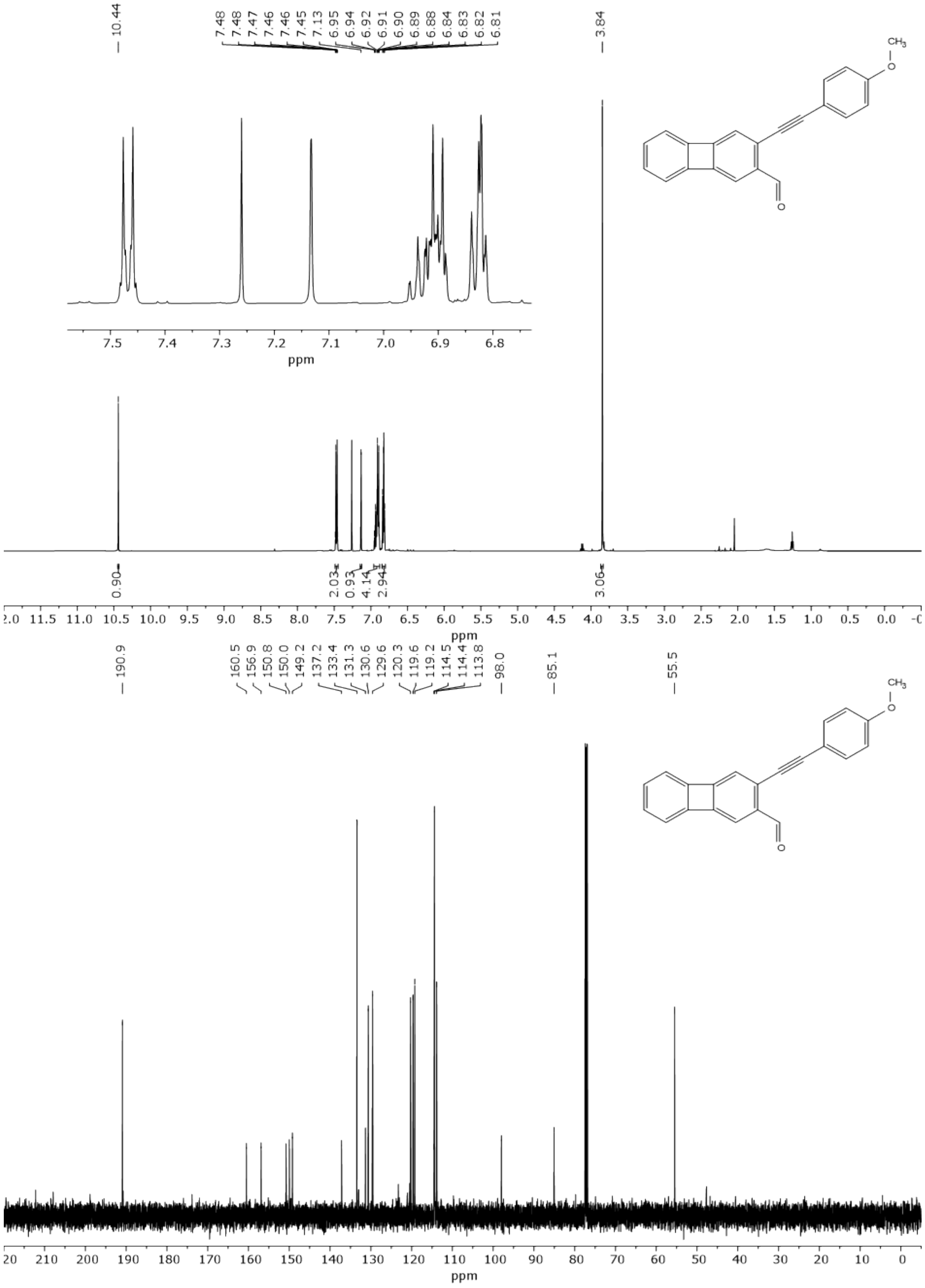

Figure S22. ^{1}H NMR spectrum (top) and $^{13}C\{^{1}H\}$ NMR spectrum (bottom) of **S16** ($CDCl_3$, 500 MHz).

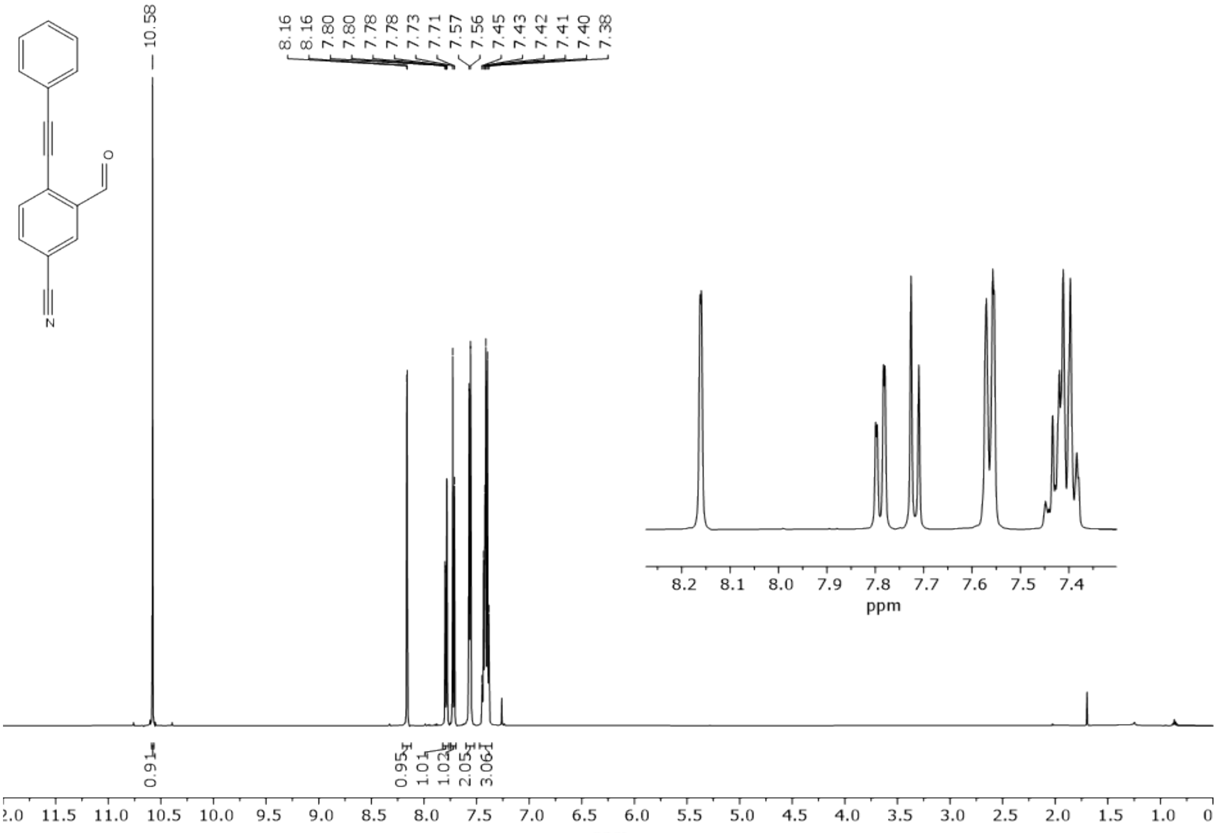

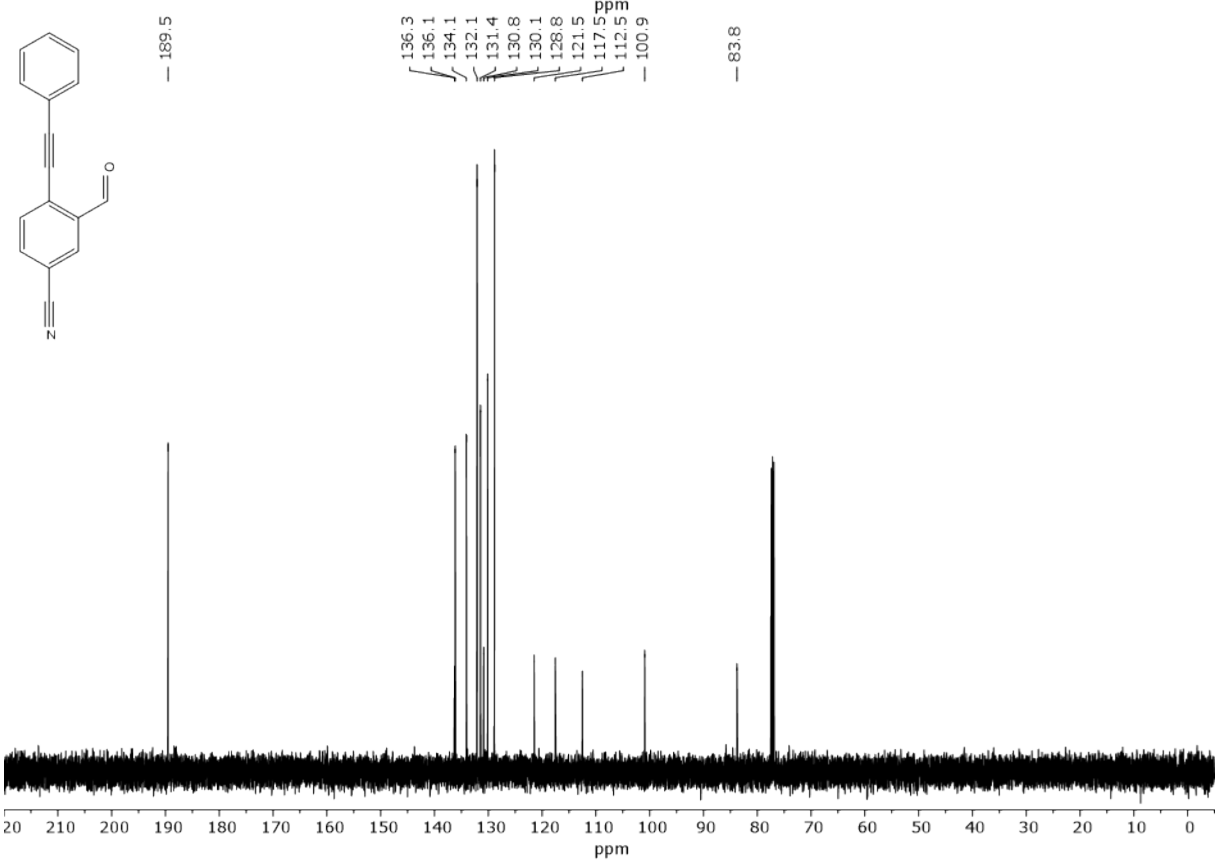

Figure S23. ^{1}H NMR spectrum (top) and $^{13}C\{^{1}H\}$ NMR spectrum (bottom) of **S17** ($CDCl_3$, 500 MHz).

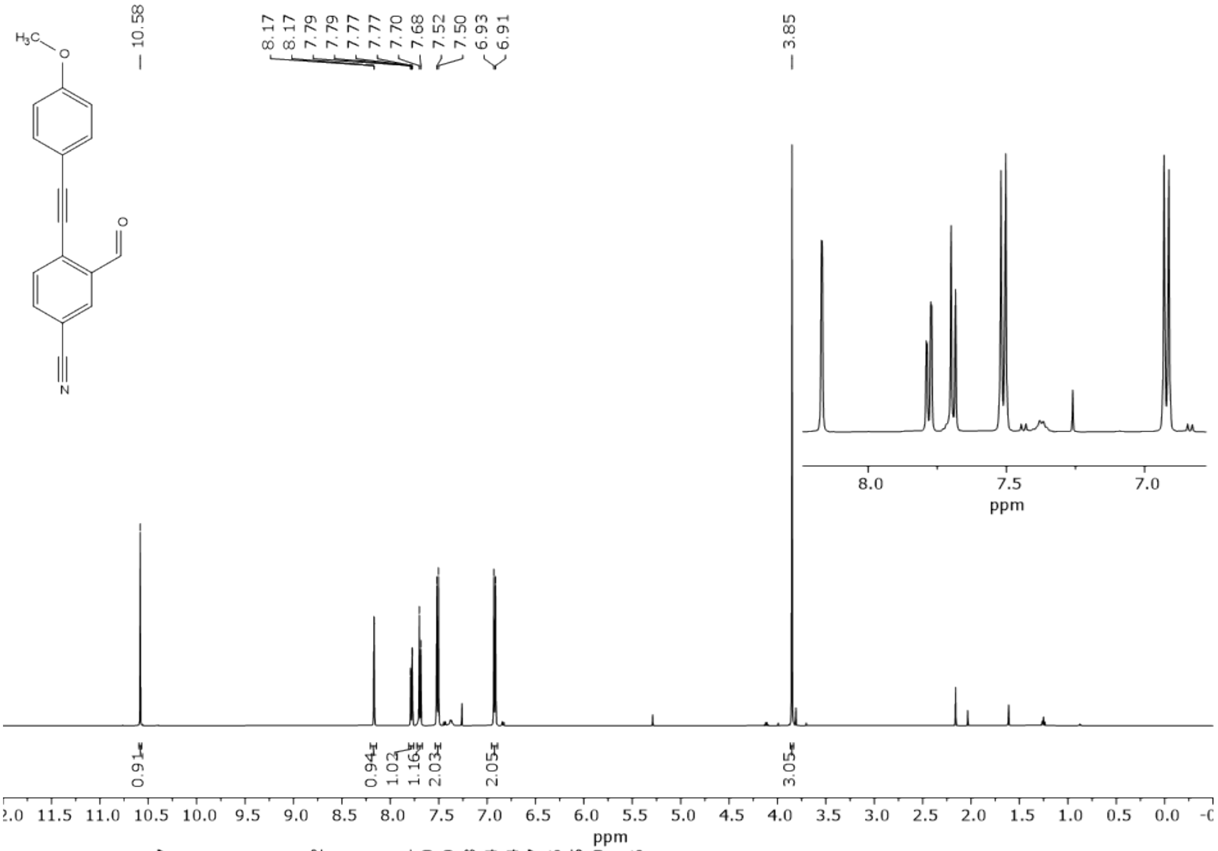

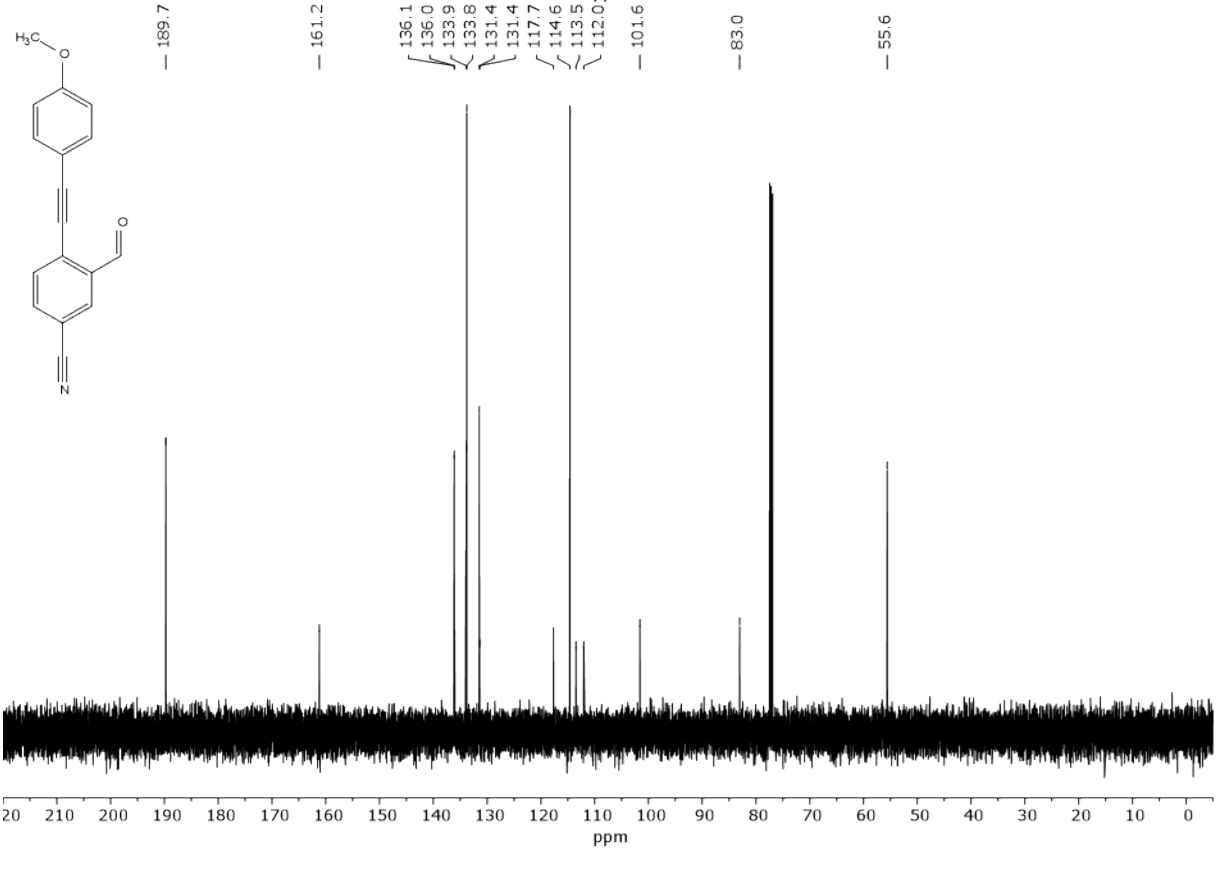

Figure S24. ^{1}H NMR spectrum (top) and $^{13}C\{^{1}H\}$ NMR spectrum (bottom) of **S18** ($CDCl_3$, 500 MHz).

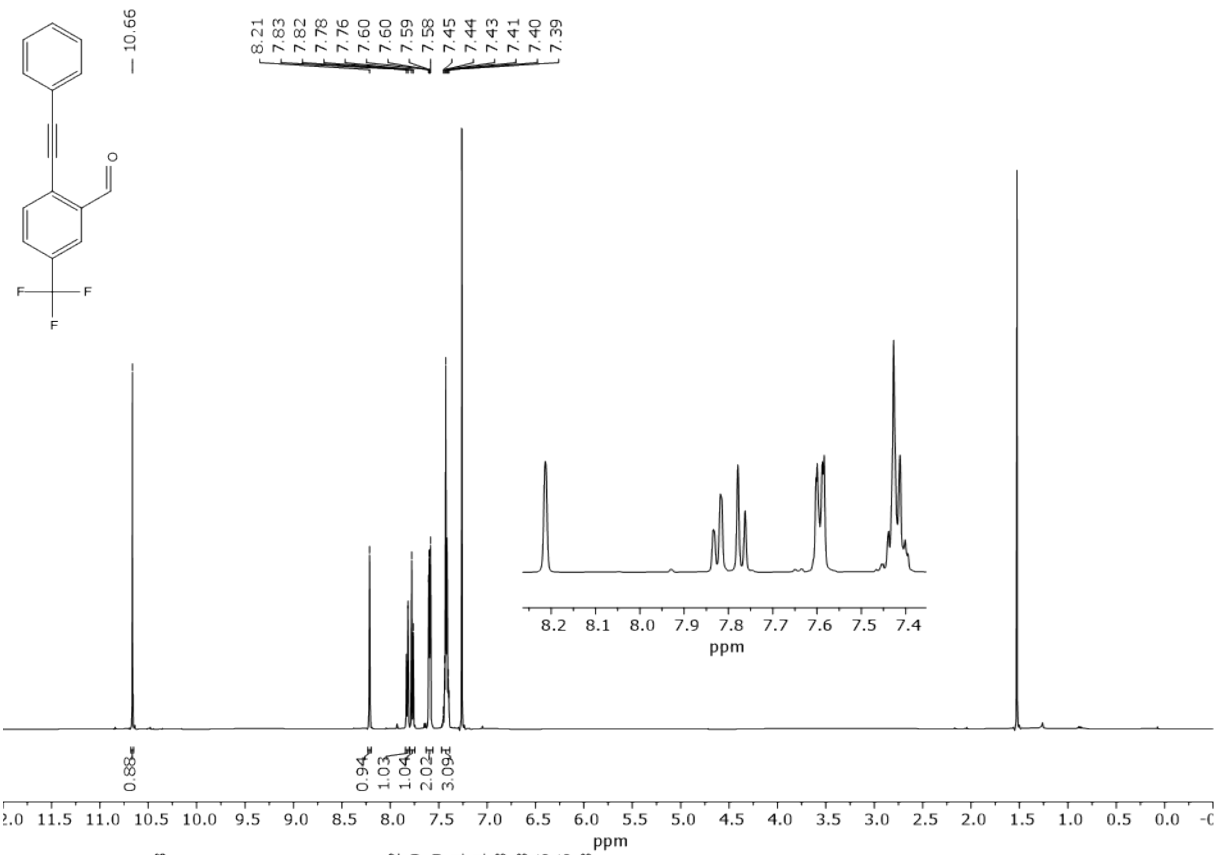

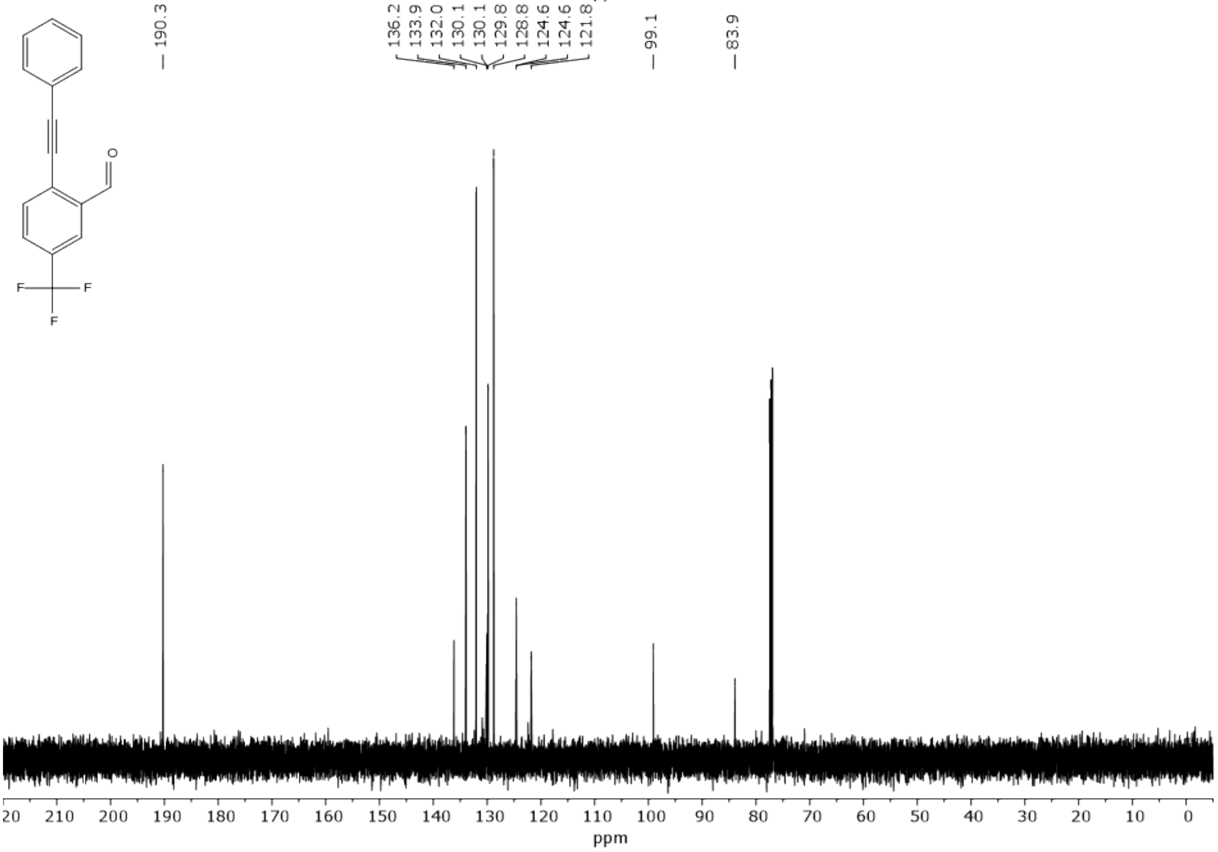

Figure S25. ^{1}H NMR spectrum (top) and $^{13}C\{^{1}H\}$ NMR spectrum (bottom) of **S19** ($CDCl_3$, 500 MHz).

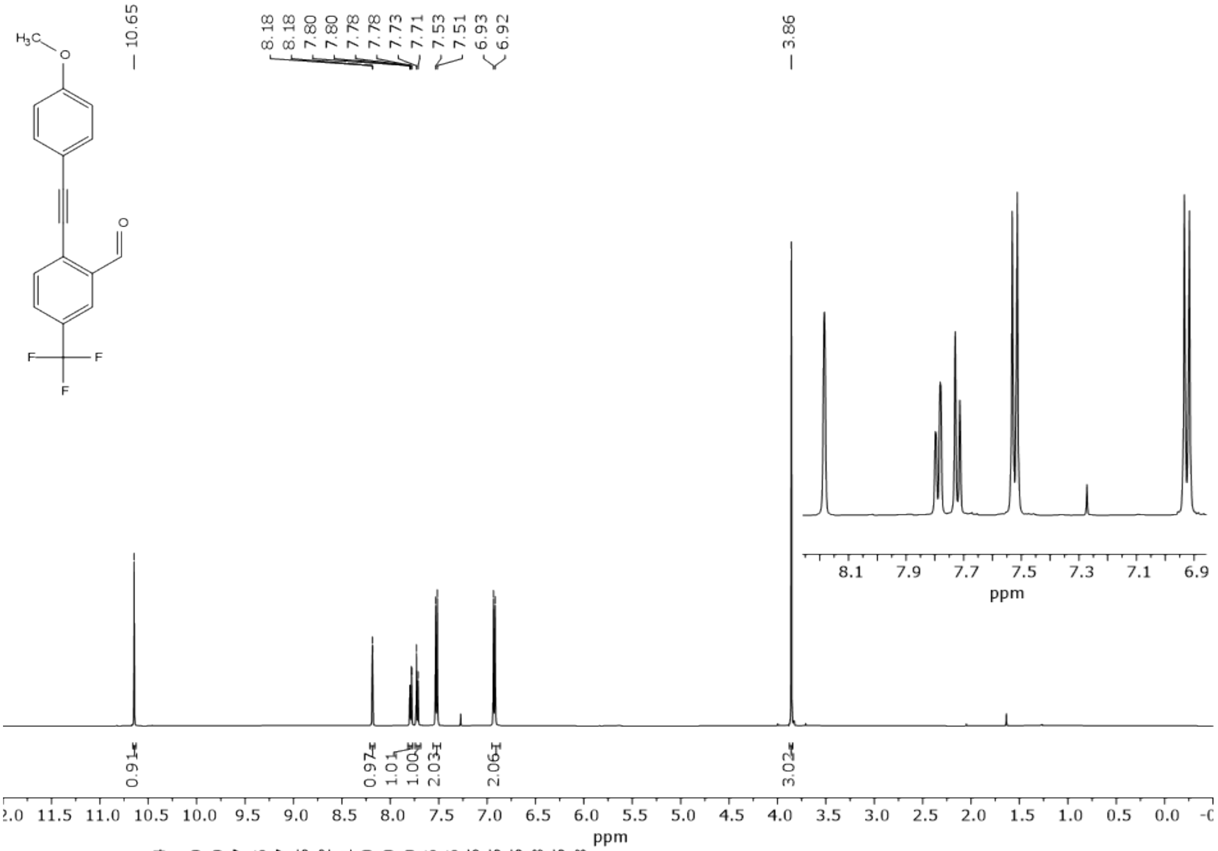

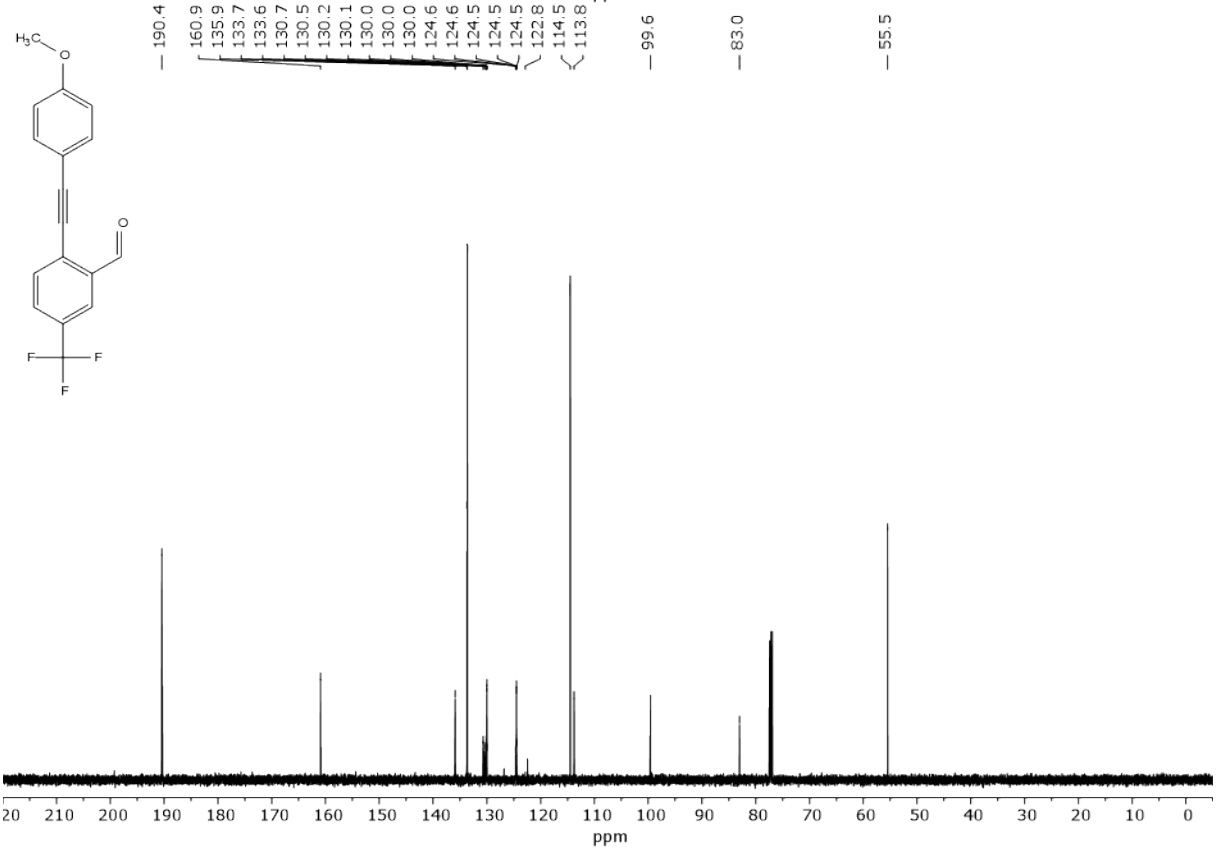

Figure S26. ^{1}H NMR spectrum (top) and $^{13}C\{^{1}H\}$ NMR spectrum (bottom) of **S20** ($CDCl_3$, 500 MHz).

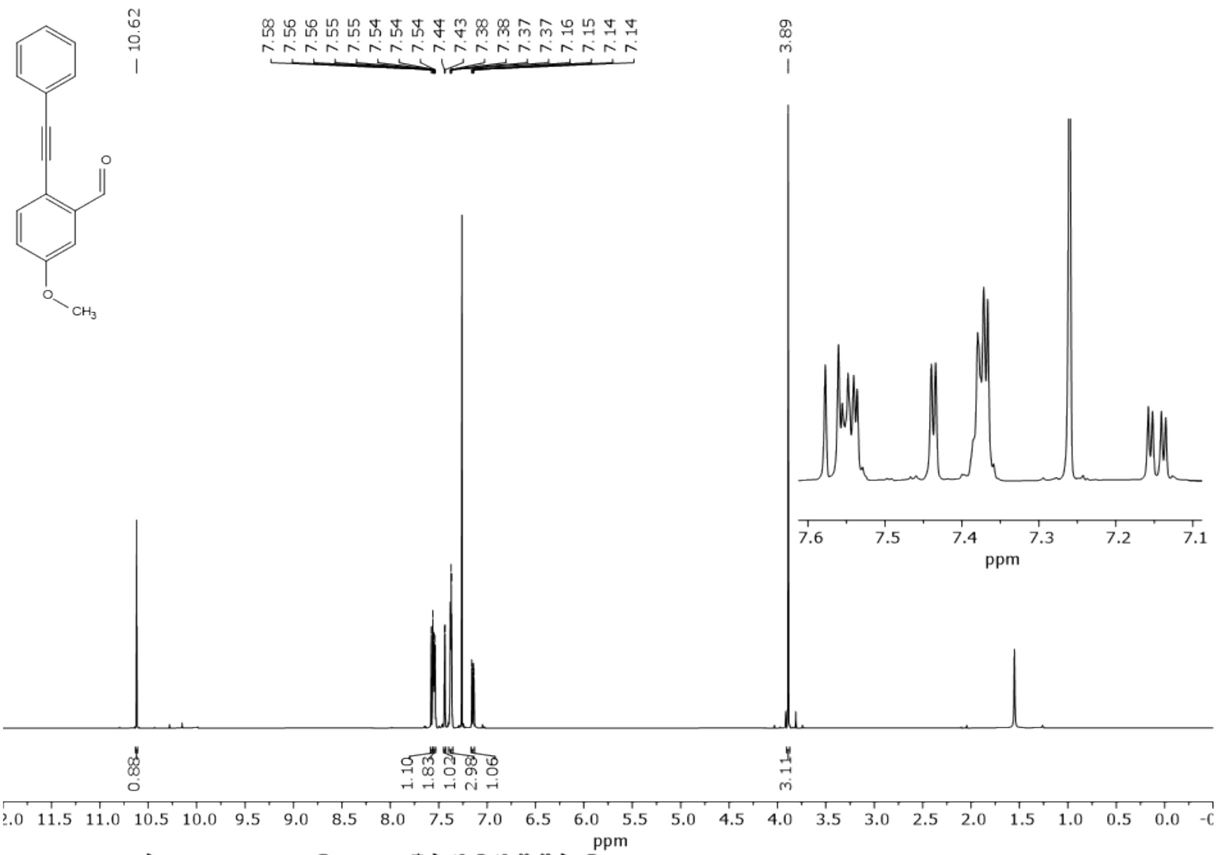

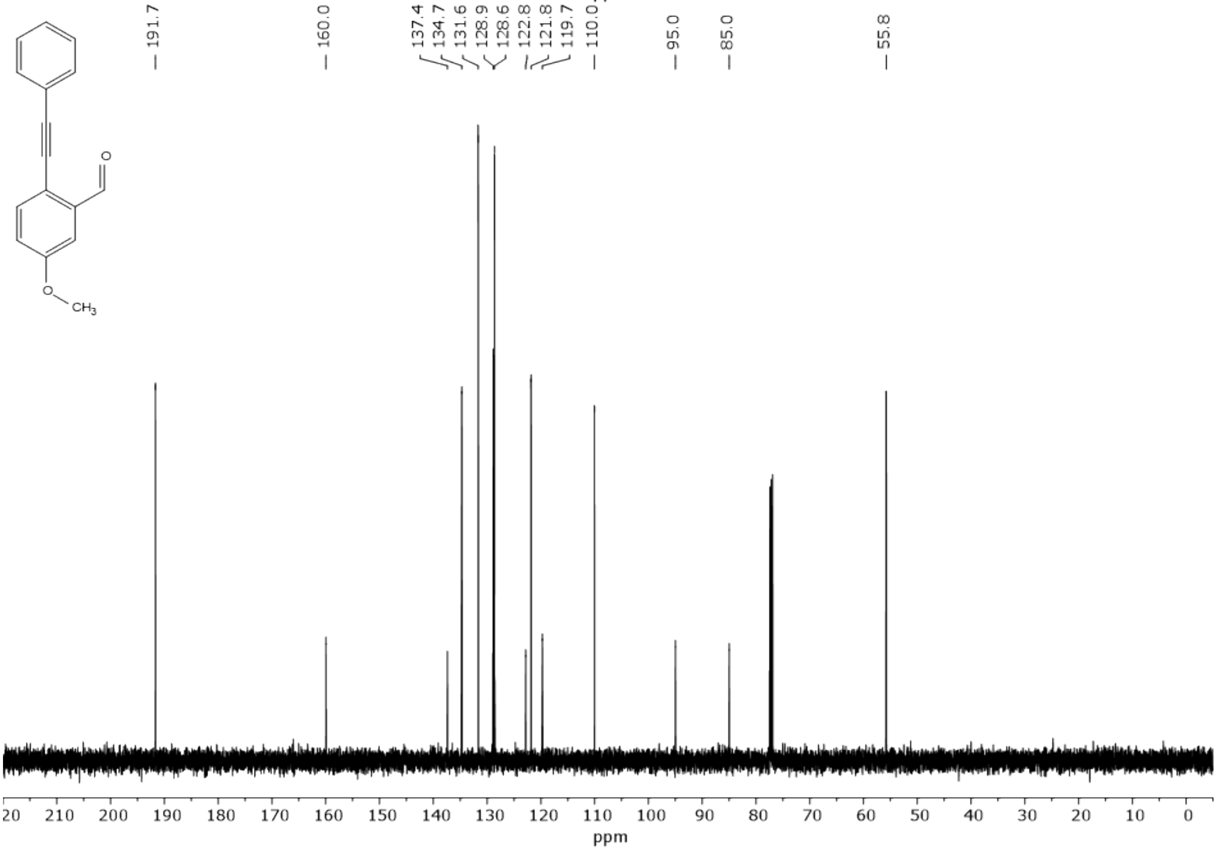

Figure S27. ^{1}H NMR spectrum (top) and $^{13}C\{^{1}H\}$ NMR spectrum (bottom) of **S21** ($CDCl_3$, 500 MHz).

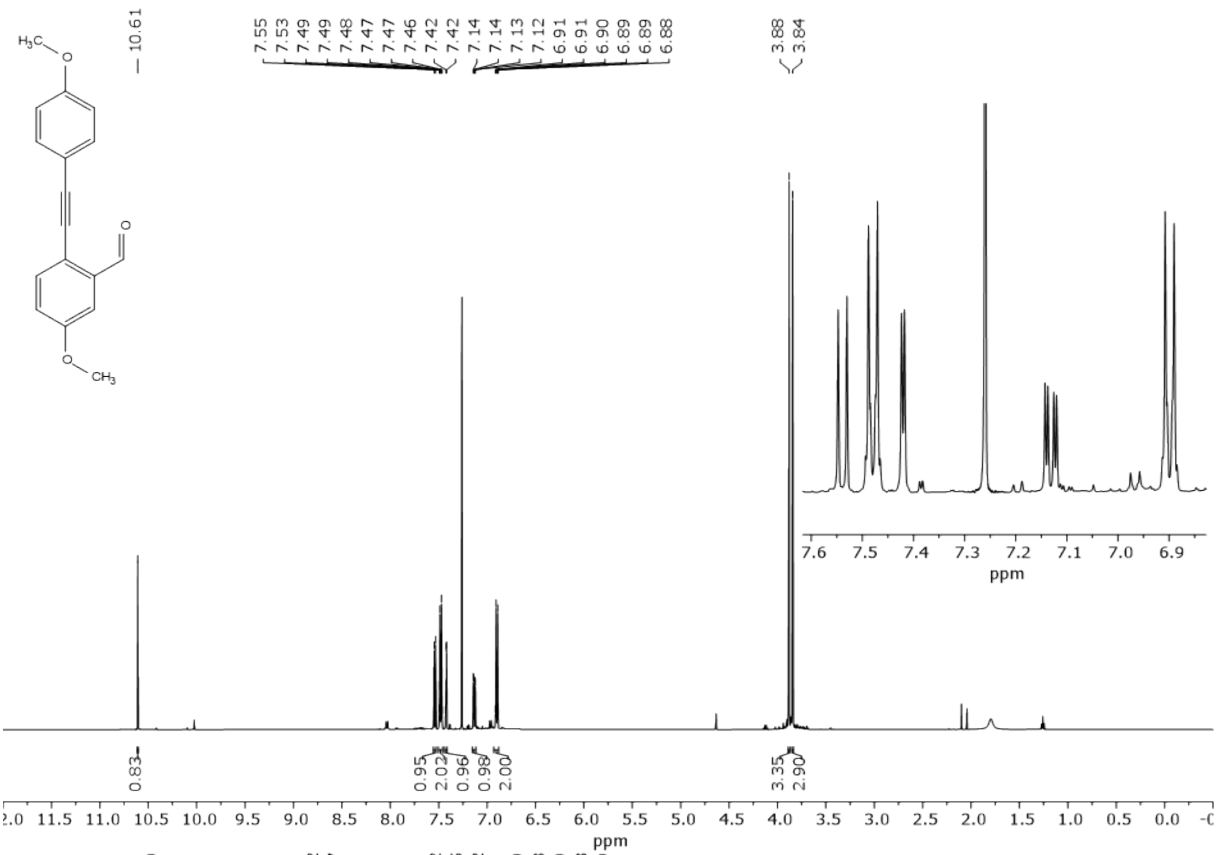

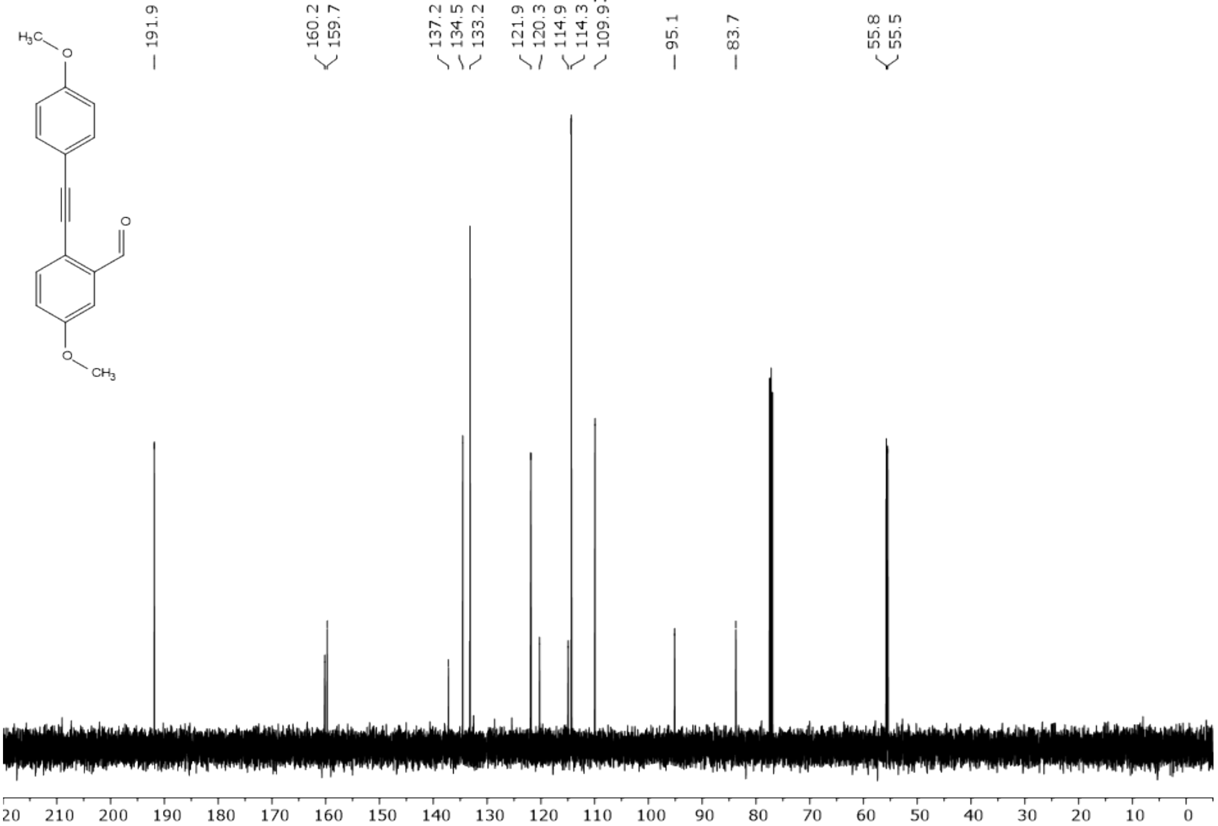

Figure S28. ^{1}H NMR spectrum (top) and ^{13}C{^{1}H} NMR spectrum (bottom) of **S22** ($CDCl_3$, 500 MHz).

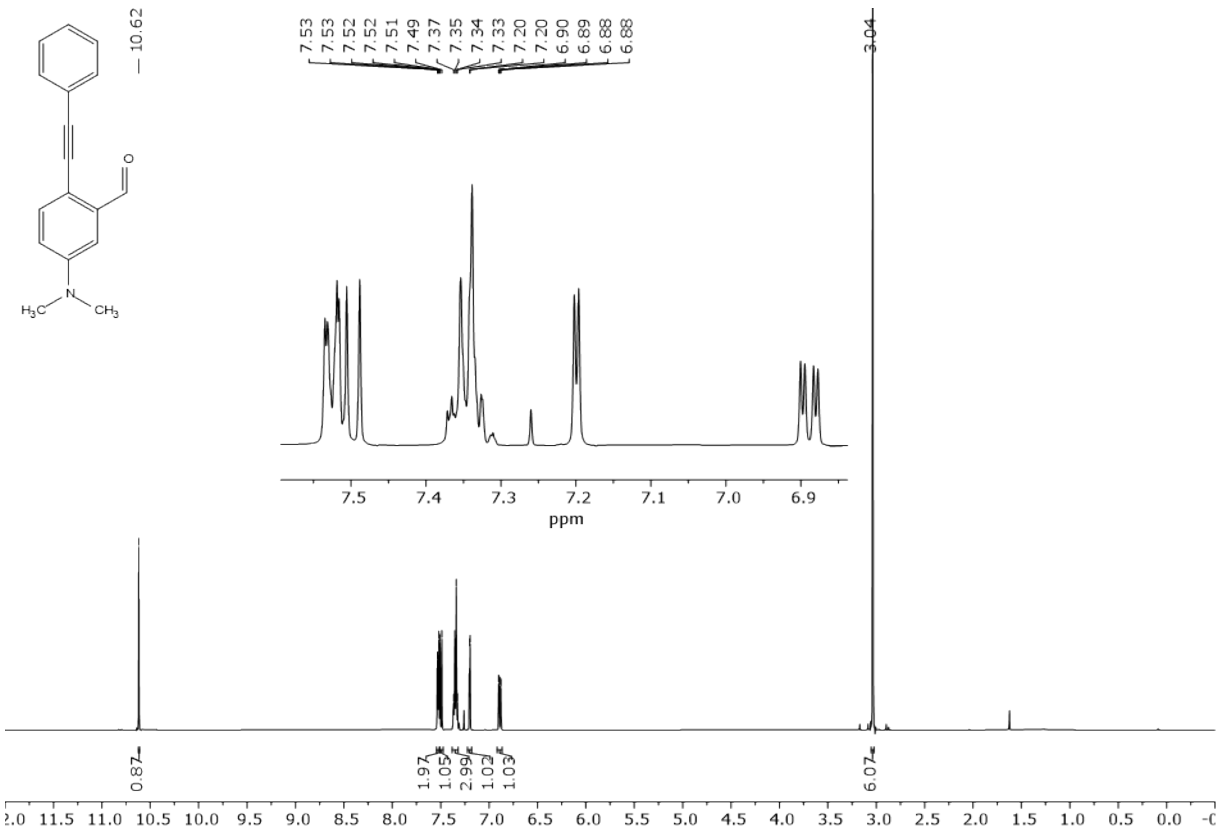

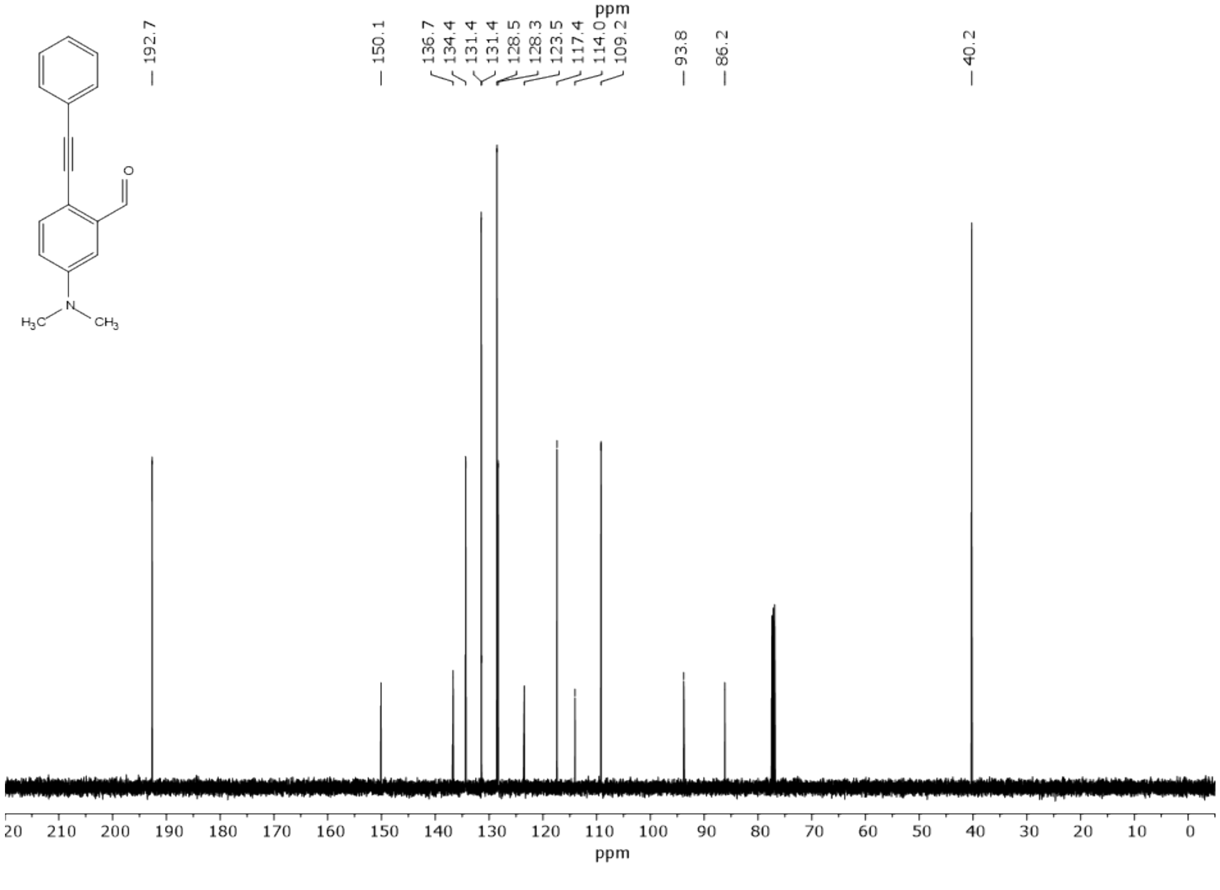

Figure S29. ^{1}H NMR spectrum (top) and $^{13}C\{^{1}H\}$ NMR spectrum (bottom) of **S23** ($CDCl_3$, 500 MHz).

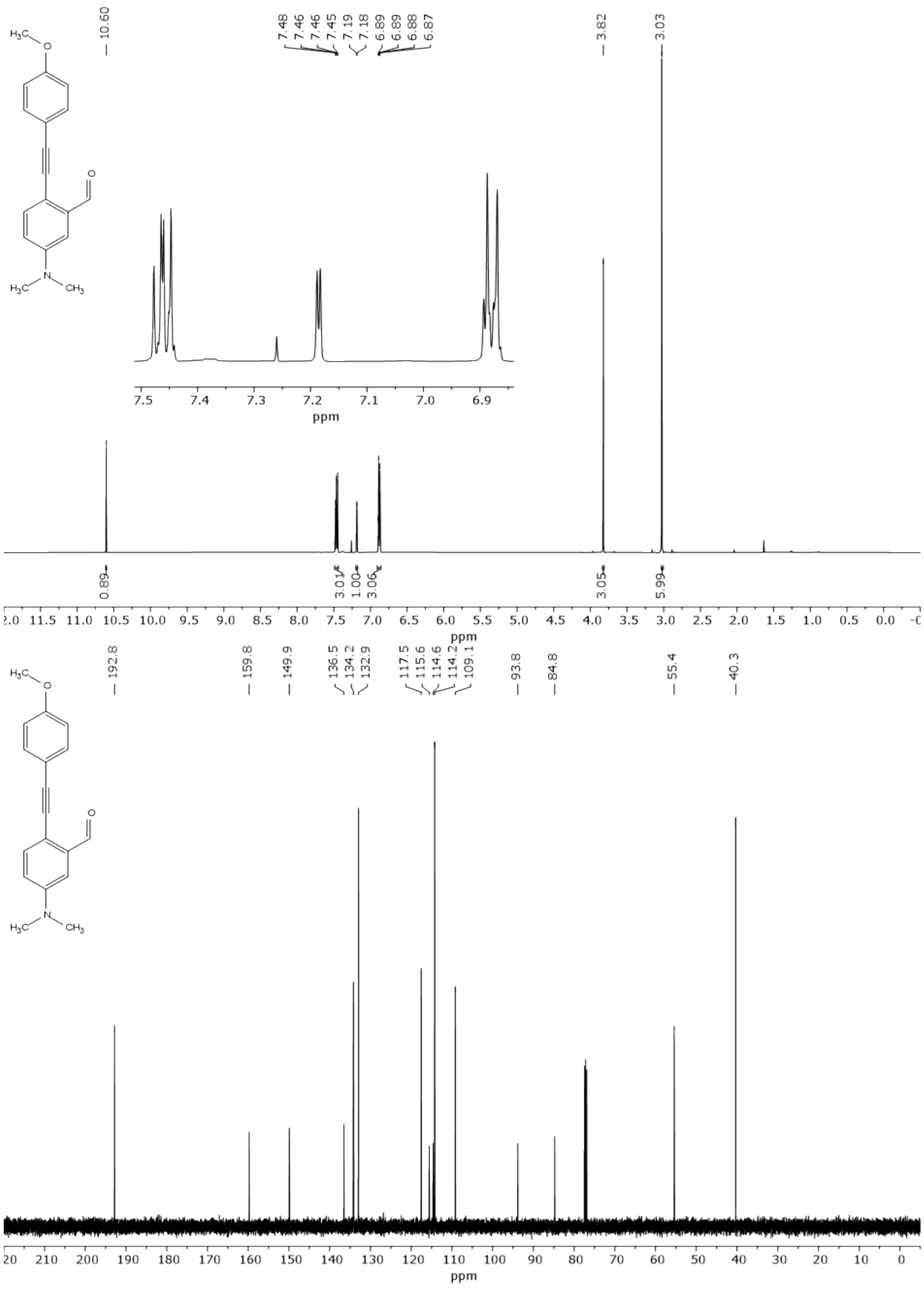

Figure S30. ^{1}H NMR spectrum (top) and $^{13}C\{^{1}H\}$ NMR spectrum (bottom) of **S24** ($CDCl_3$, 500 MHz).

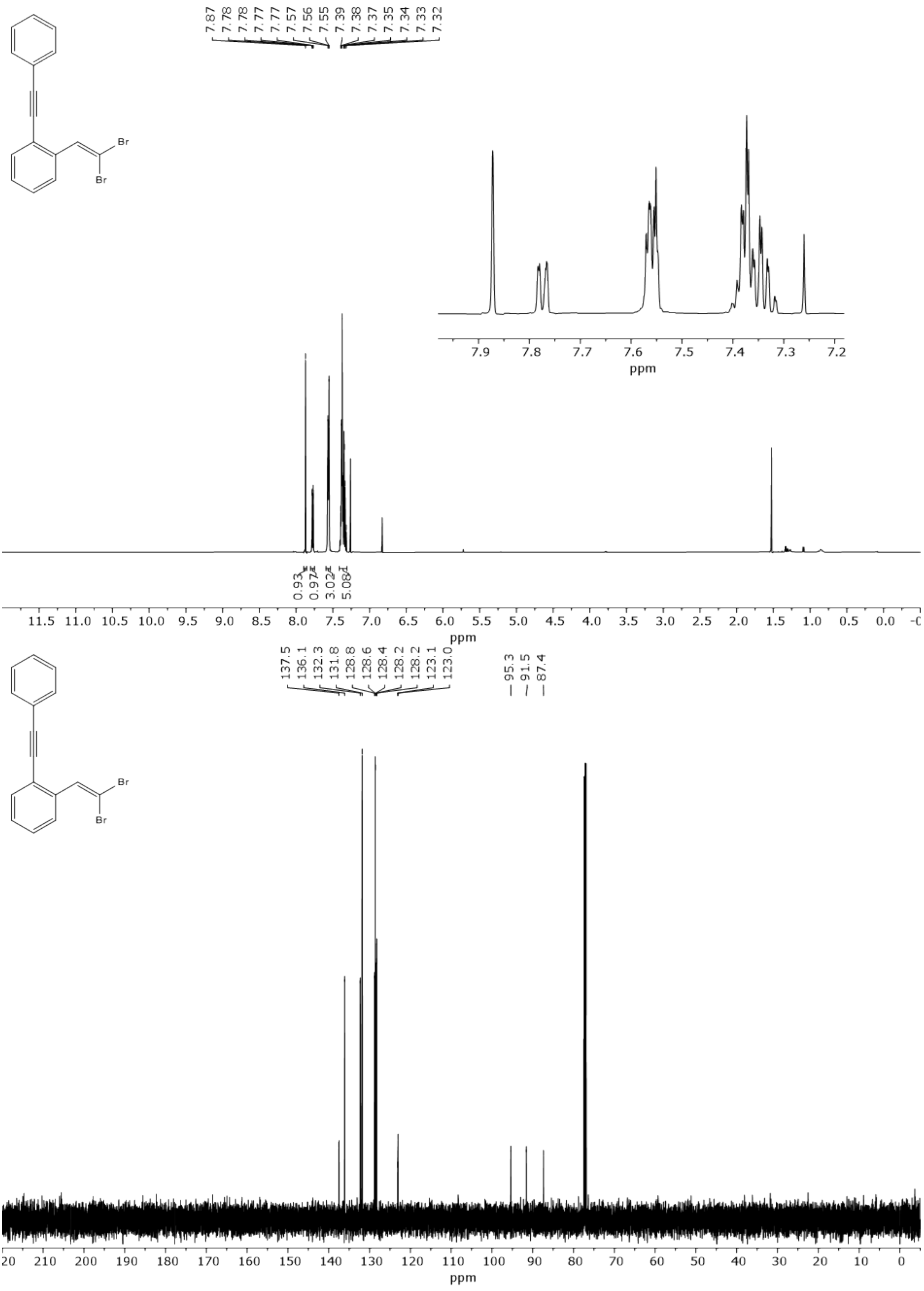

Figure S31. ^{1}H NMR spectrum (top) and $^{13}C\{^{1}H\}$ NMR spectrum (bottom) of **S25** ($CDCl_3$, 500 MHz).

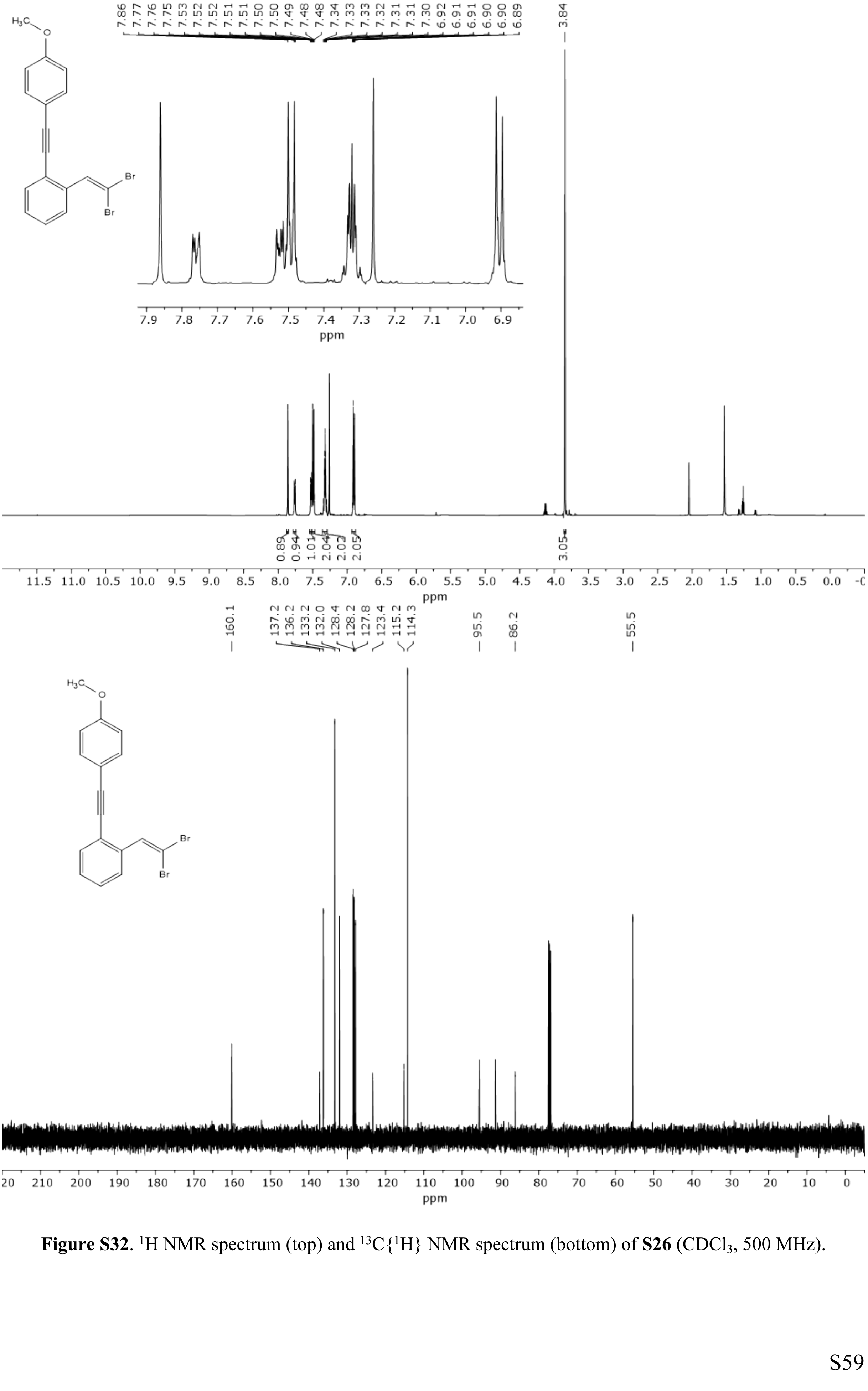

Figure S32. ^{1}H NMR spectrum (top) and $^{13}C\{^{1}H\}$ NMR spectrum (bottom) of **S26** ($CDCl_3$, 500 MHz).

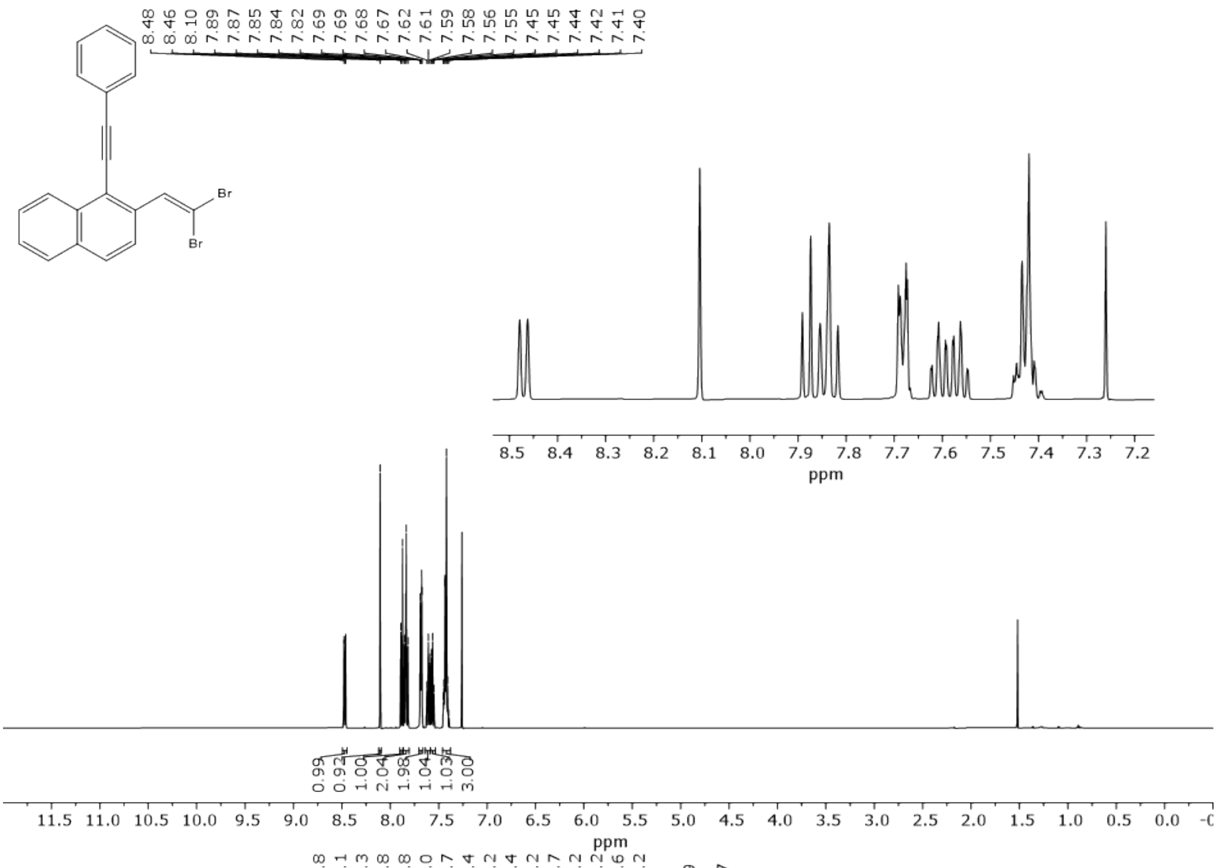

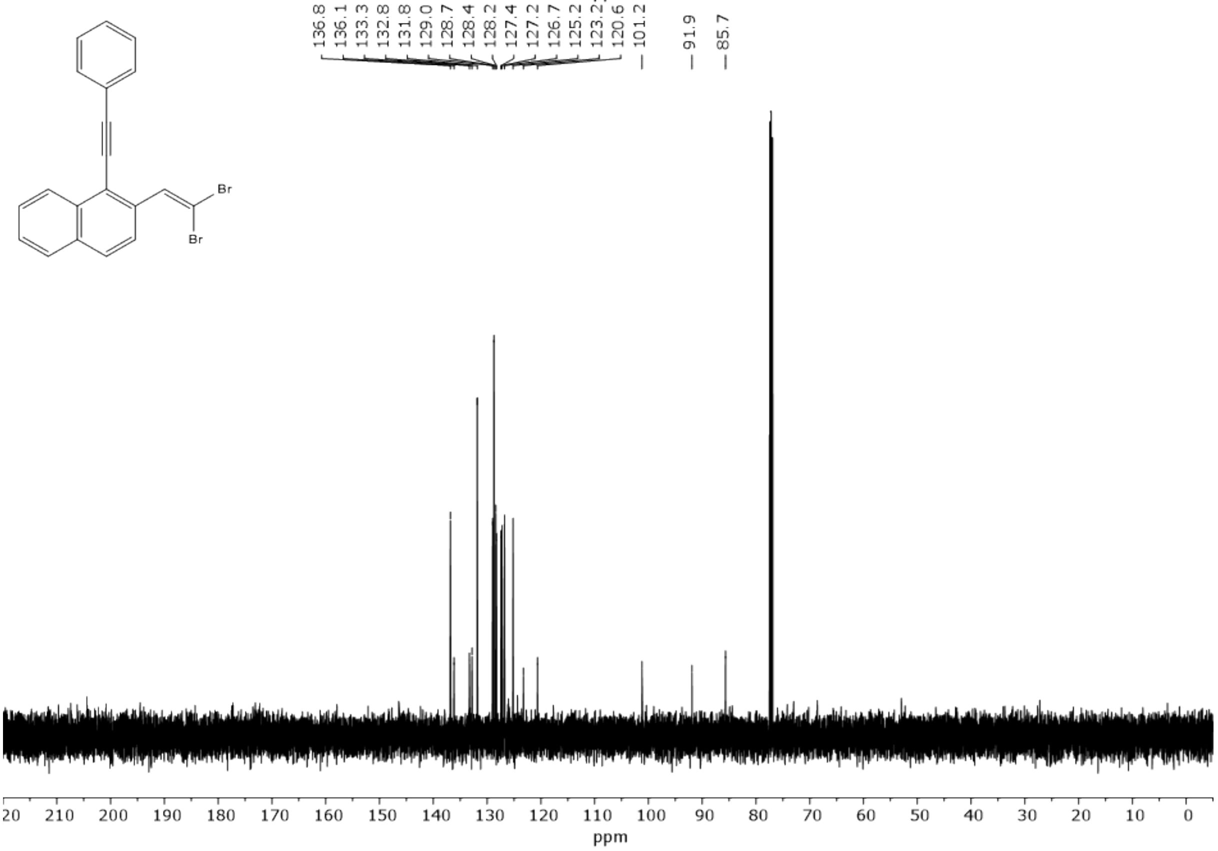

Figure S33. ^{1}H NMR spectrum (top) and $^{13}C\{^1H\}$ NMR spectrum (bottom) of **S27** ($CDCl_3$, 500 MHz).

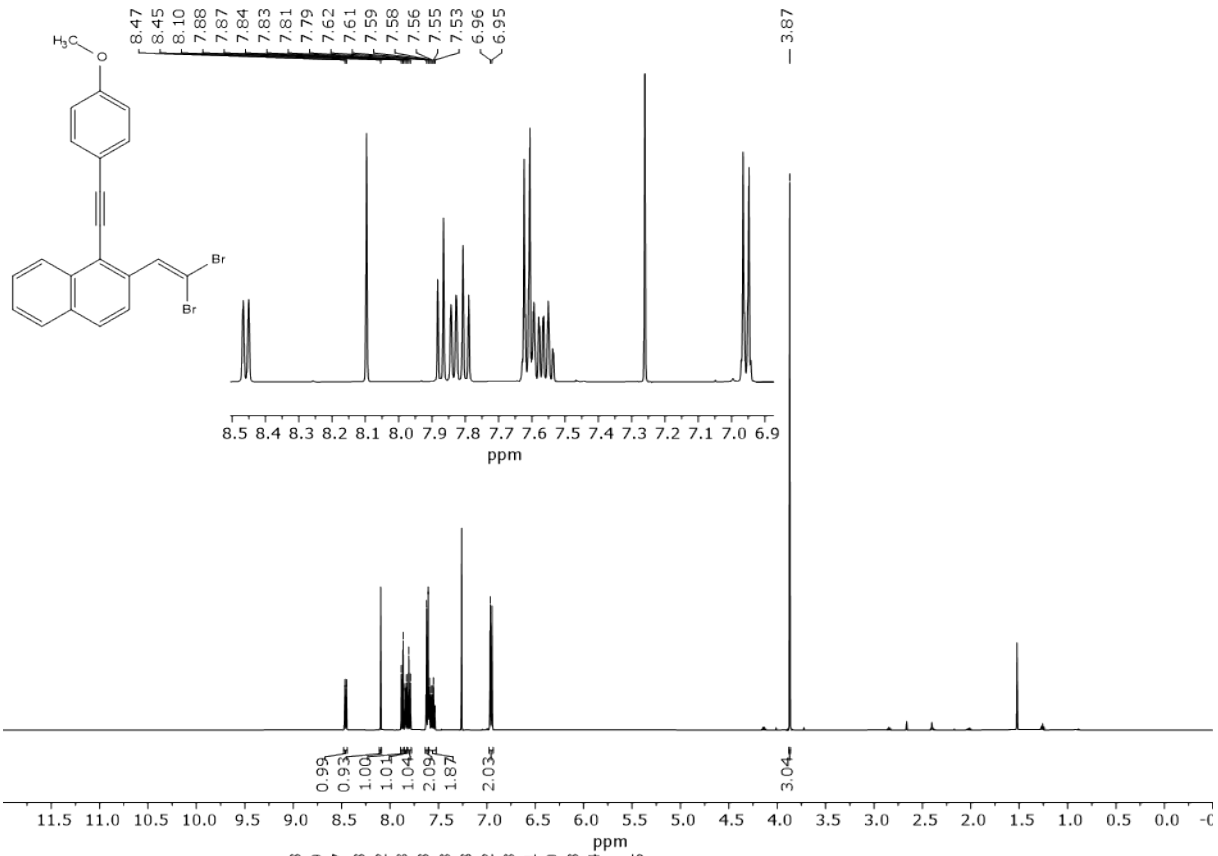

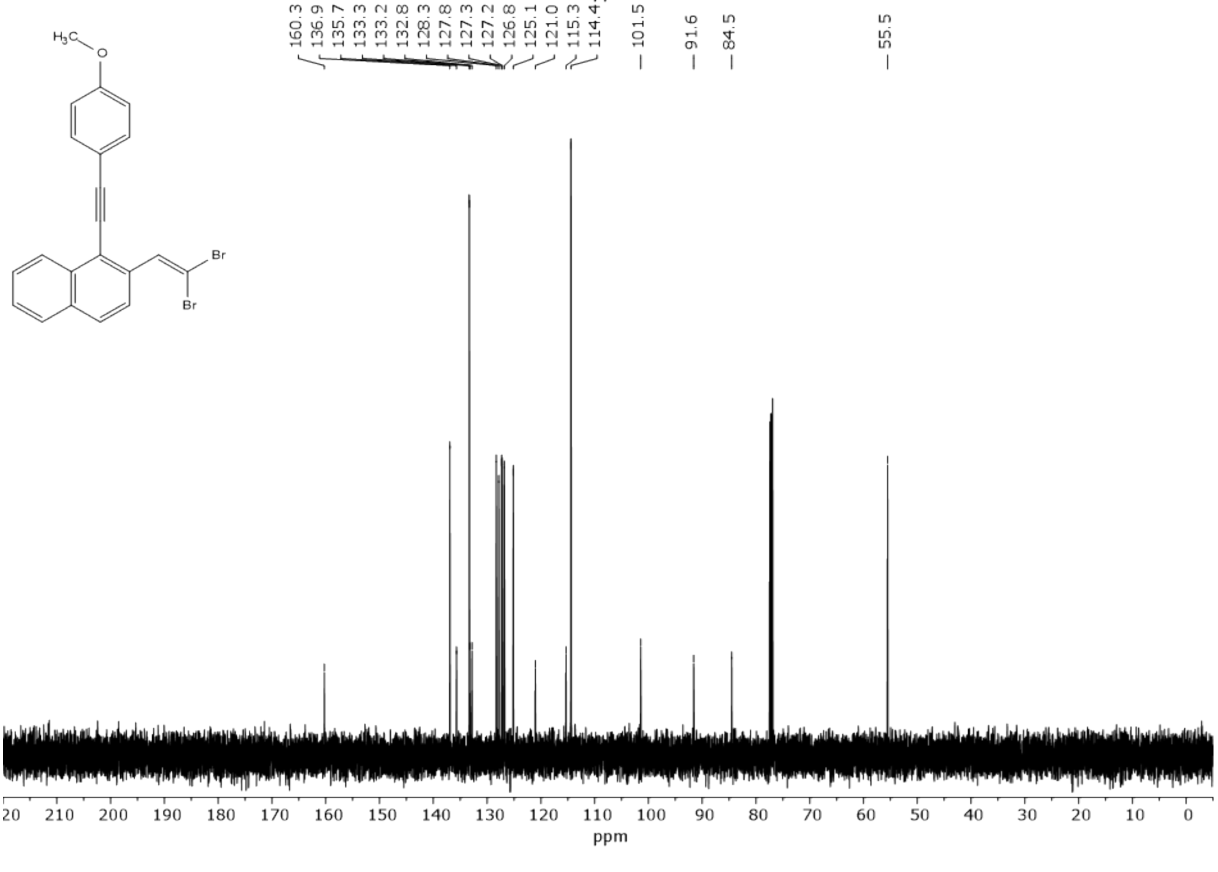

Figure S34. ^{1}H NMR spectrum (top) and $^{13}C\{^{1}H\}$ NMR spectrum (bottom) of **S28** ($CDCl_3$, 500 MHz).

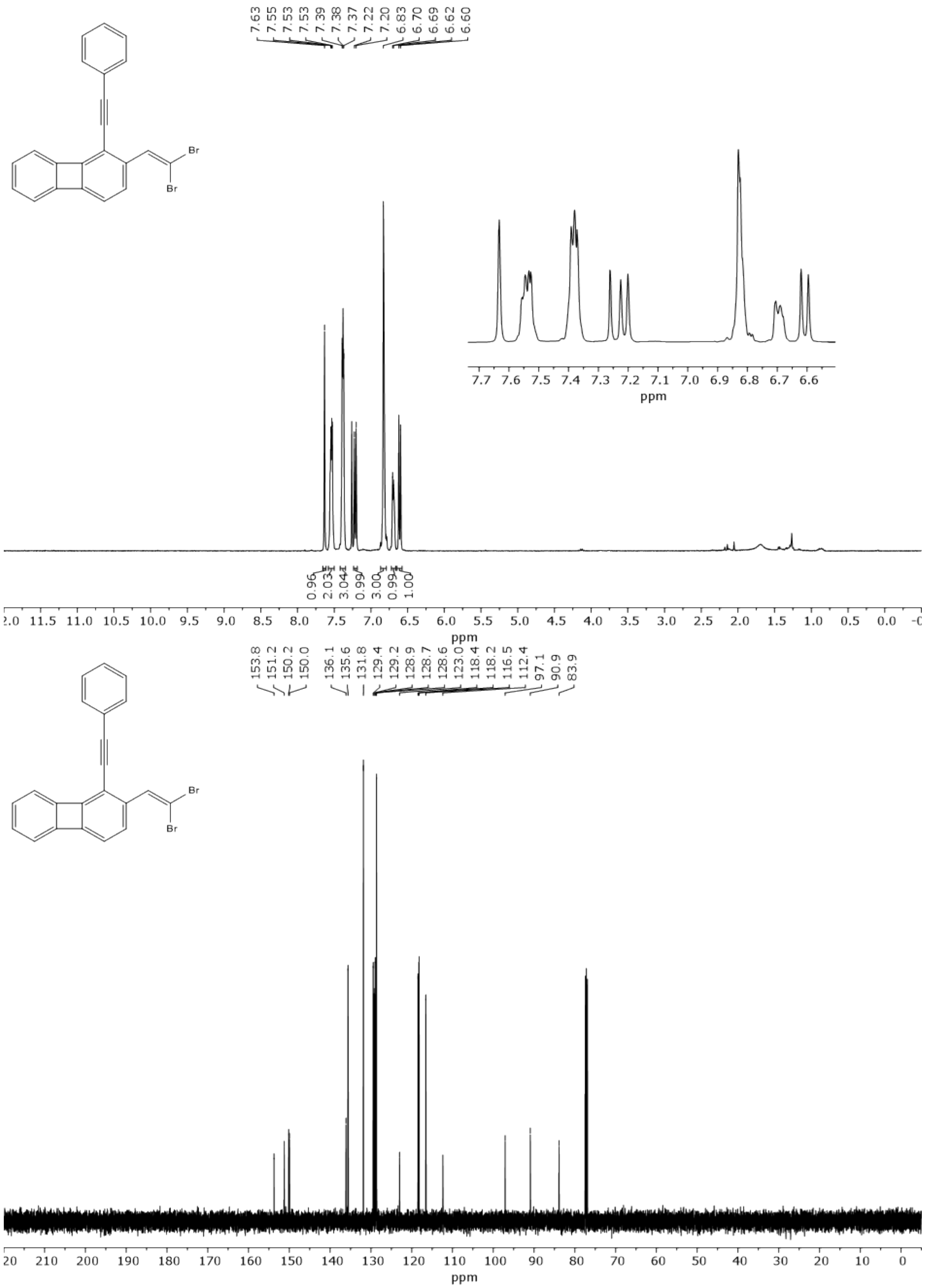

Figure S35. ^{1}H NMR spectrum (top) and ^{13}C{^{1}H} NMR spectrum (bottom) of **S29** ($CDCl_3$, 300 MHz).

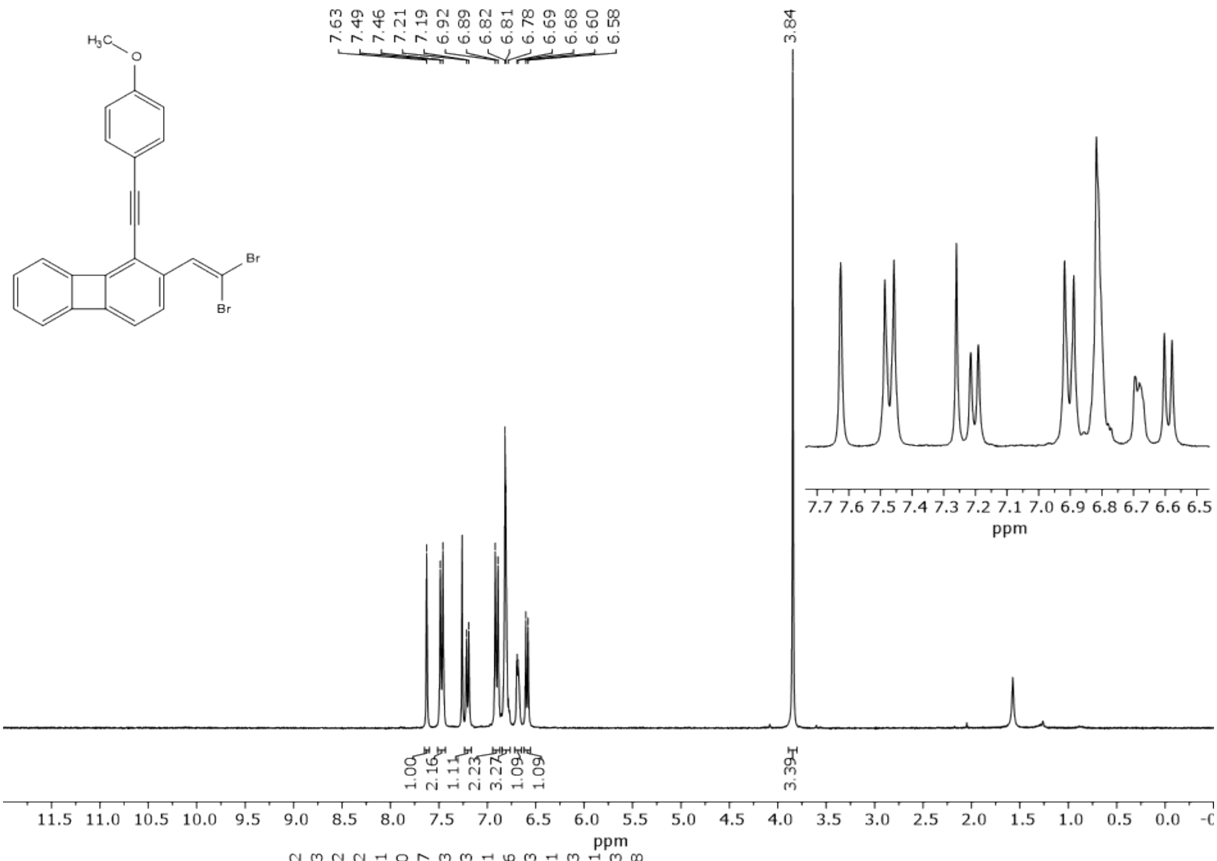

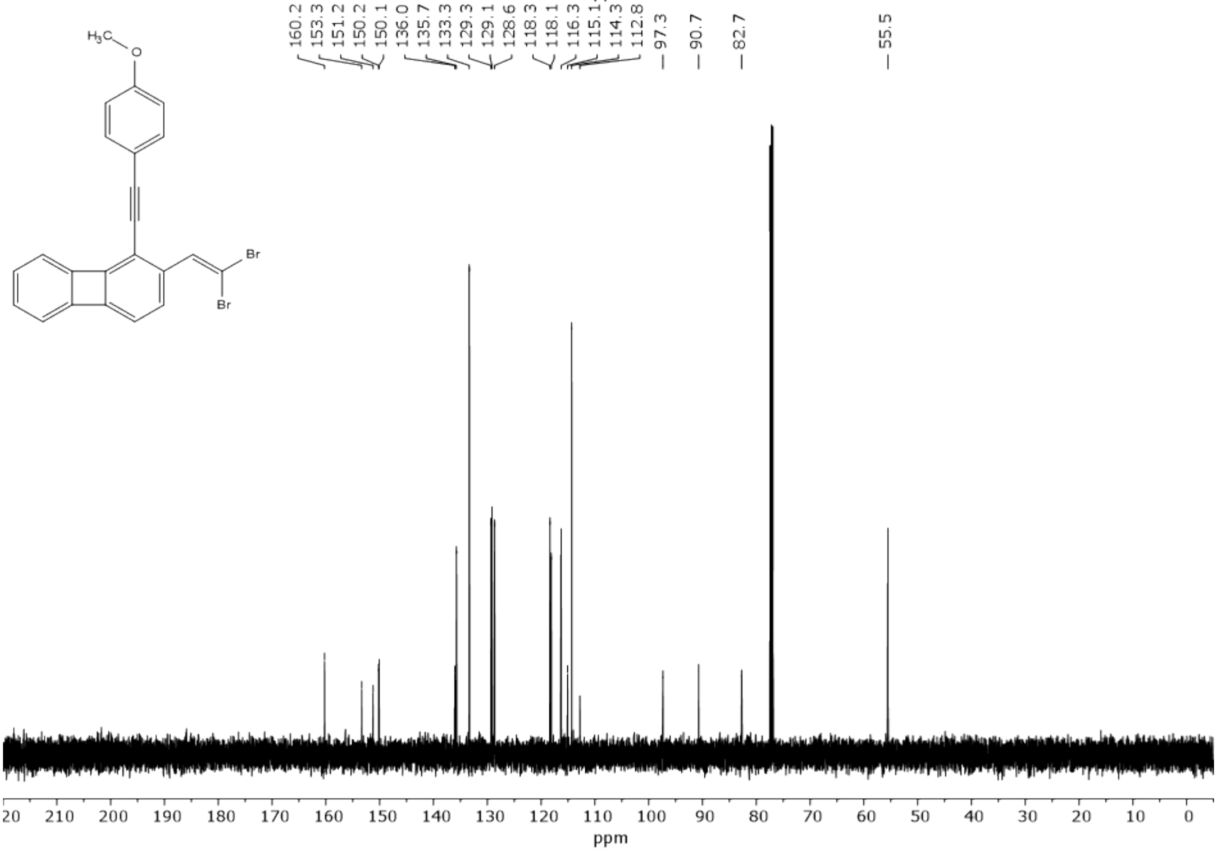

Figure S36. ^{1}H NMR spectrum (top) and $^{13}C\{^1H\}$ NMR spectrum (bottom) of **S30** ($CDCl_3$, 300 MHz).

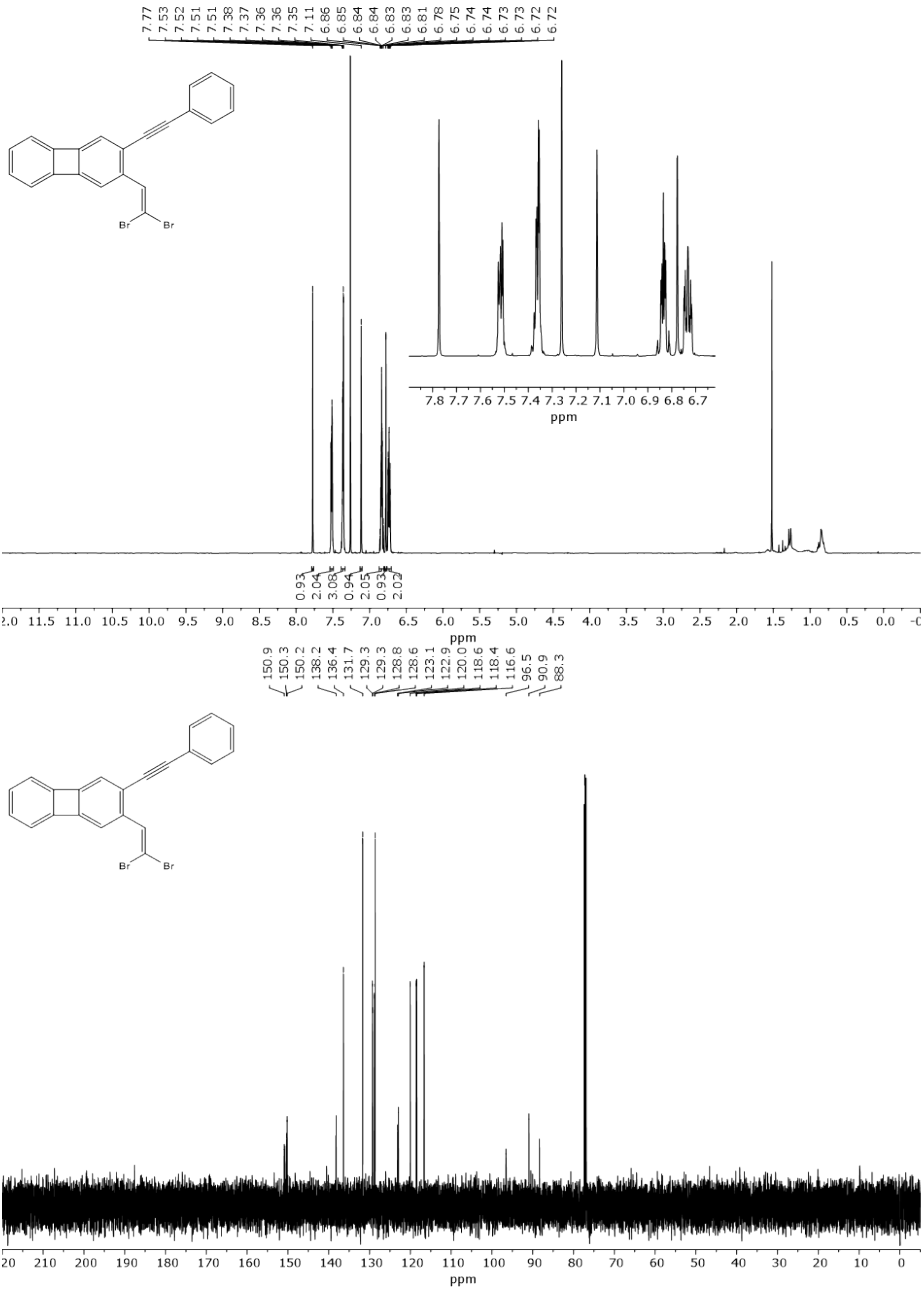

Figure S37. ^{1}H NMR spectrum (top) and $^{13}C\{^{1}H\}$ NMR spectrum (bottom) of **S31** ($CDCl_3$, 500 MHz).

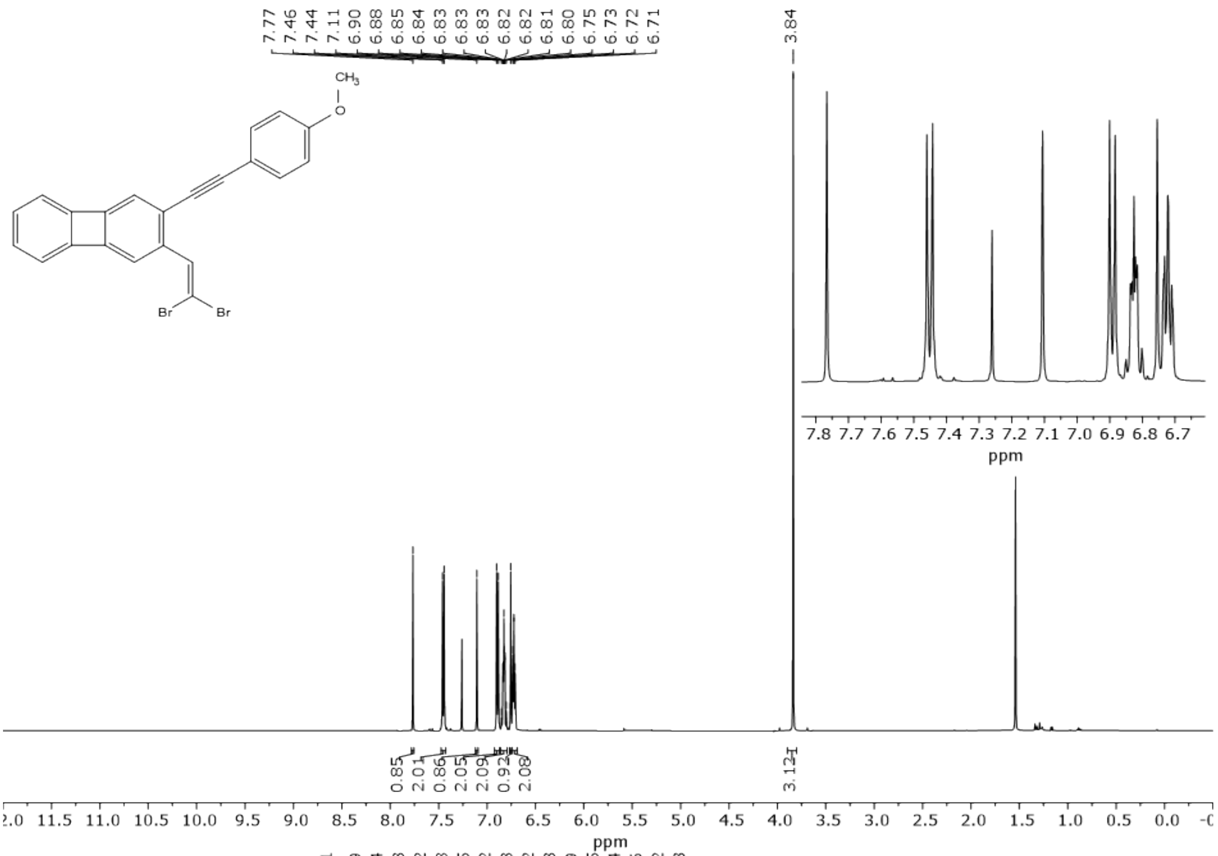

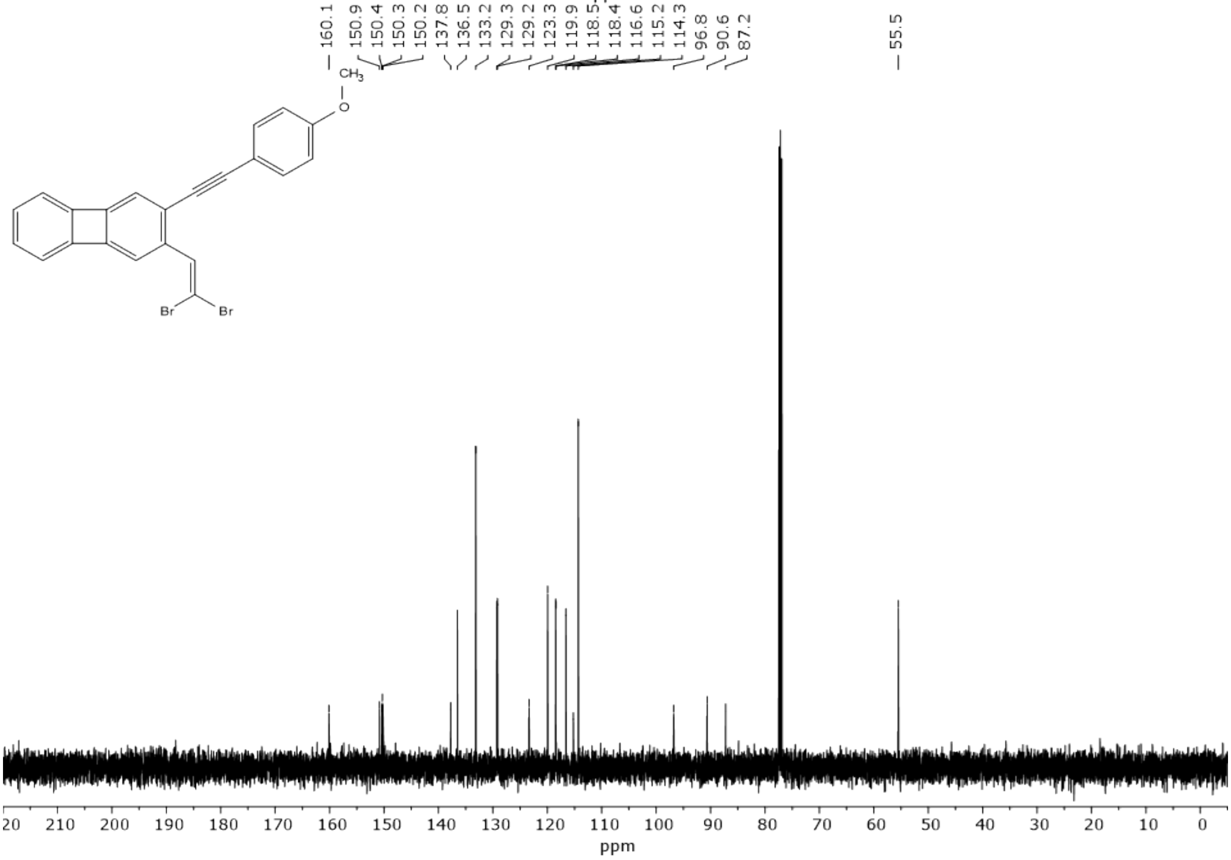

Figure S38. ^{1}H NMR spectrum (top) and $^{13}C\{^1H\}$ NMR spectrum (bottom) of **S32** ($CDCl_3$, 500 MHz).

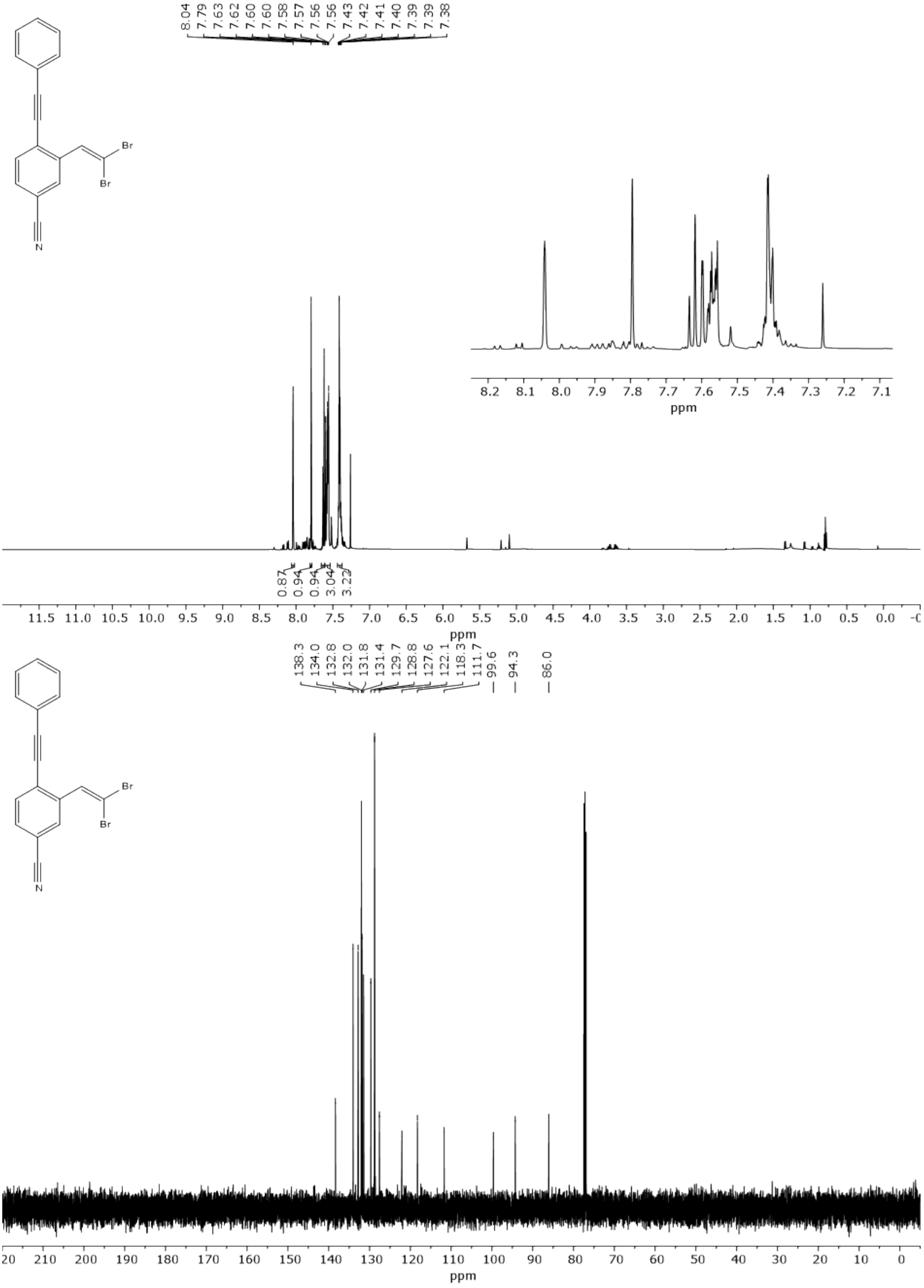

Figure S39. ^{1}H NMR spectrum (top) and ^{13}C{^{1}H} NMR spectrum (bottom) of **S33** ($CDCl_3$, 500 MHz).

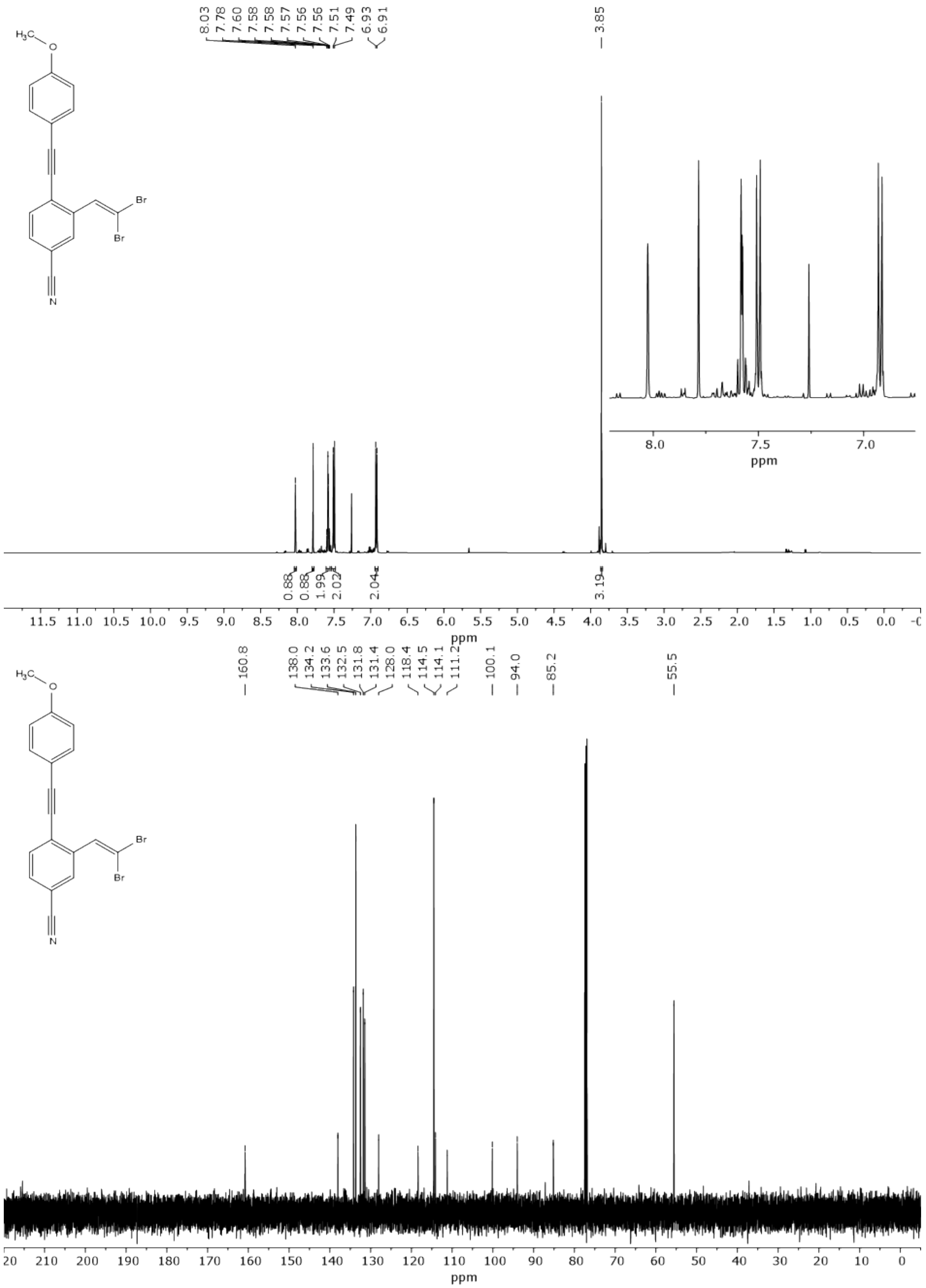

Figure S40. ^{1}H NMR spectrum (top) and $^{13}C\{^{1}H\}$ NMR spectrum (bottom) of **S34** ($CDCl_3$, 500 MHz).

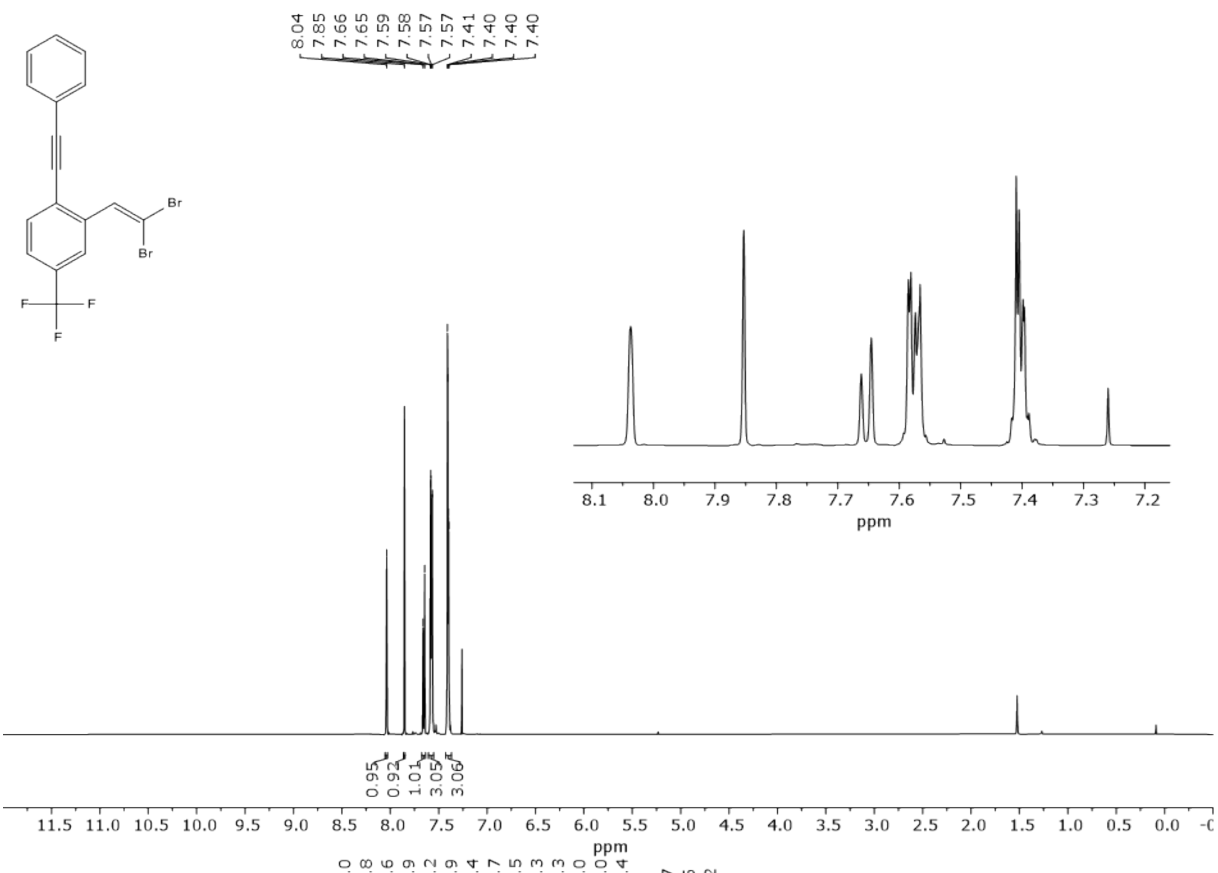

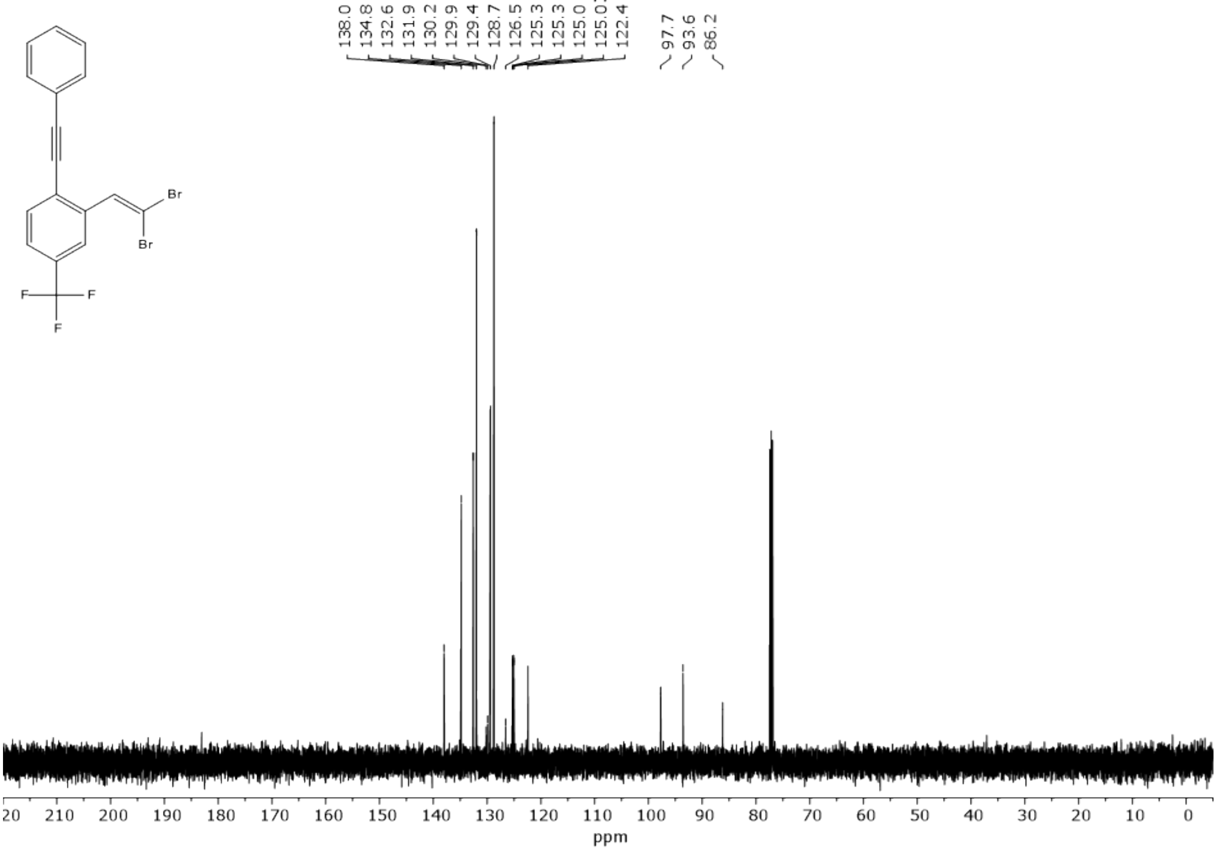

Figure S41. ^{1}H NMR spectrum (top) and $^{13}C\{^{1}H\}$ NMR spectrum (bottom) of **S35** ($CDCl_3$, 500 MHz).

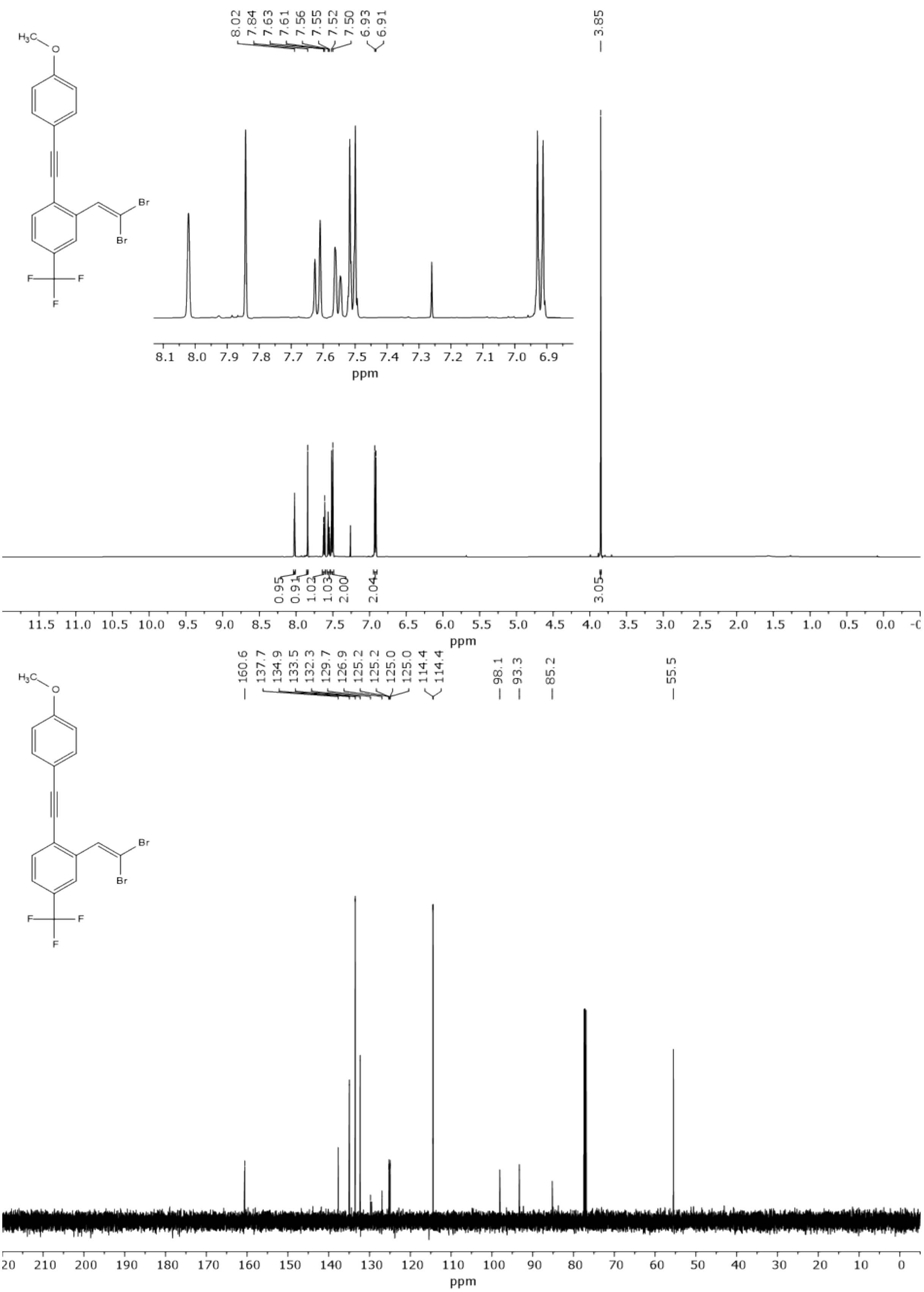

Figure S42. ^{1}H NMR spectrum (top) and $^{13}C\{^{1}H\}$ NMR spectrum (bottom) of **S36** ($CDCl_3$, 500 MHz).

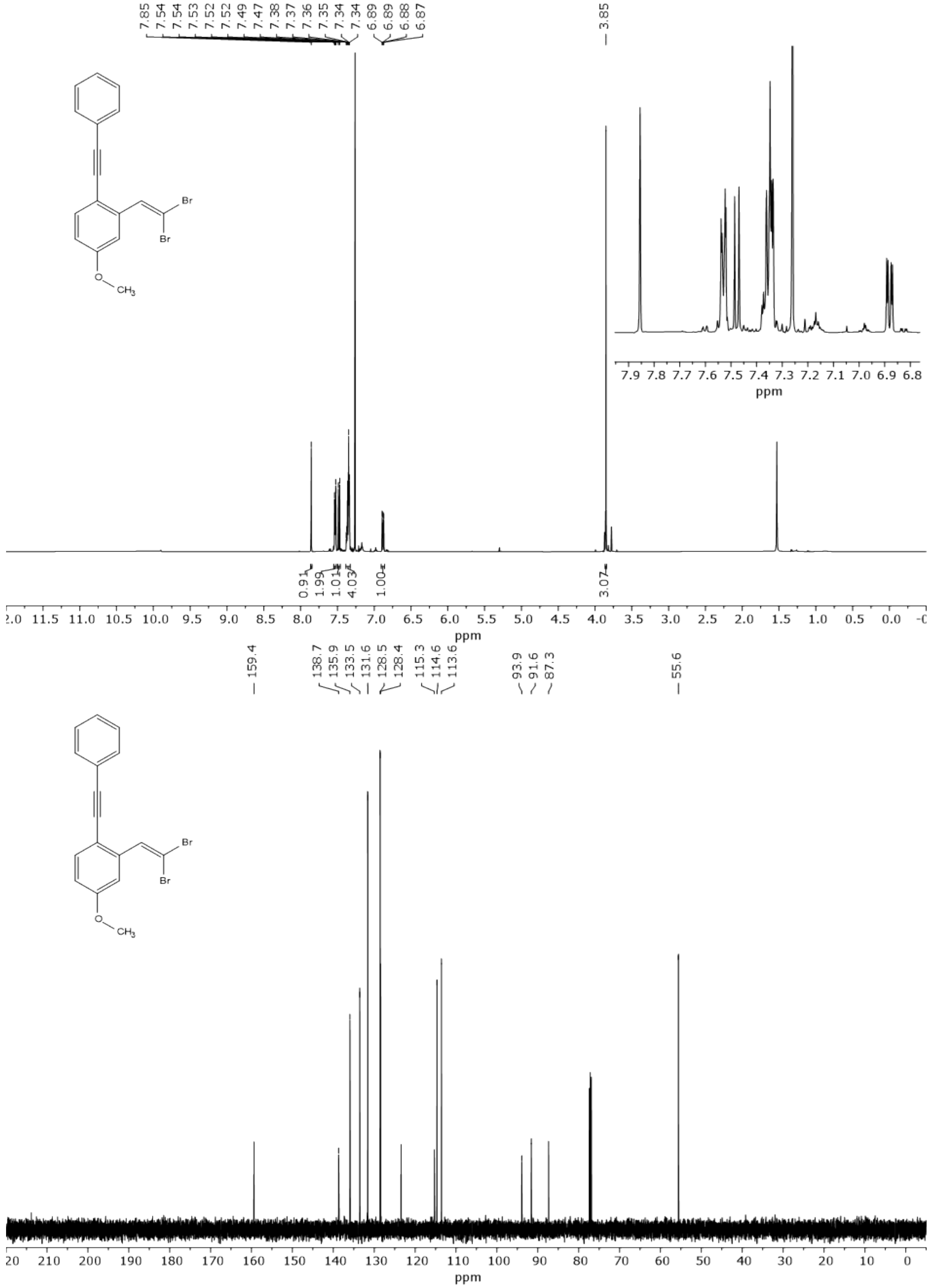

Figure S43. ^{1}H NMR spectrum (top) and $^{13}C\{^{1}H\}$ NMR spectrum (bottom) of **S37** ($CDCl_3$, 500 MHz).

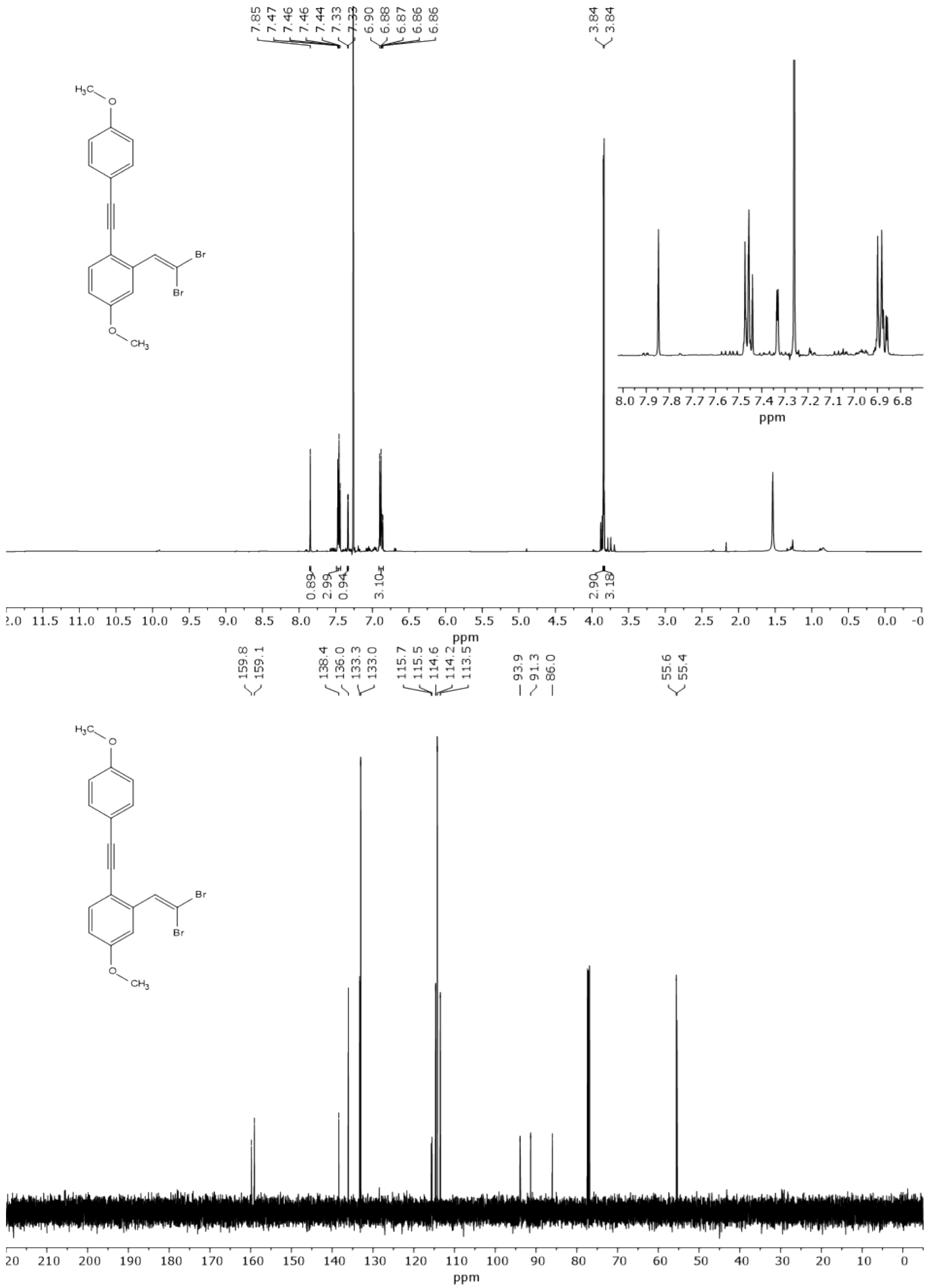

Figure S44. ^{1}H NMR spectrum (top) and $^{13}C\{^{1}H\}$ NMR spectrum (bottom) of **S38** ($CDCl_3$, 500 MHz).

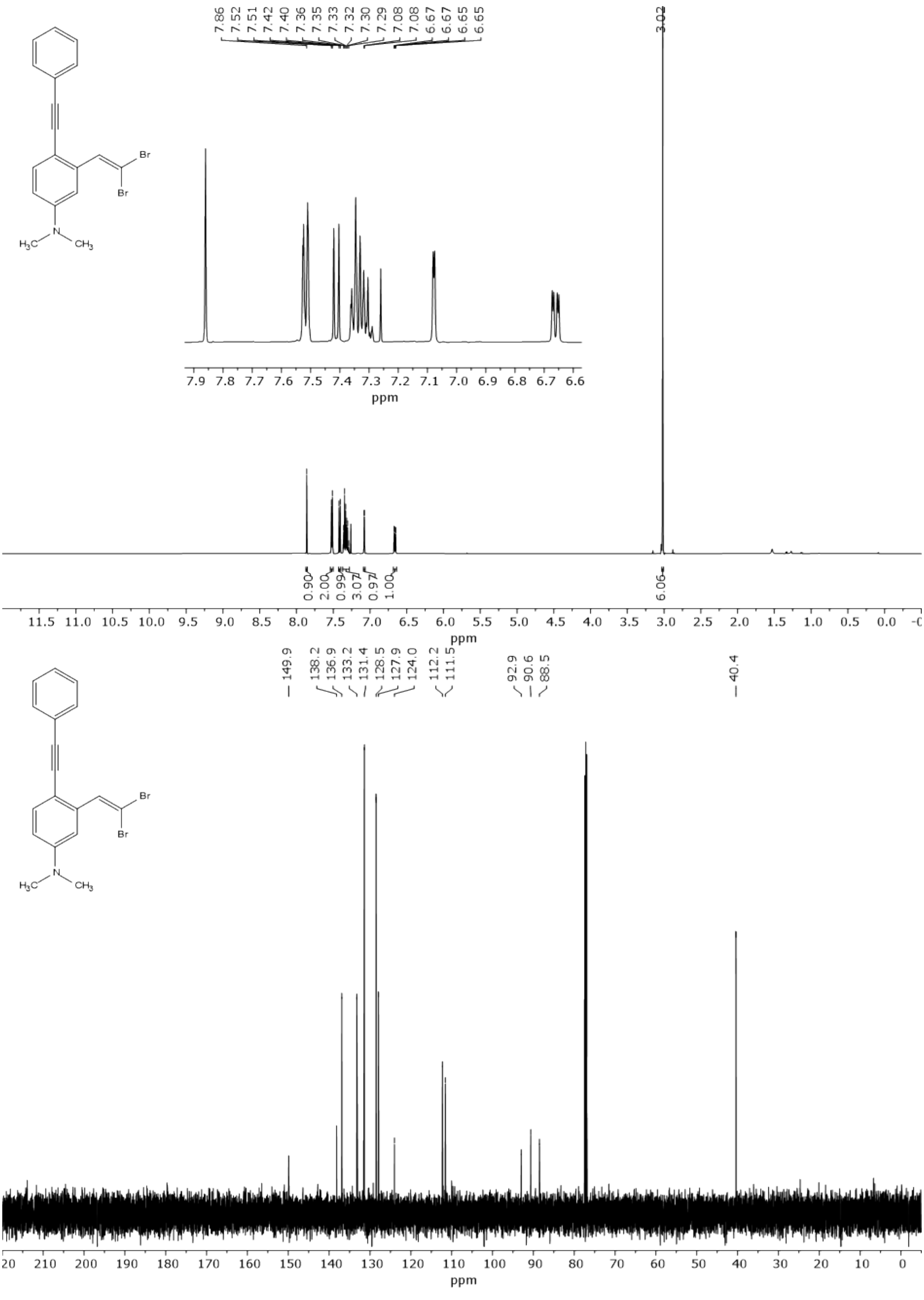

Figure S45. ^{1}H NMR spectrum (top) and $^{13}C\{^1H\}$ NMR spectrum (bottom) of **S39** ($CDCl_3$, 500 MHz).

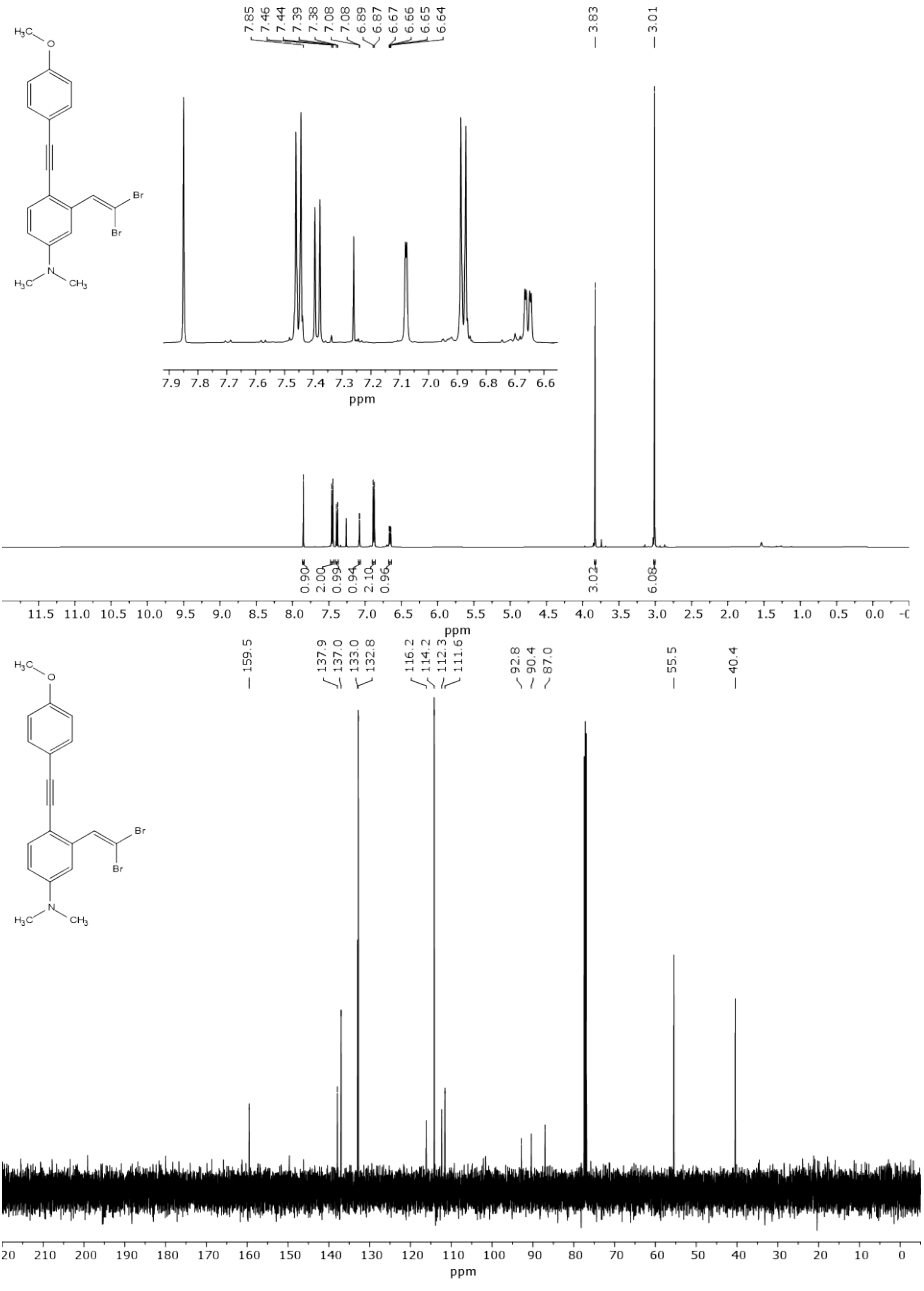

Figure S46. ^{1}H NMR spectrum (top) and $^{13}C\{^{1}H\}$ NMR spectrum (bottom) of **S40** ($CDCl_3$, 500 MHz).

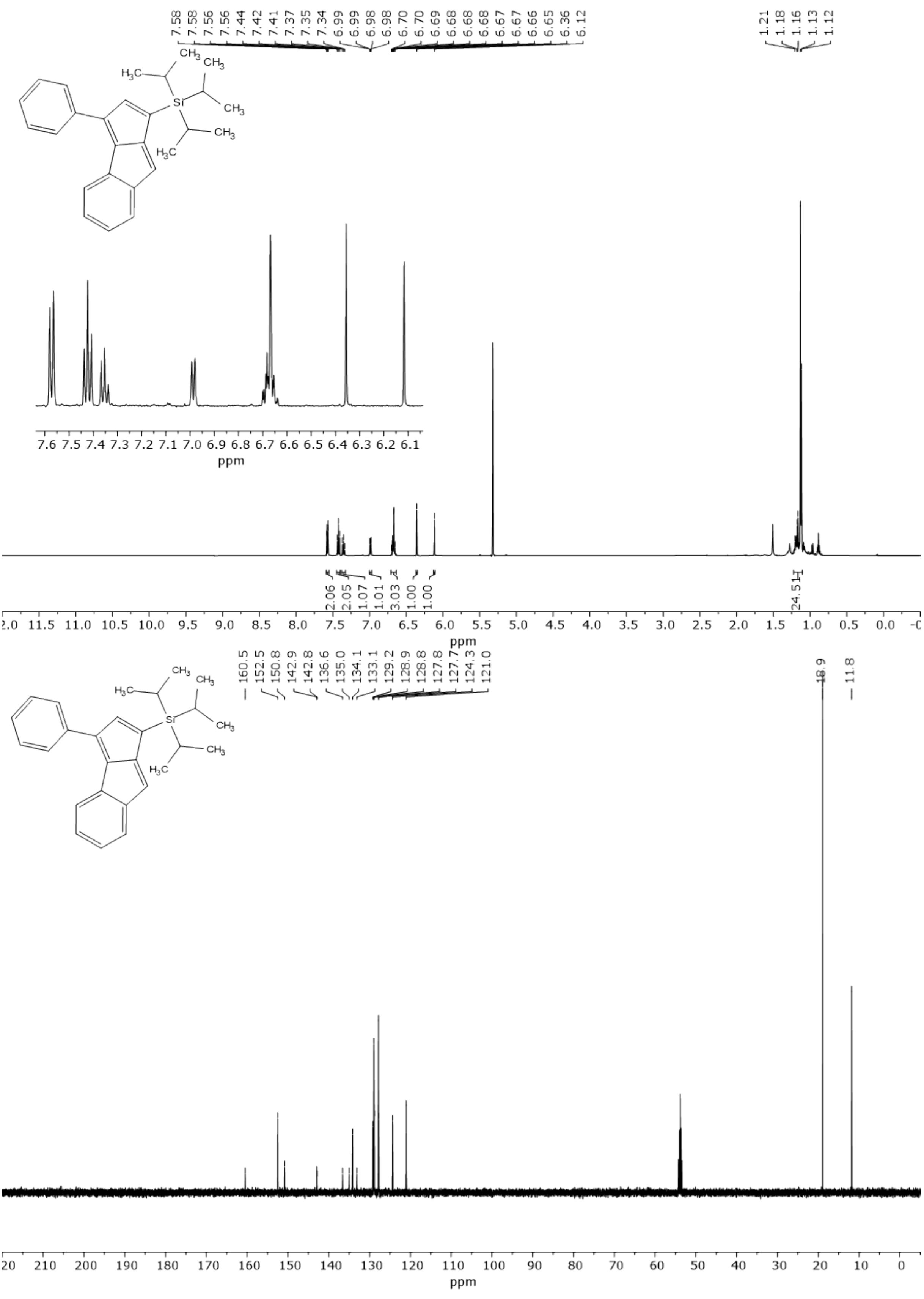

Figure S47. ^{1}H NMR spectrum (top) and $^{13}C\{^{1}H\}$ NMR spectrum (bottom) of **1** (CD_2Cl_2, 500 MHz).

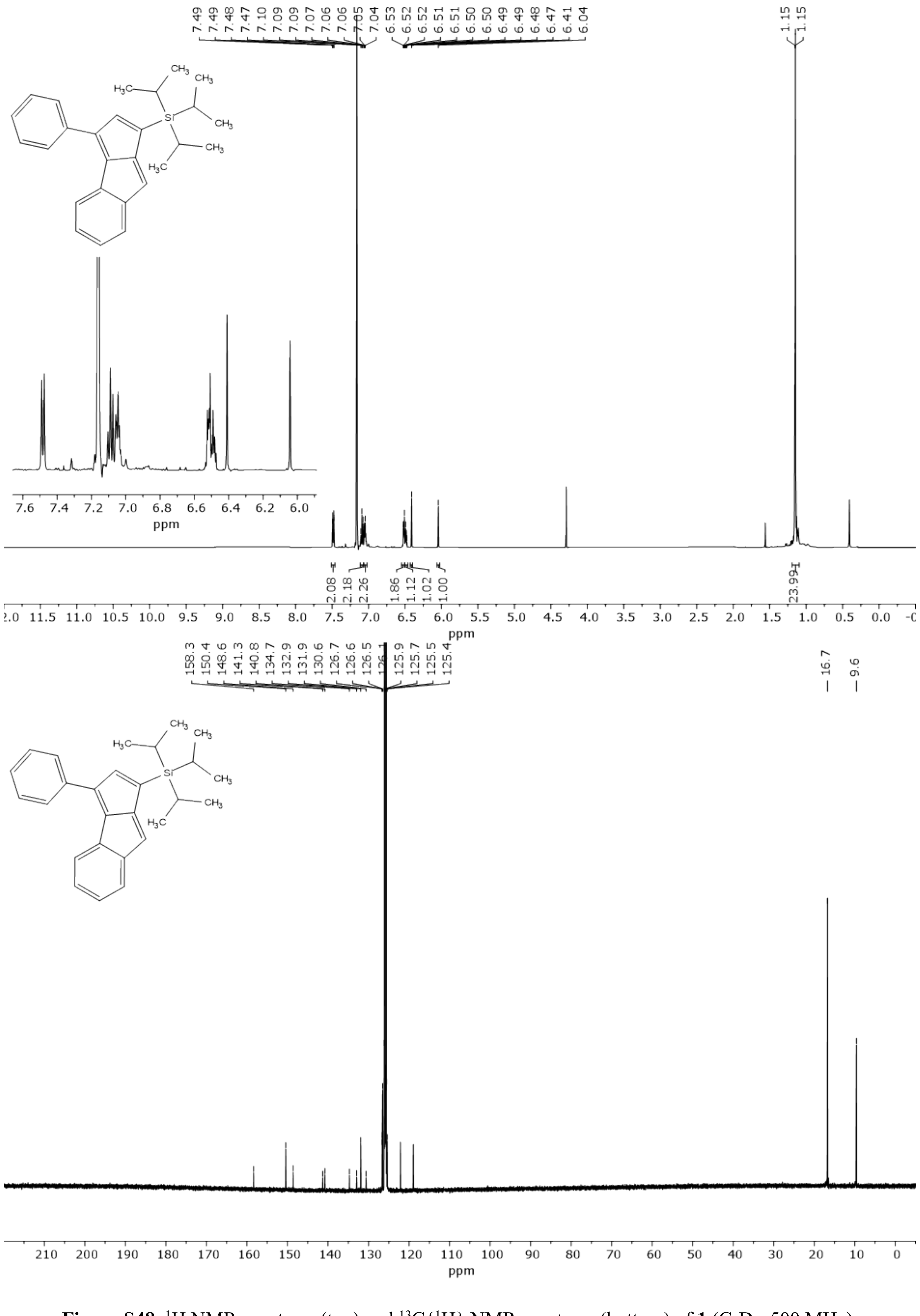

Figure S48. ^{1}H NMR spectrum (top) and $^{13}C\{^{1}H\}$ NMR spectrum (bottom) of **1** (C_6D_6, 500 MHz).

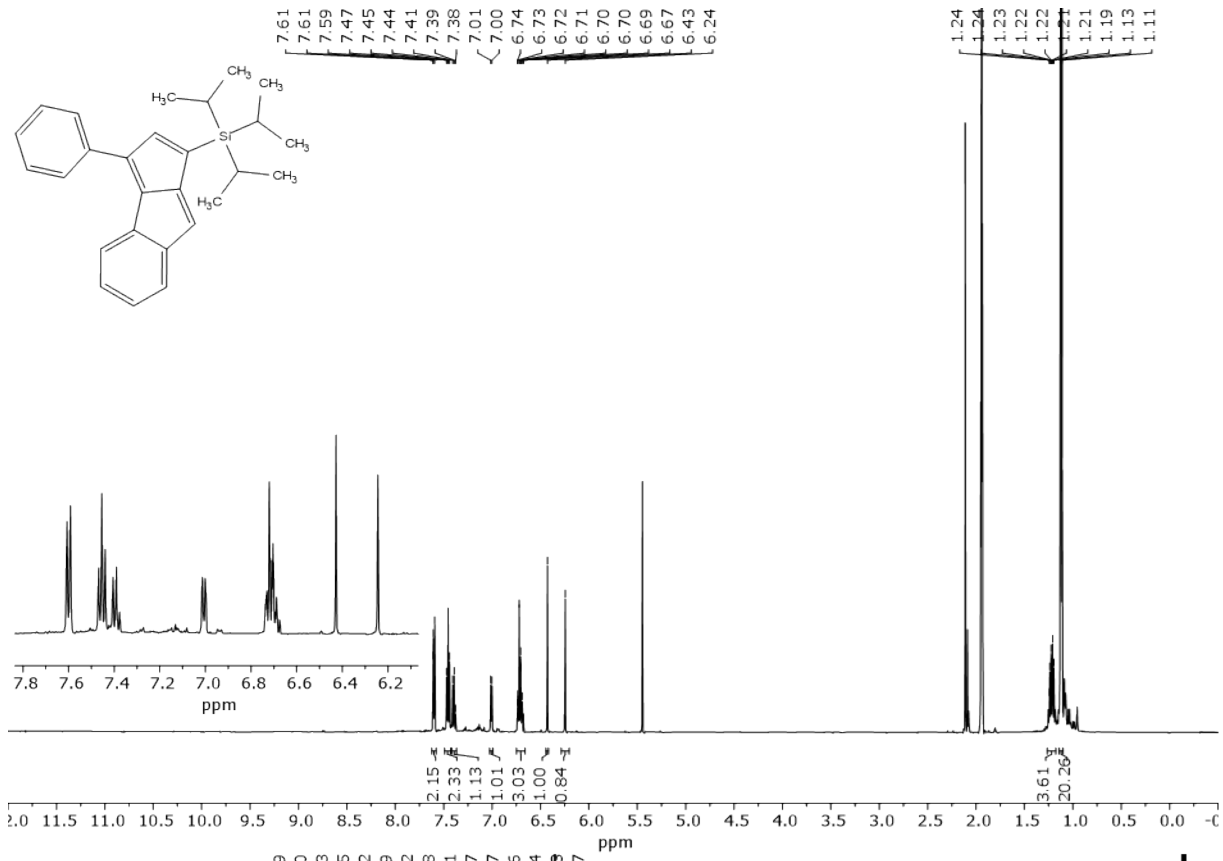

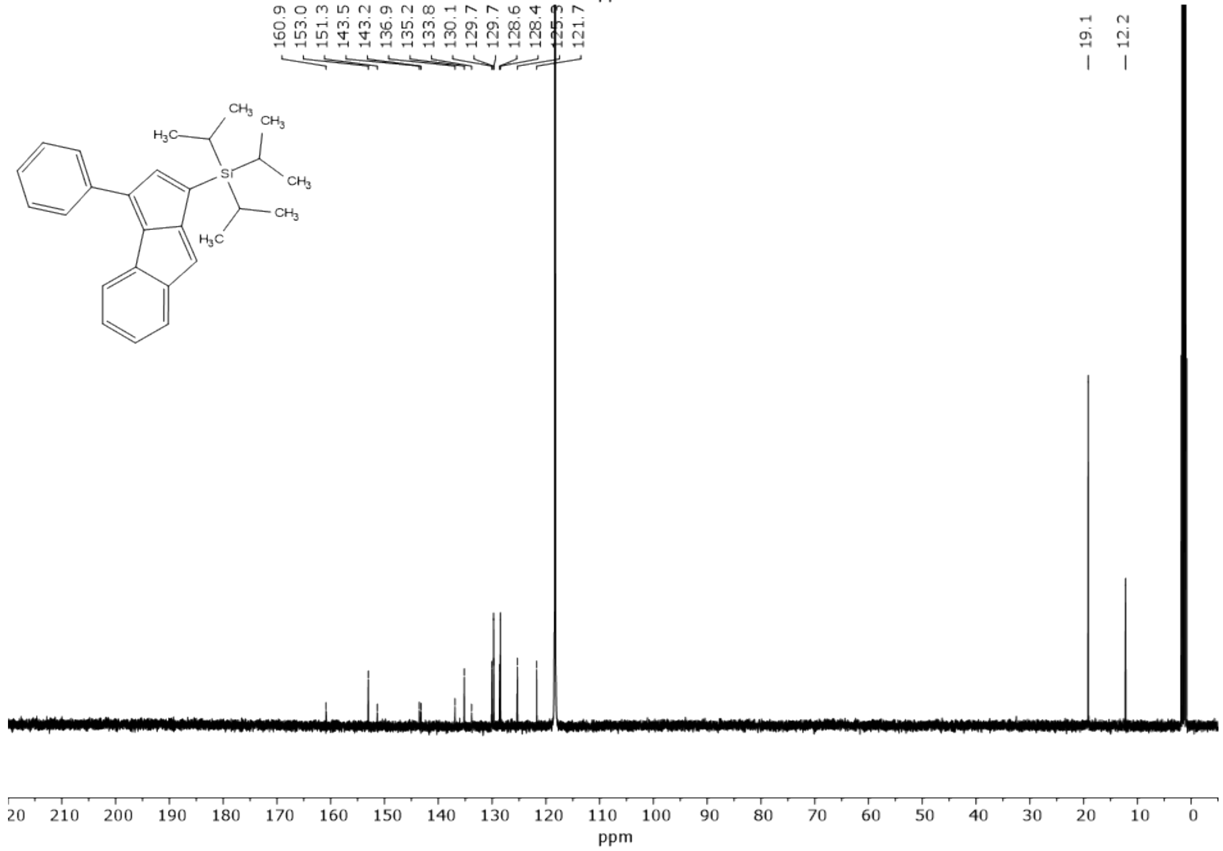

Figure S49. ^{1}H NMR spectrum (top) and $^{13}C\{^{1}H\}$ NMR spectrum (bottom) of **1** (CD_3CN, 500 MHz).

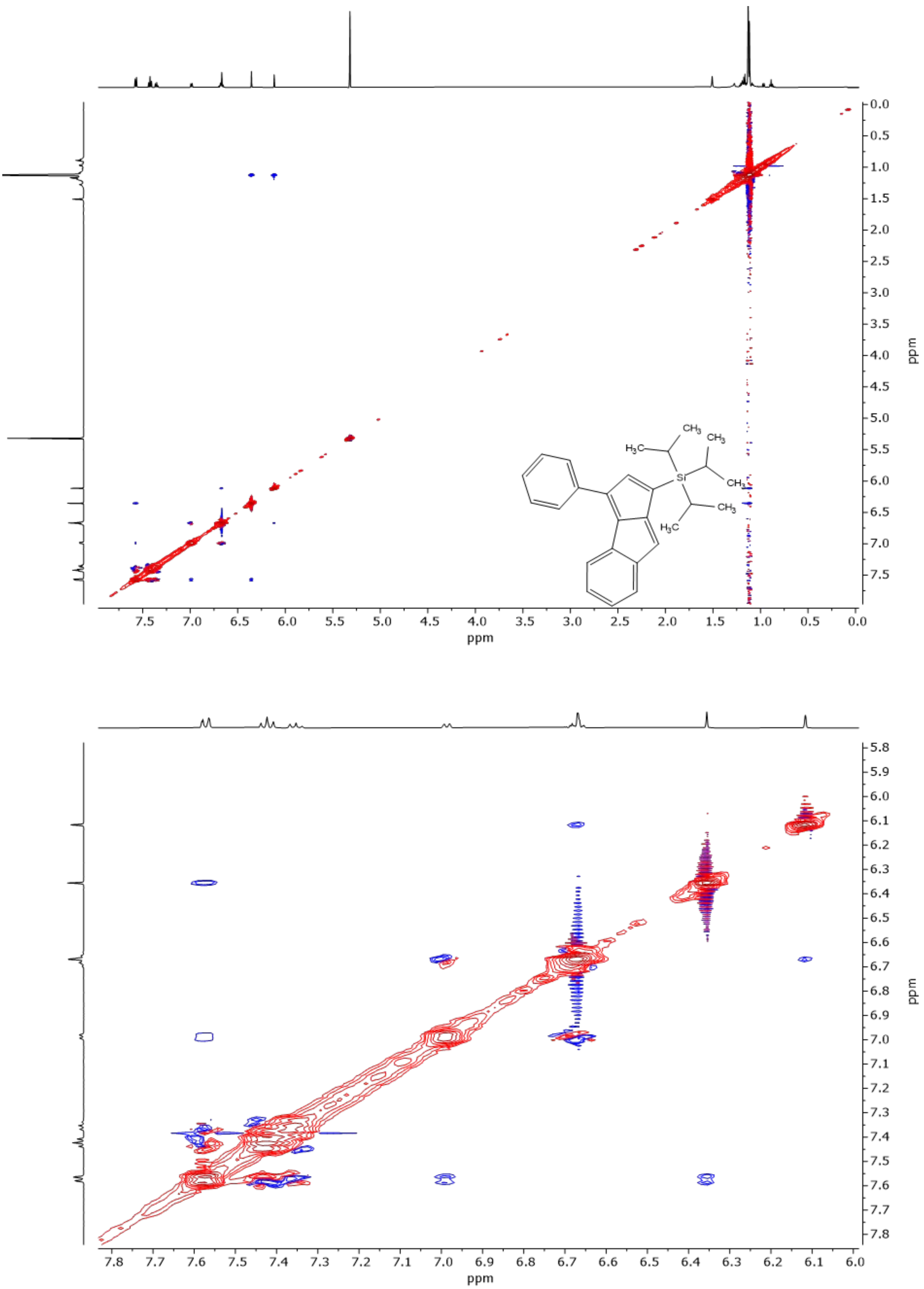

Figure S50. NOESY spectrum of **1** (CD_2Cl_2, 300 MHz, top-full, bottom-zoomed in on aromatic region).

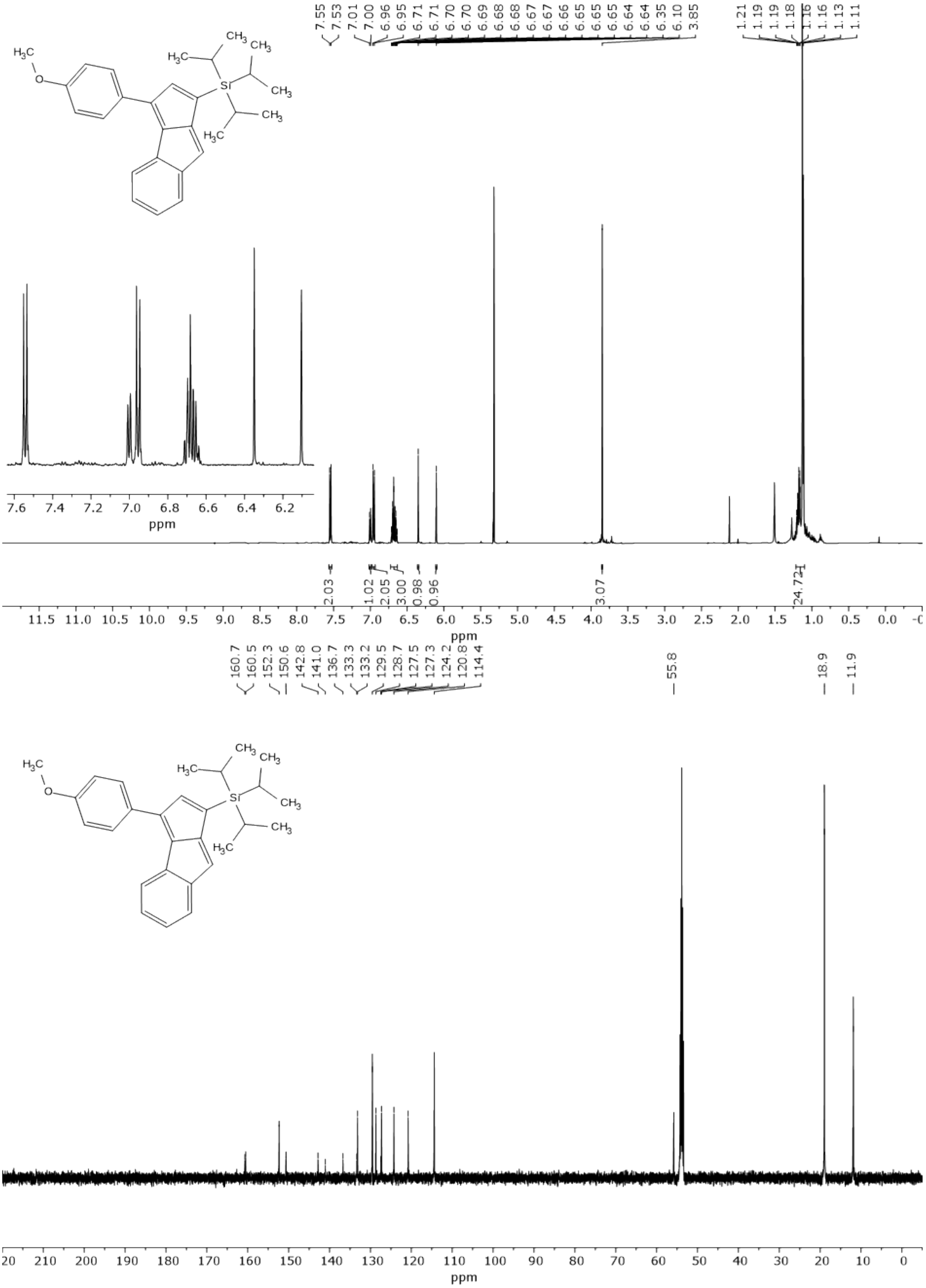

Figure S51. ^{1}H NMR spectrum (top) and $^{13}C\{^{1}H\}$ NMR spectrum (bottom) of **1'** (CD_2Cl_2, 500 MHz).

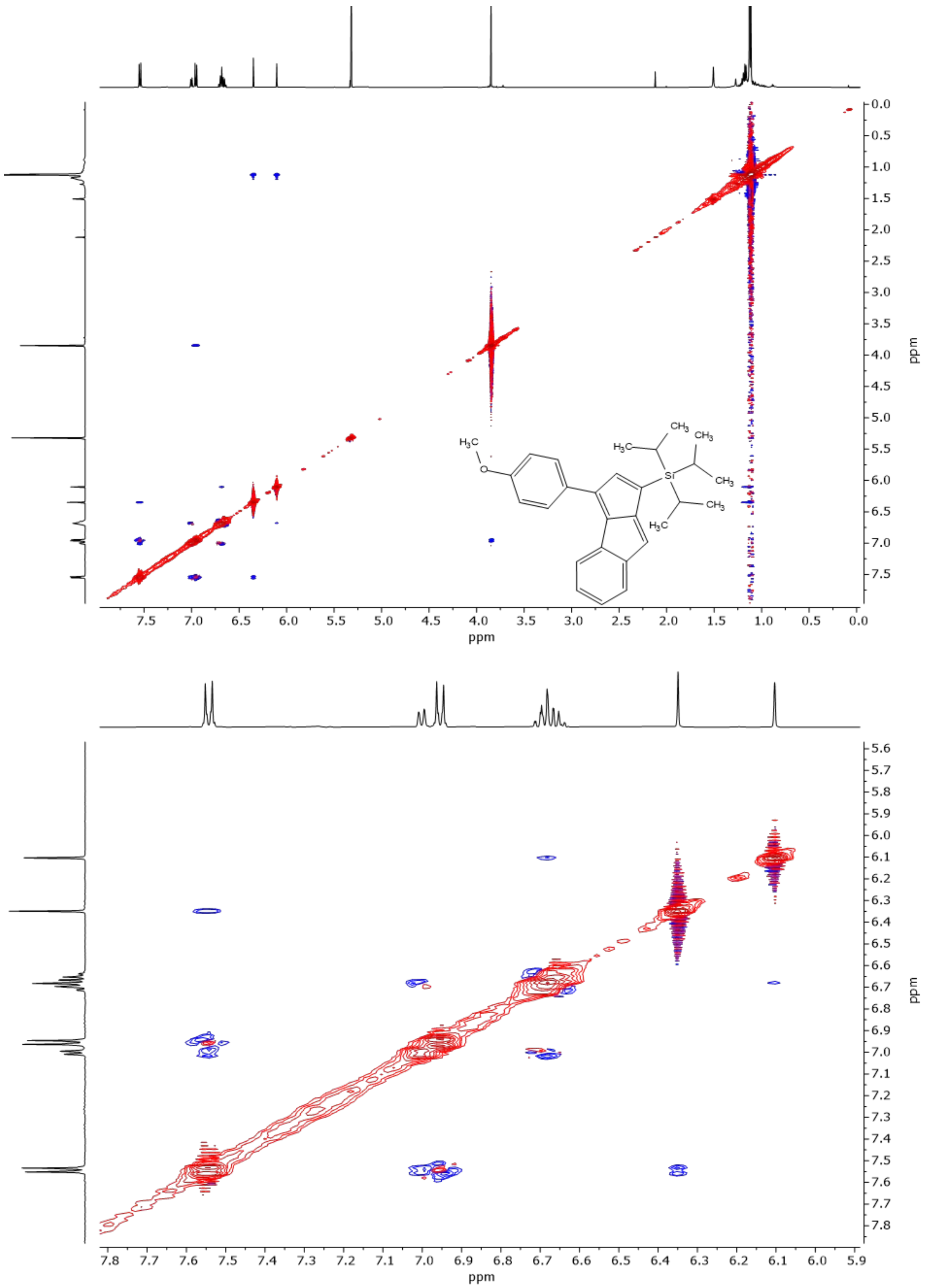

Figure S52. NOESY spectrum of **1’** (CD_2Cl_2, 300 MHz, top-full, bottom-zoomed in on aromatic region).

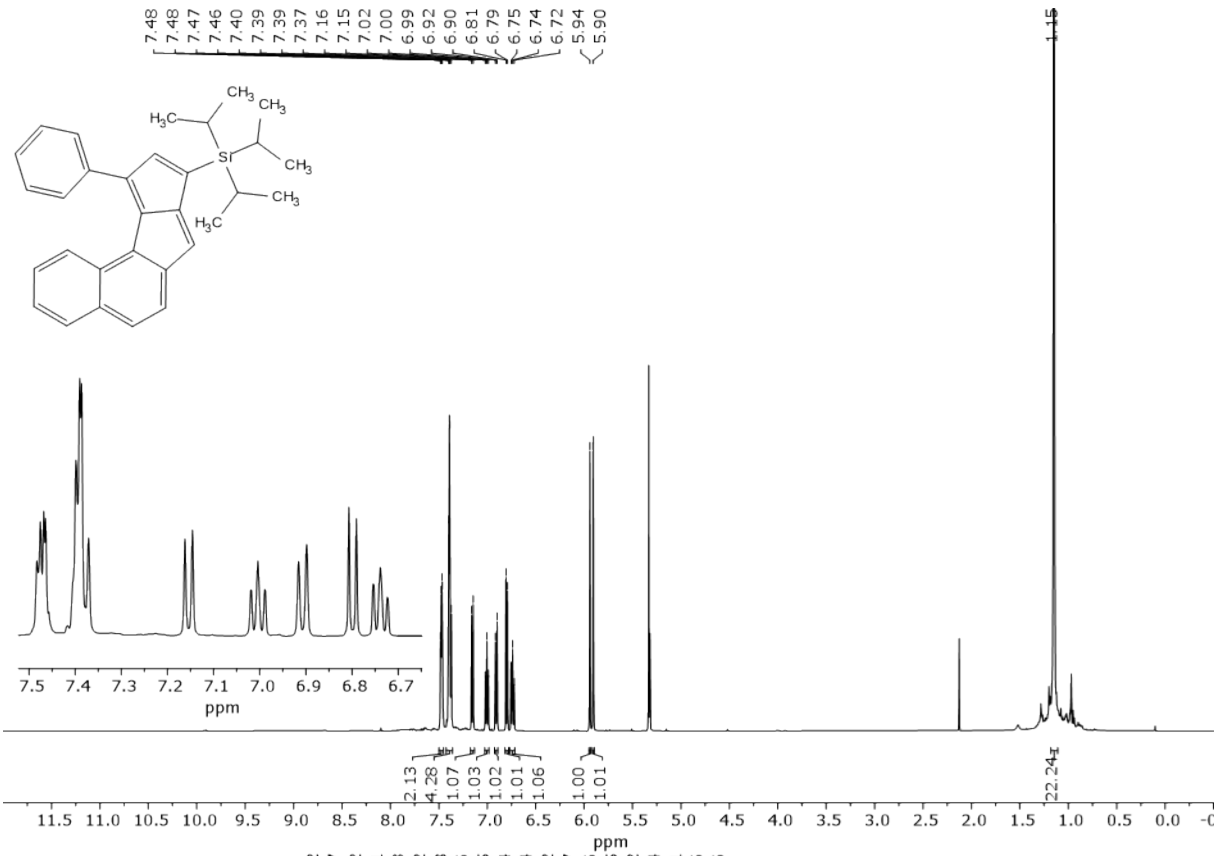

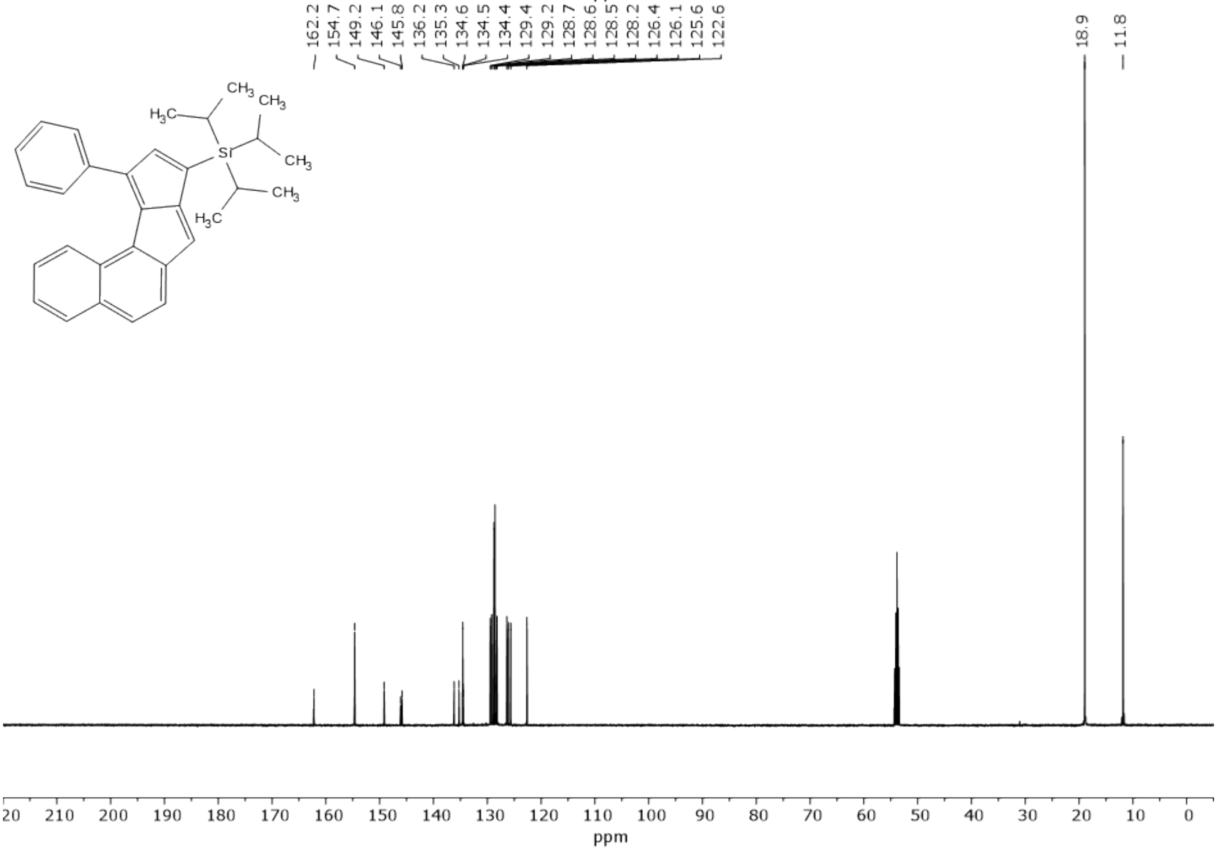

Figure S53. ^{1}H NMR spectrum (top) and $^{13}C\{^{1}H\}$ NMR spectrum (bottom) of **2** (CD_2Cl_2, 500 MHz).

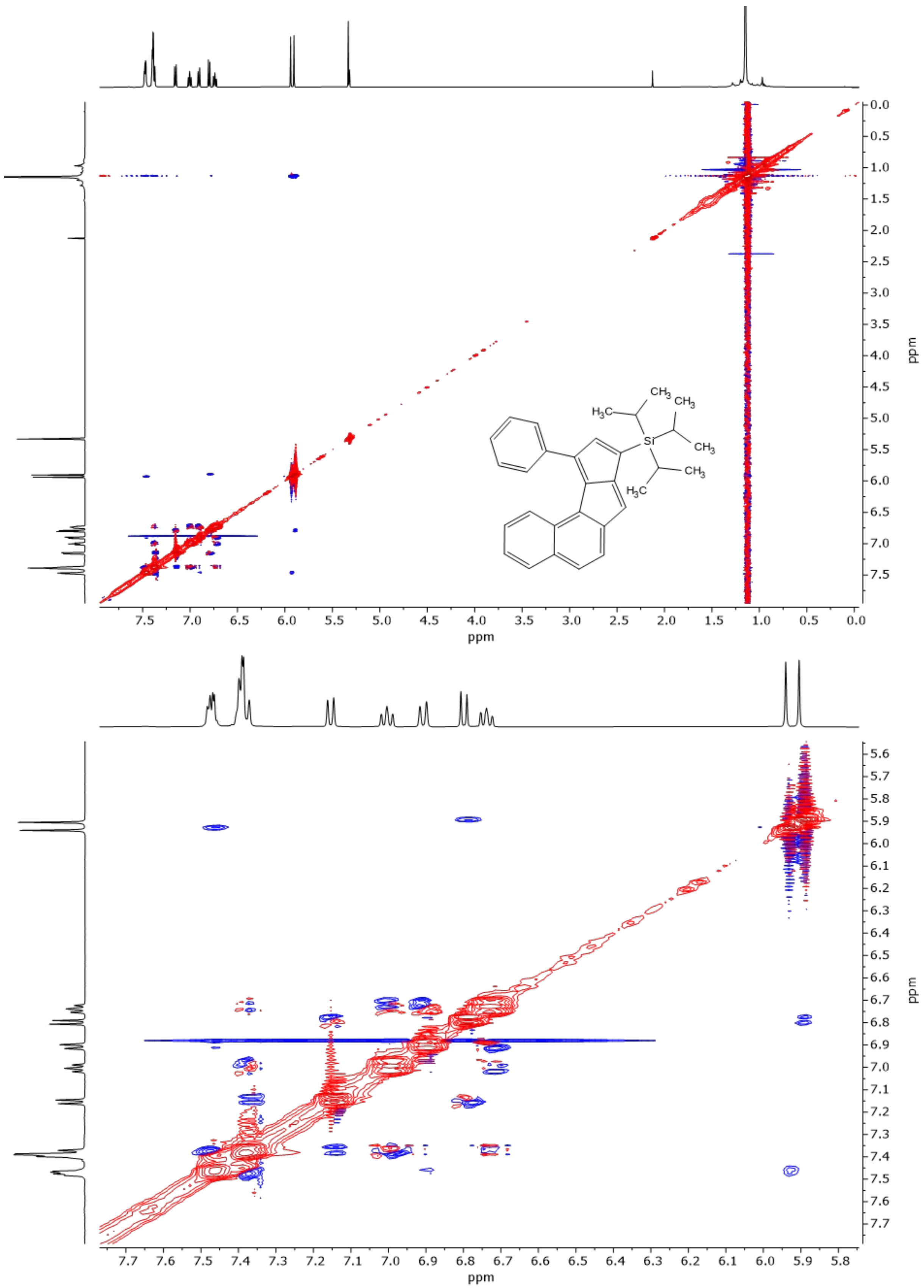

Figure S54. NOESY spectrum of **2** (CD_2Cl_2, 300 MHz, top-full, bottom-zoomed in on aromatic region).

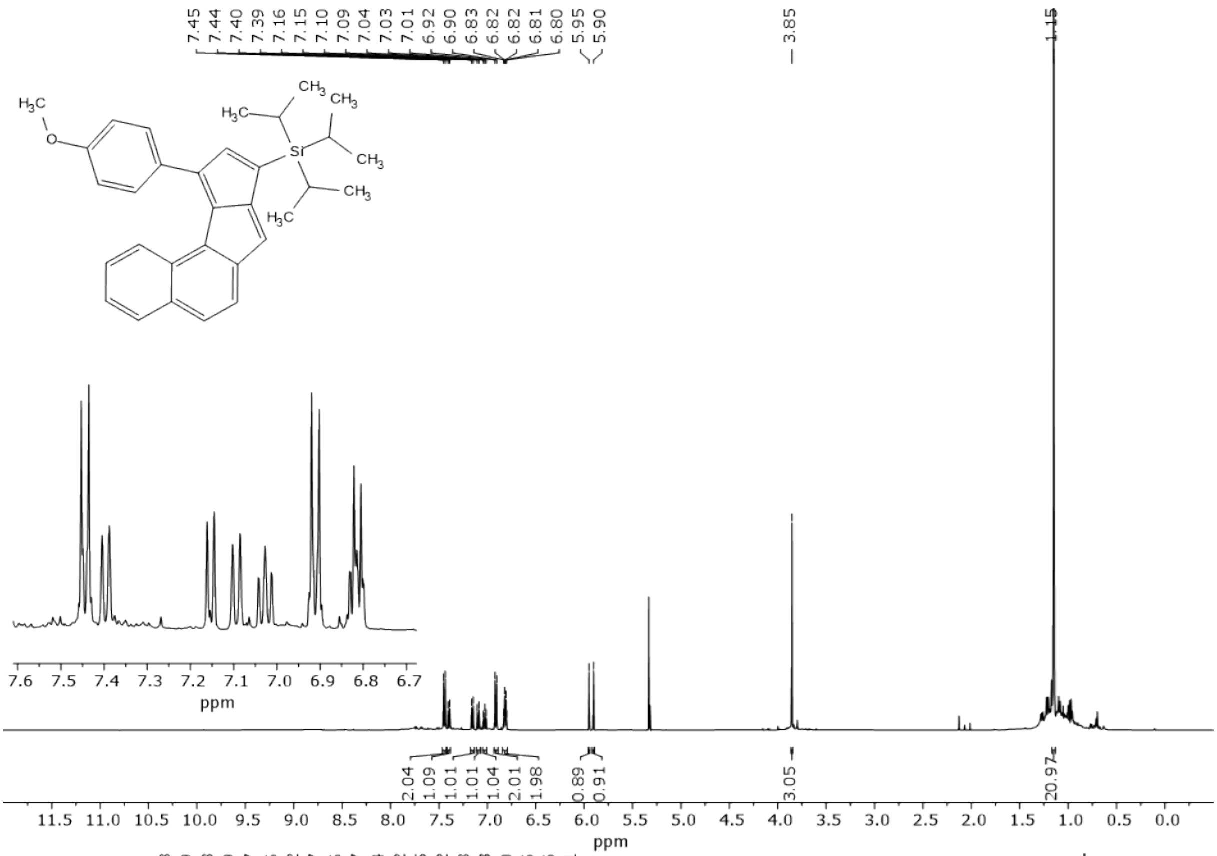

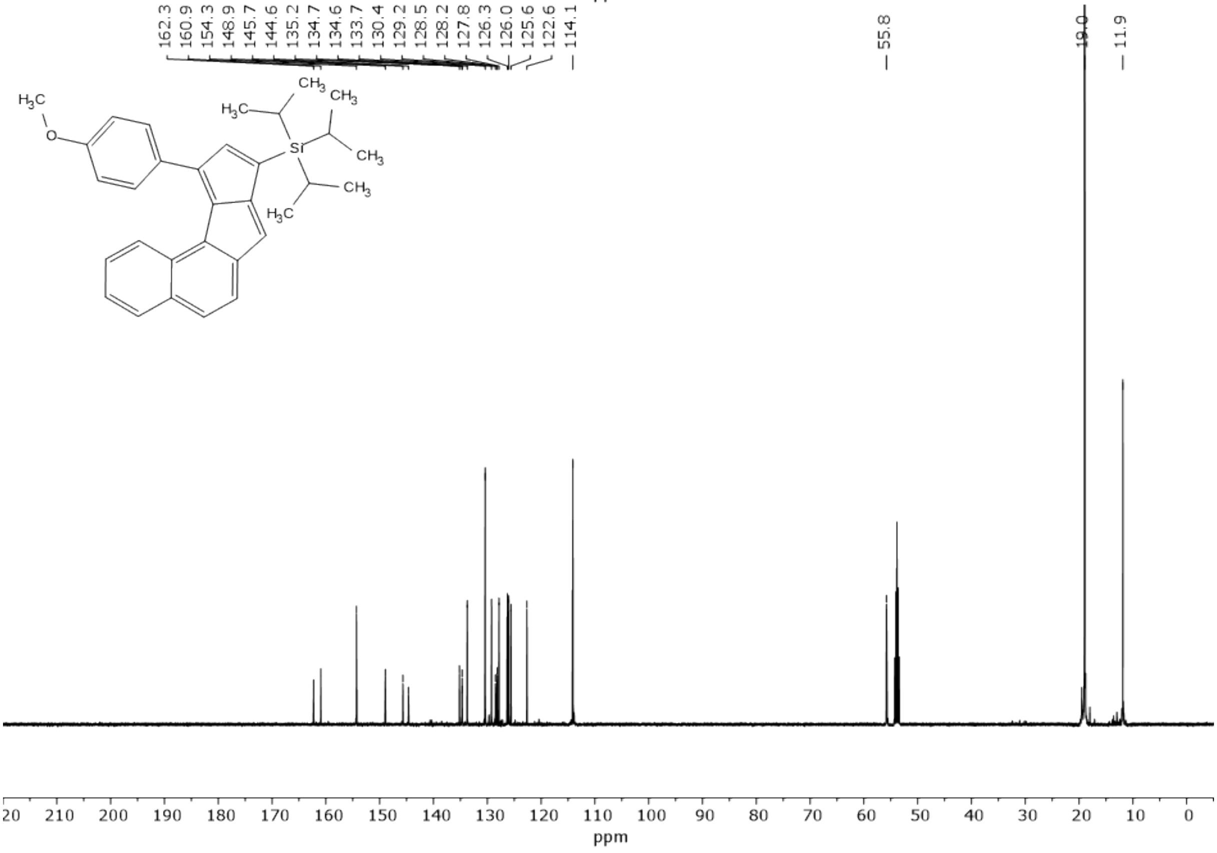

Figure S55. ^{1}H NMR spectrum (top) and $^{13}C\{^{1}H\}$ NMR spectrum (bottom) of **2’** (CD_2Cl_2, 500 MHz).

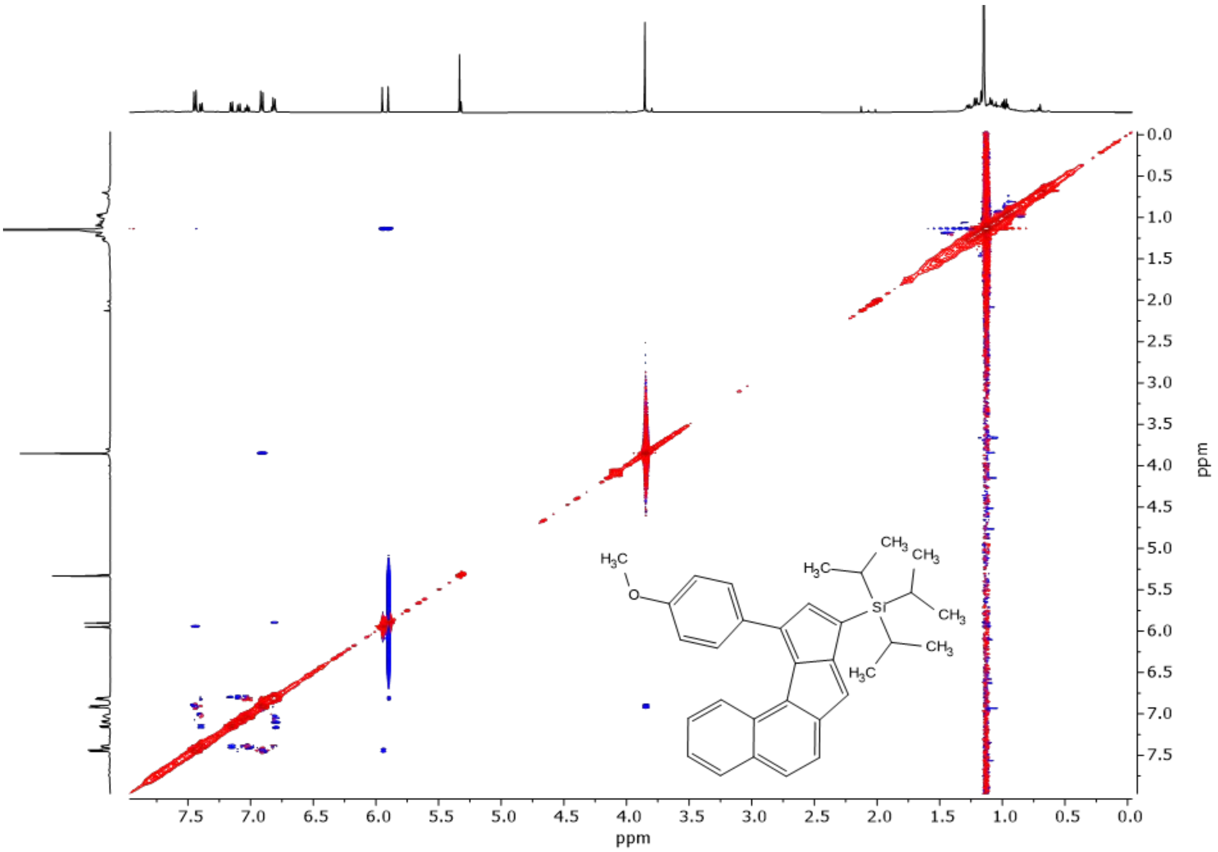

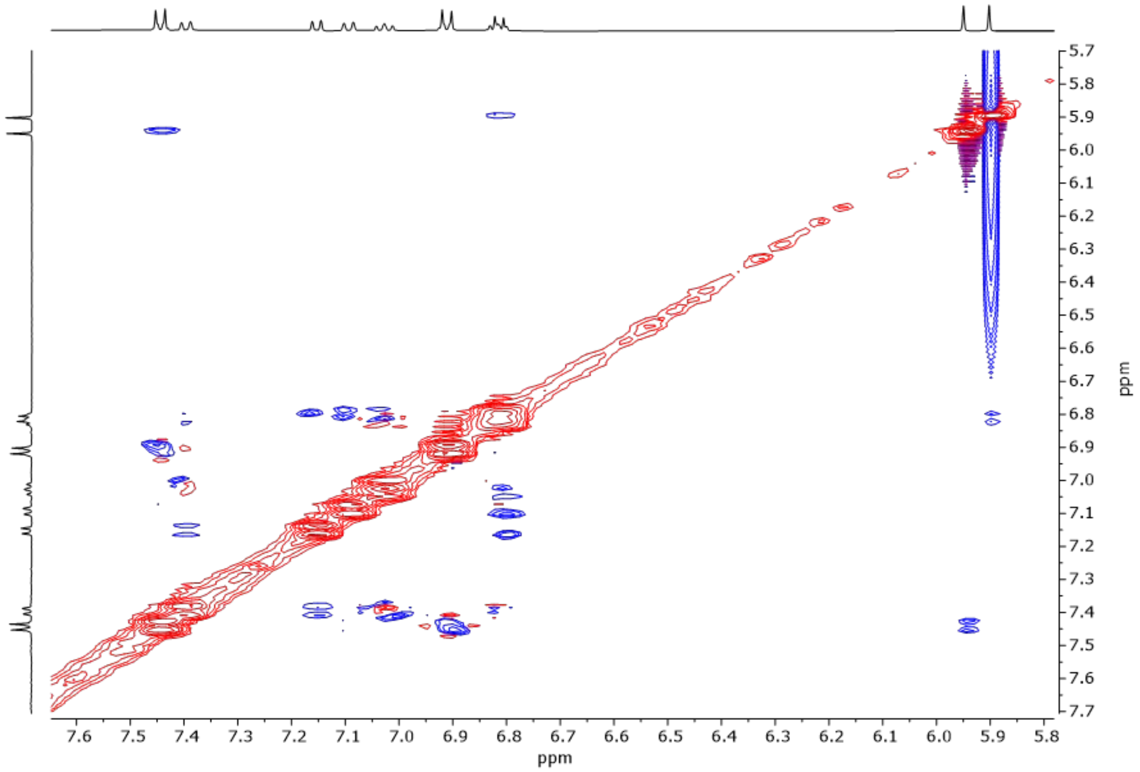

Figure S56. NOESY spectrum of **2'** (CD_2Cl_2, 300 MHz, top-full, bottom-zoomed in on aromatic region).

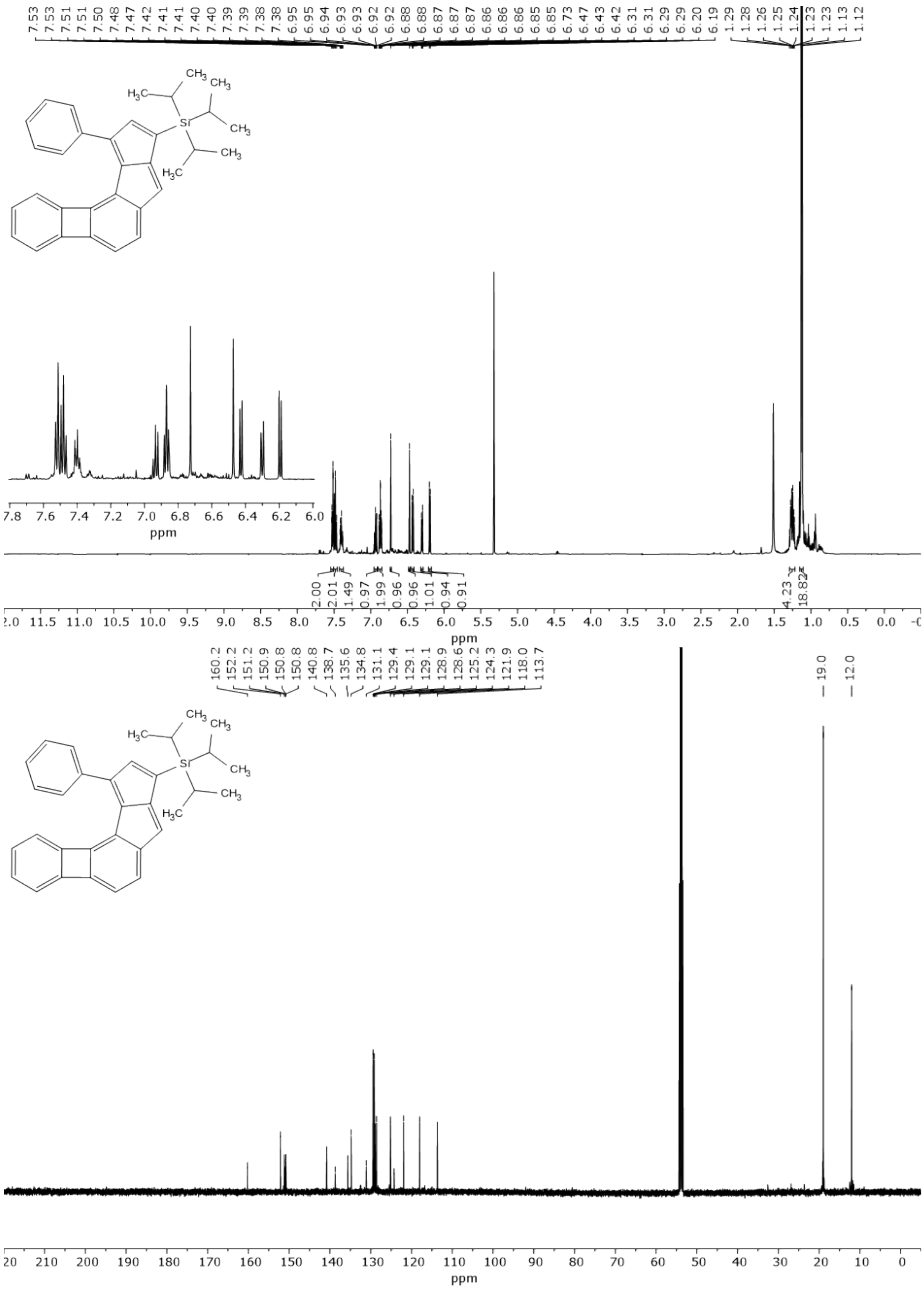

Figure S57. ^{1}H NMR spectrum (top) and $^{13}C\{^{1}H\}$ NMR spectrum (bottom) of **3** (CD_2Cl_2, 500 MHz).

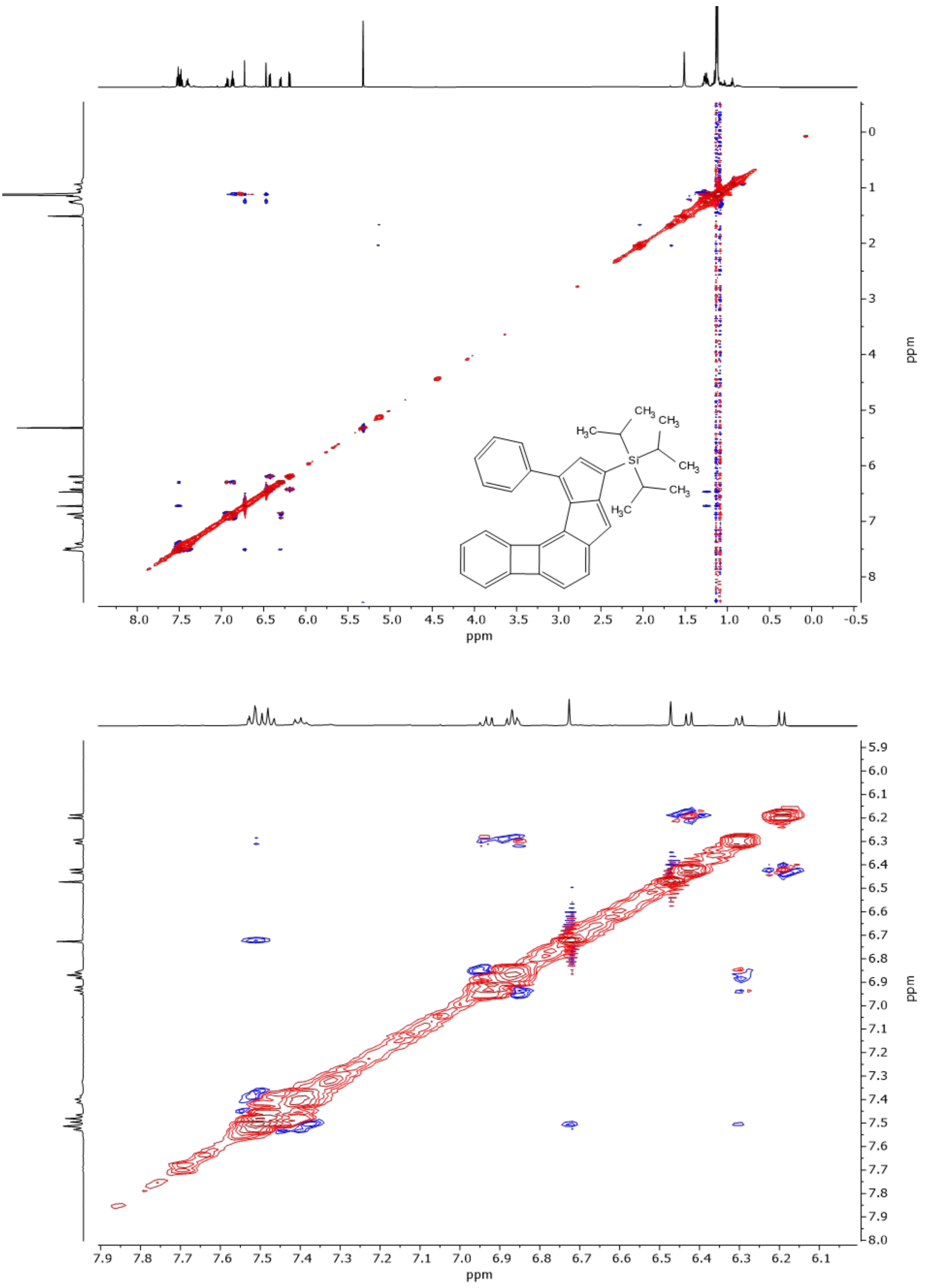

Figure S58. NOESY spectrum of **3** (CD_2Cl_2, 300 MHz, top-full, bottom-zoomed in on aromatic region).

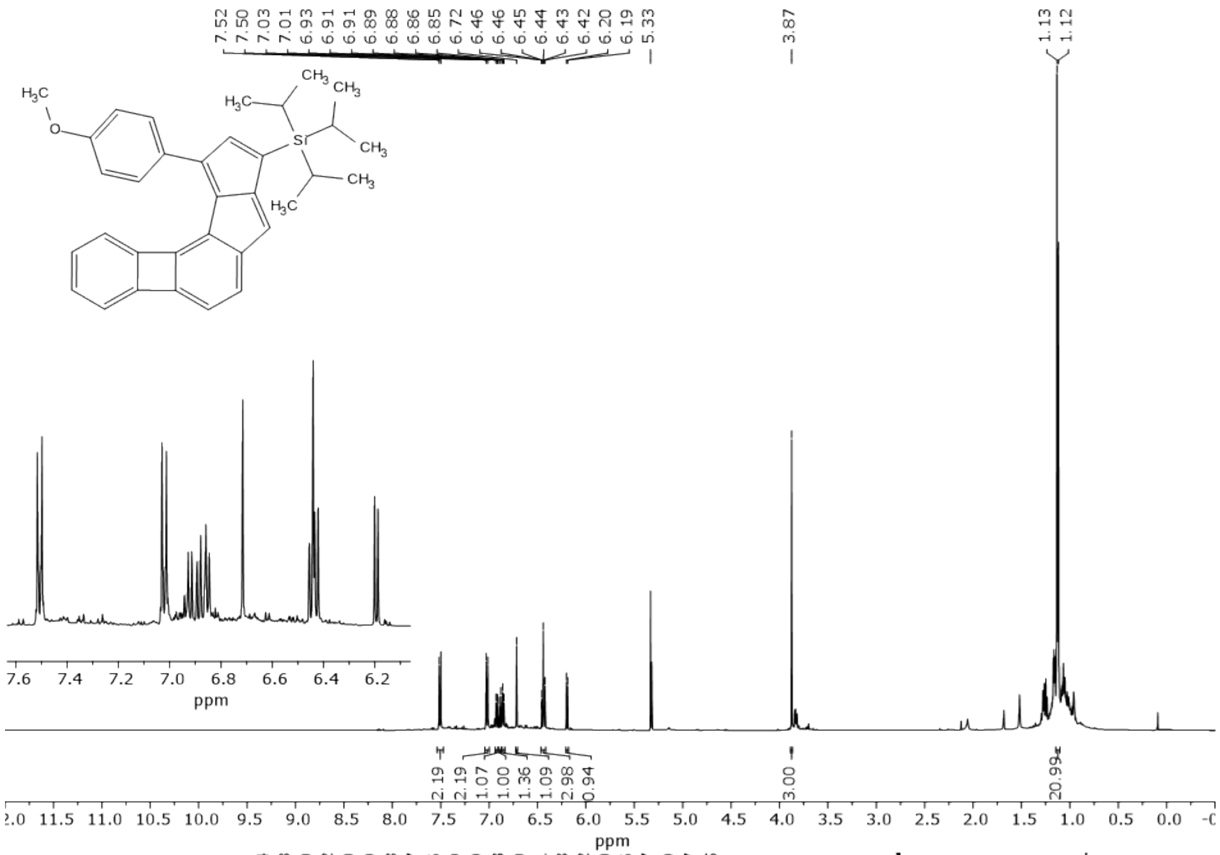

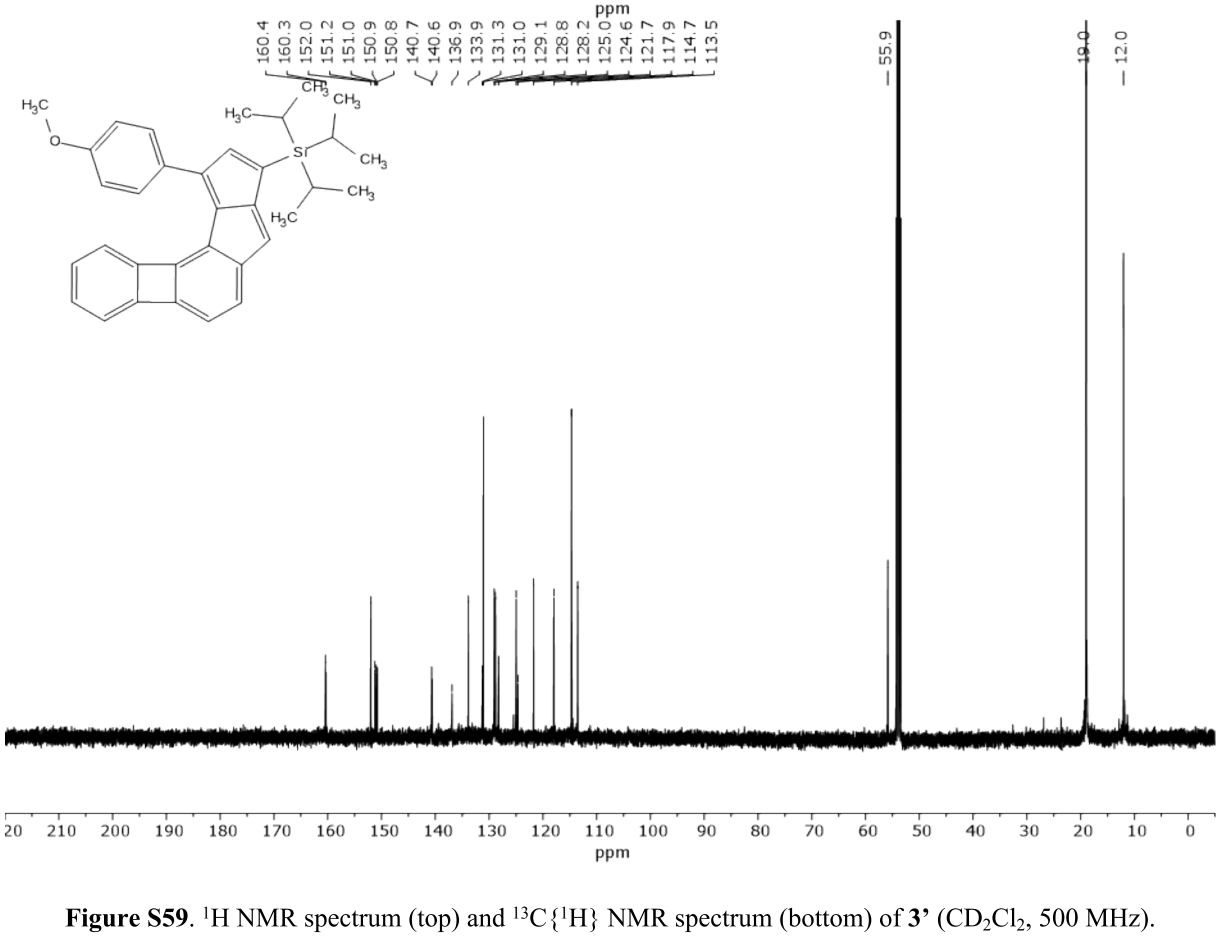

Figure S59. ^{1}H NMR spectrum (top) and $^{13}C\{^{1}H\}$ NMR spectrum (bottom) of **3’** (CD_2Cl_2, 500 MHz).

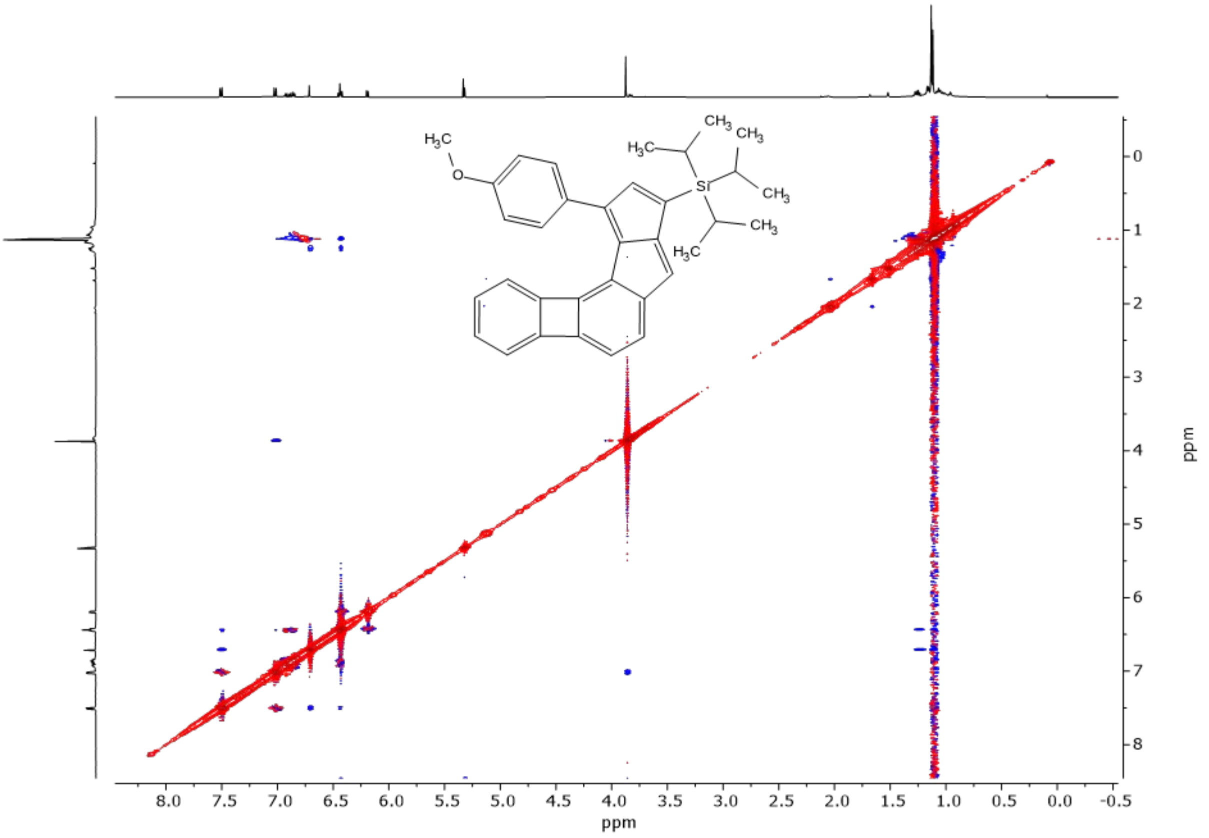

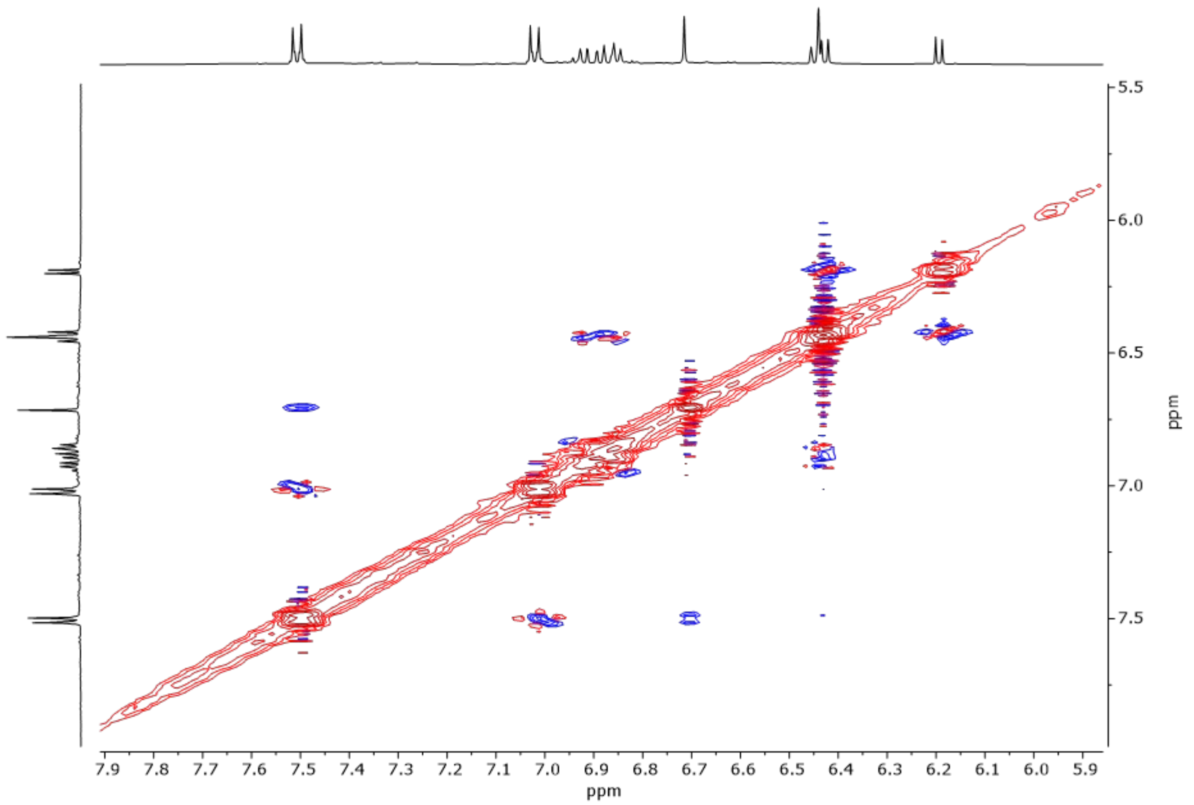

Figure S60. NOESY spectrum of **3'** (CD_2Cl_2, 300 MHz, top-full, bottom-zoomed in on aromatic region).

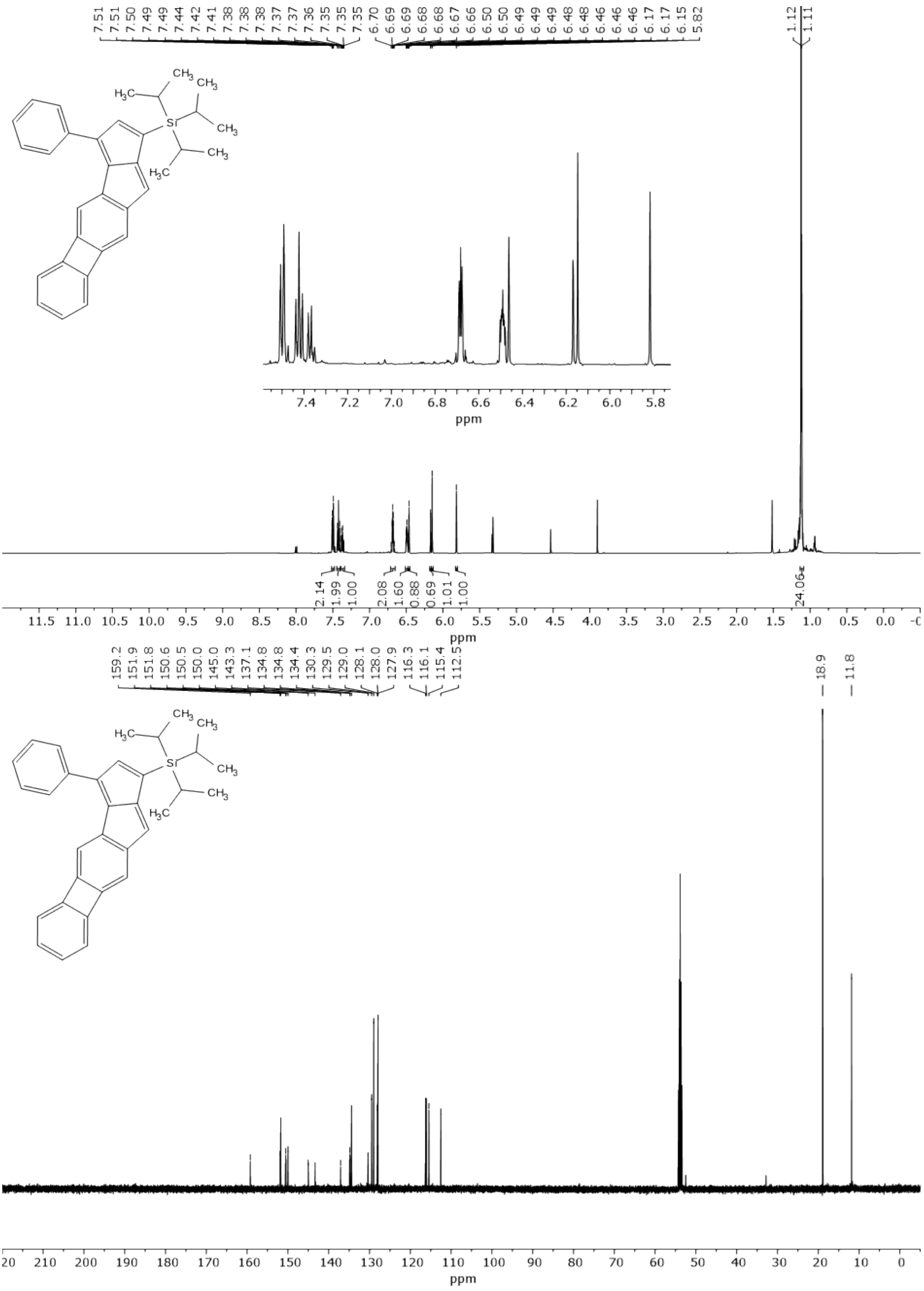

Figure S61. ^{1}H NMR spectrum (top) and $^{13}C\{^{1}H\}$ NMR spectrum (bottom) of **4** (CD_2Cl_2, 500 MHz).

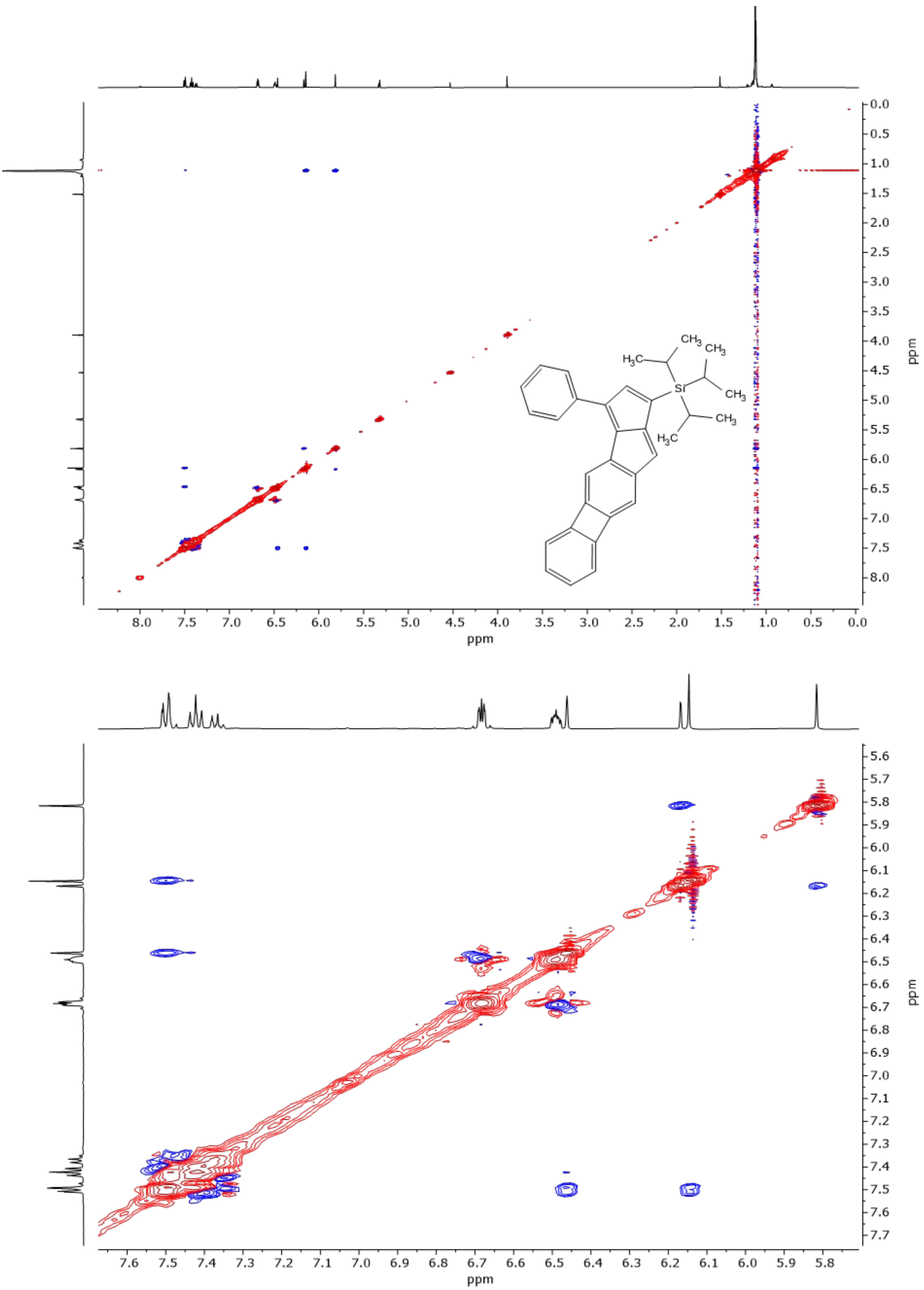

Figure S62. NOESY spectrum of **4** (CD_2Cl_2, 300 MHz, top-full, bottom-zoomed in on aromatic region).

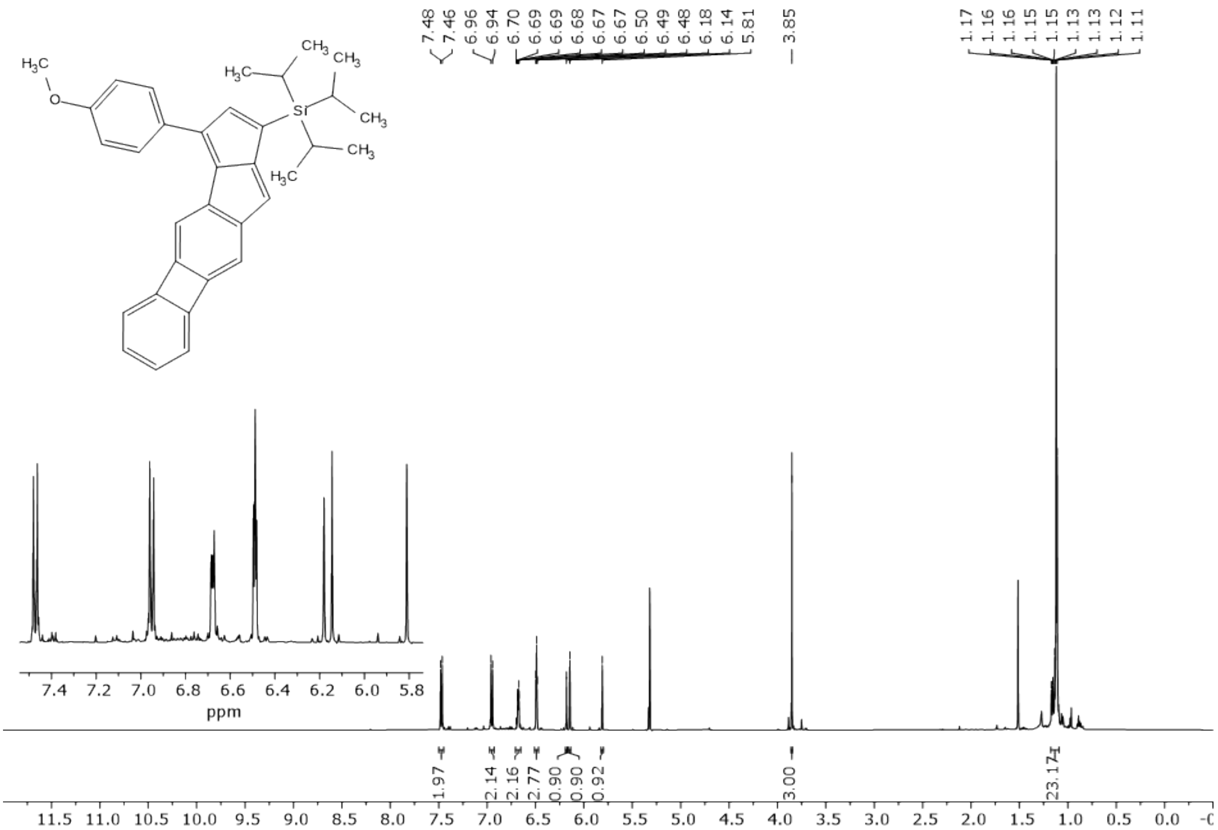

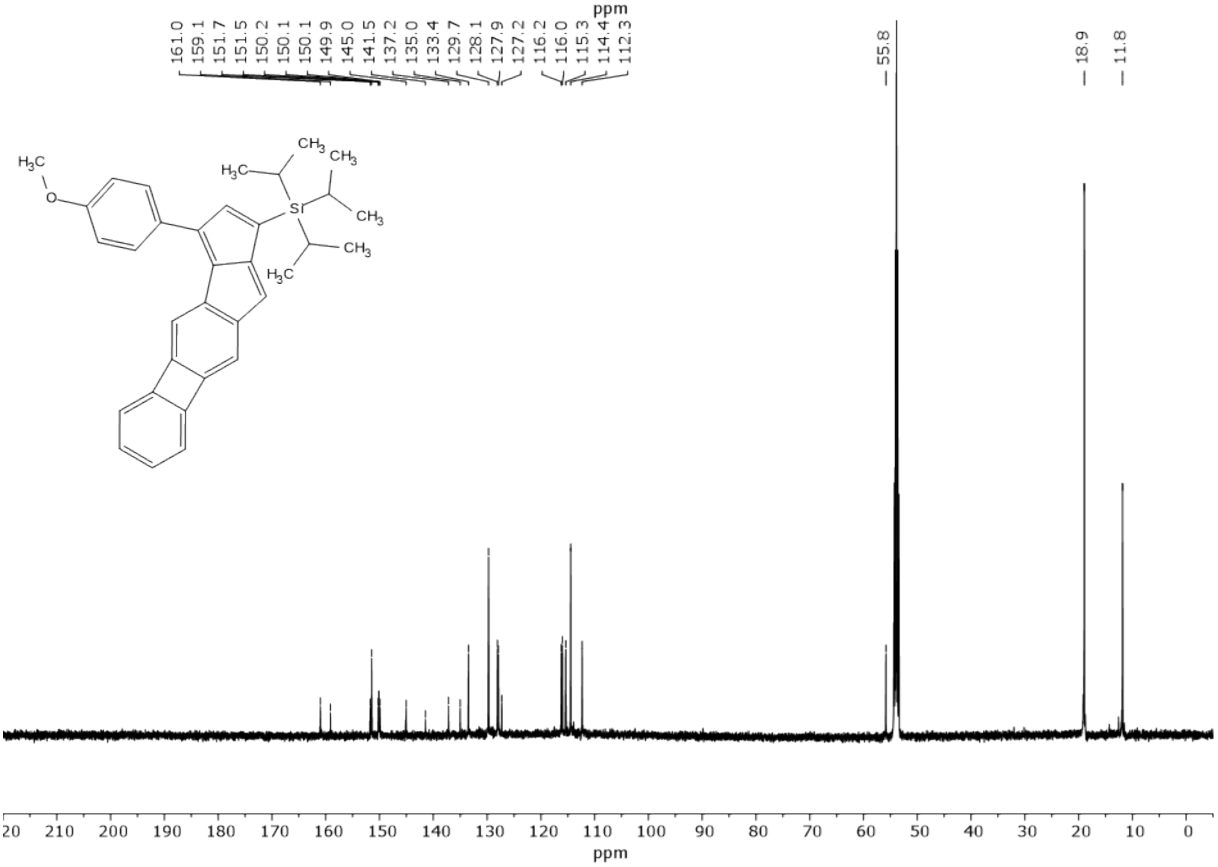

Figure S63. ^{1}H NMR spectrum (top) and $^{13}C\{^{1}H\}$ NMR spectrum (bottom) of **4’** (CD_2Cl_2, 500 MHz).

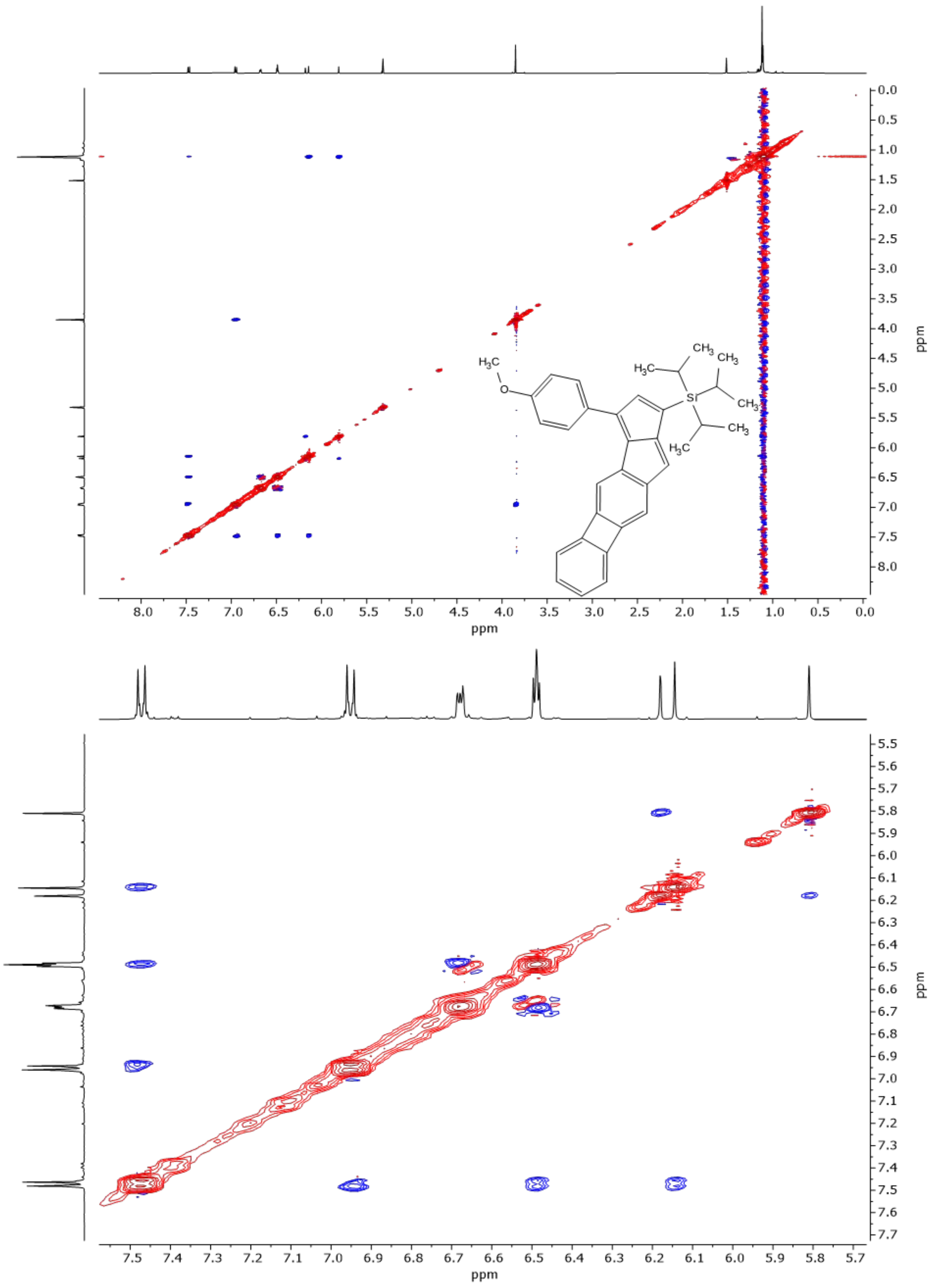

Figure S64. NOESY spectrum of **4’** (CD_2Cl_2, 300 MHz, top-full, bottom-zoomed in on aromatic region).

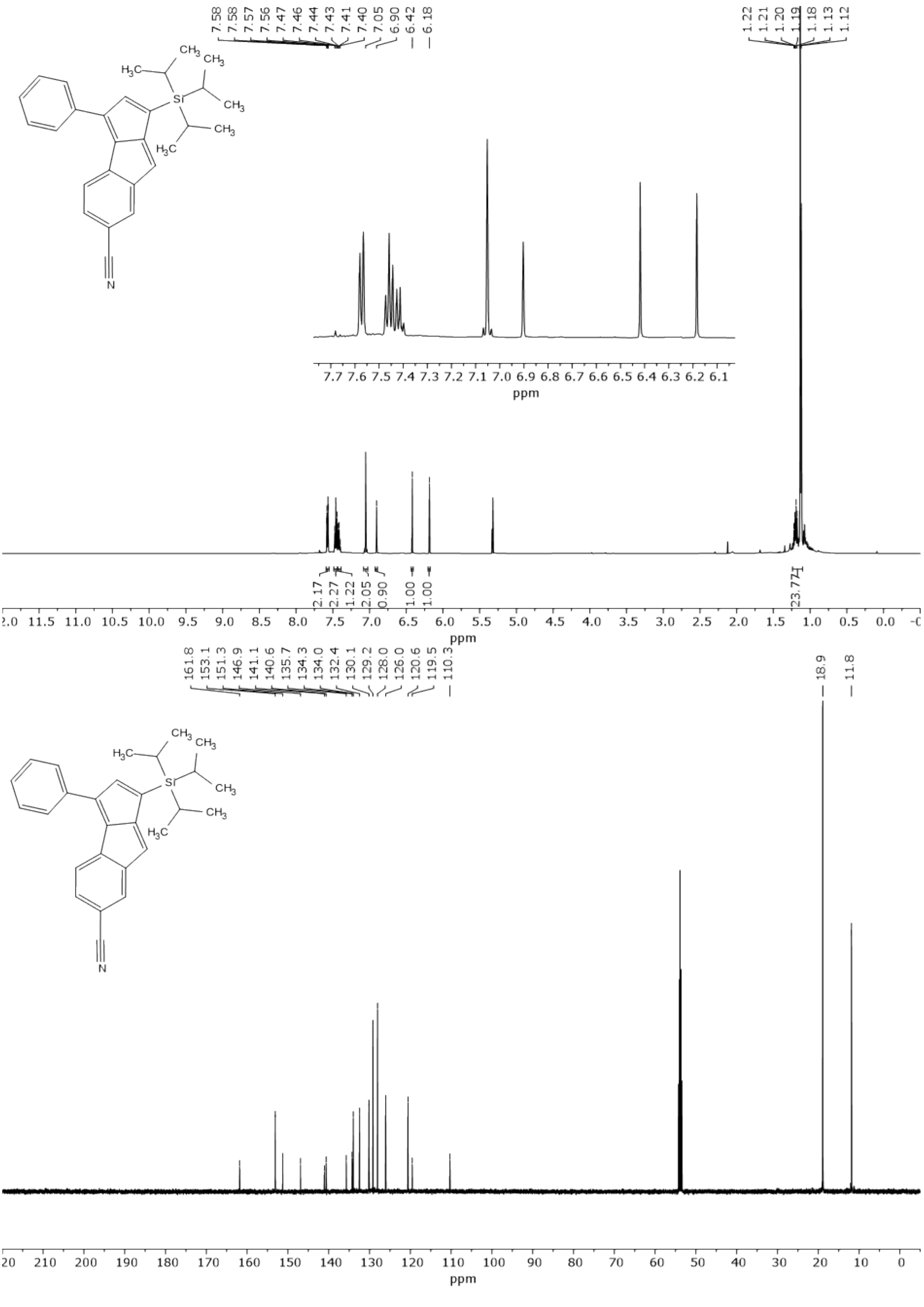

Figure S65. ^{1}H NMR spectrum (top) and ^{13}C{^{1}H} NMR spectrum (bottom) of **5** (CD_2Cl_2, 500 MHz).

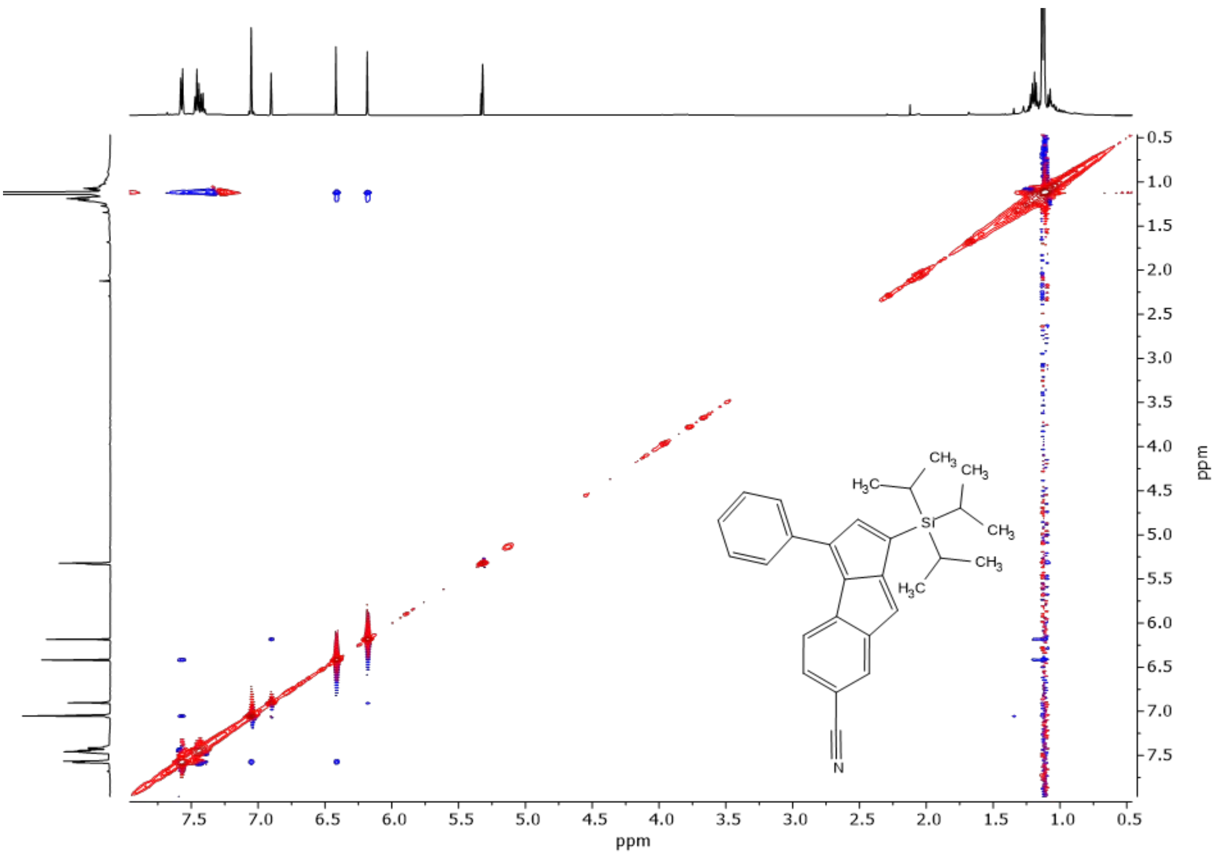

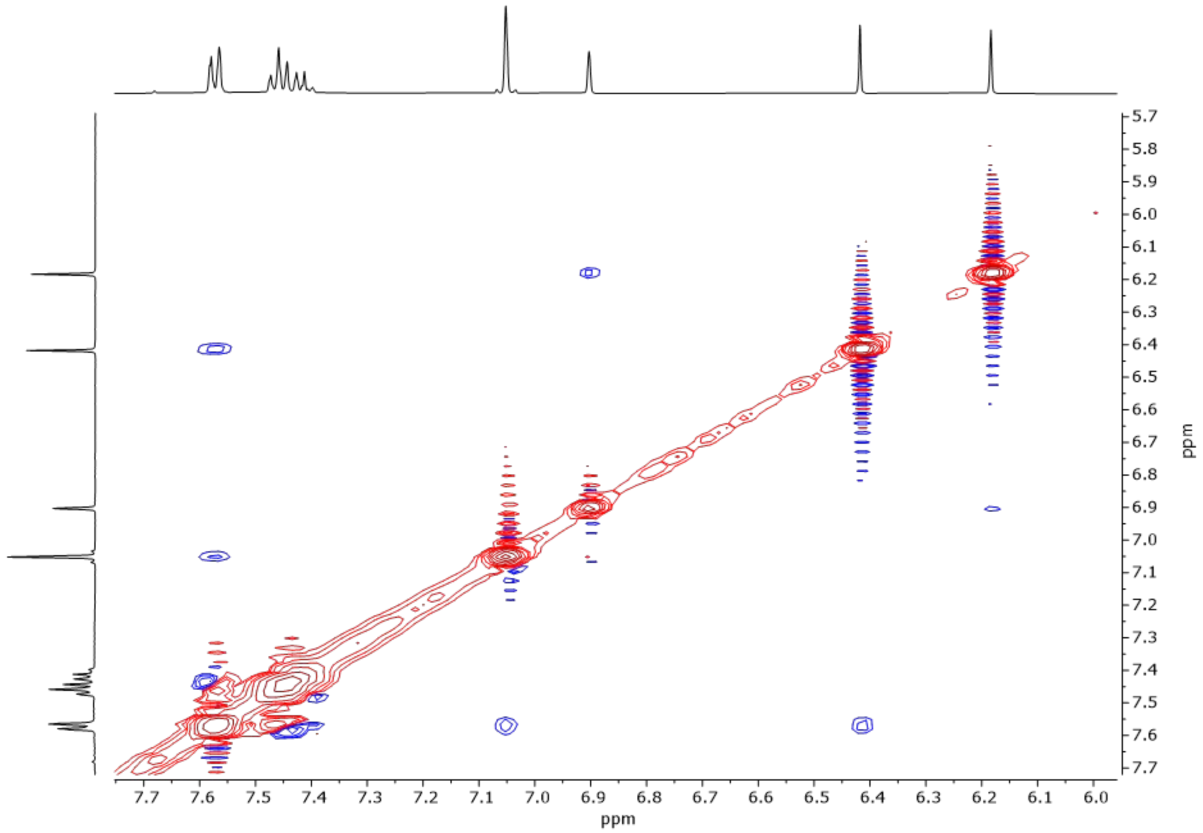

Figure S66. NOESY spectrum of **5** (CD_2Cl_2, 300 MHz, top-full, bottom-zoomed in on aromatic region).

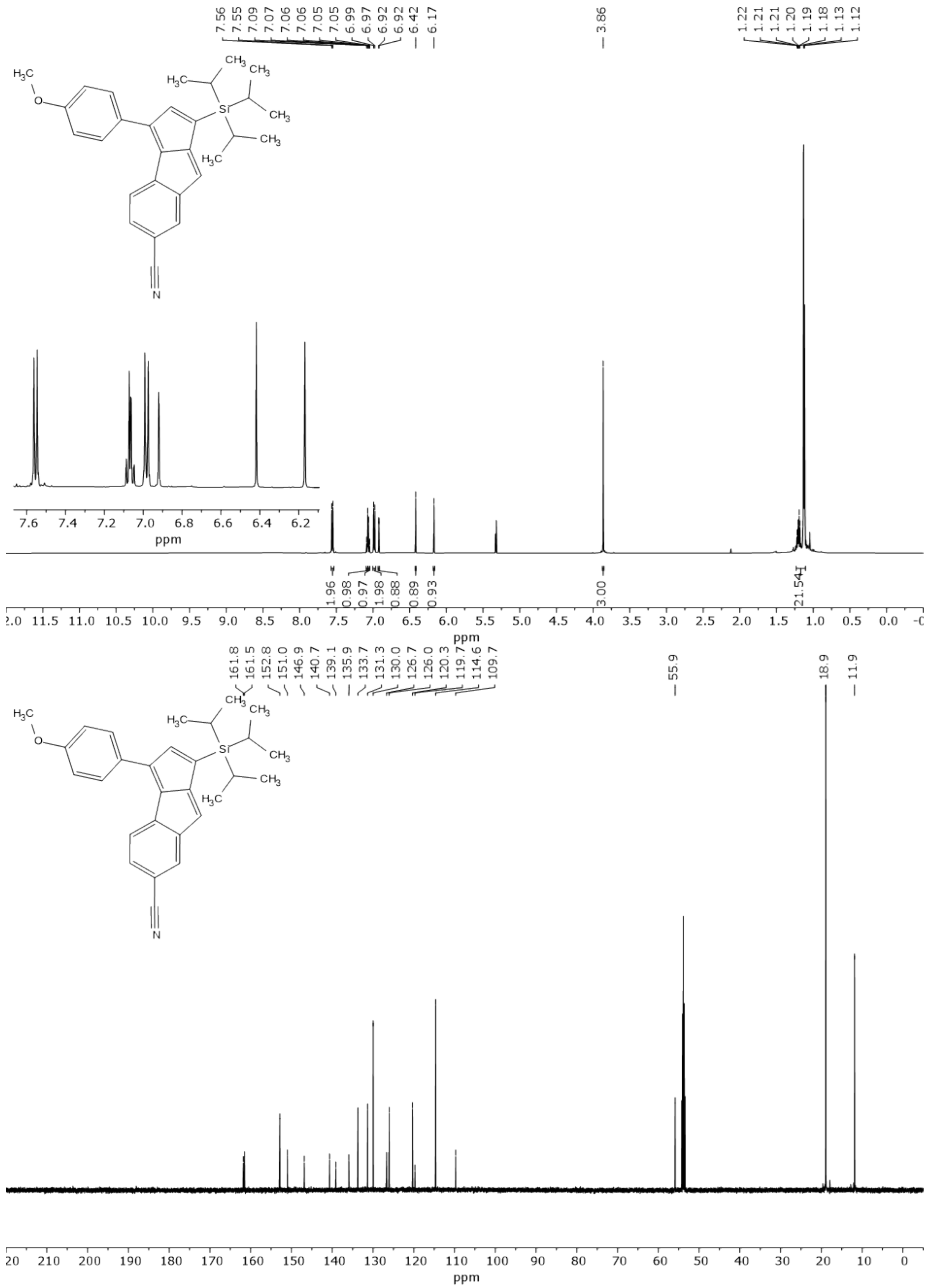

Figure S67. ^{1}H NMR spectrum (top) and $^{13}C\{^{1}H\}$ NMR spectrum (bottom) of **5'** (CD_2Cl_2, 500 MHz).

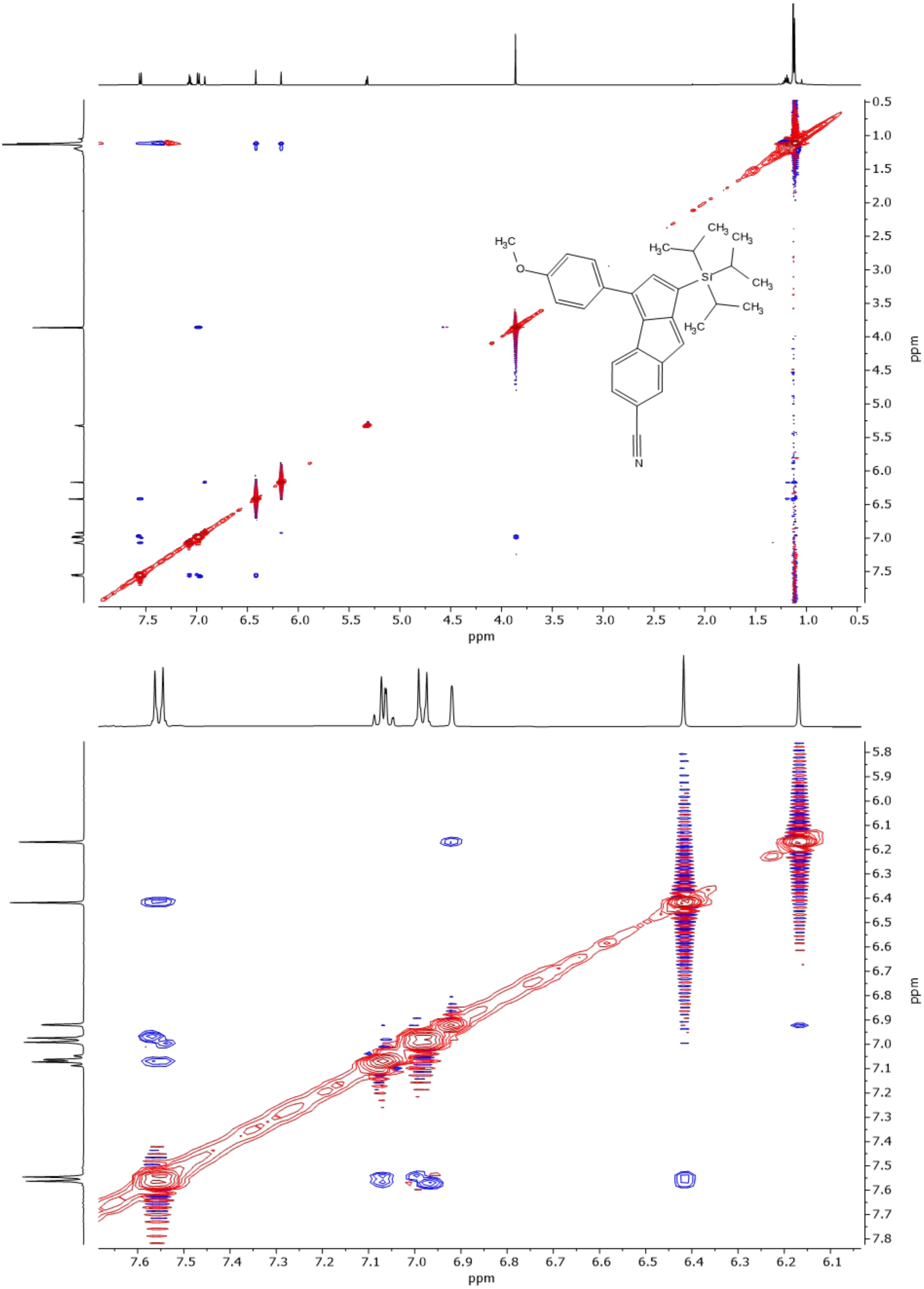

Figure S68. NOESY spectrum of **5’** (CD_2Cl_2, 300 MHz, top-full, bottom-zoomed in on aromatic region).

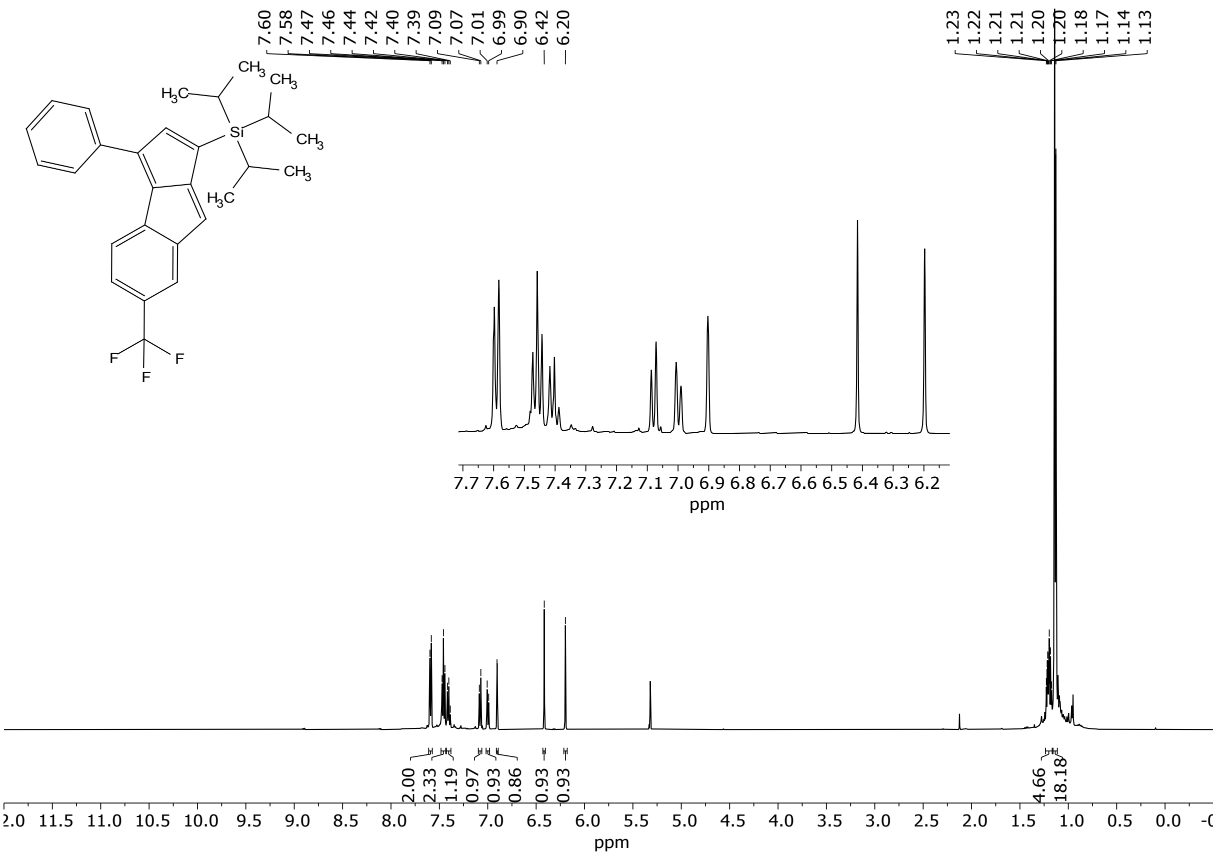

Figure S69. ^{1}H NMR spectrum of **6** (CD_2Cl_2, 500 MHz).

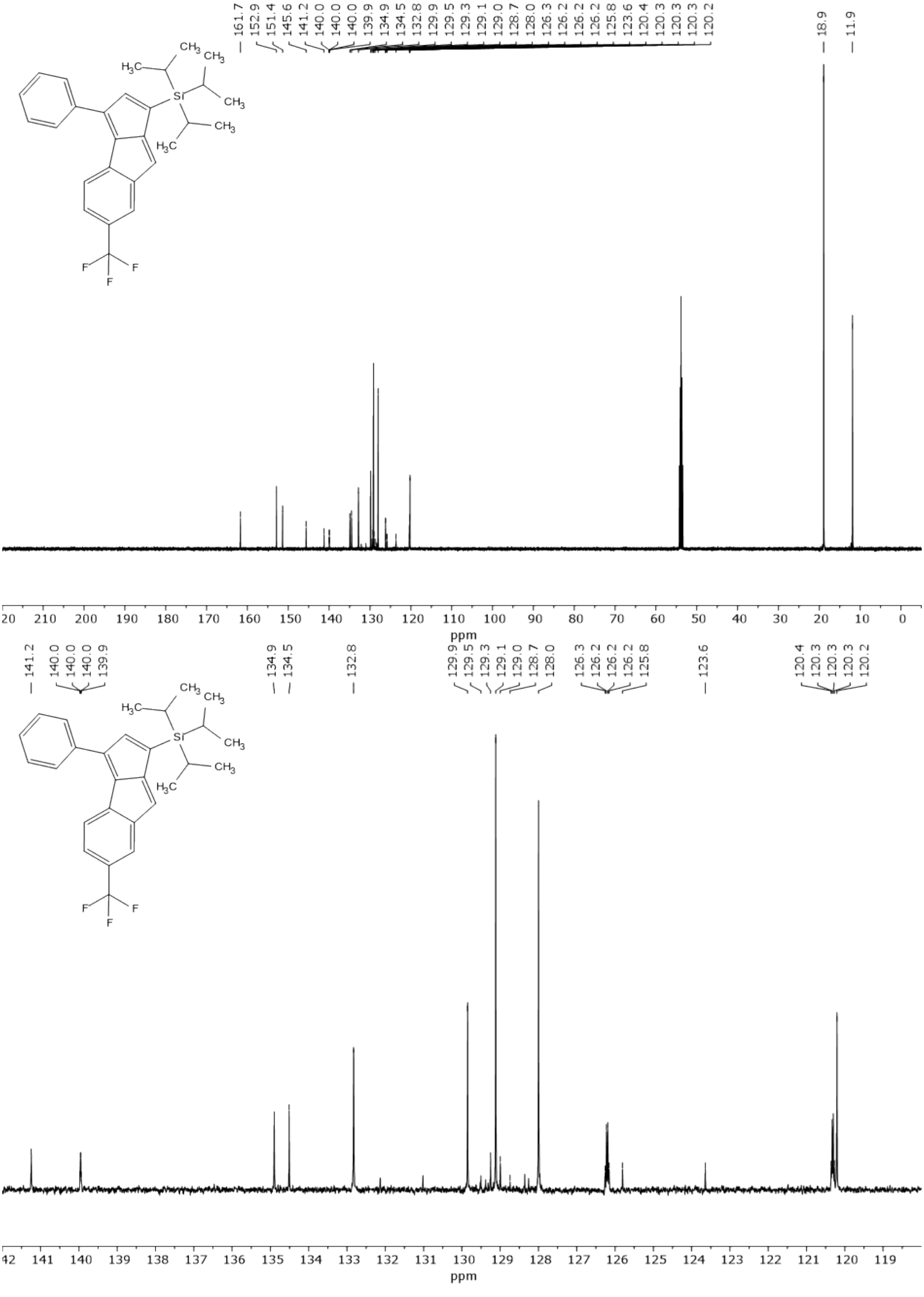

Figure S70. $^{13}C\{^{1}H\}$ NMR spectrum of **6** (CD_2Cl_2, 500 MHz, top-full, bottom-zoomed in on aromatic region).

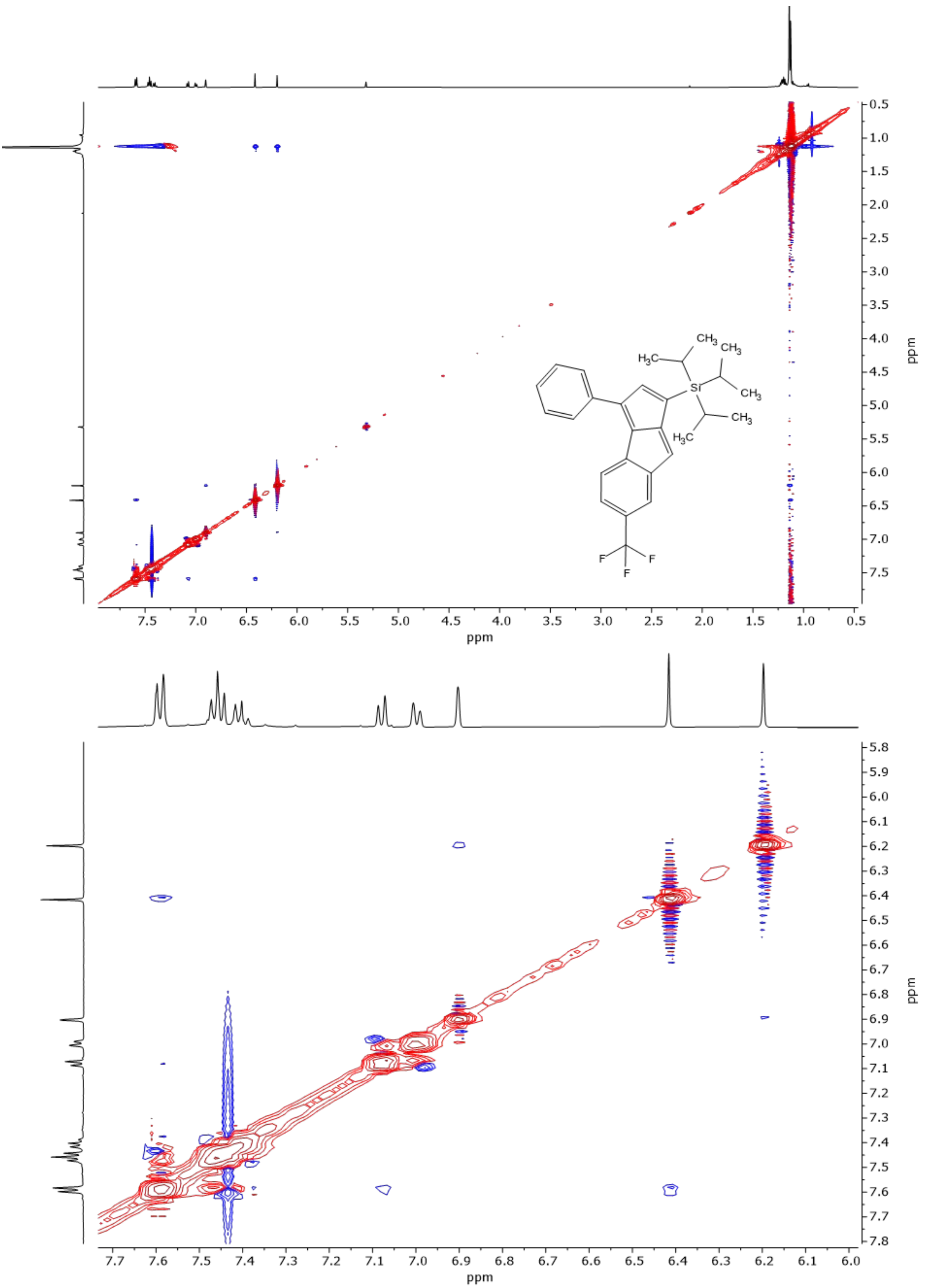

Figure S71. NOESY spectrum of **6** (CD_2Cl_2, 300 MHz, top-full, bottom-zoomed in on aromatic region).

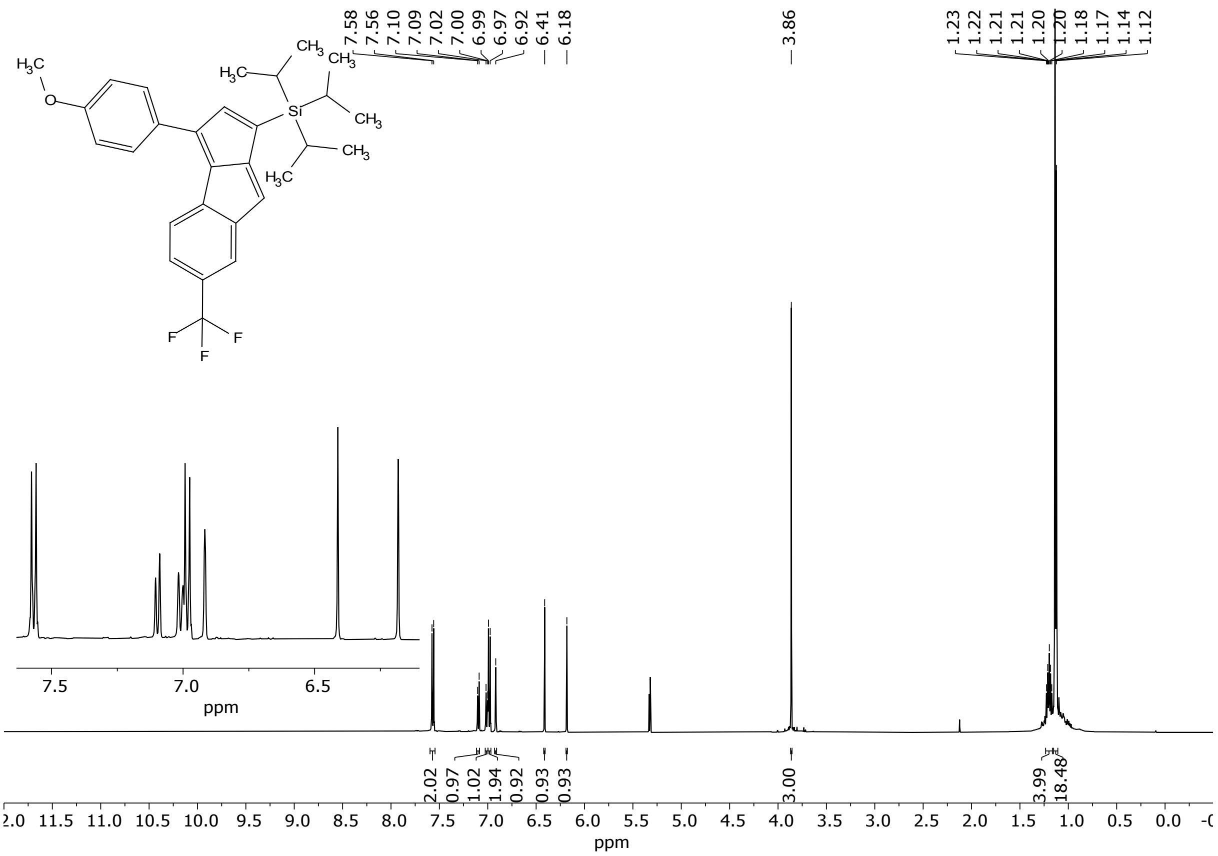

Figure S72. ^{1}H NMR spectrum of **6’** (CD_2Cl_2, 500 MHz).

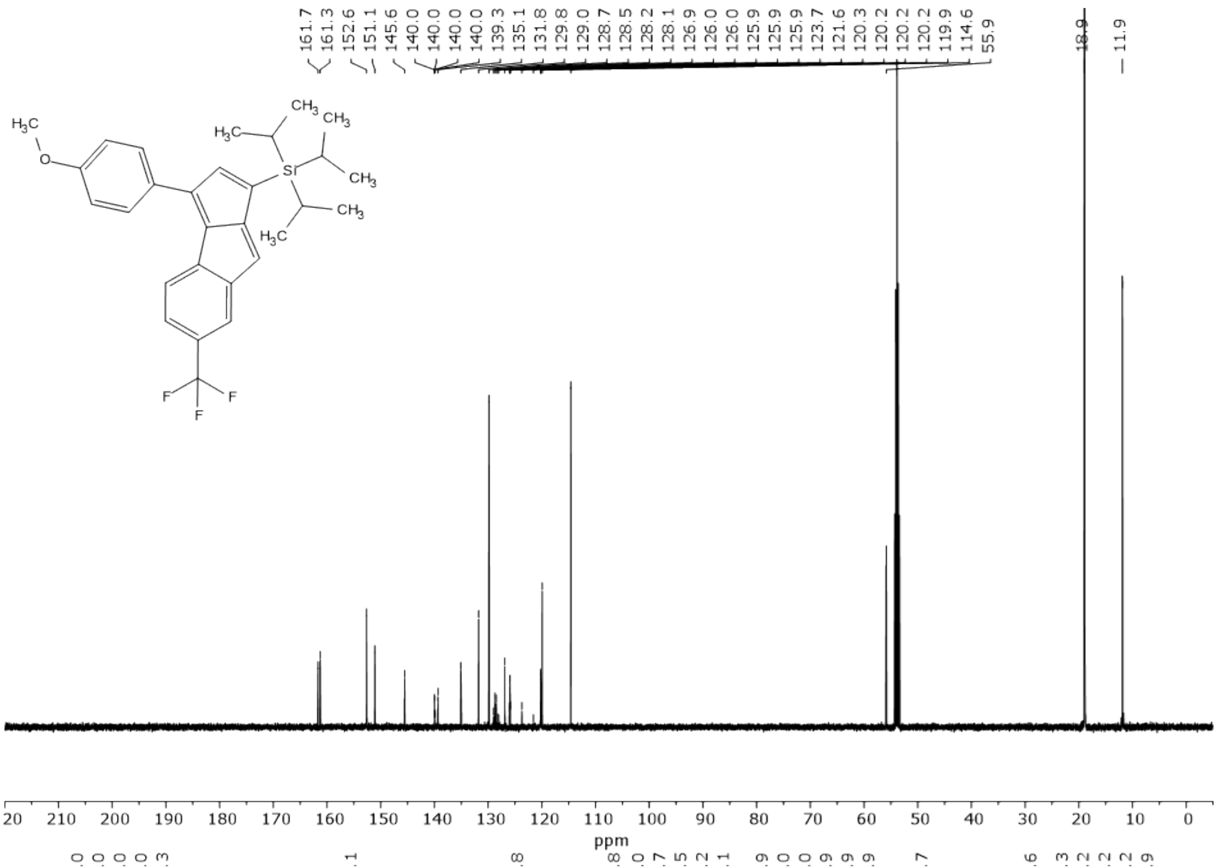

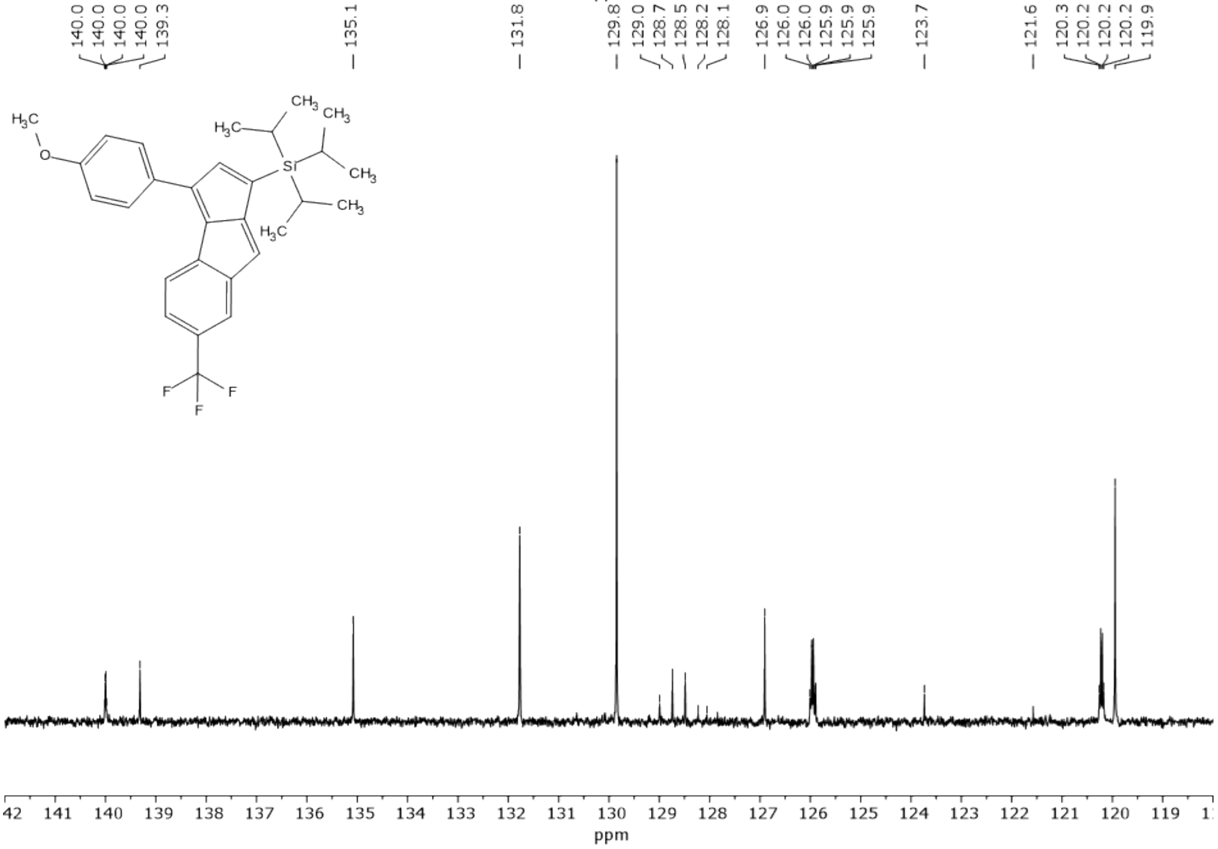

Figure S73. $^{13}C\{^1H\}$ NMR spectrum of **6'** (CD_2Cl_2, 500 MHz, top-full, bottom-zoomed in on aromatic region).

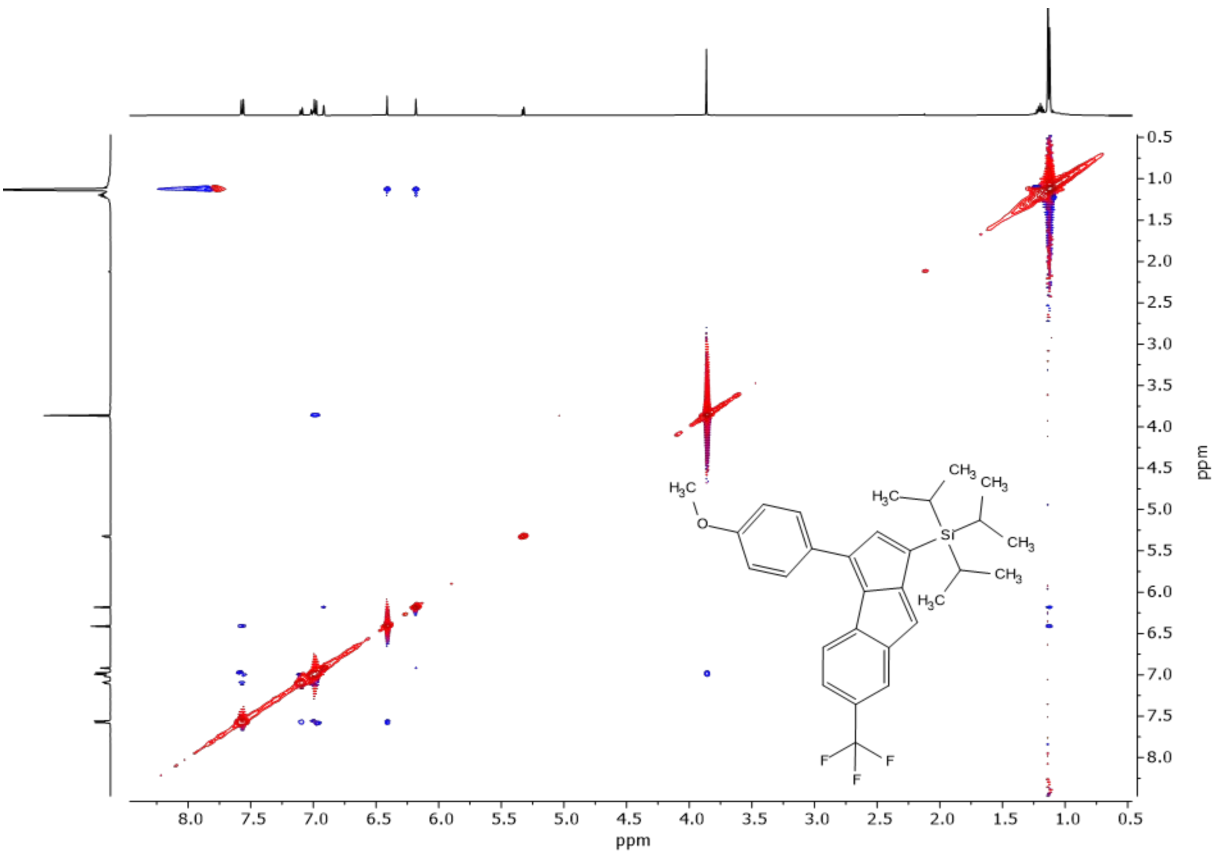

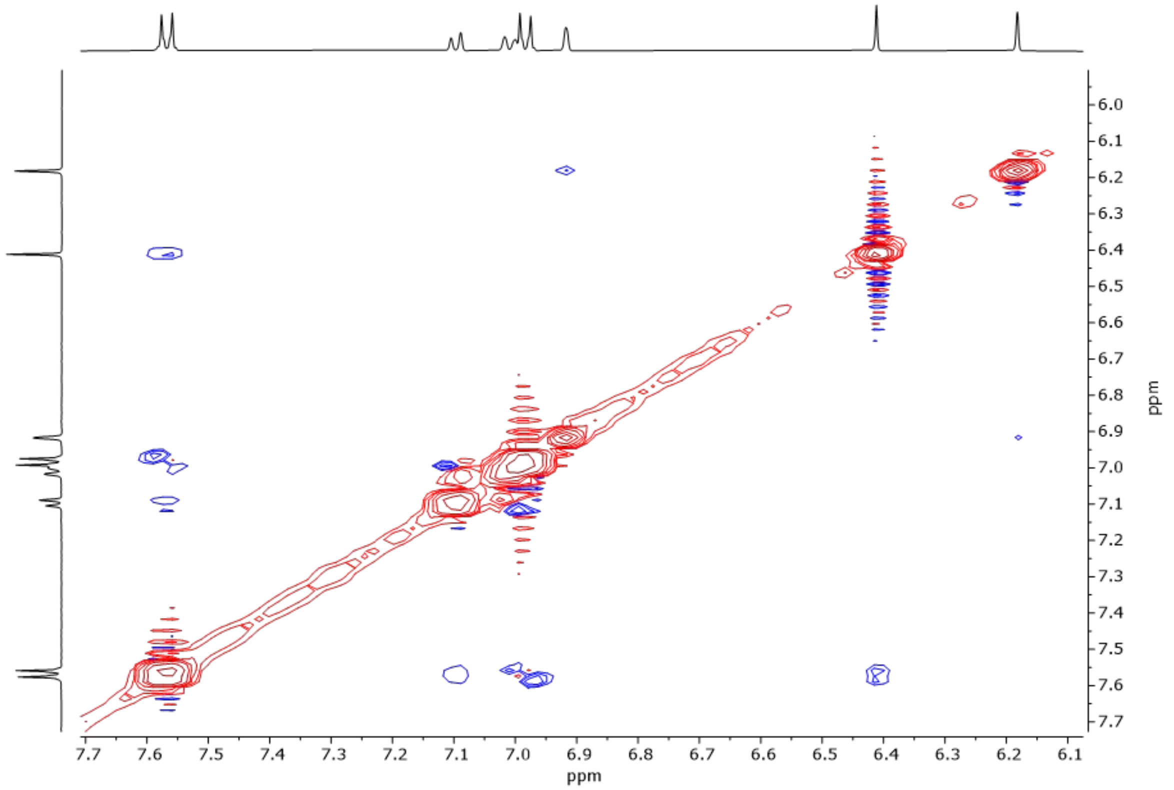

Figure S74. NOESY spectrum of **6'** (CD_2Cl_2, 300 MHz, top-full, bottom-zoomed in on aromatic region).

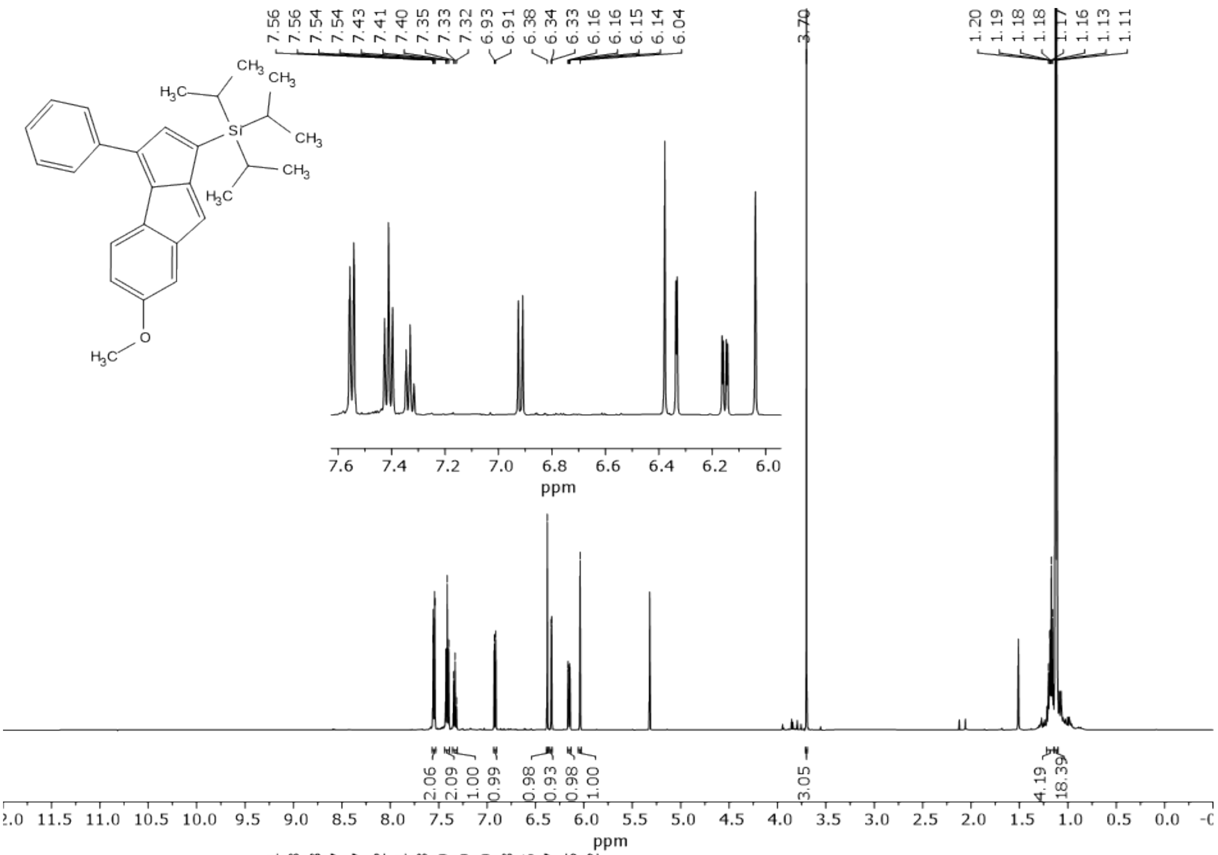

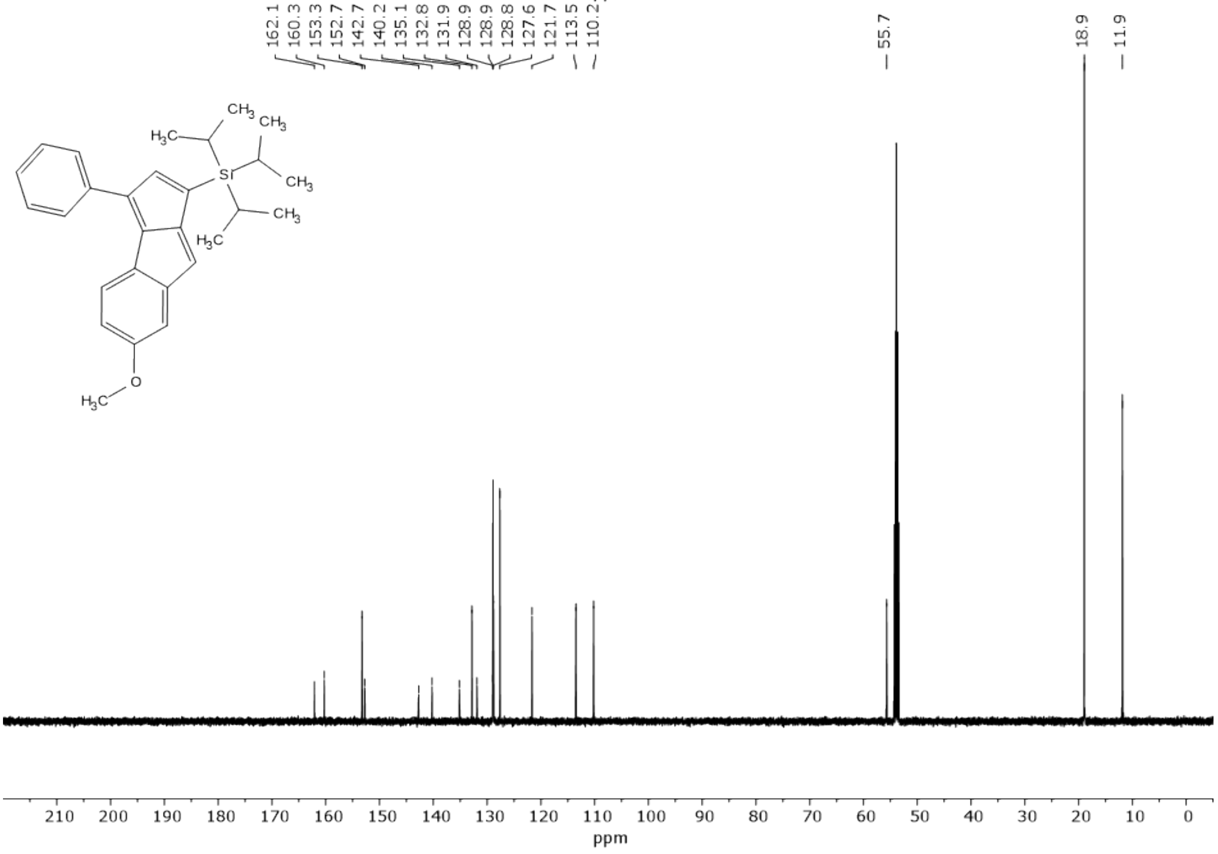

Figure S75. ^{1}H NMR spectrum (top) and ^{13}C{^{1}H} NMR spectra (bottom) of **7** (CD_2Cl_2, 500 MHz).

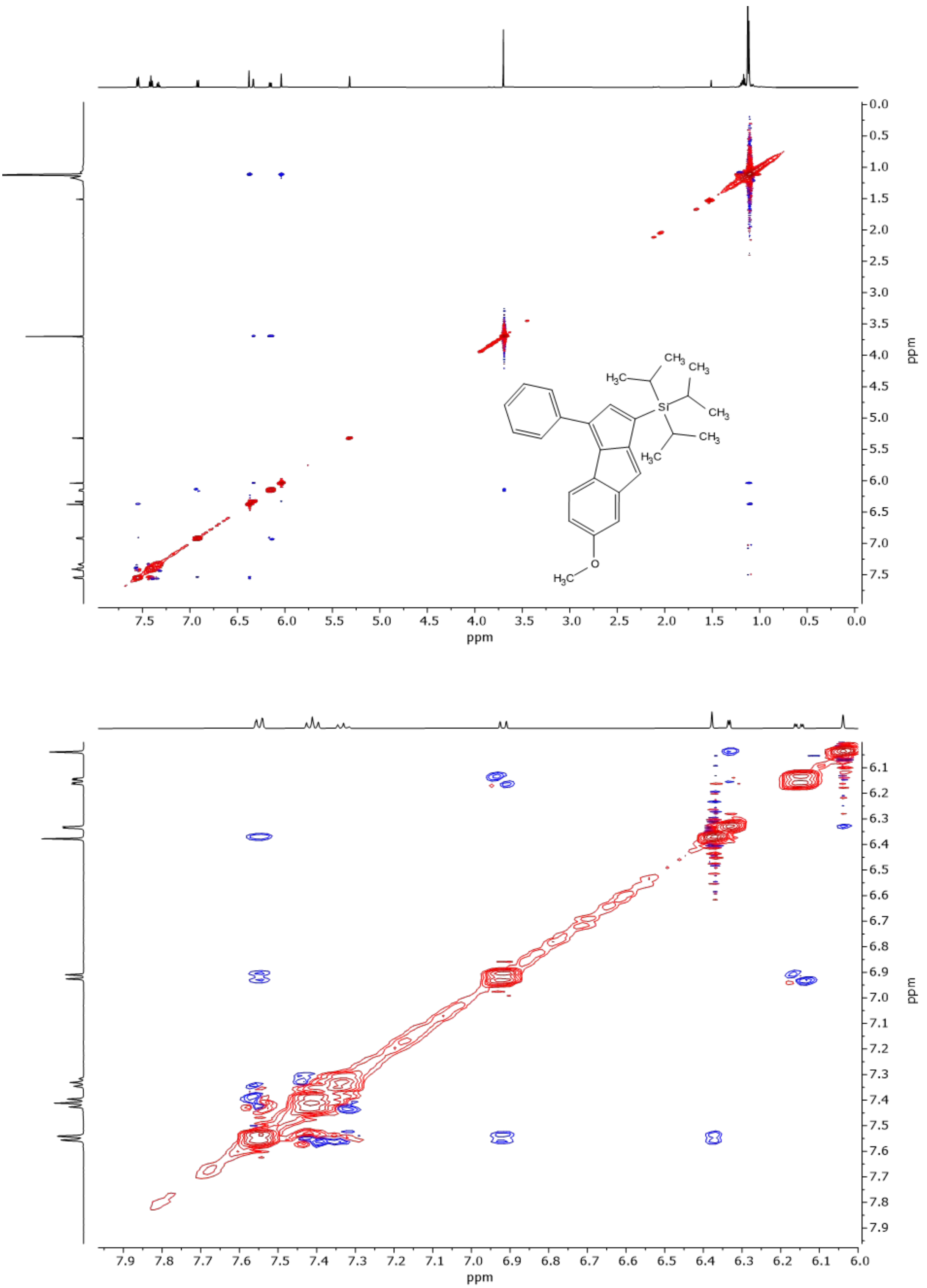

Figure S76. NOESY spectrum of **7** (CD_2Cl_2, 300 MHz, top-full, bottom-zoomed in on aromatic region).

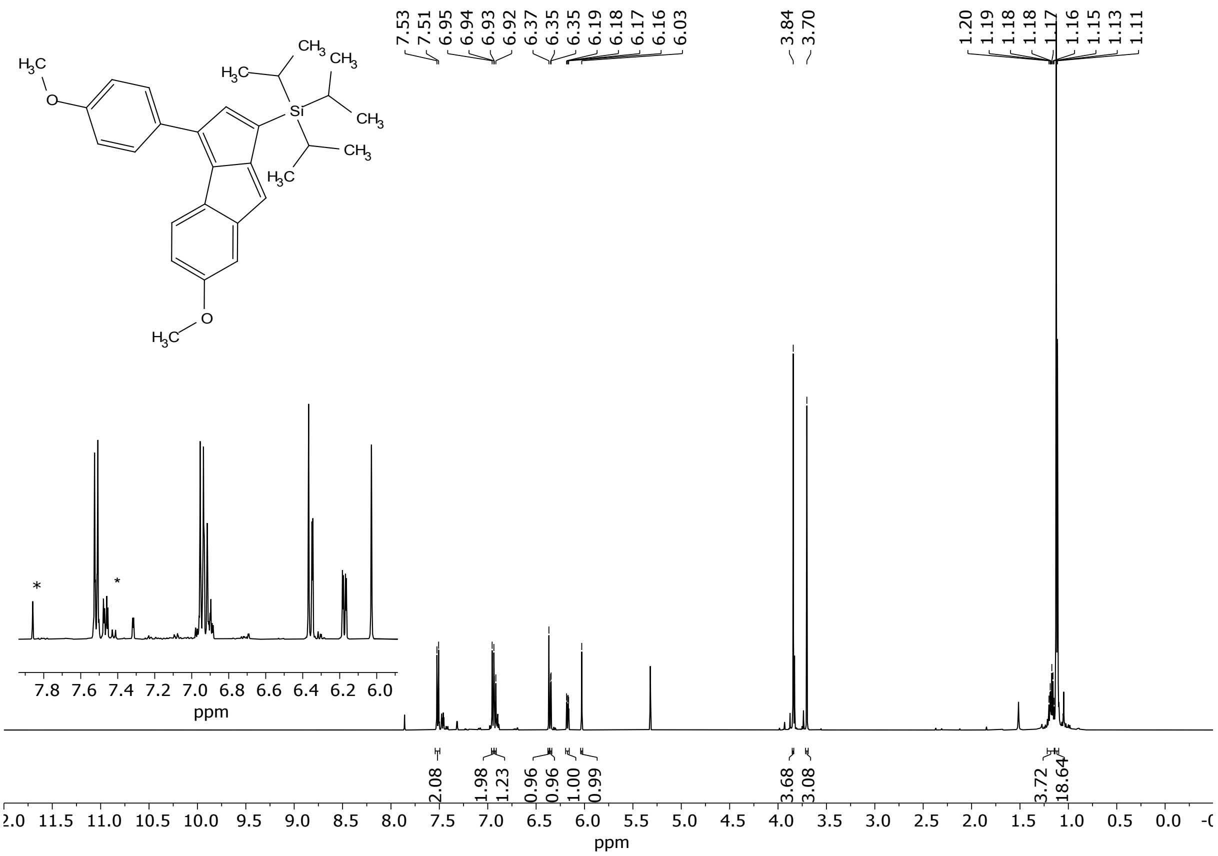

Figure S77. ^{1}H NMR spectrum **7’** (CD_2Cl_2, 500 MHz). * indicates residual starting material

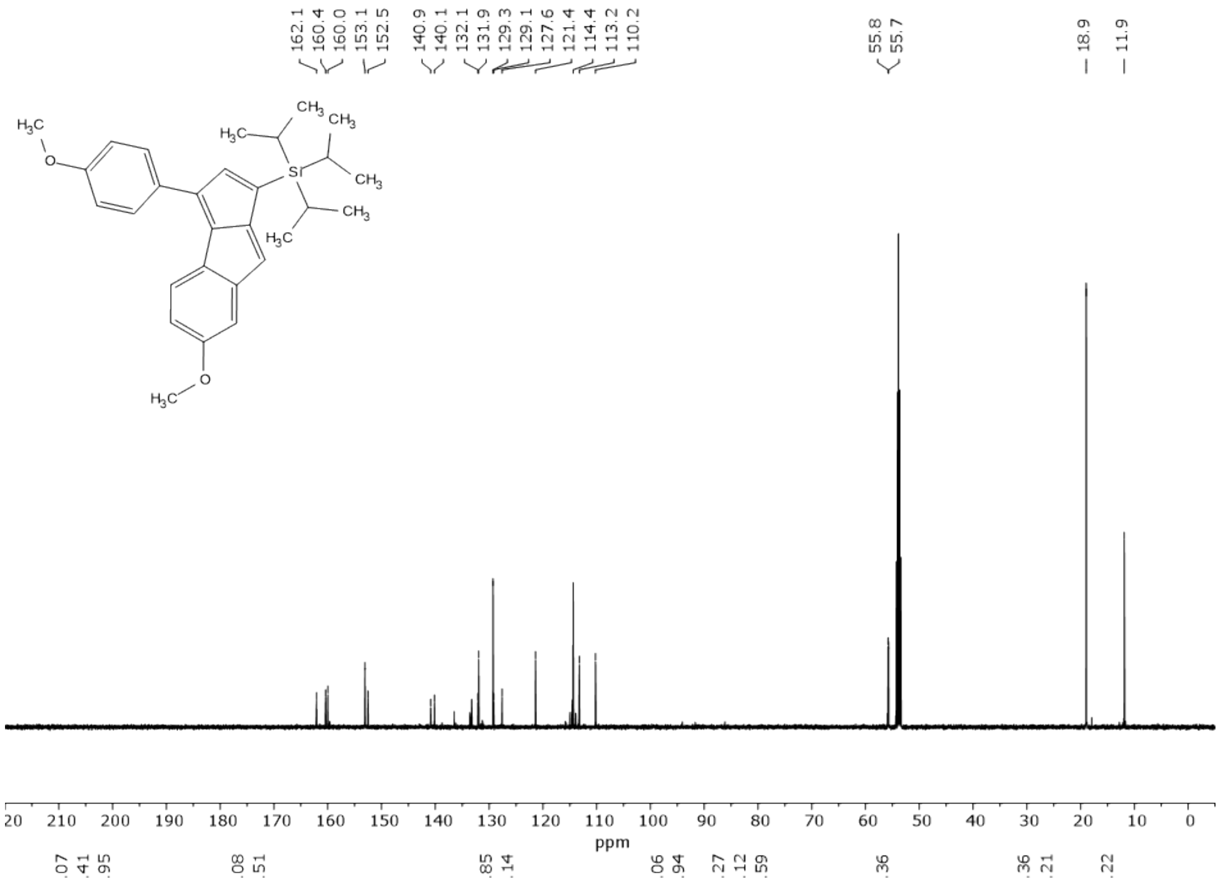

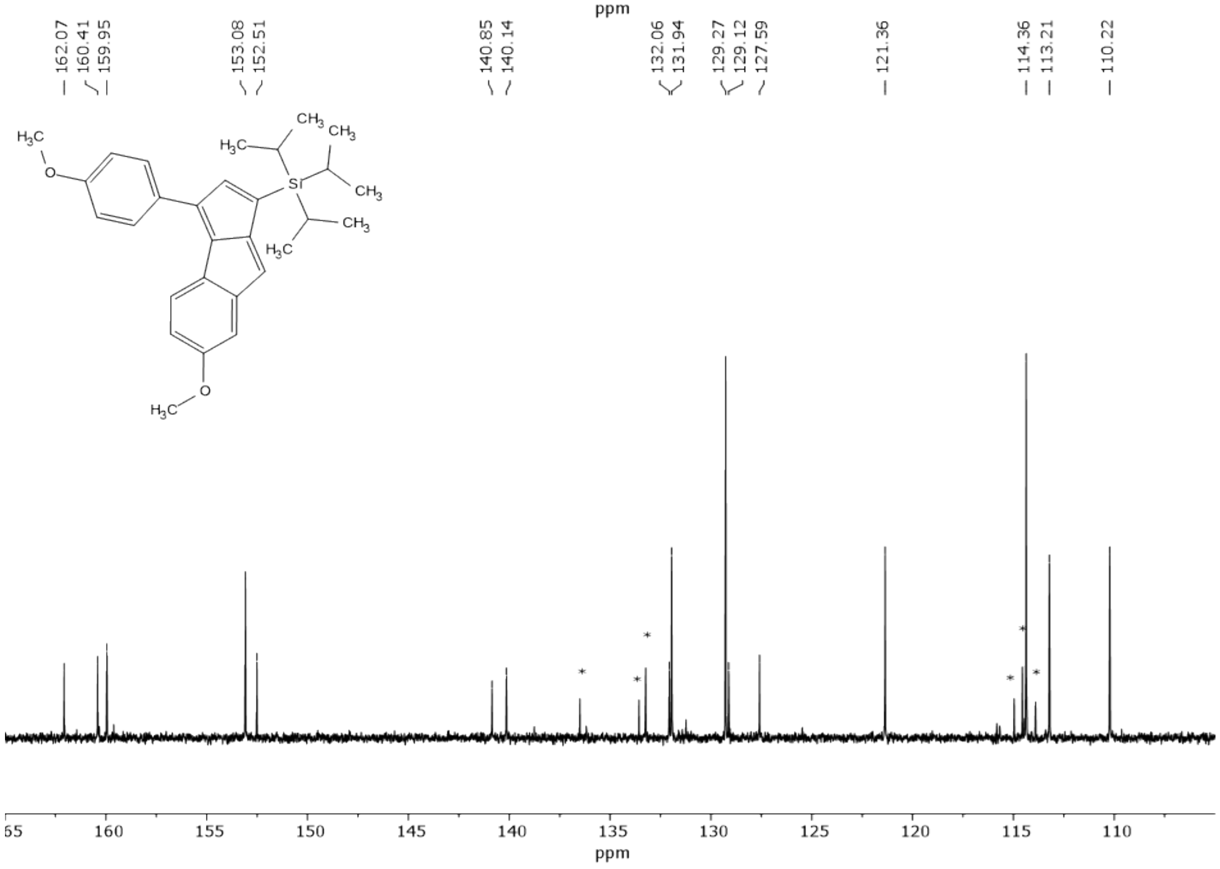

Figure S78. $^{13}C\{^{1}H\}$ NMR spectrum of **7’** (CD_2Cl_2, 500 MHz, top-full, bottom-zoomed in on aromatic region).
* indicates residual starting material

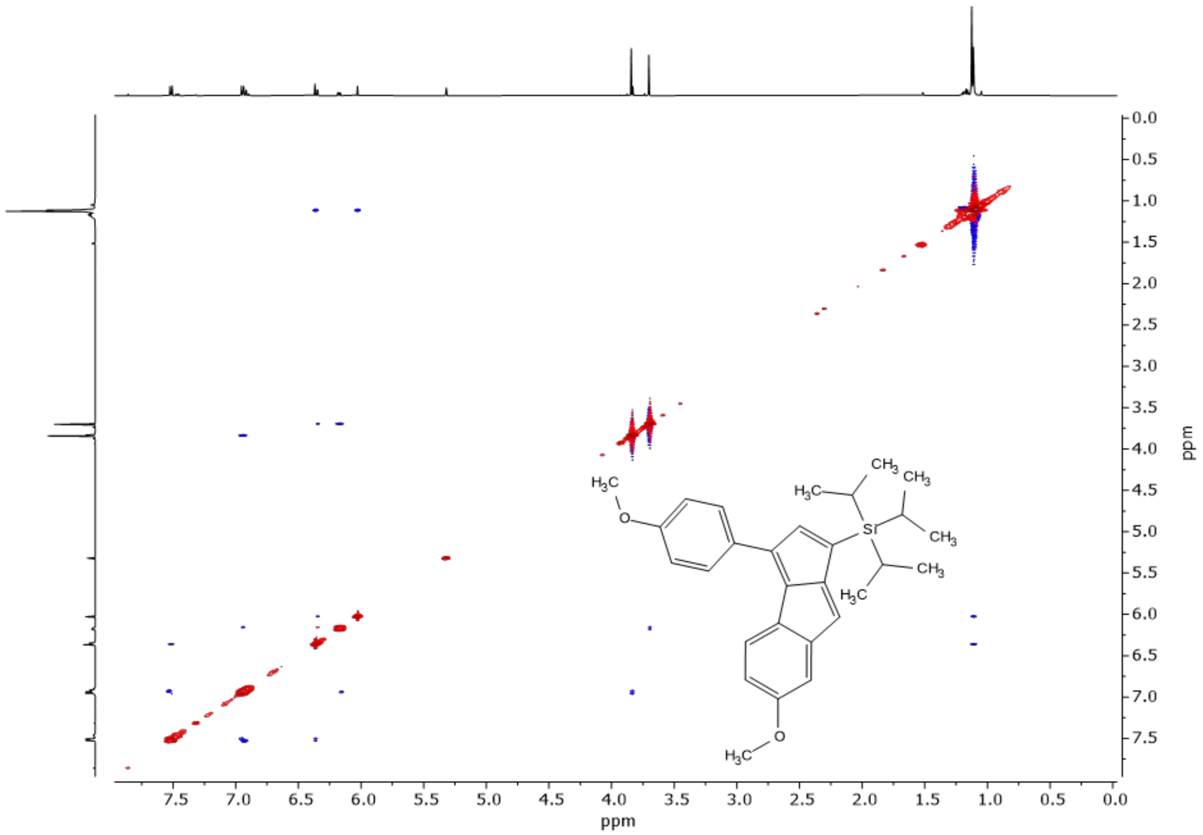

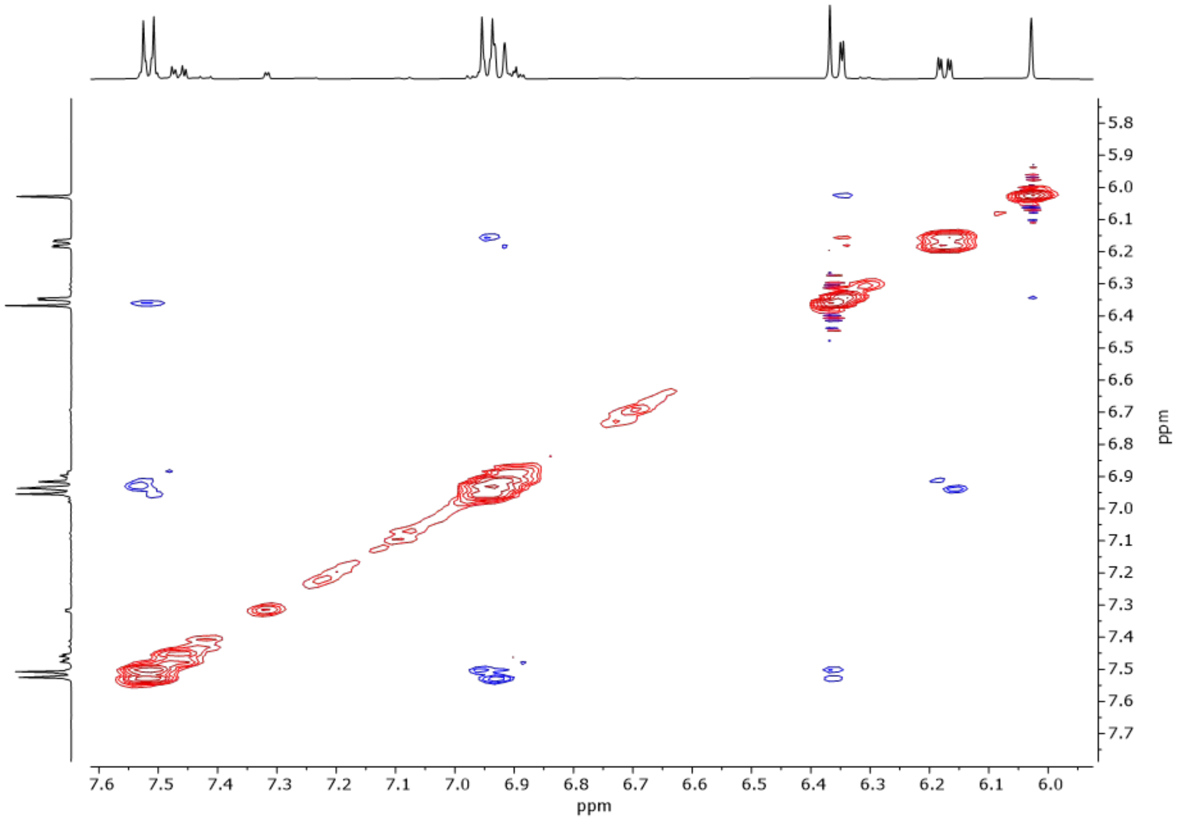

Figure S79. NOESY spectrum of **7'** (CD_2Cl_2, 300 MHz, top-full, bottom-zoomed in on aromatic region).

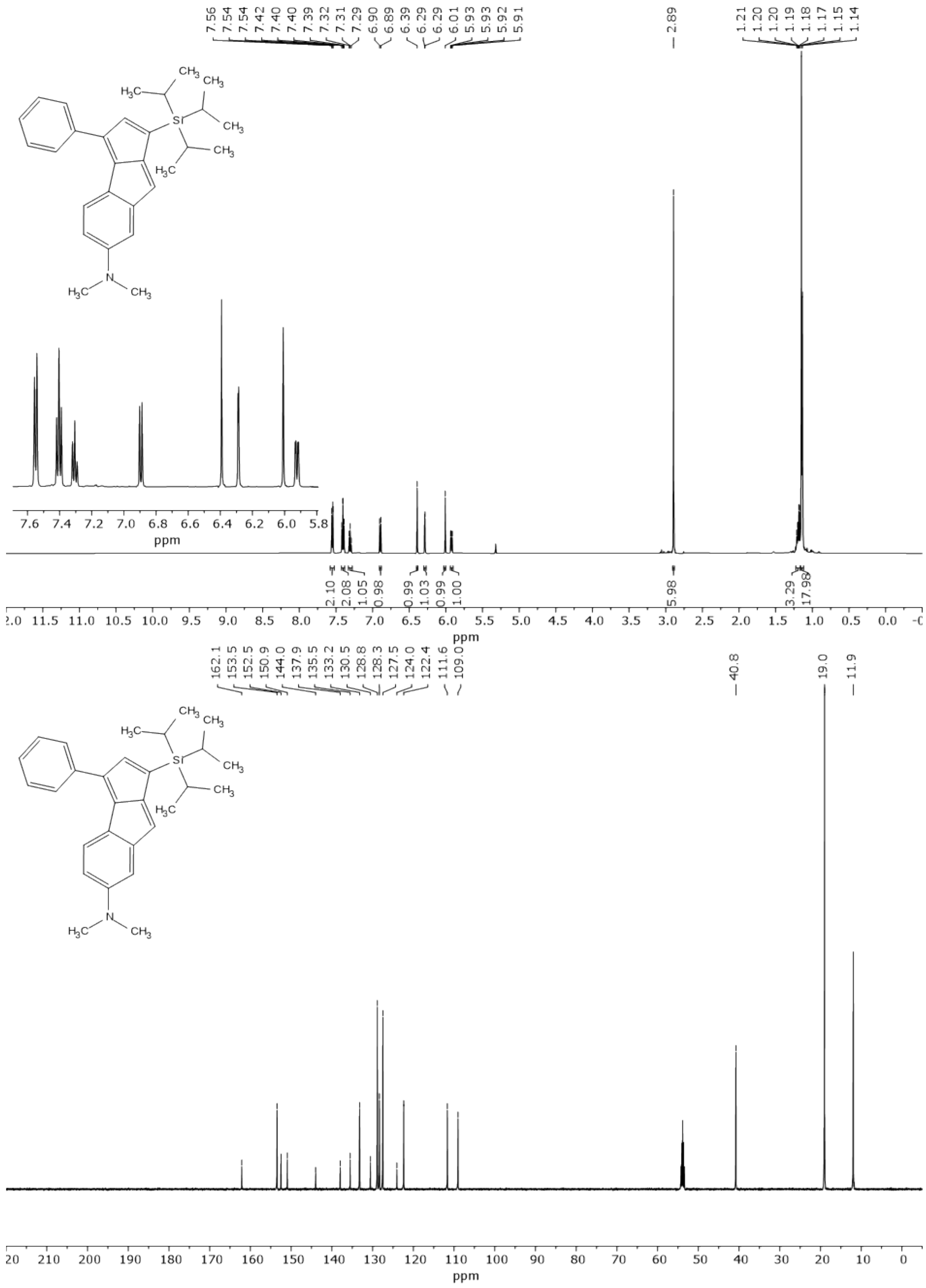

Figure S80. ^{1}H NMR spectrum (top) and $^{13}C\{^{1}H\}$ NMR spectrum (bottom) of **8** (CD_2Cl_2, 500 MHz).

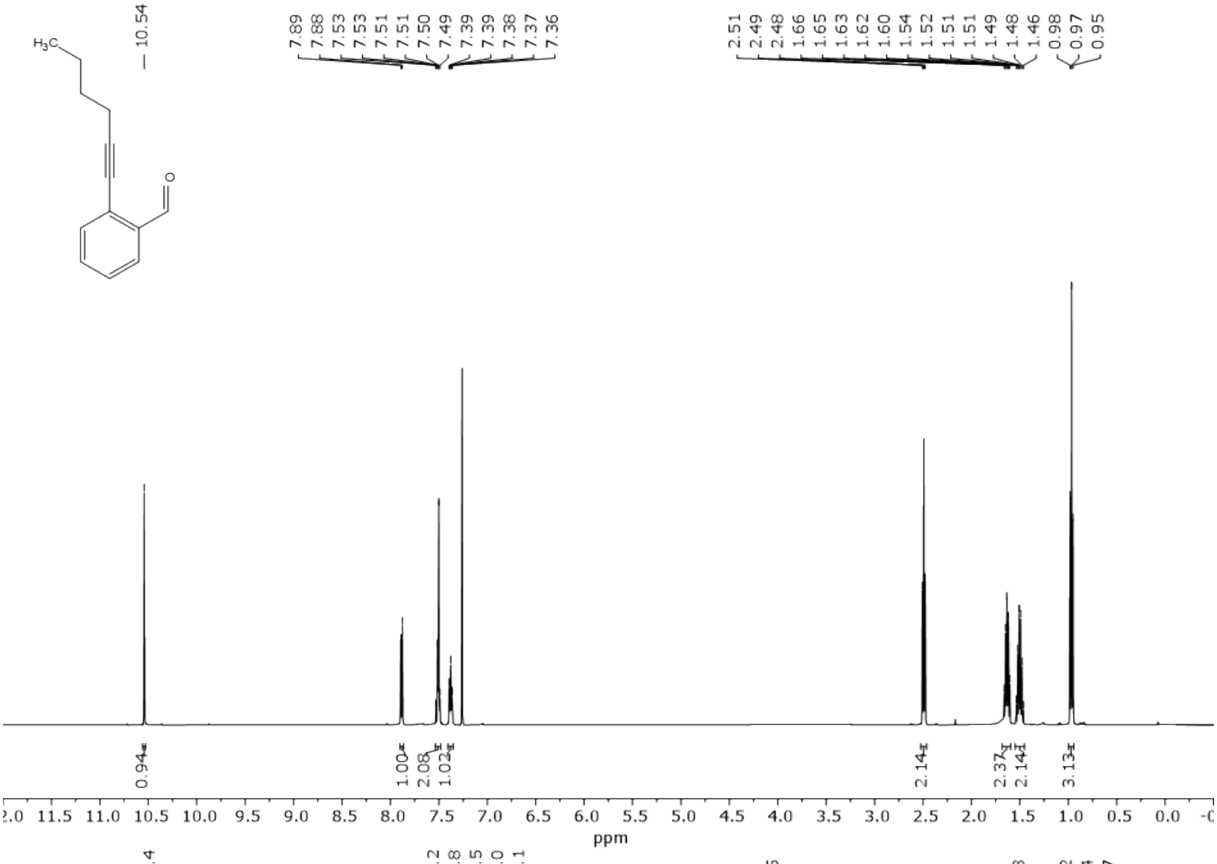

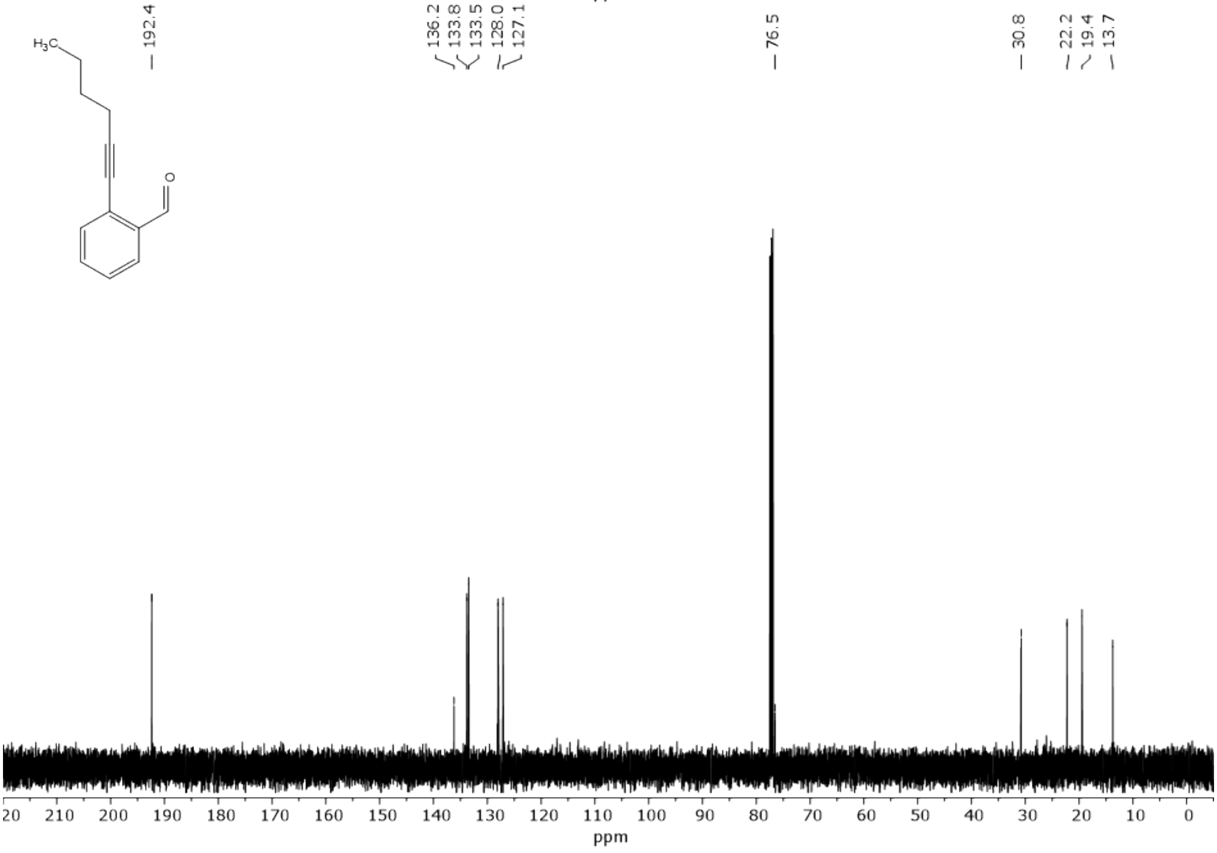

Figure S81. ^{1}H NMR spectrum (top) and ^{13}C{^{1}H} NMR spectra (bottom) of **S41** ($CDCl_3$, 500 MHz).

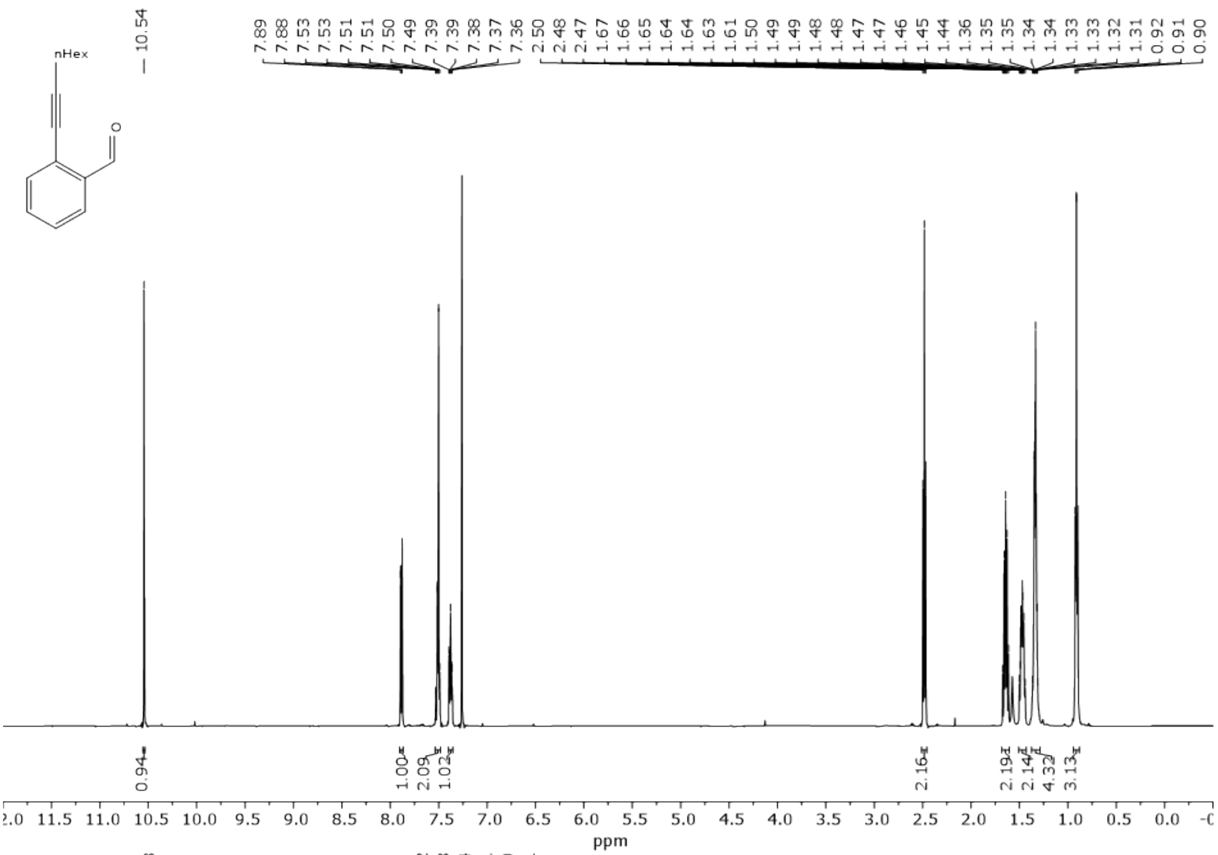

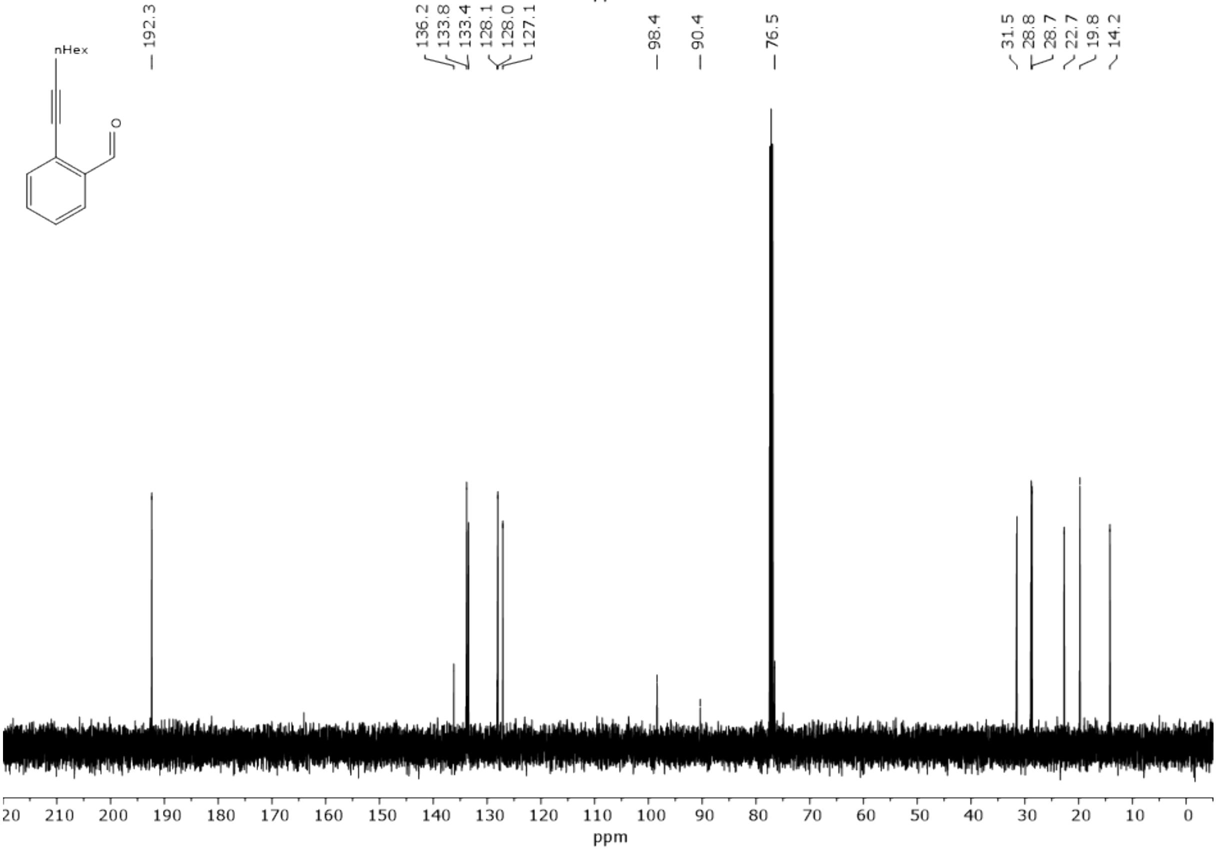

Figure S82. ^{1}H NMR spectrum (top) and $^{13}C\{^{1}H\}$ NMR spectra (bottom) of **S42** ($CDCl_3$, 500 MHz).

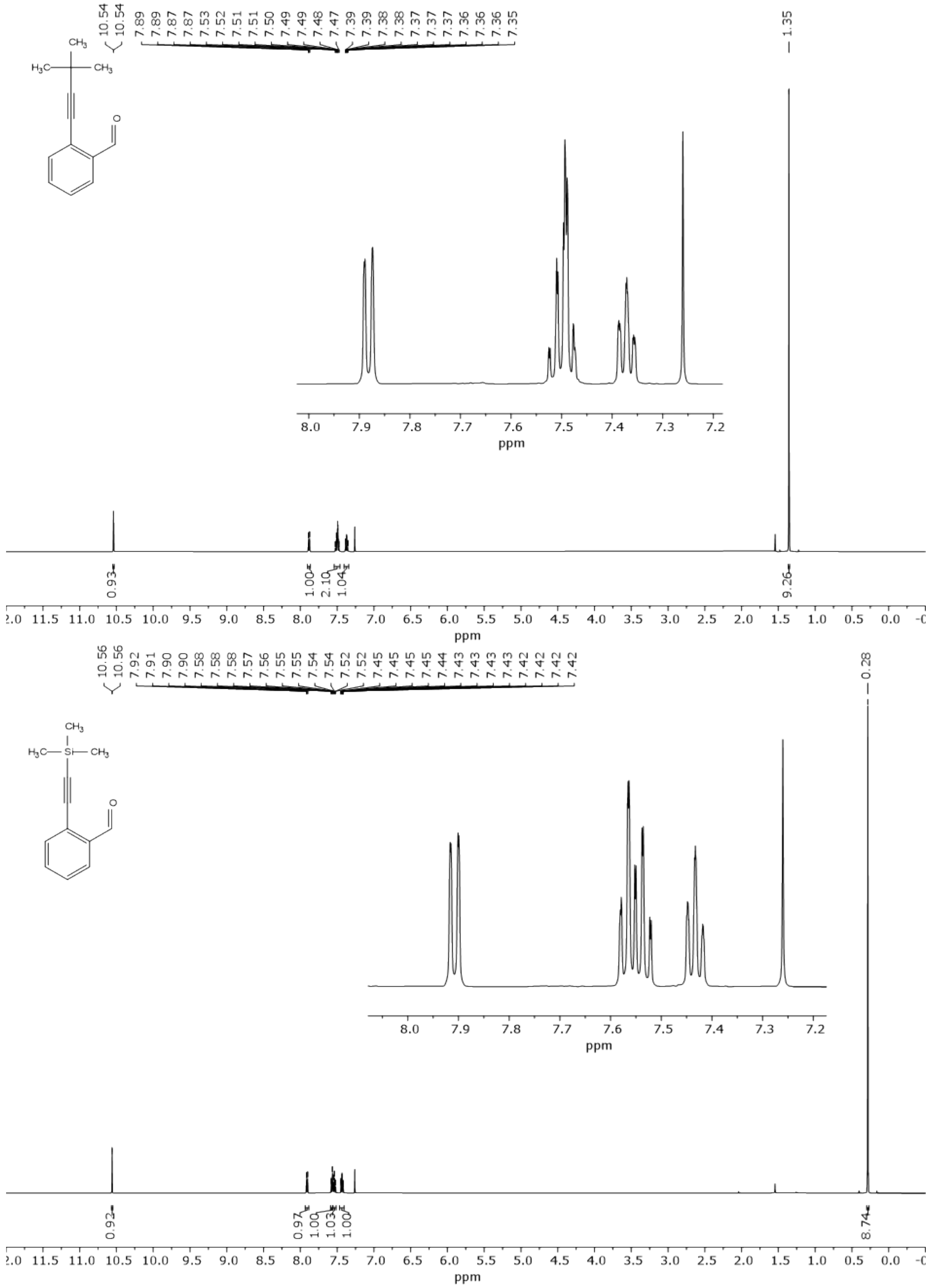

Figure S83. ^{1}H NMR spectra of **S43** (top, $CDCl_3$, 500 MHz) and **S44** (bottom, $CDCl_3$, 500 MHz).

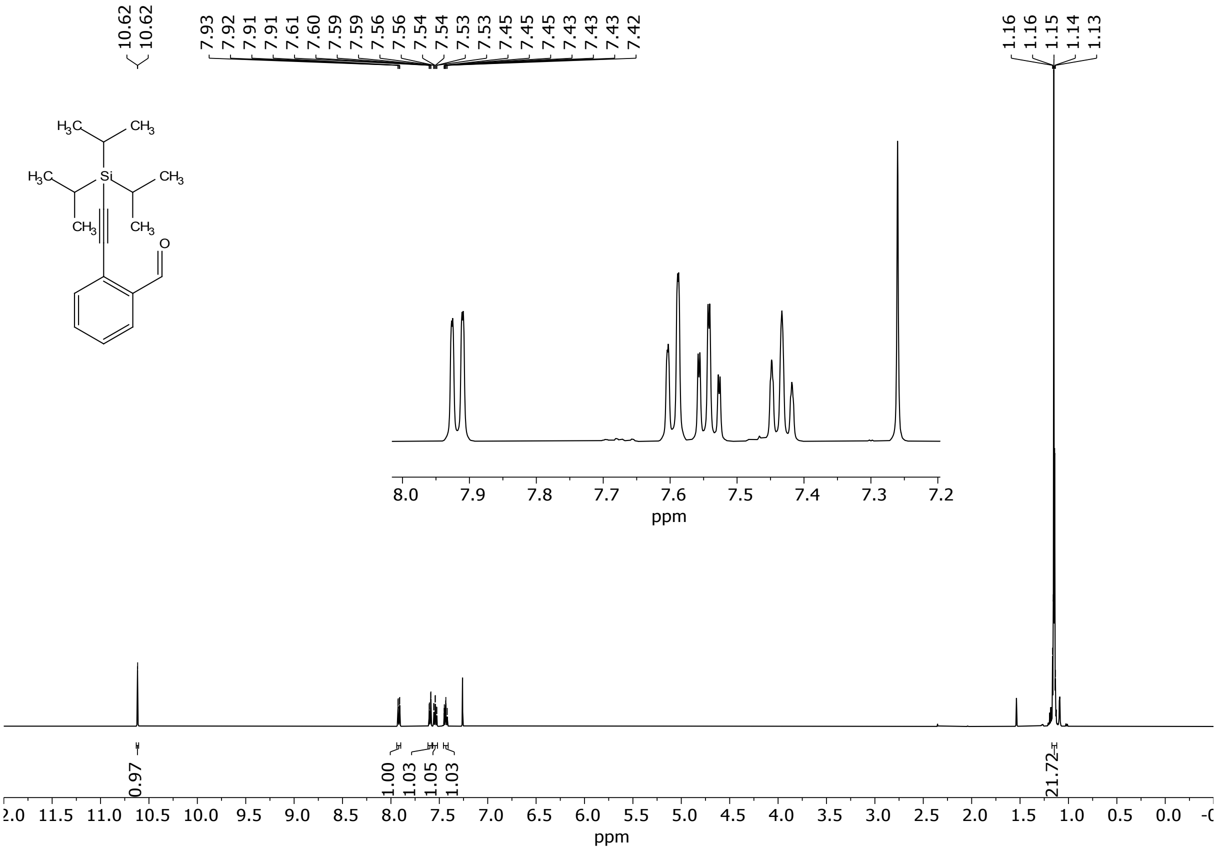

Figure S84. ^{1}H NMR spectra of **S45** ($CDCl_3$, 500 MHz).

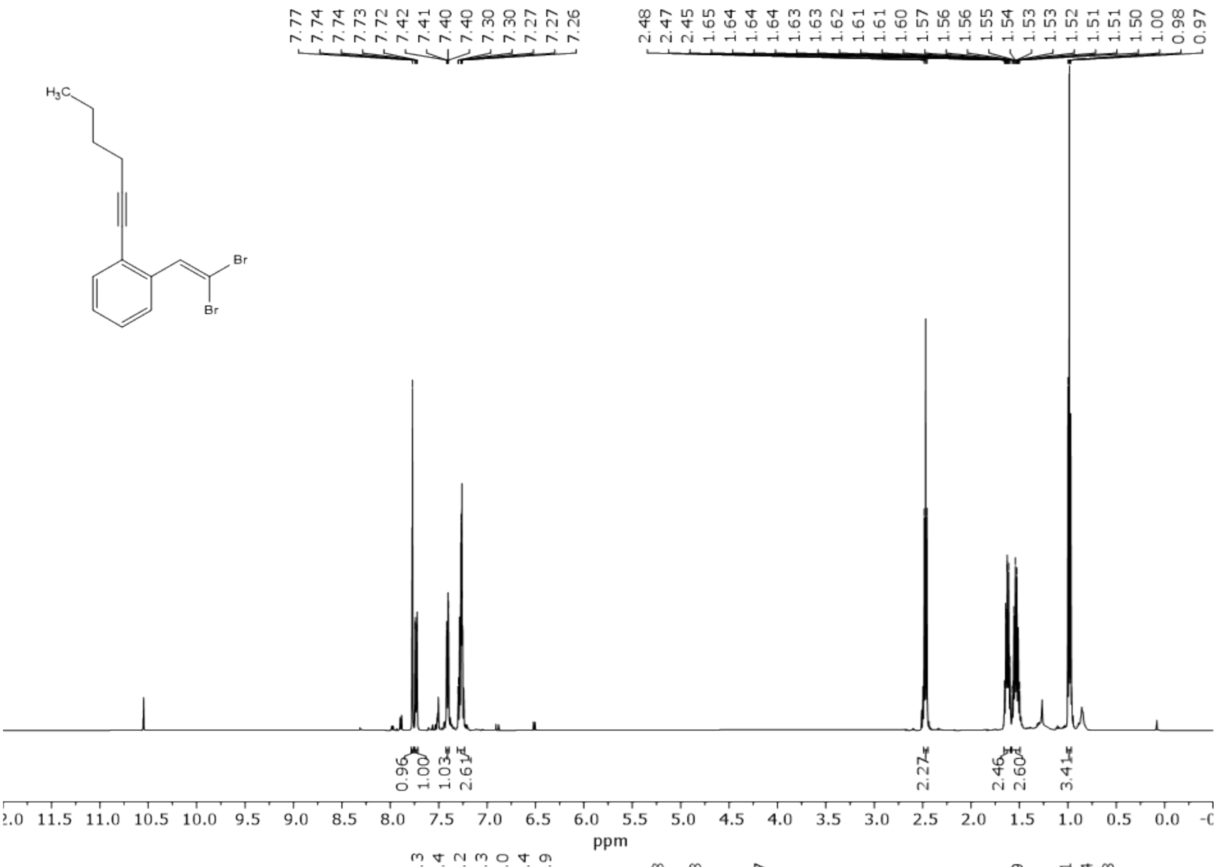

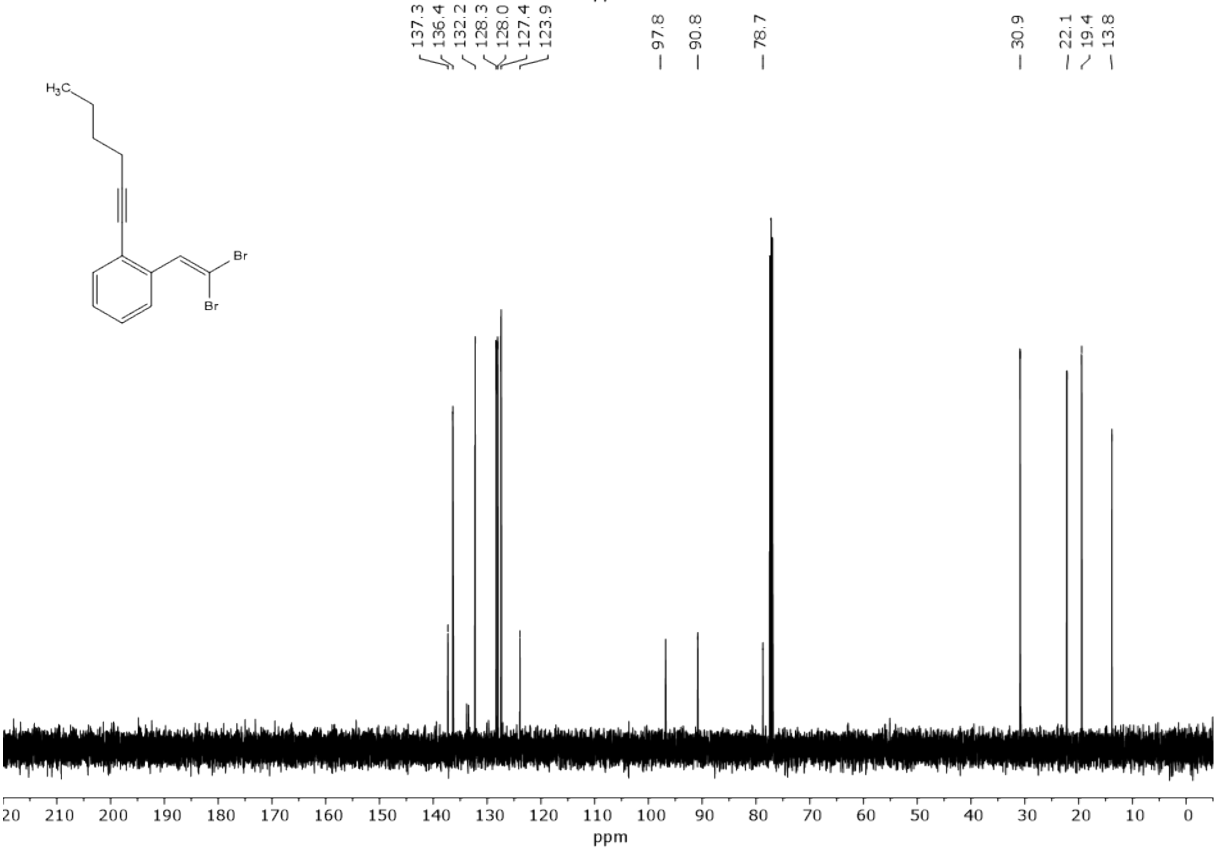

Figure S85. ^{1}H NMR spectrum (top) and ^{13}C{^{1}H} NMR spectra (bottom) of **S46** ($CDCl_3$, 500 MHz).

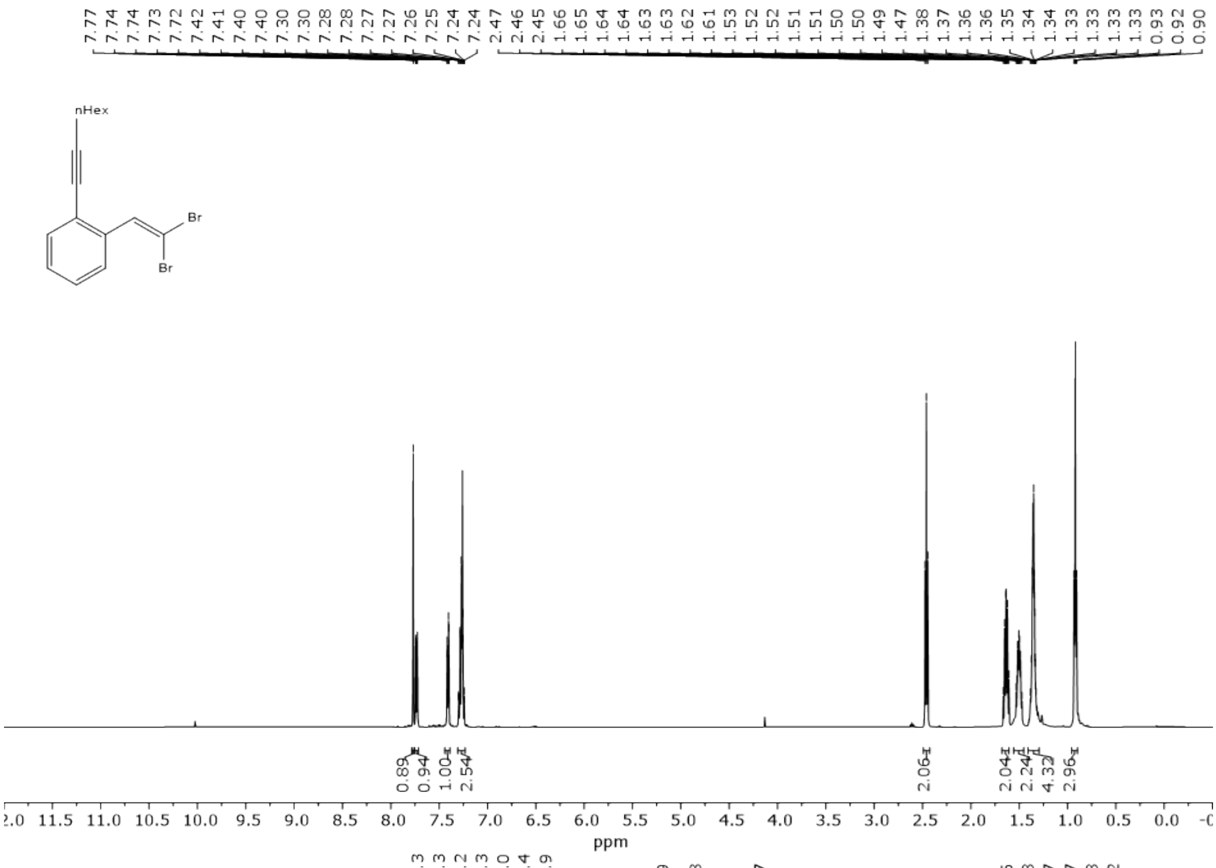

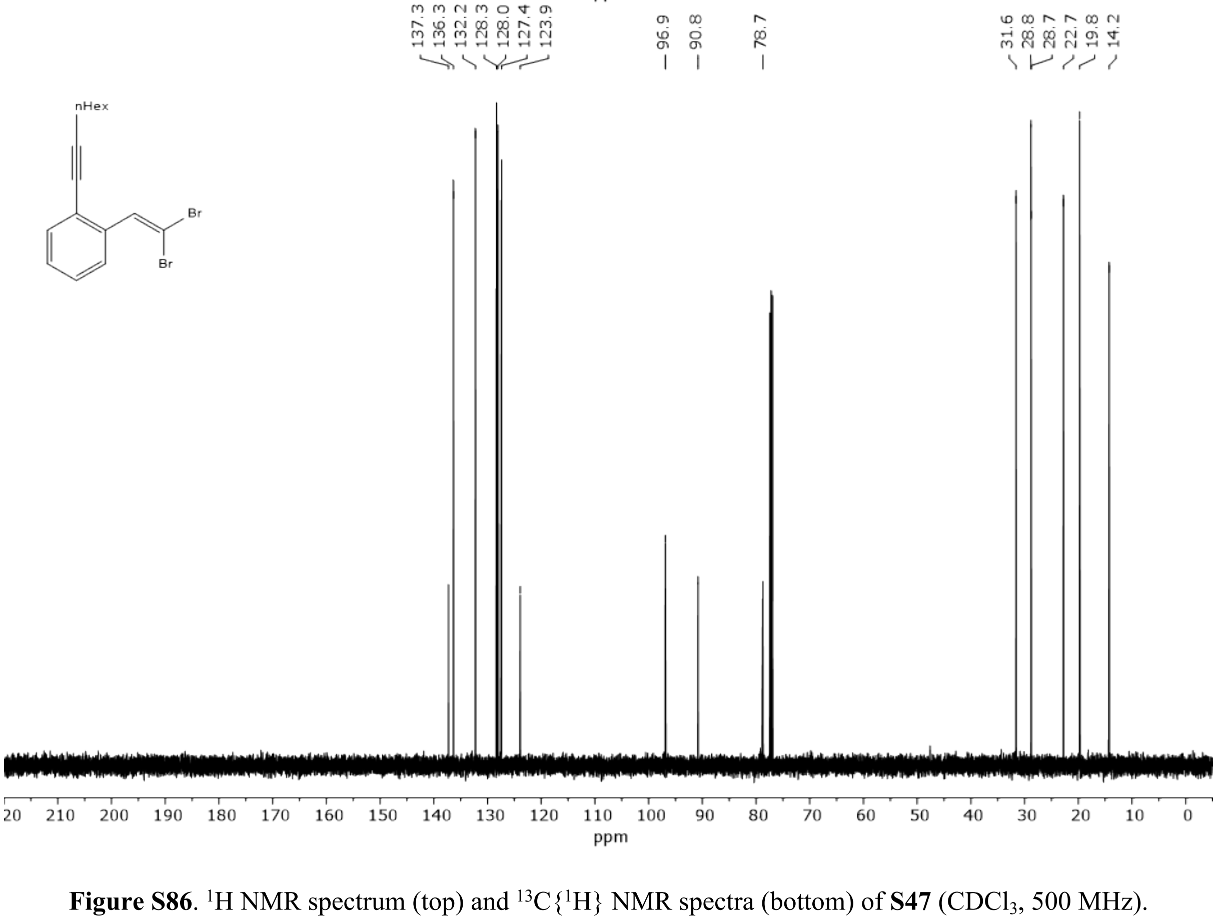

Figure S86. ^{1}H NMR spectrum (top) and $^{13}C\{^{1}H\}$ NMR spectra (bottom) of **S47** ($CDCl_3$, 500 MHz).

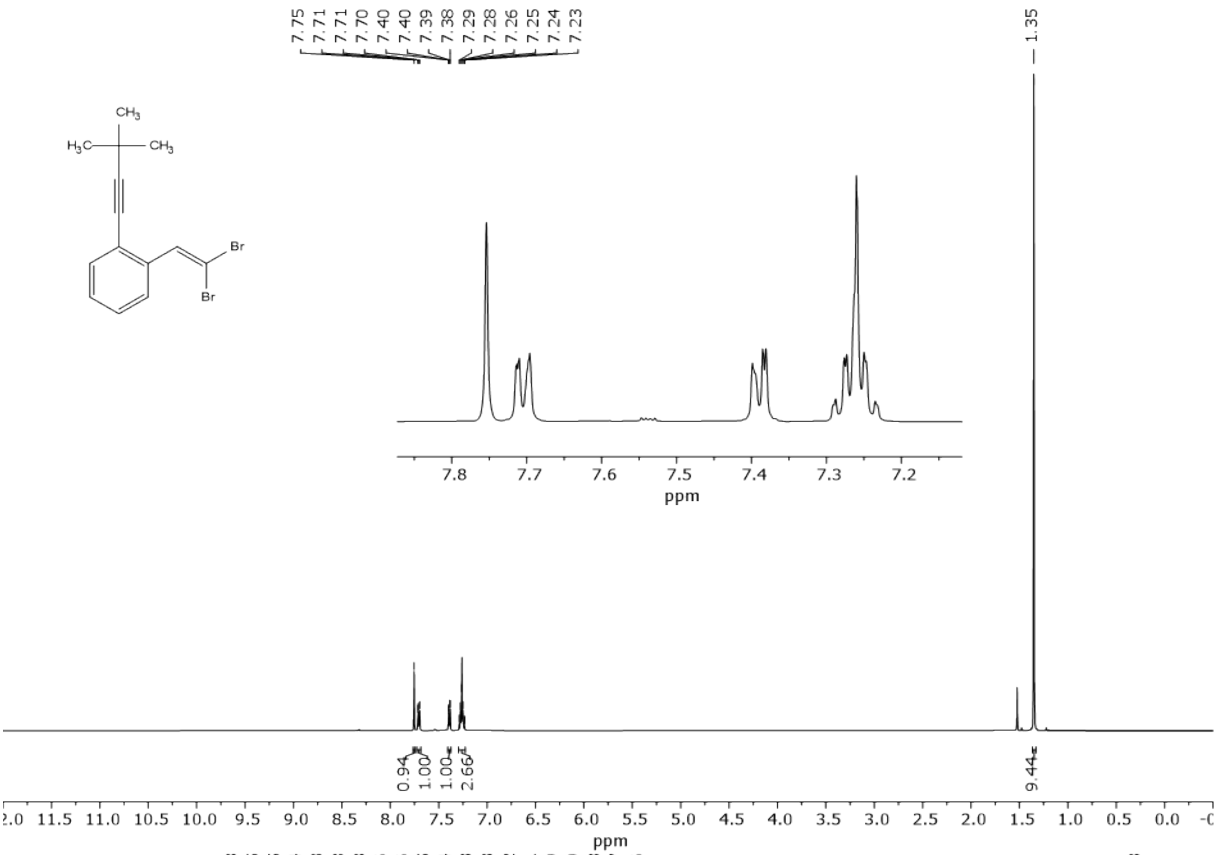

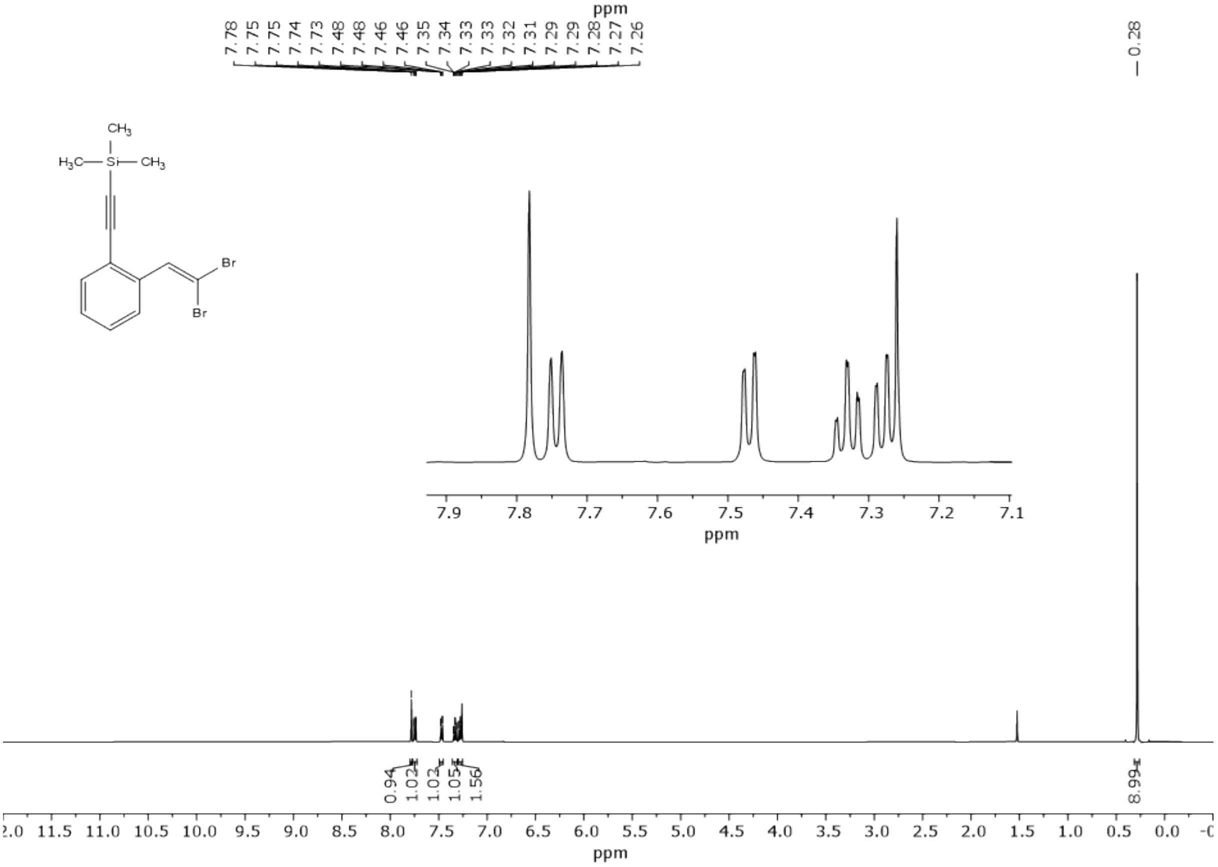

Figure S87. ^{1}H NMR spectra of **S48** (top, $CDCl_3$, 500 MHz) and **S49** (bottom, $CDCl_3$, 500 MHz).

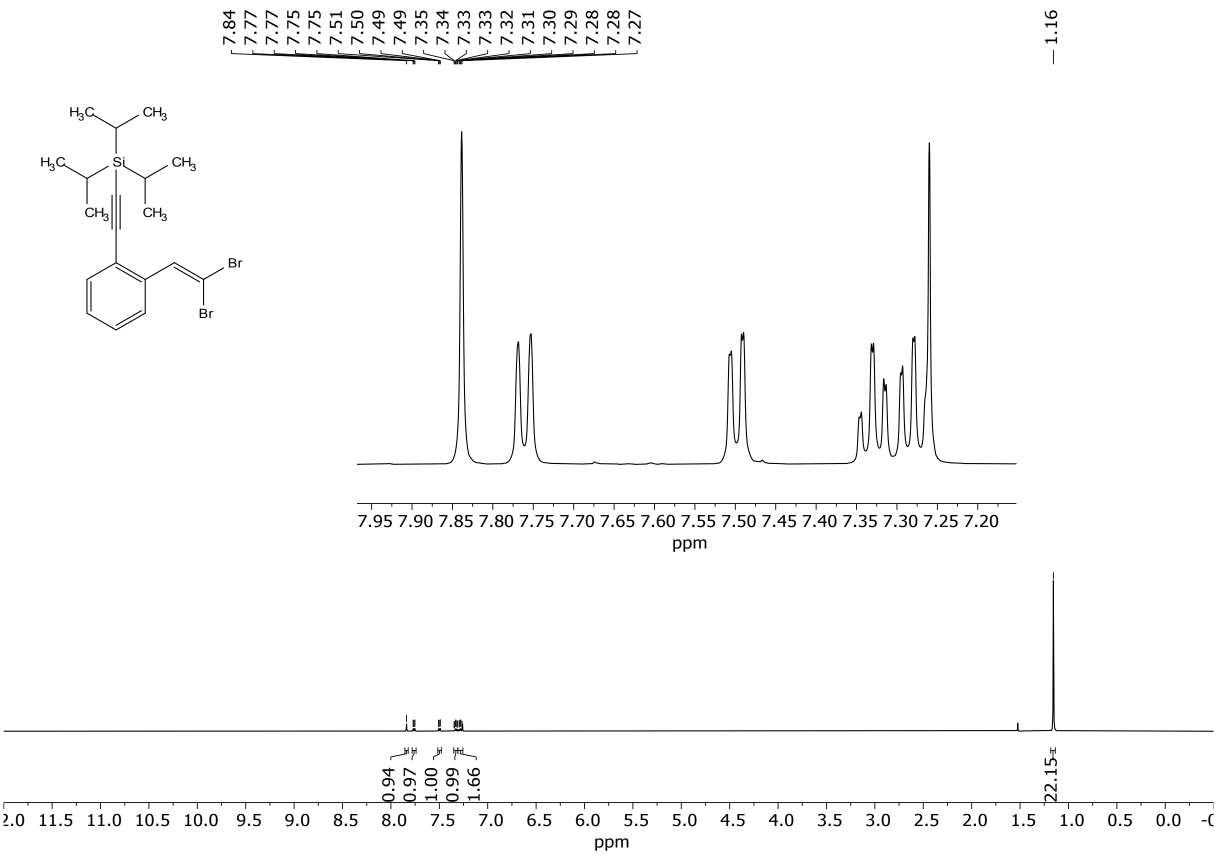

Figure S88. ^{1}H NMR spectra of **S50** ($CDCl_3$, 500 MHz).

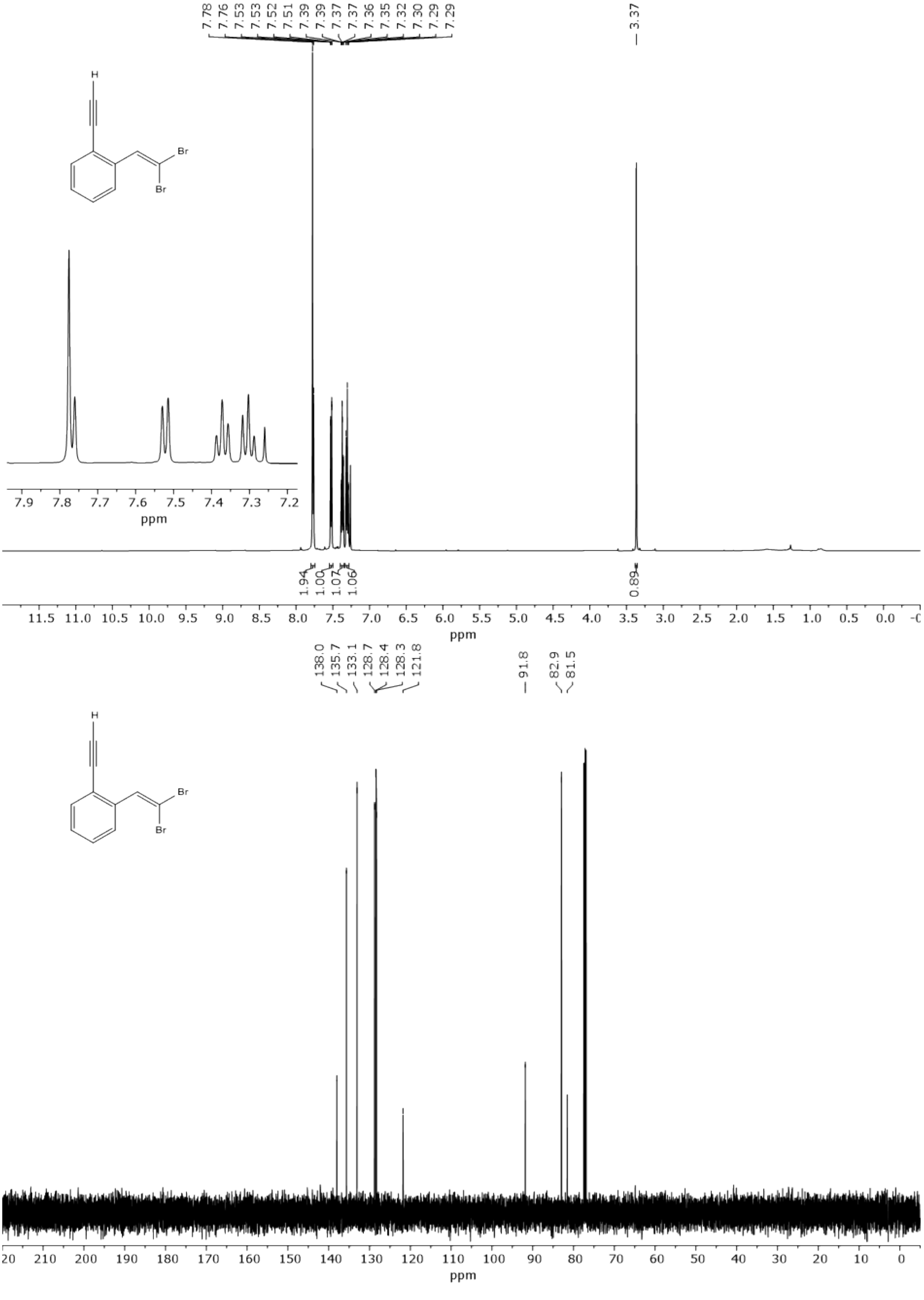

Figure S89. ^{1}H NMR spectrum (top) and $^{13}C\{^1H\}$ NMR spectra (bottom) of **S51** ($CDCl_3$, 500 MHz).

S4 HRMS spectra

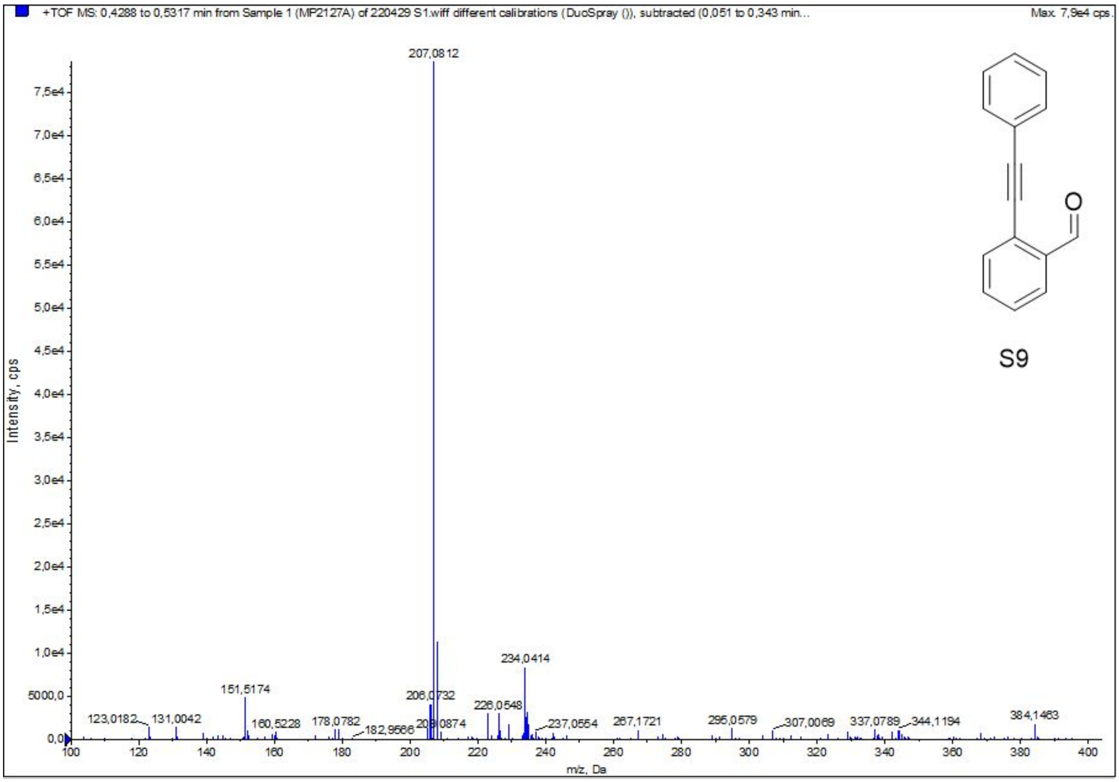

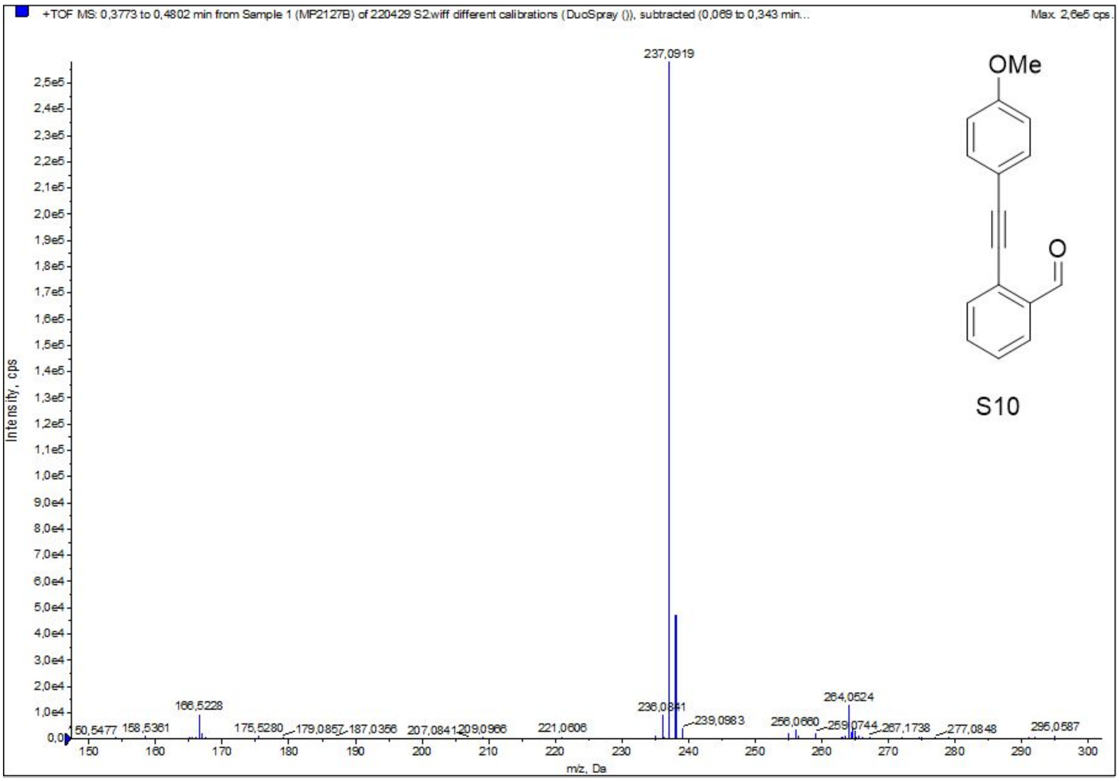

Figure S90. HRMS spectra of **S9** (top) and **S10** (bottom).

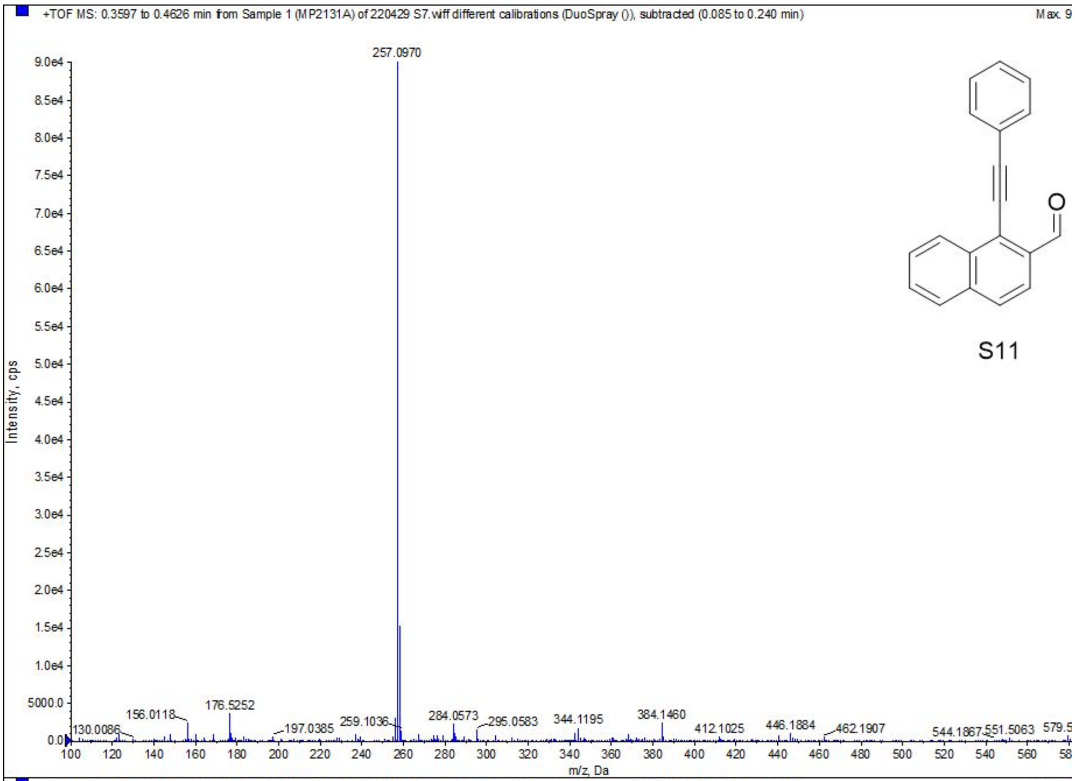

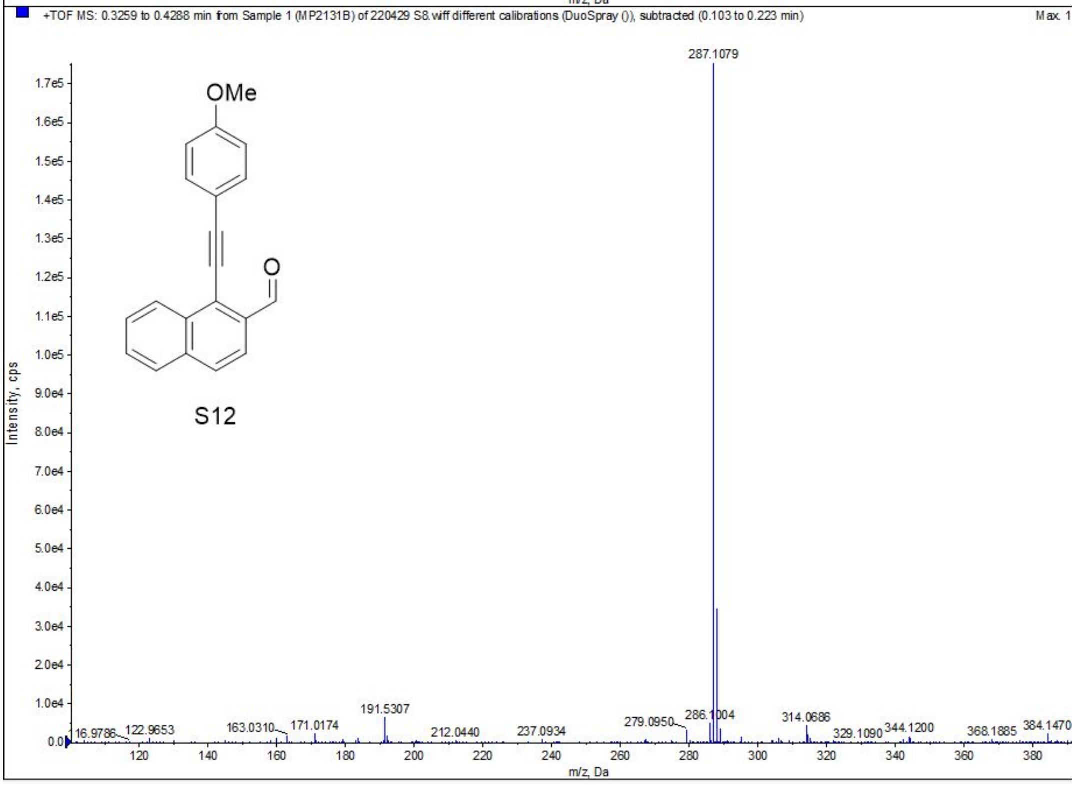

Figure S91. HRMS spectra of **S11** (top) and **S12** (bottom).

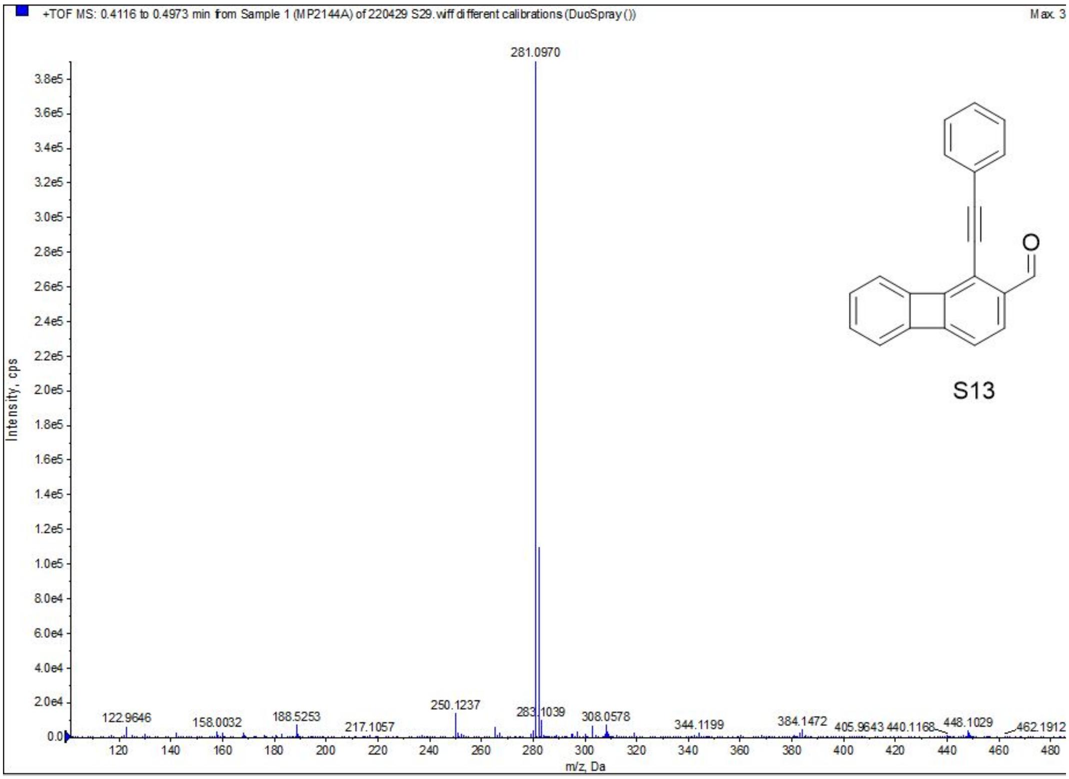

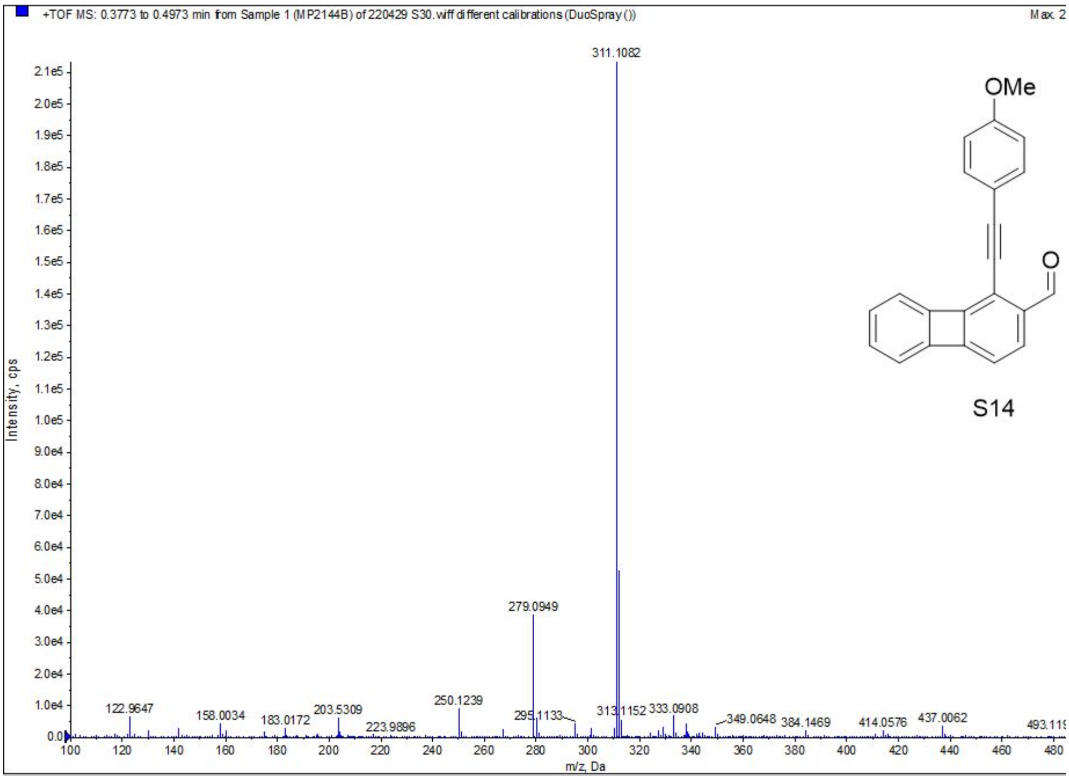

Figure S92. HRMS spectra of **S13** (top) and **S14** (bottom).

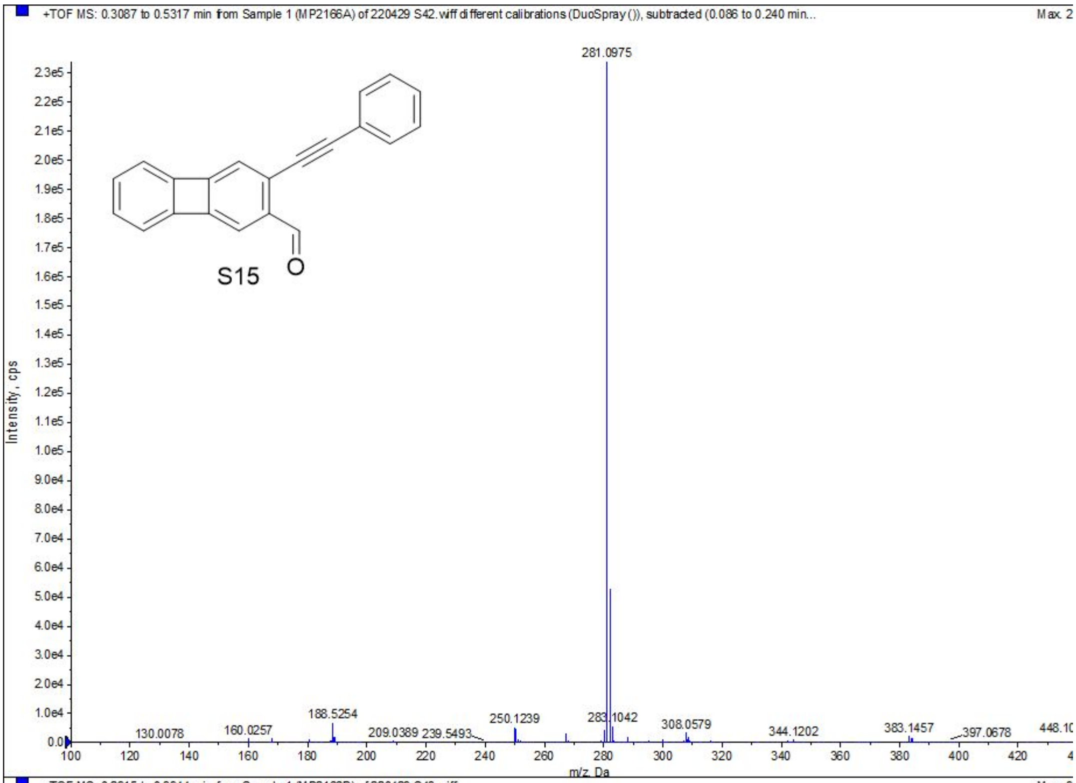

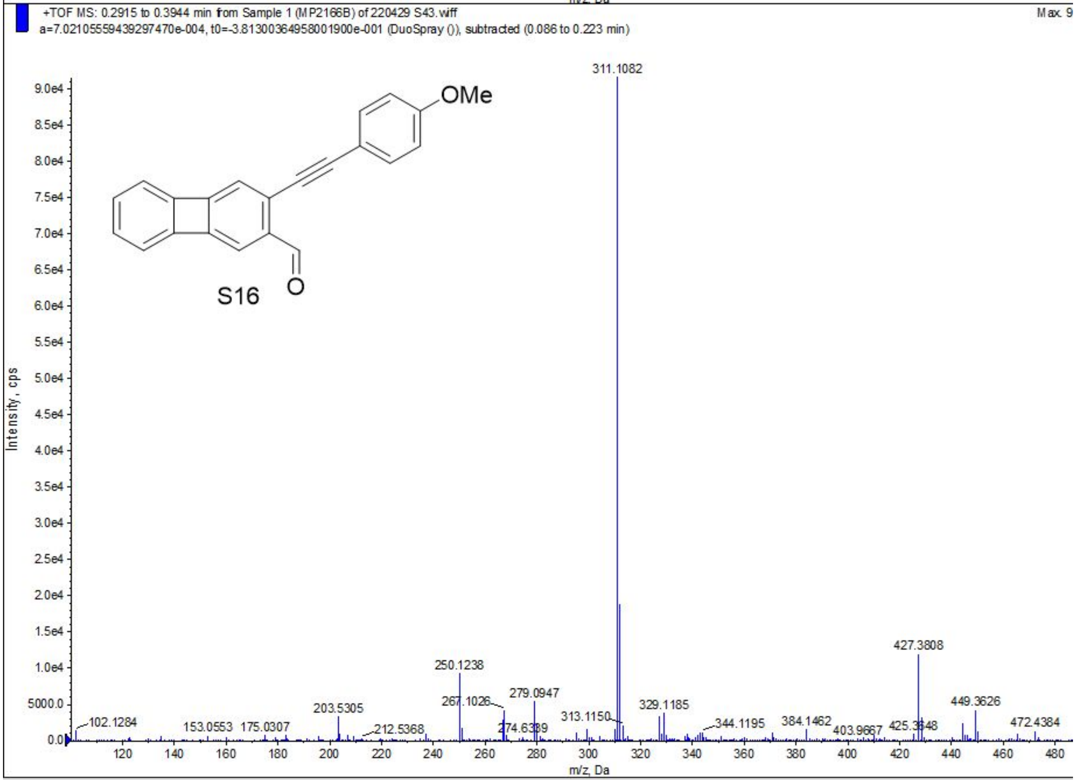

Figure S93. HRMS spectra of **S15** (top) and **S16** (bottom).

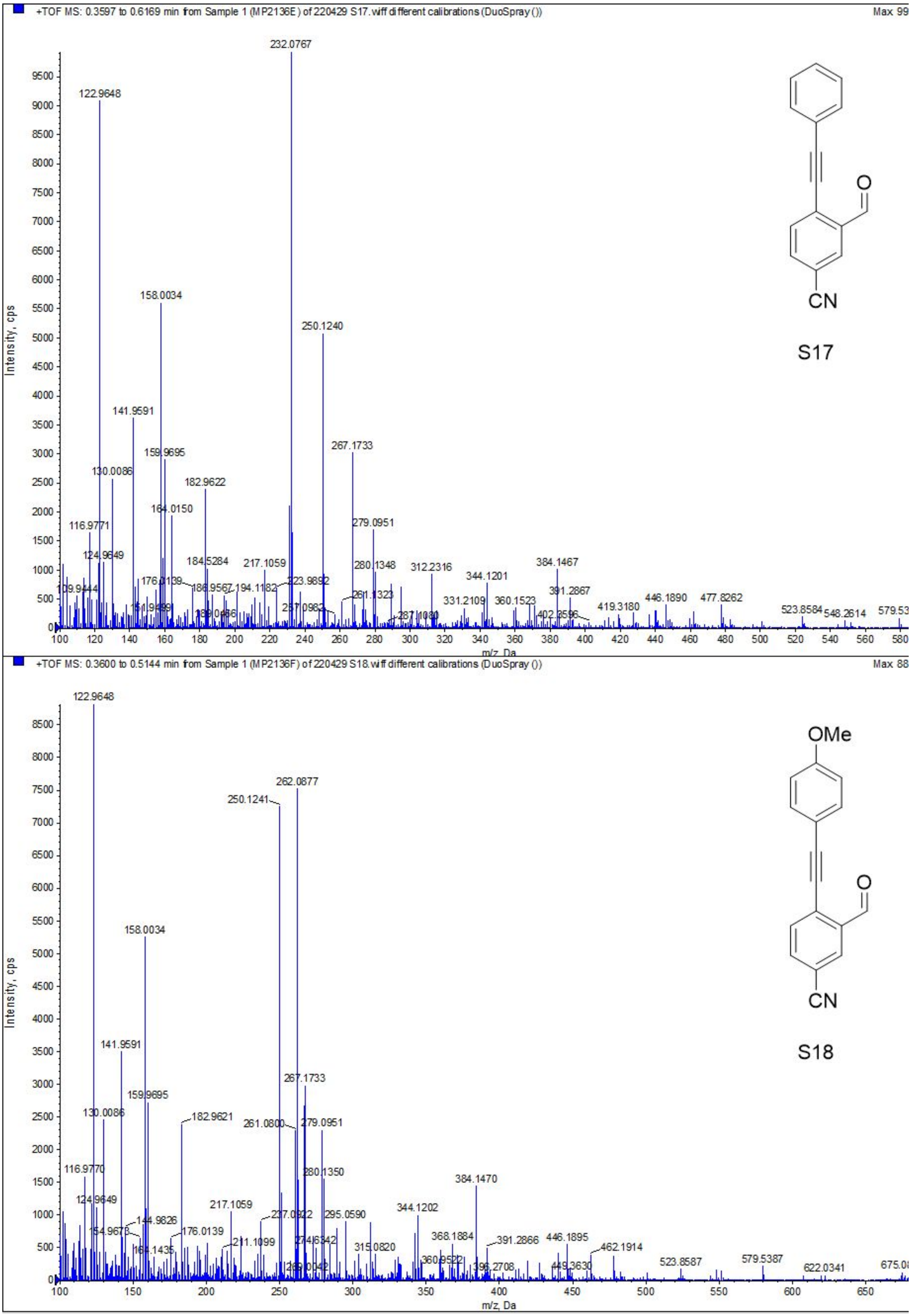

Figure S94. HRMS spectra of **S17** (top) and **S18** (bottom).

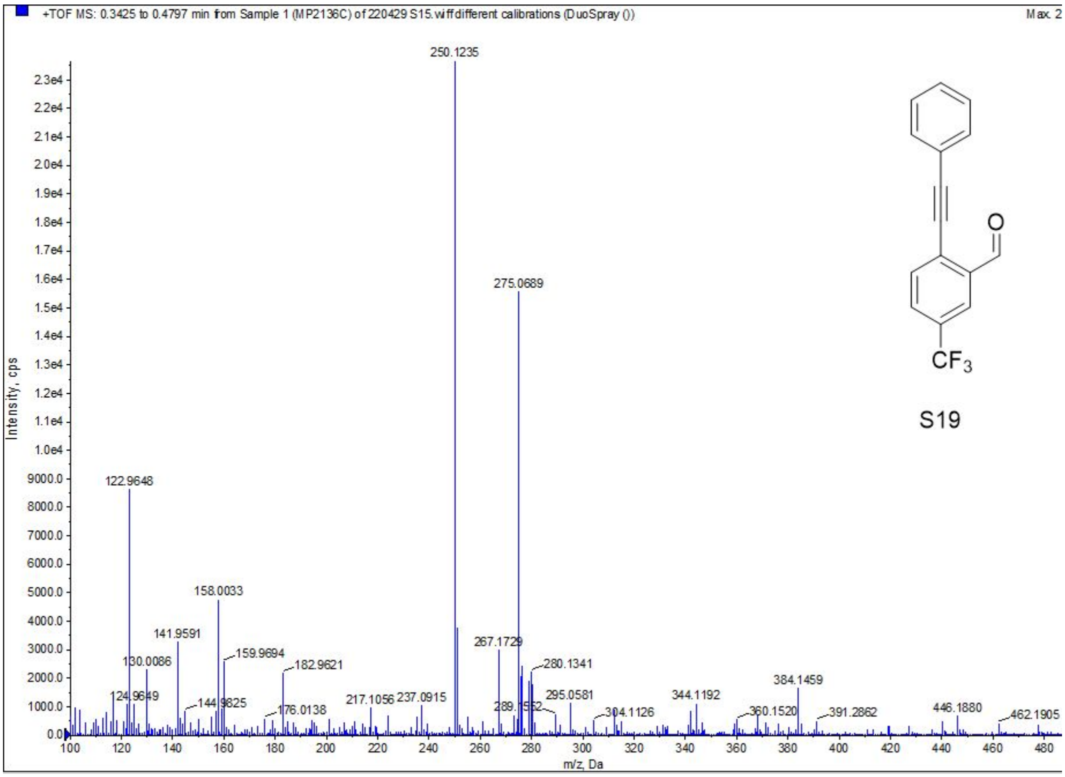

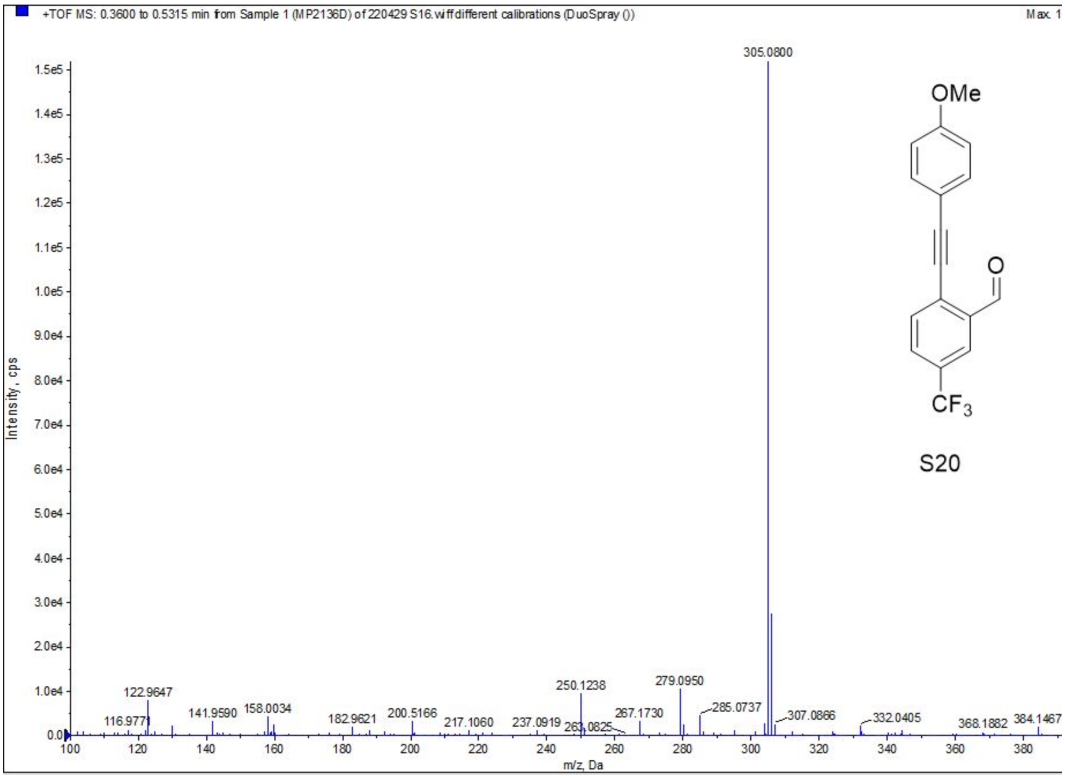

Figure S95. HRMS spectra of **S19** (top) and **S20** (bottom).

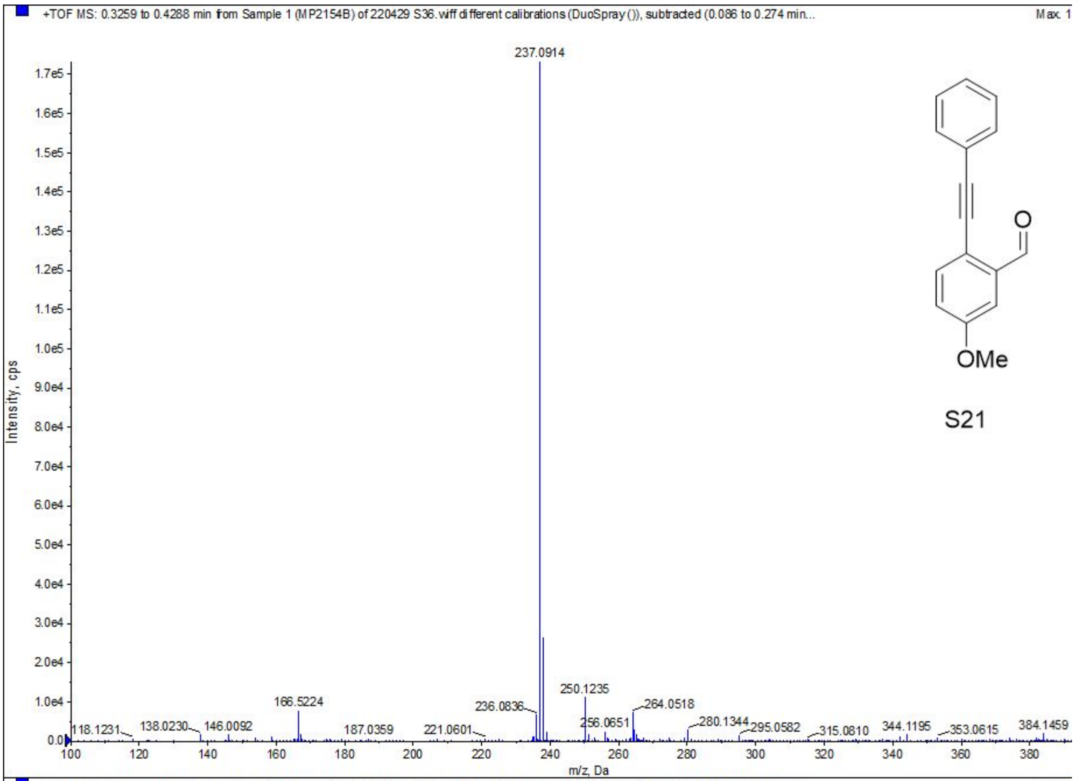

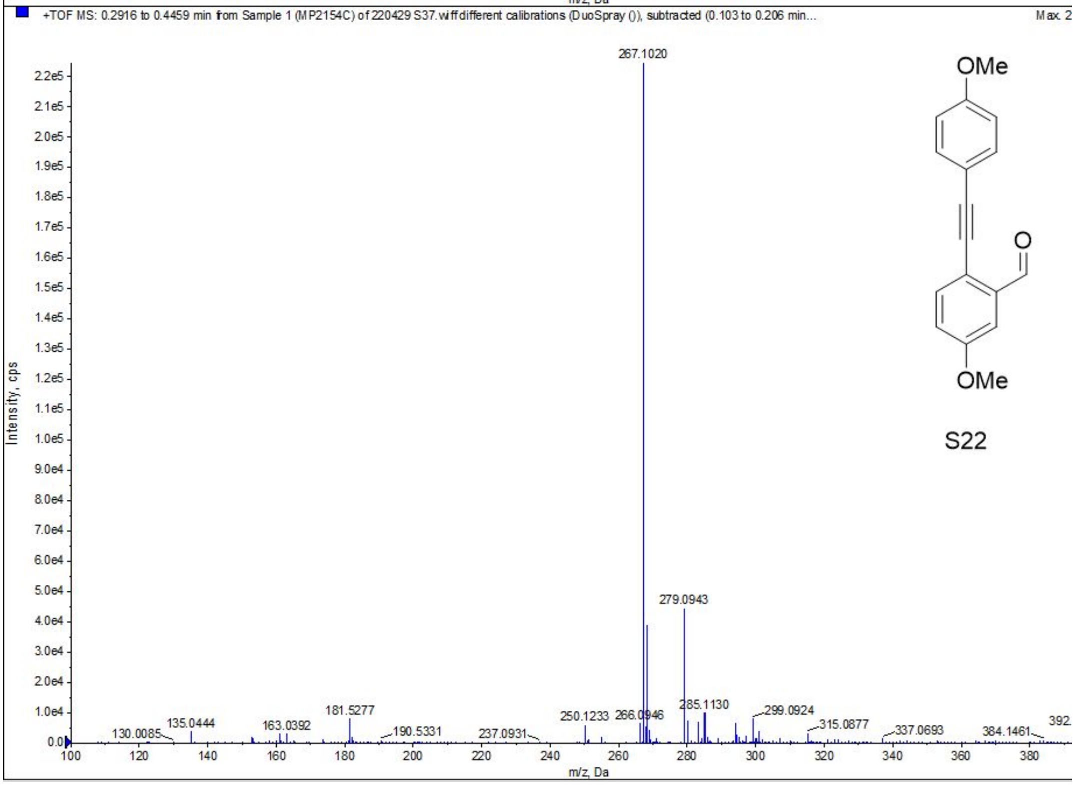

Figure S96. HRMS spectra of **S21** (top) and **S22** (bottom).

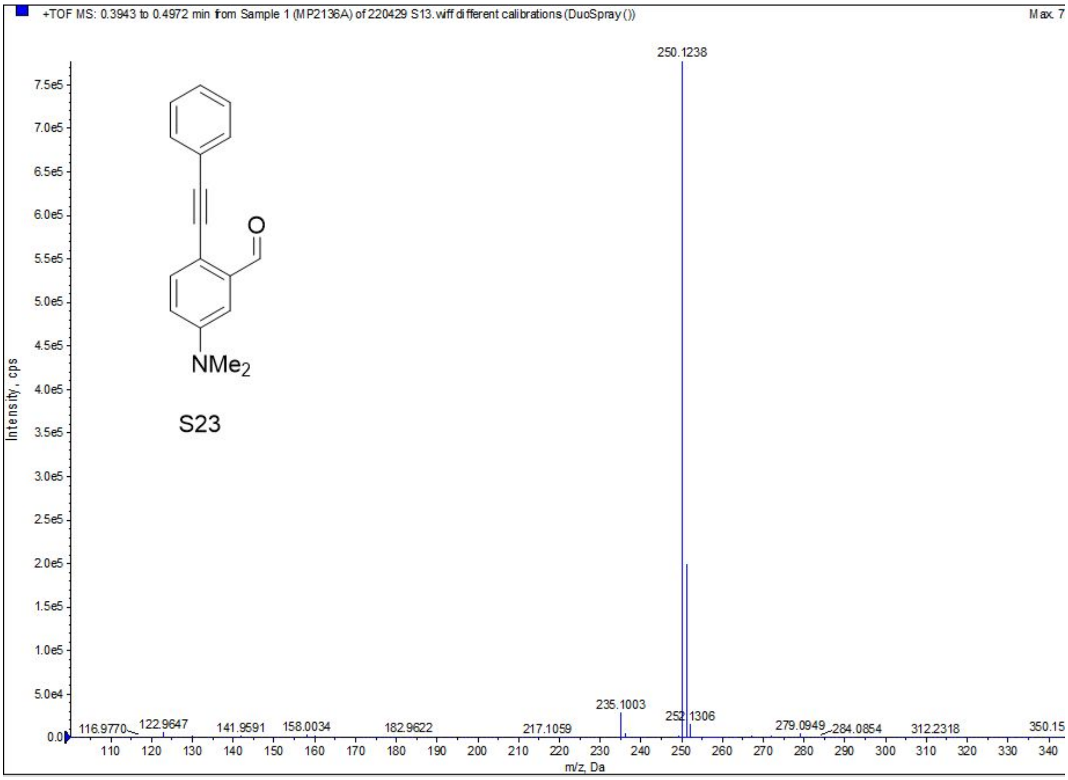

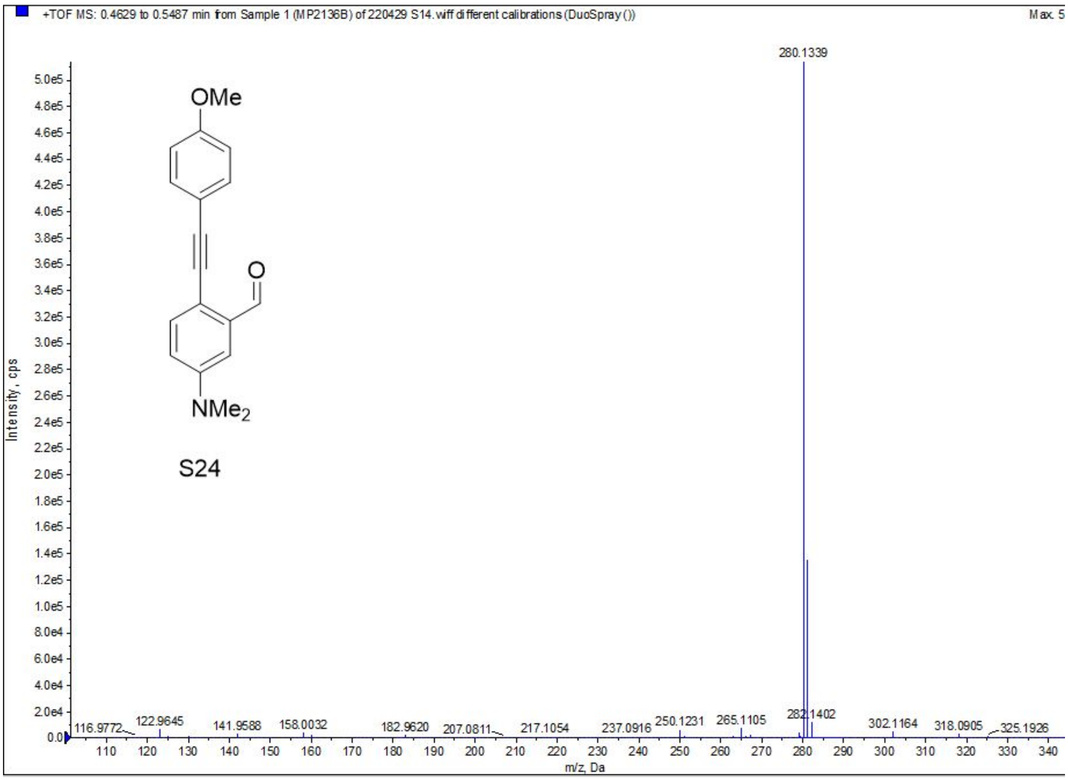

Figure S97. HRMS spectra of **S23** (top) and **S24** (bottom).

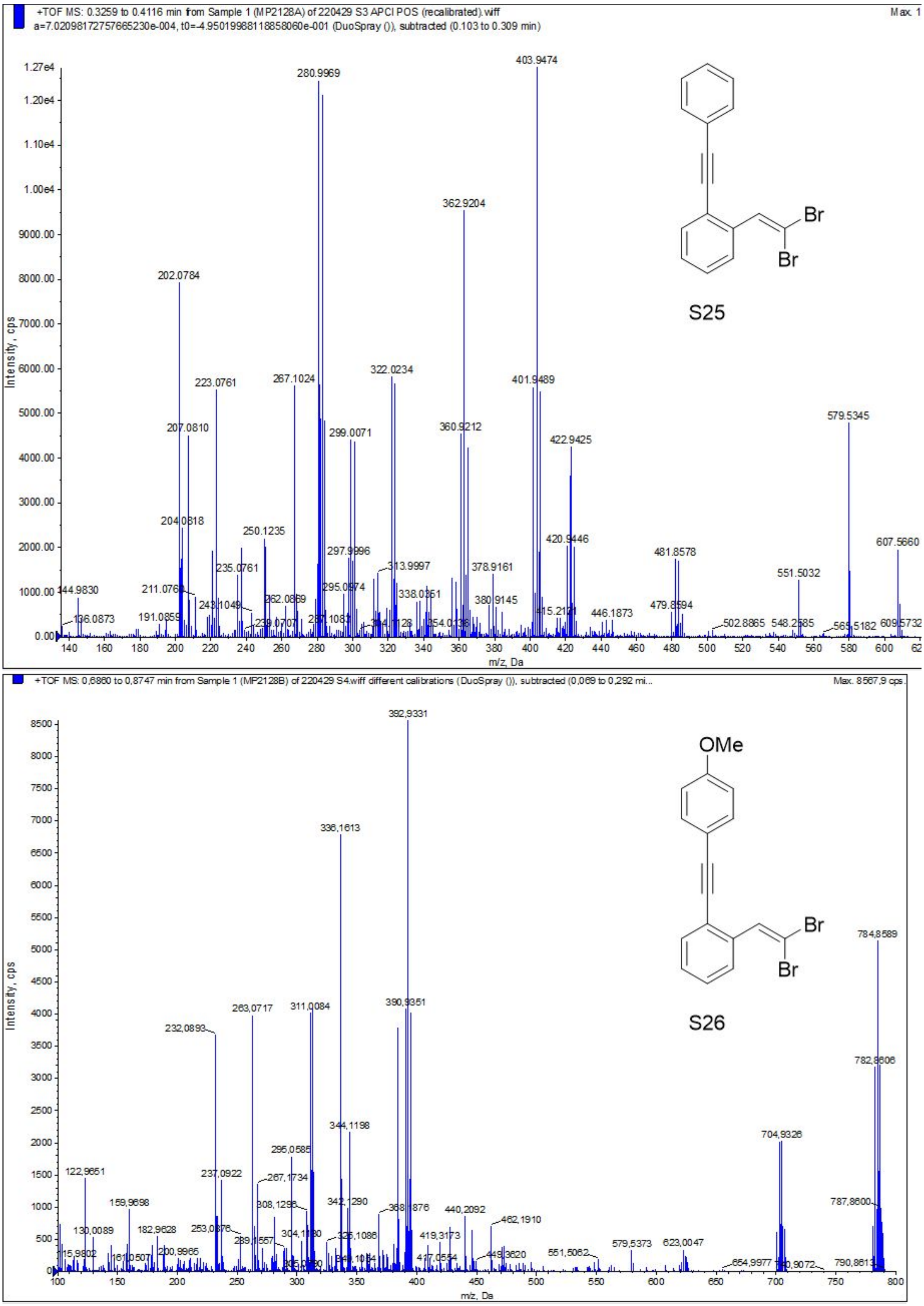

Figure S98. HRMS spectra of **S25** (top) and **S26** (bottom).

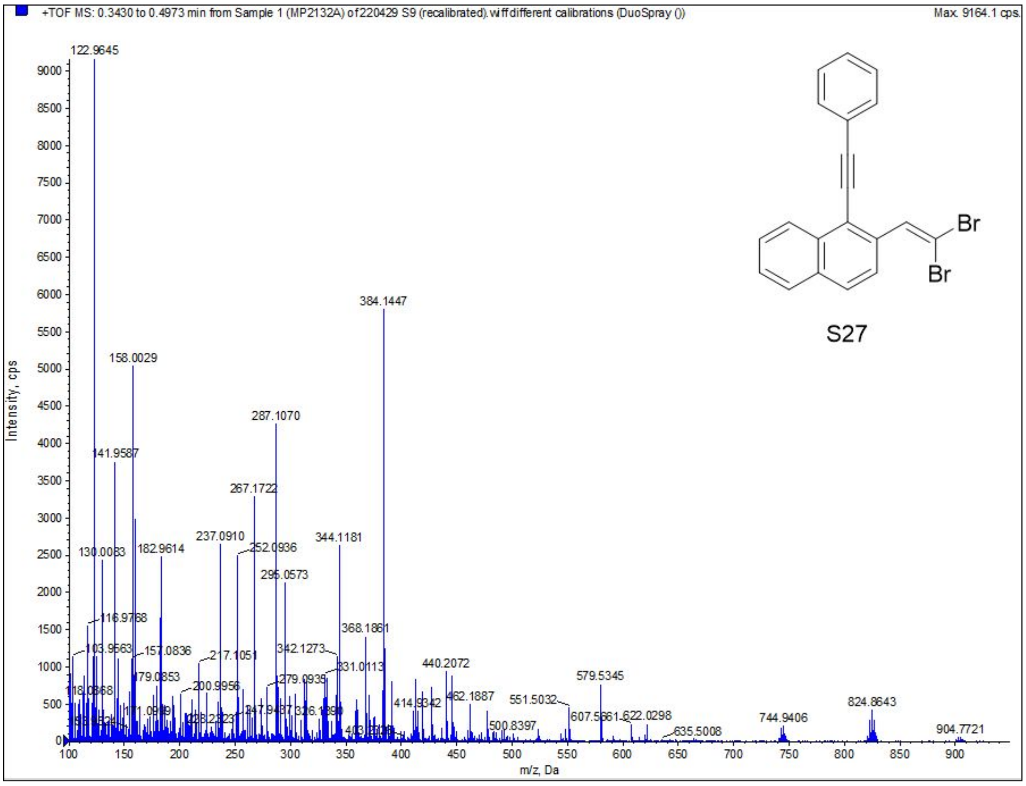

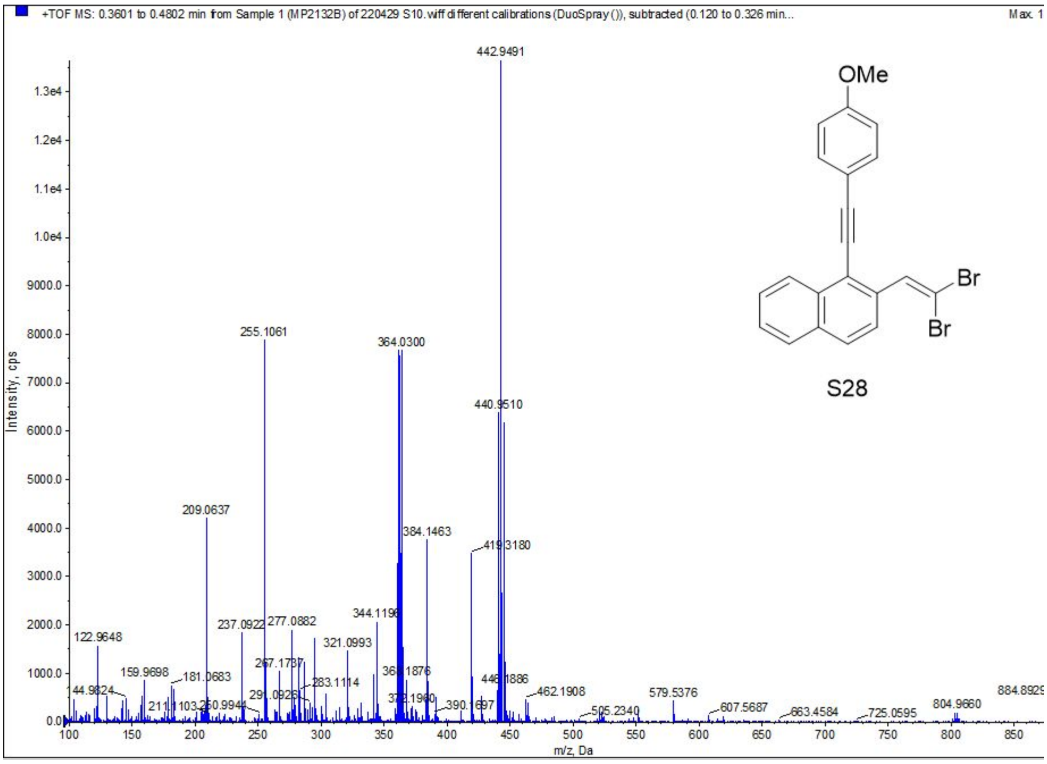

Figure S99. HRMS spectra of **S27** (top) and **S28** (bottom).

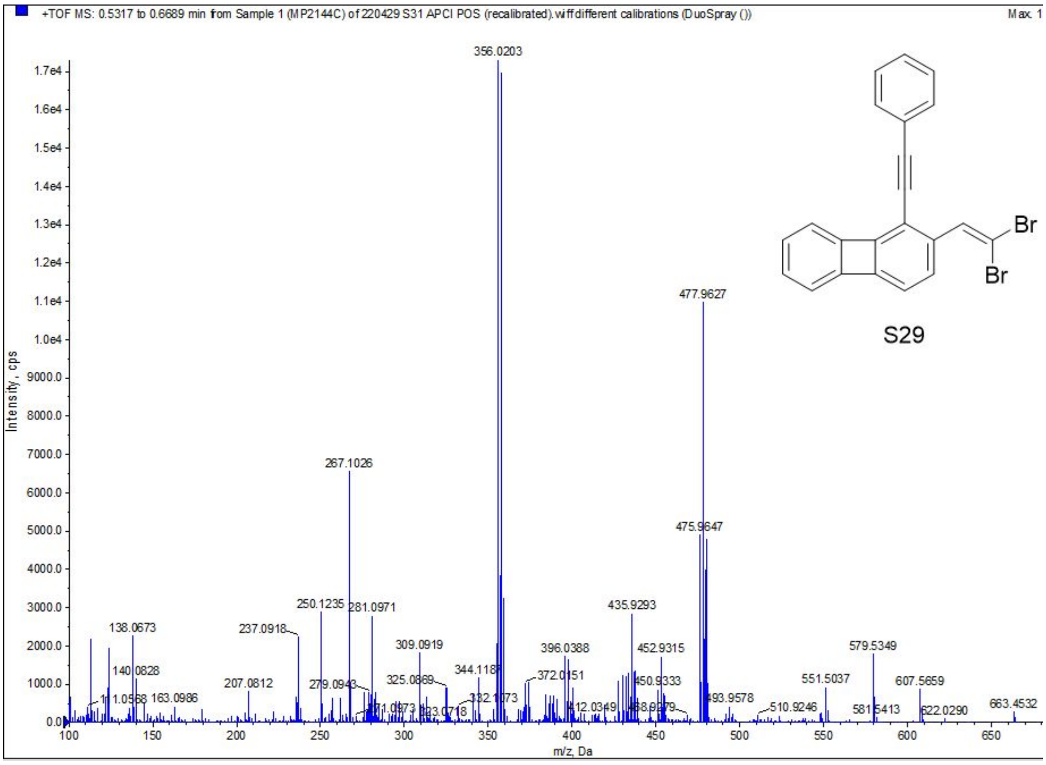

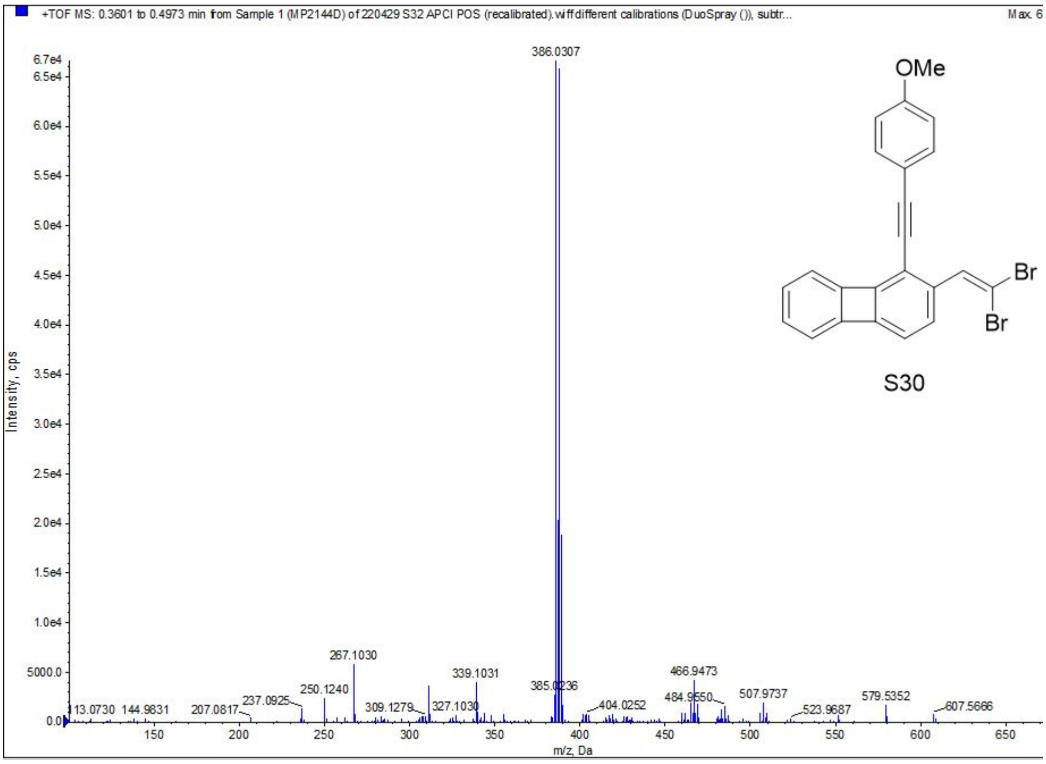

Figure S100. HRMS spectra of **S29** (top) and **S30** (bottom).

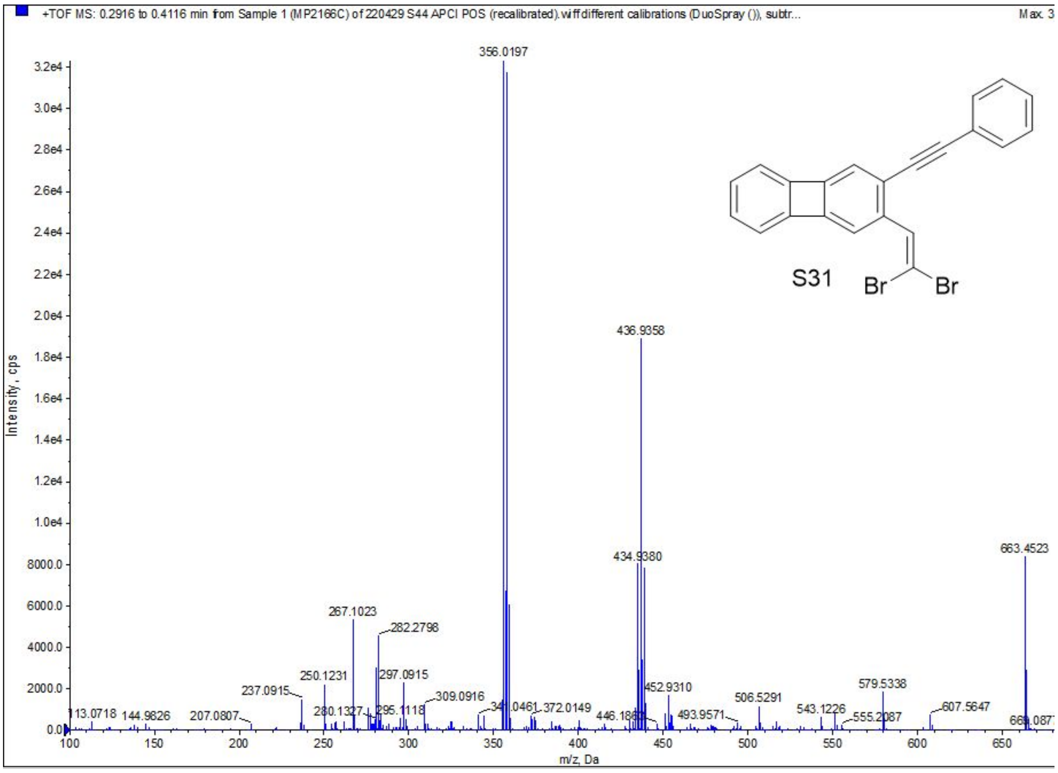

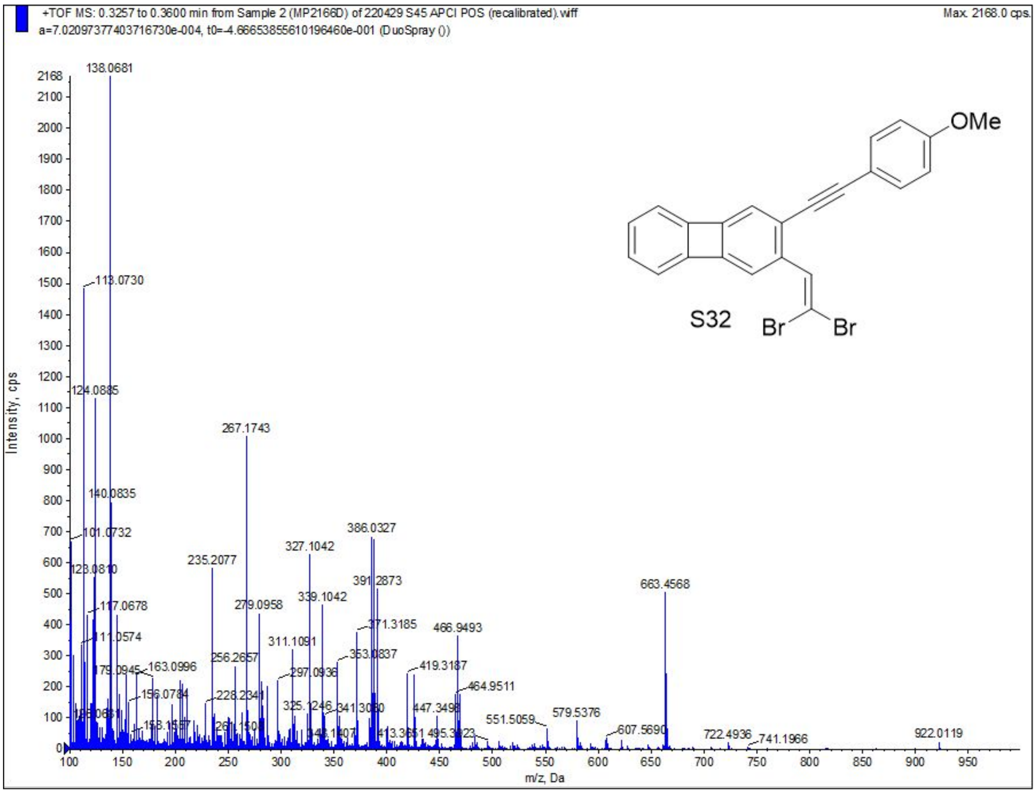

Figure S101. HRMS spectra of **S31** (top) and **S32** (bottom).

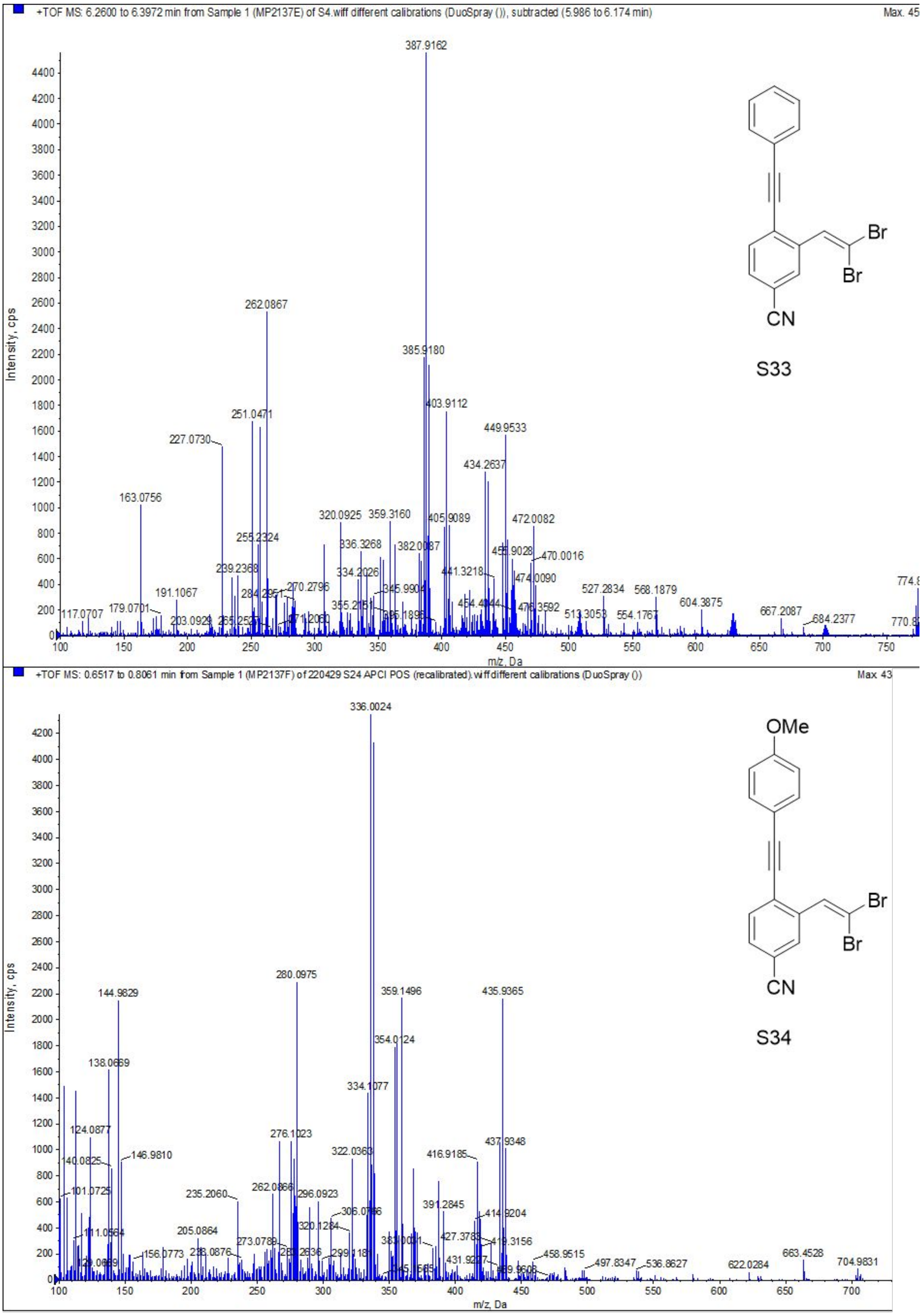

Figure S102. HRMS spectra of **S33** (top) and **S34** (bottom).

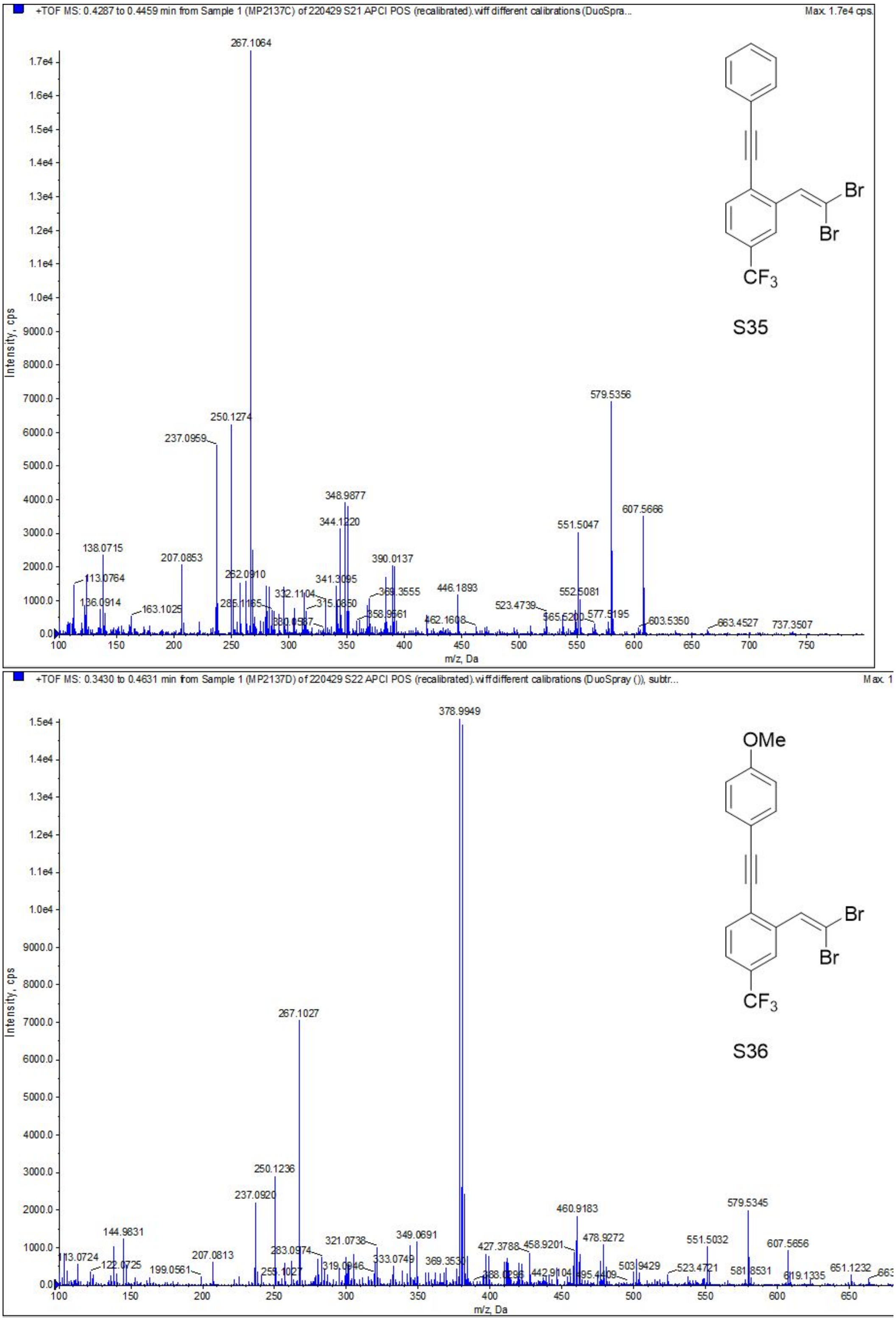

Figure S103. HRMS spectra of **S35** (top) and **S36** (bottom).

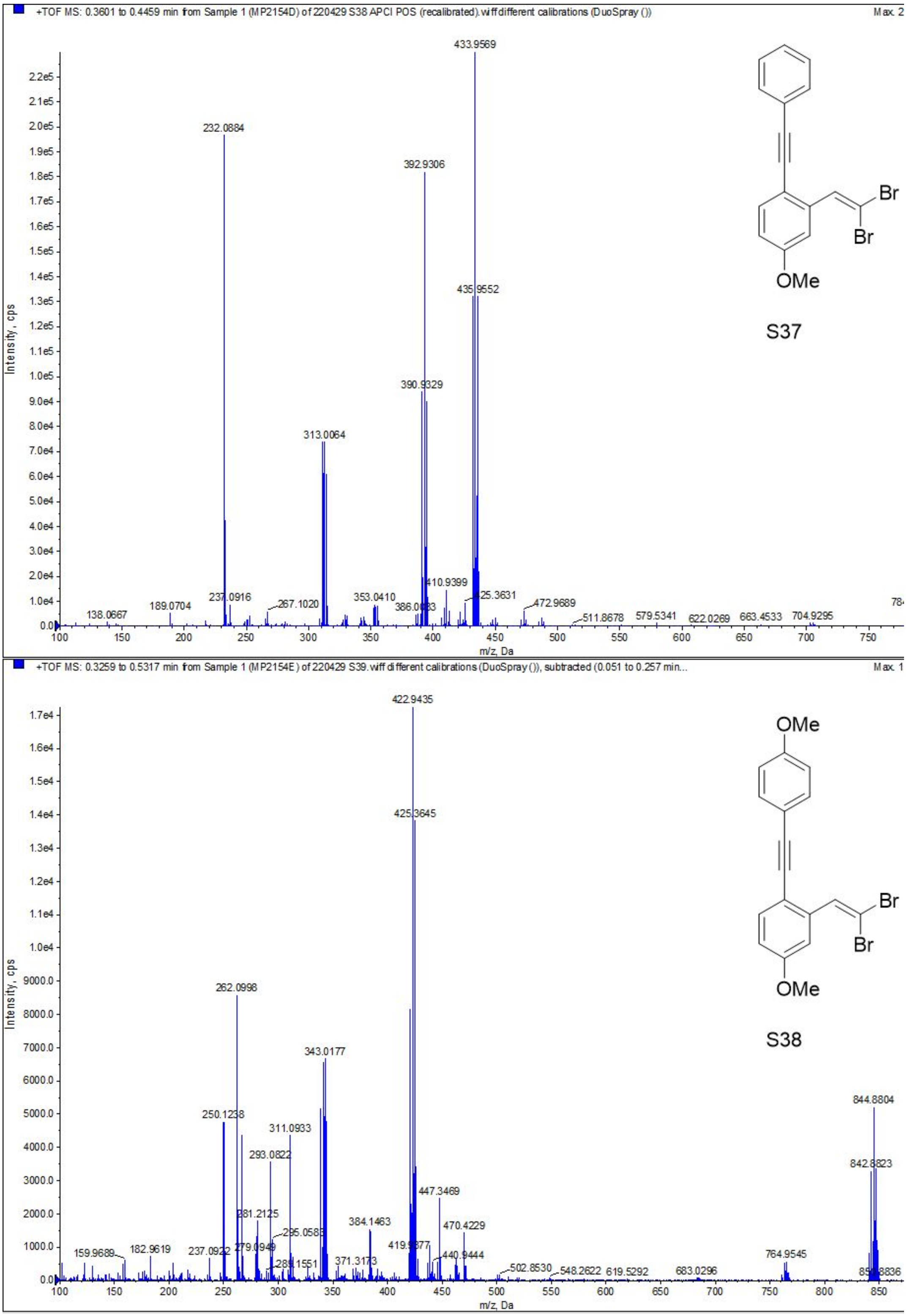

Figure S104. HRMS spectra of **S37** (top) and **S38** (bottom).

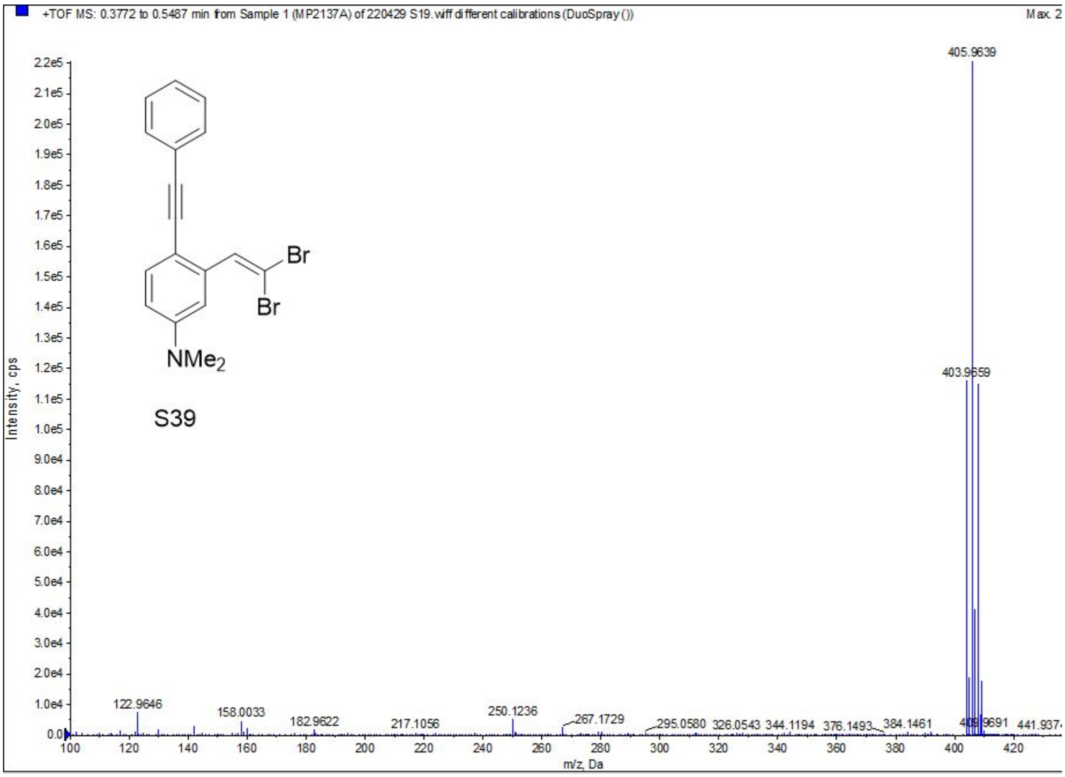

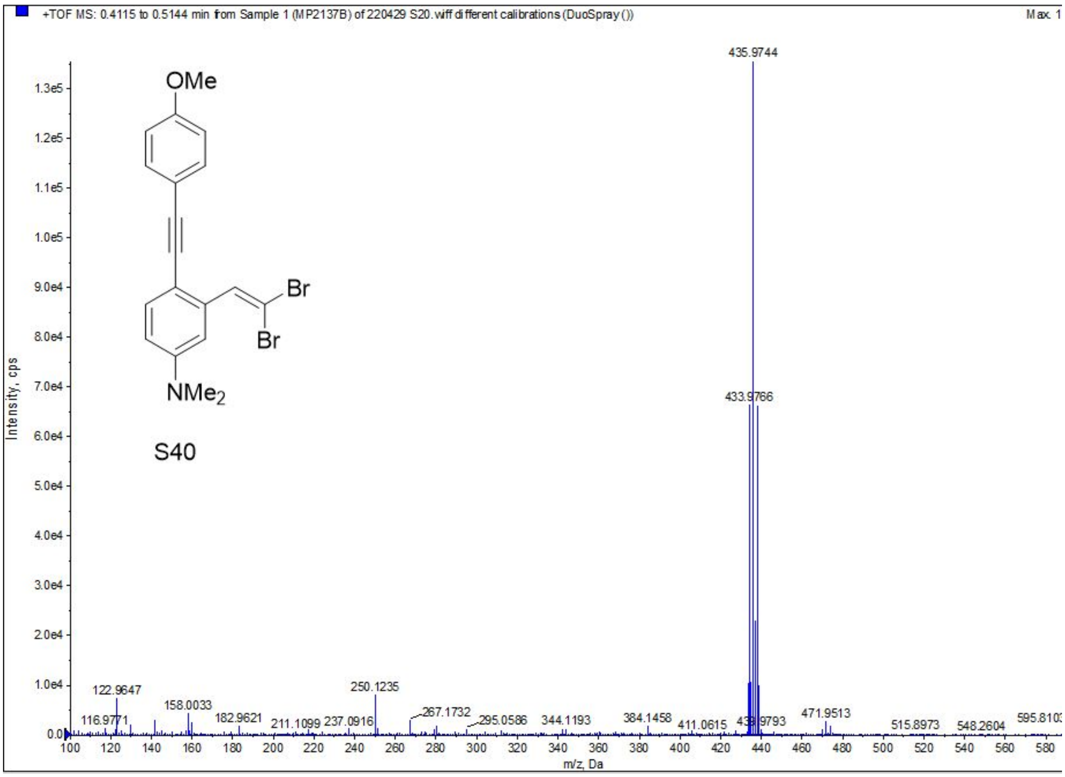

Figure S105. HRMS spectra of **S39** (top) and **S40** (bottom).

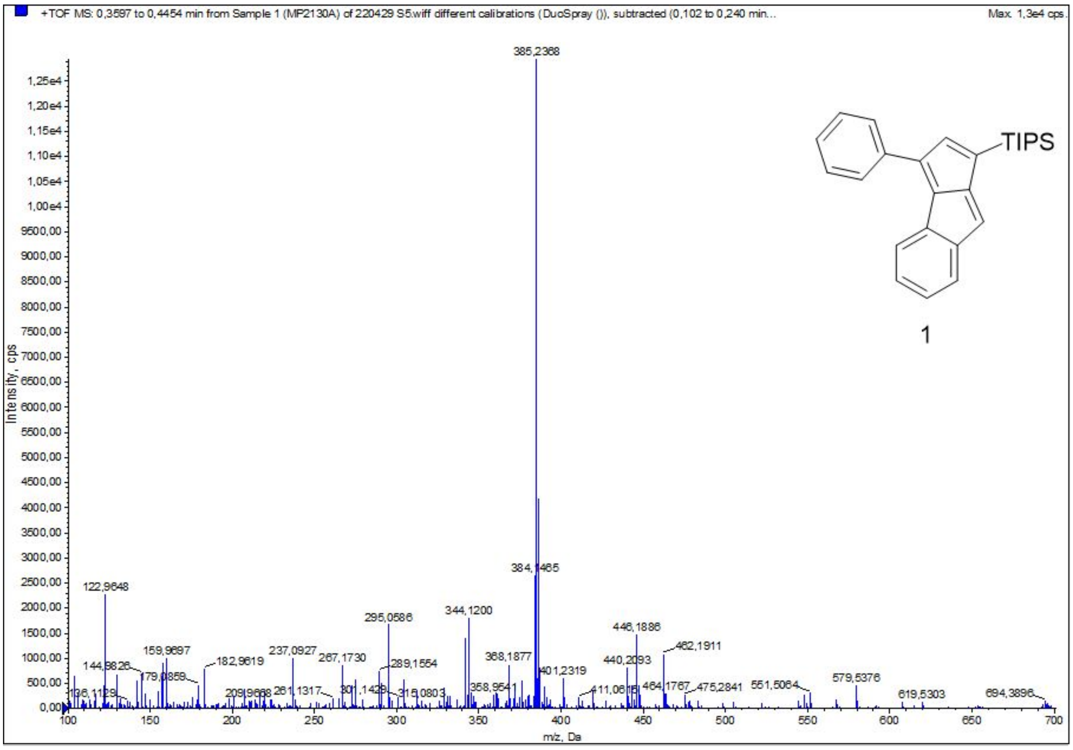

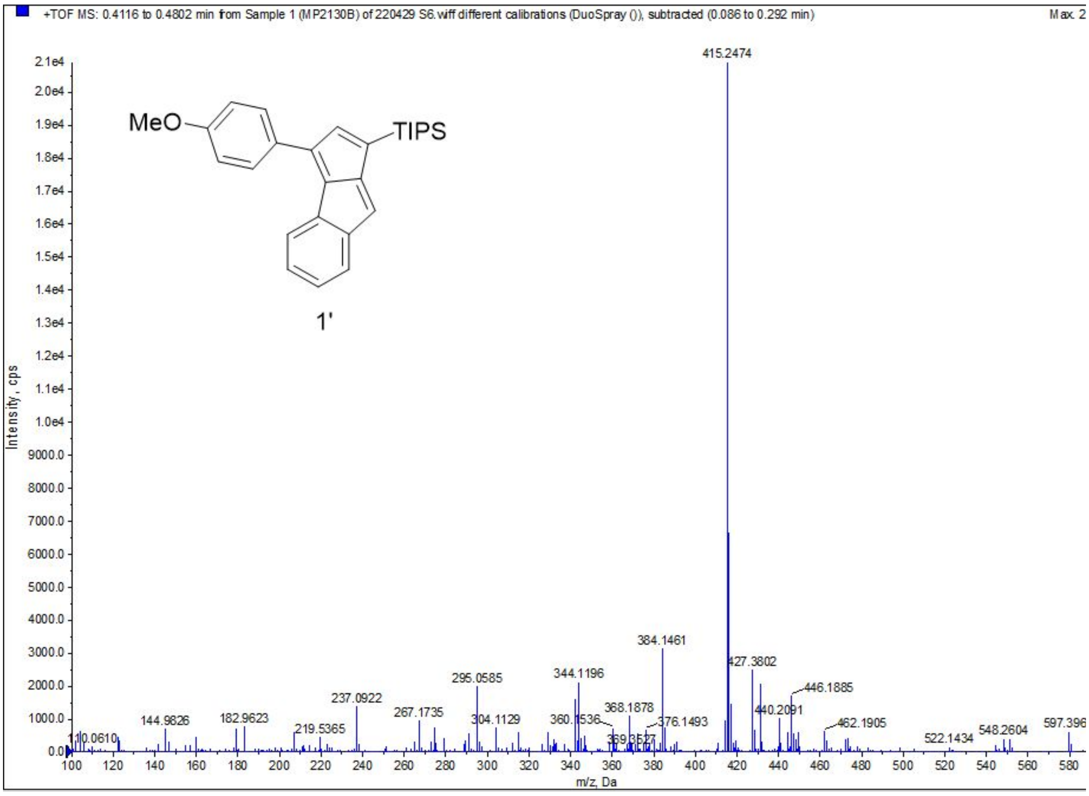

Figure S106. HRMS spectra of **1** (top) and **1’** (bottom).

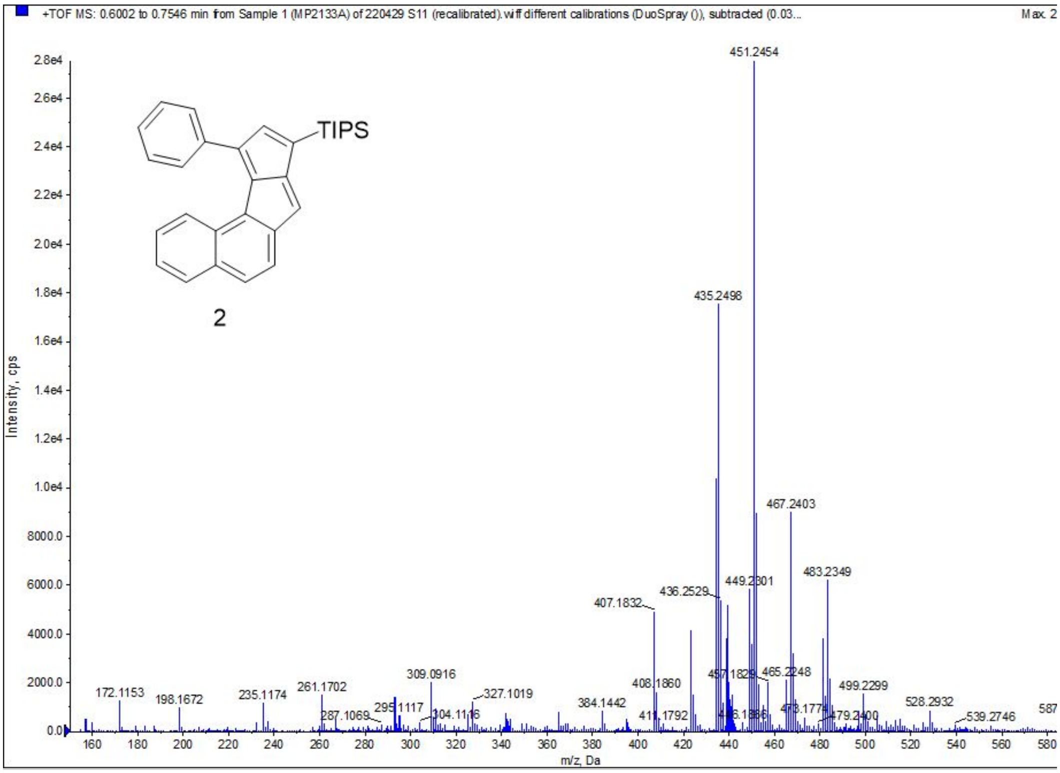

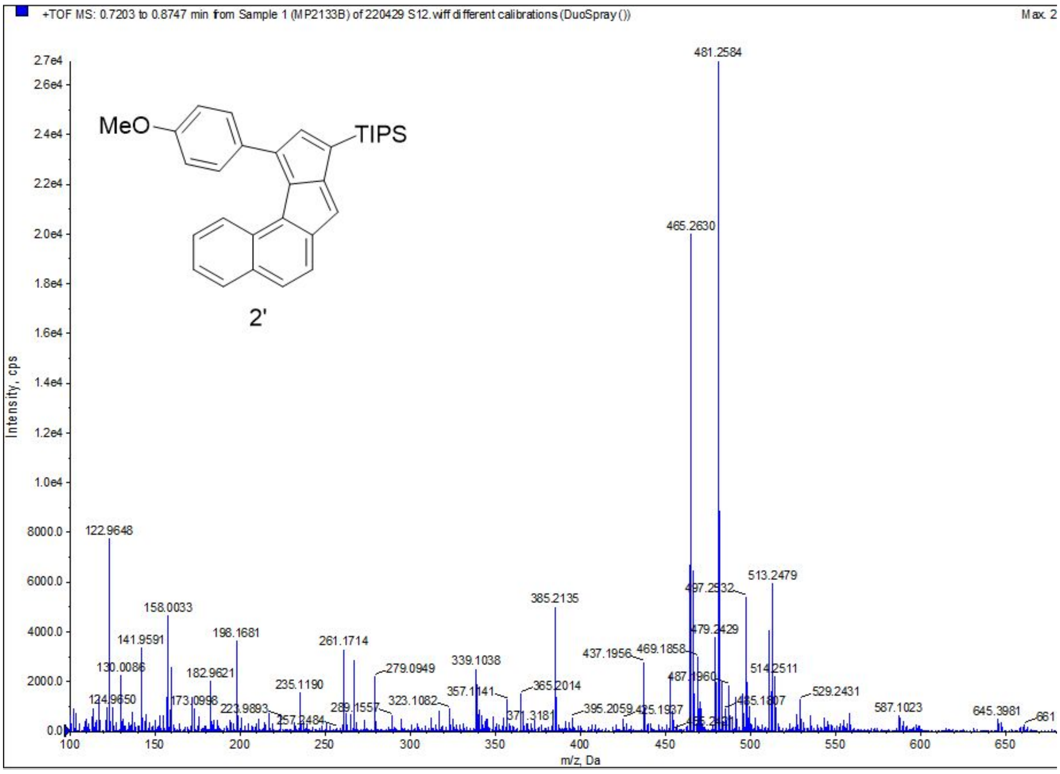

Figure S107. HRMS spectra of **2** (top) and **2’** (bottom).

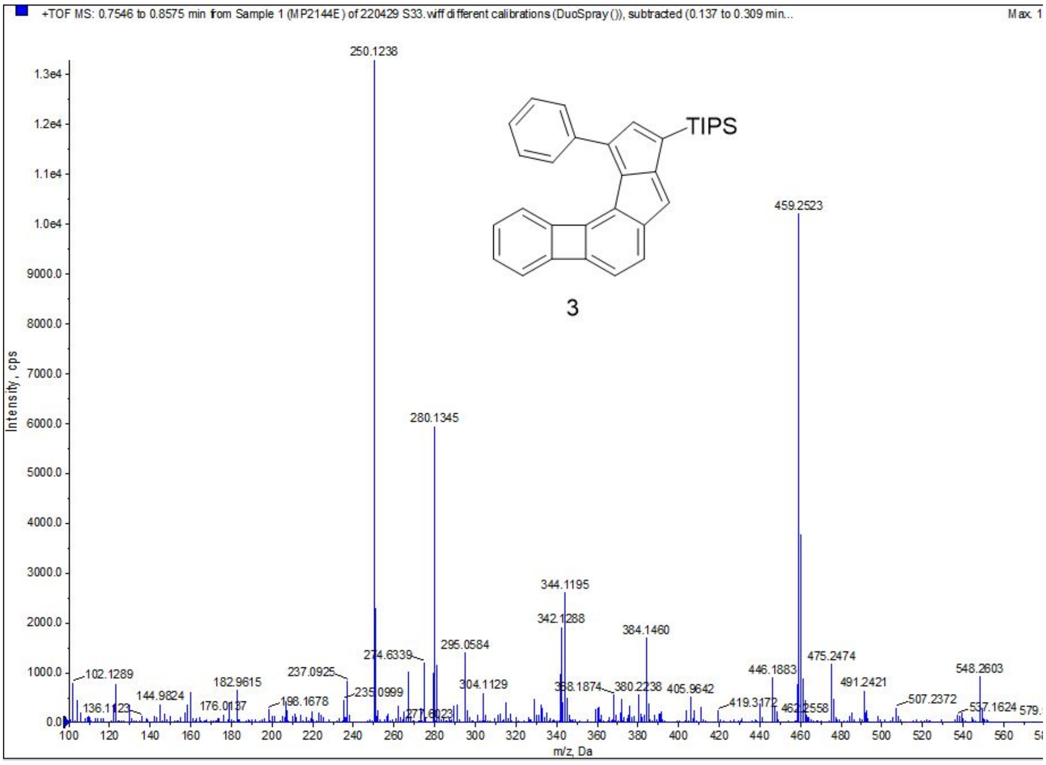

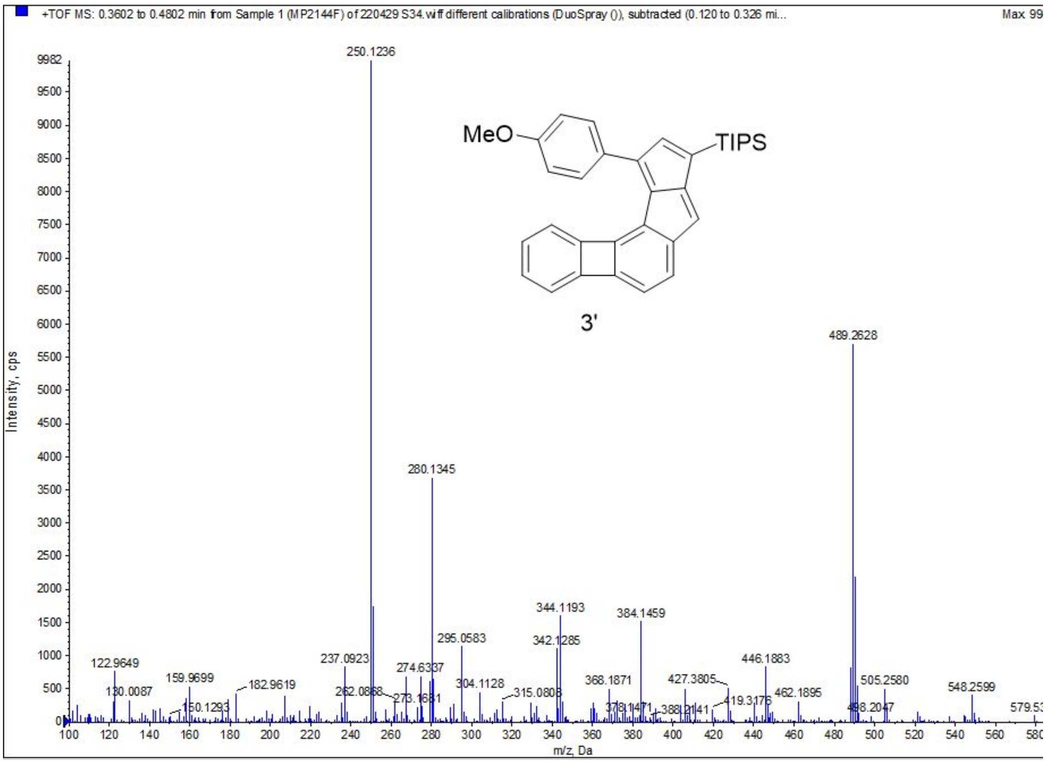

Figure S108. HRMS spectra of **3** (top) and **3'** (bottom).

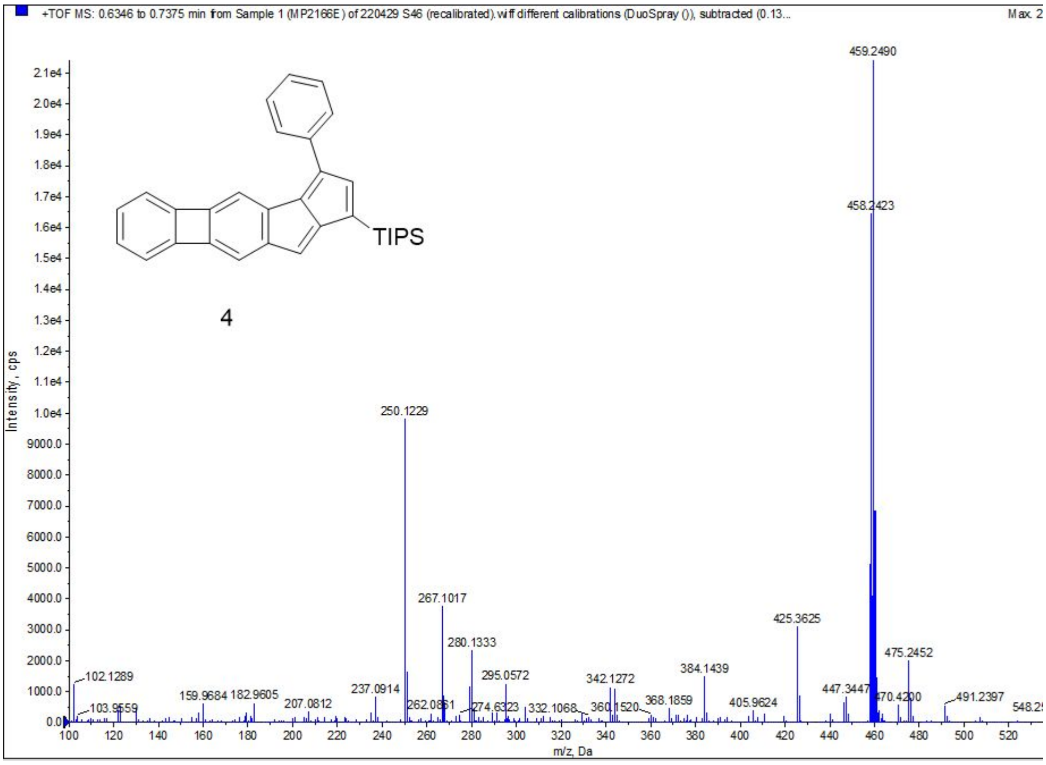

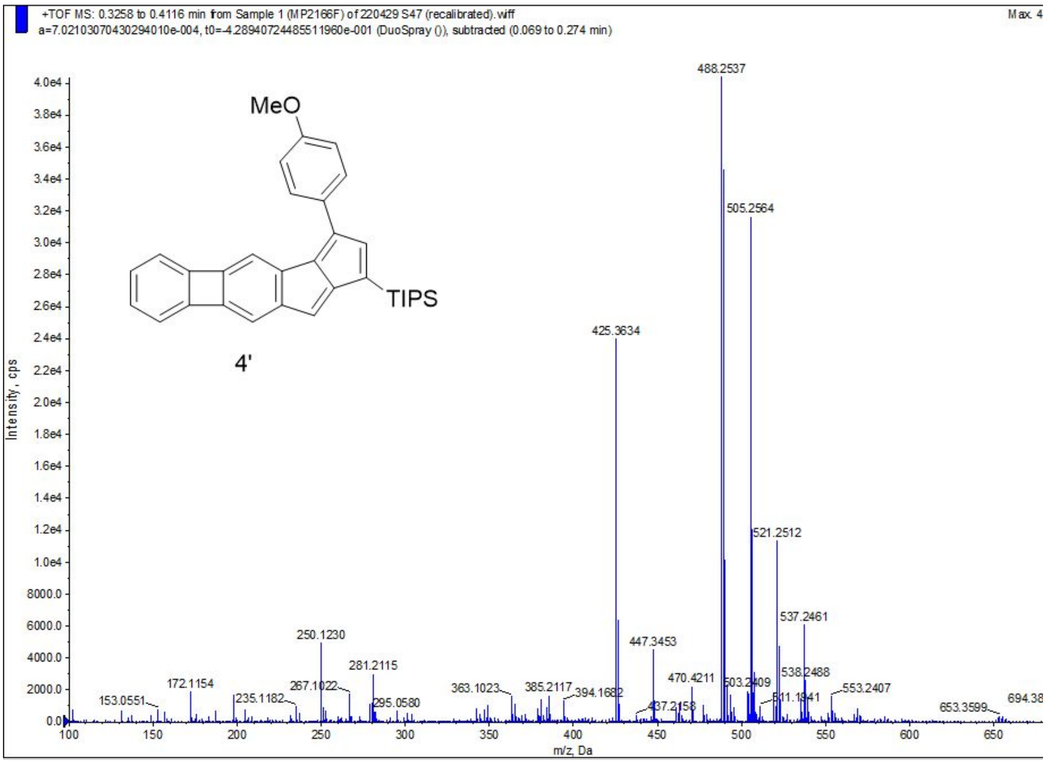

Figure S109. HRMS spectra of **4** (top) and **4’** (bottom).

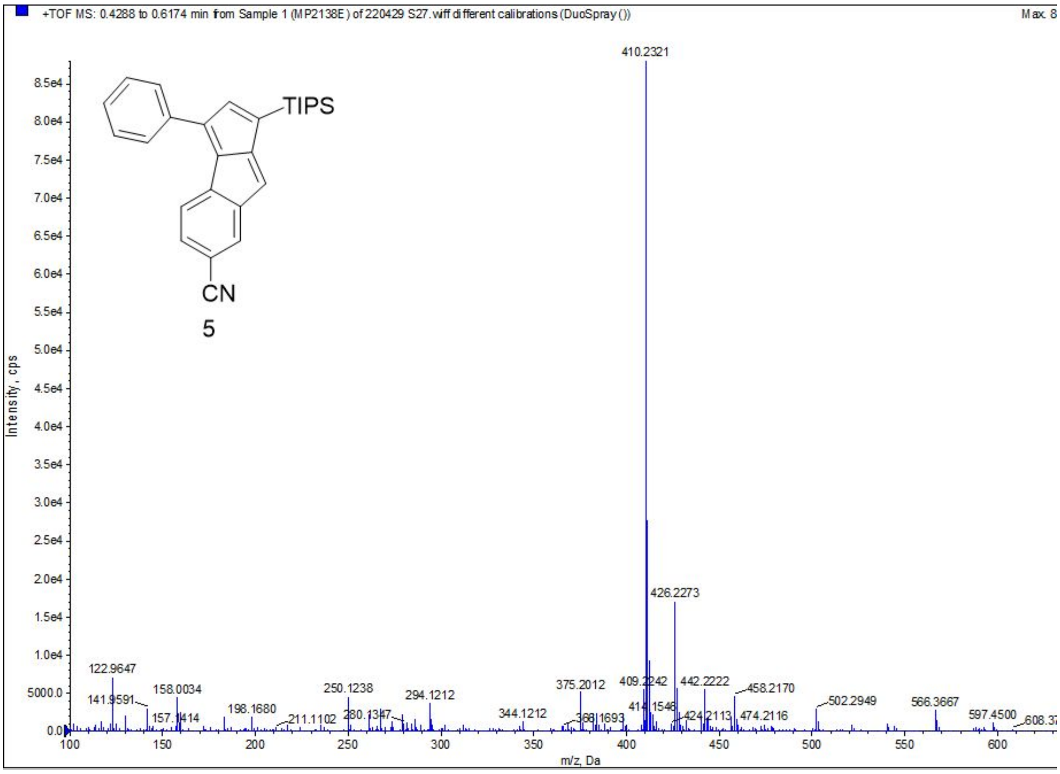

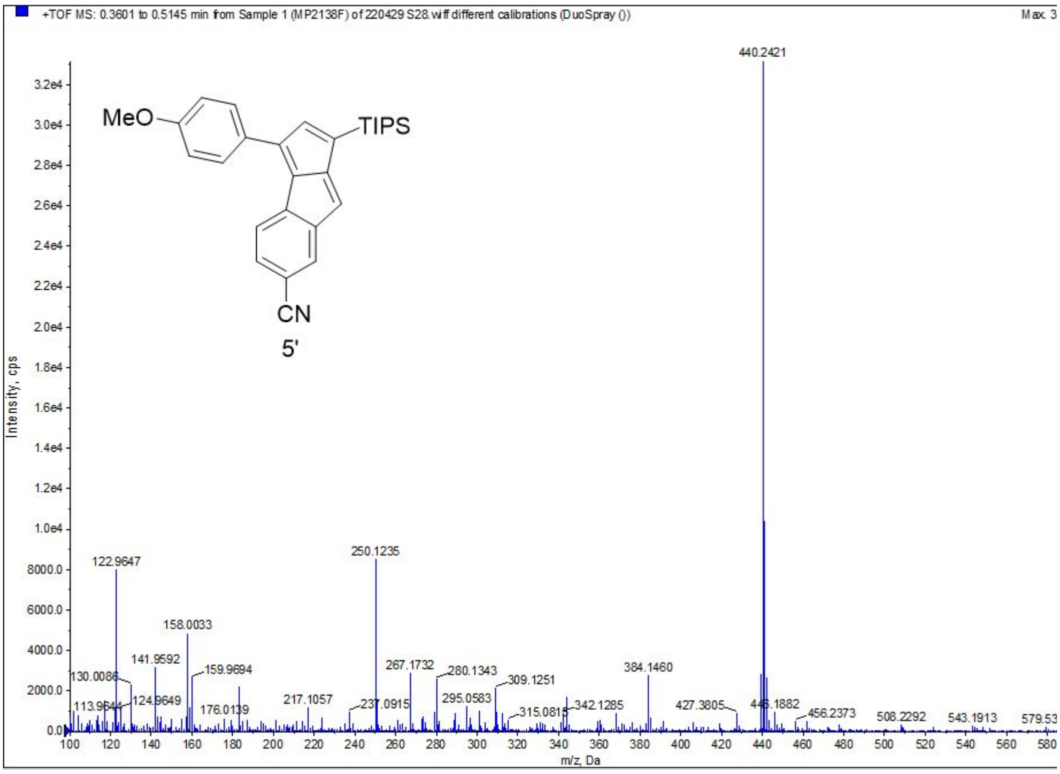

Figure S110. HRMS spectra of **5** (top) and **5’** (bottom).

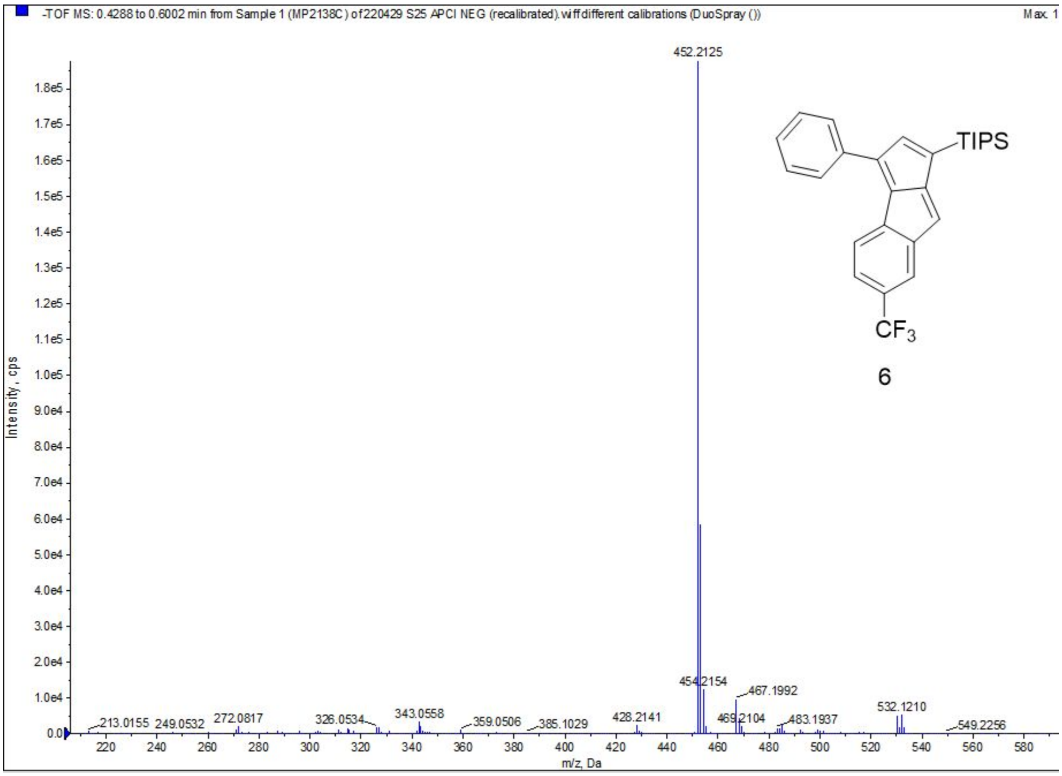

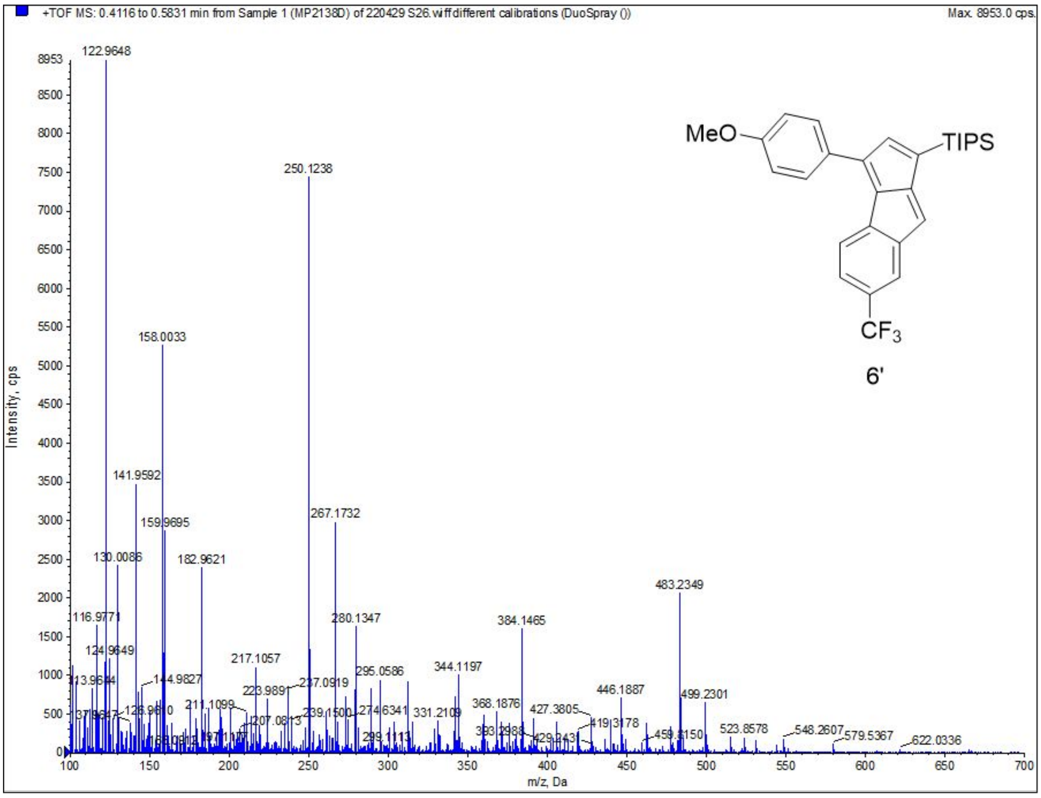

Figure S111. HRMS spectra of **6** (top) and **6’** (bottom).

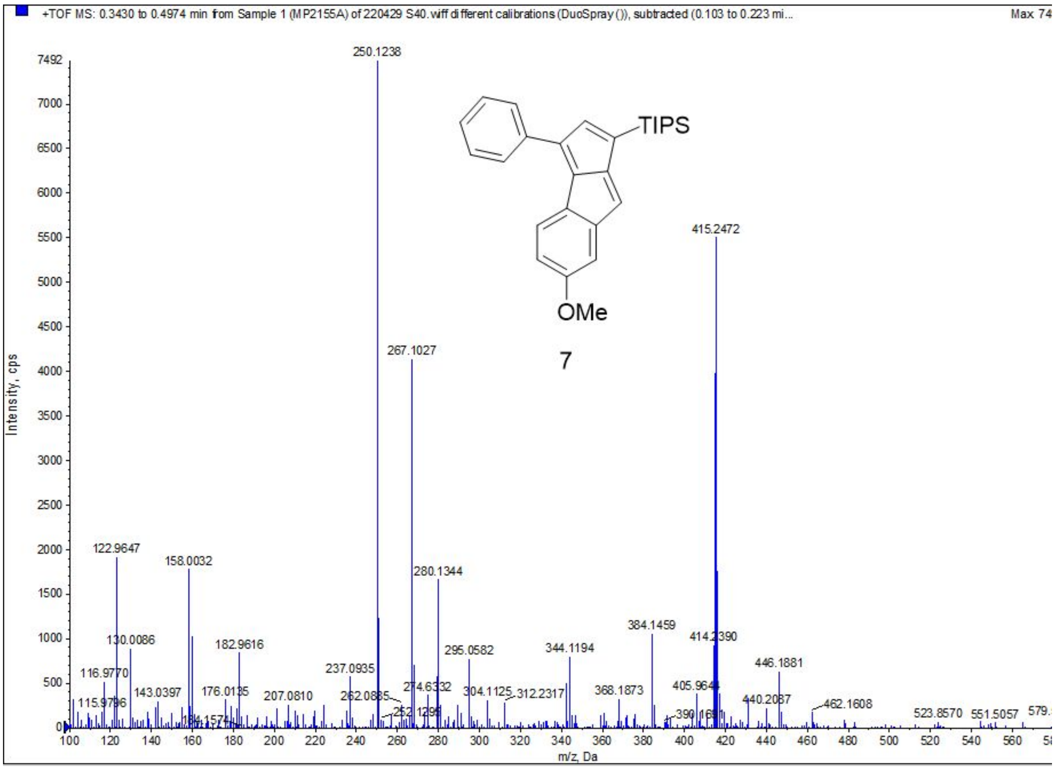

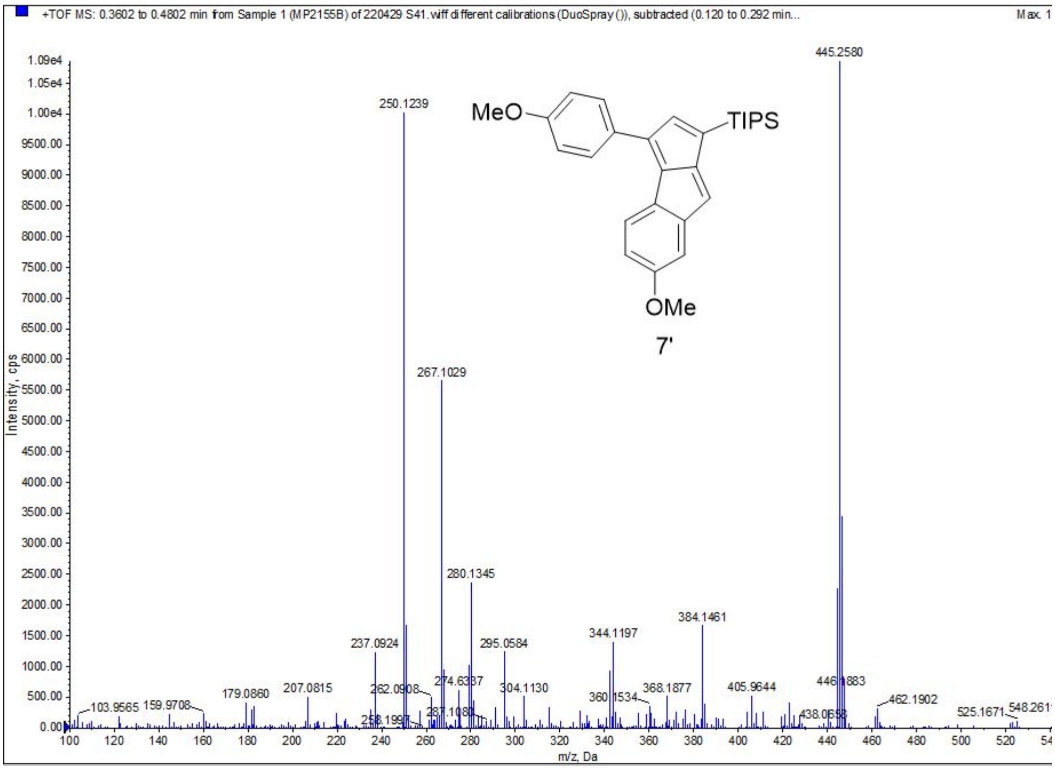

Figure S112. HRMS spectra of **7** (top) and **7'** (bottom).

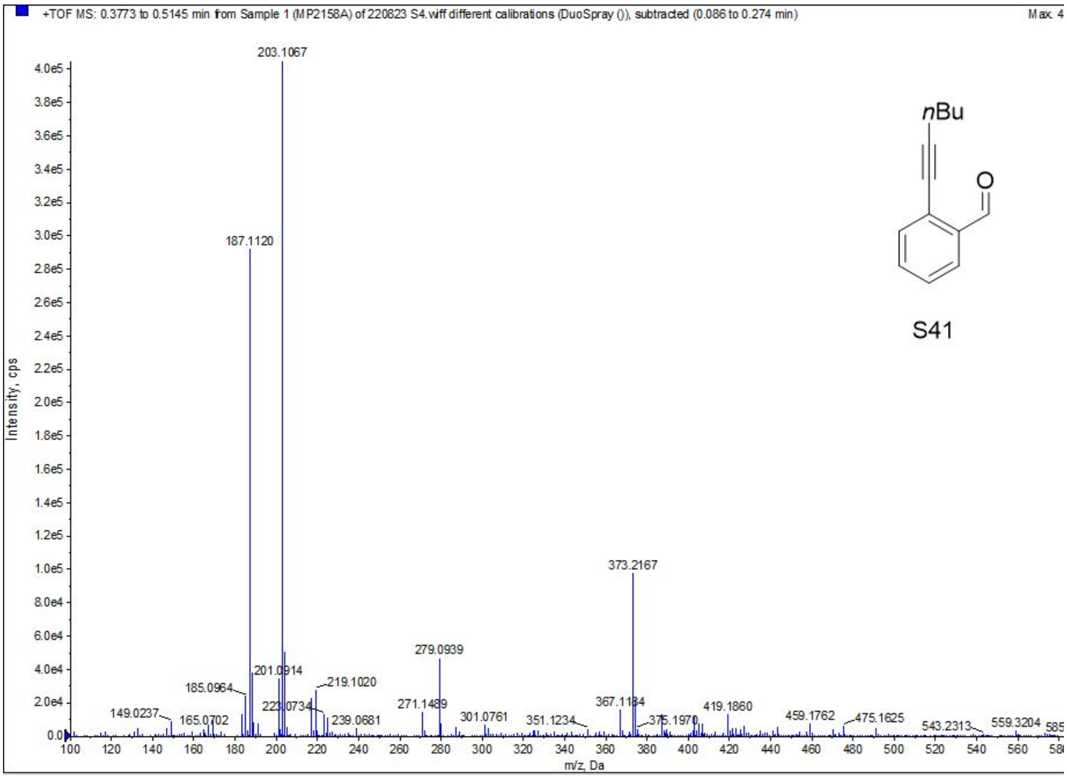

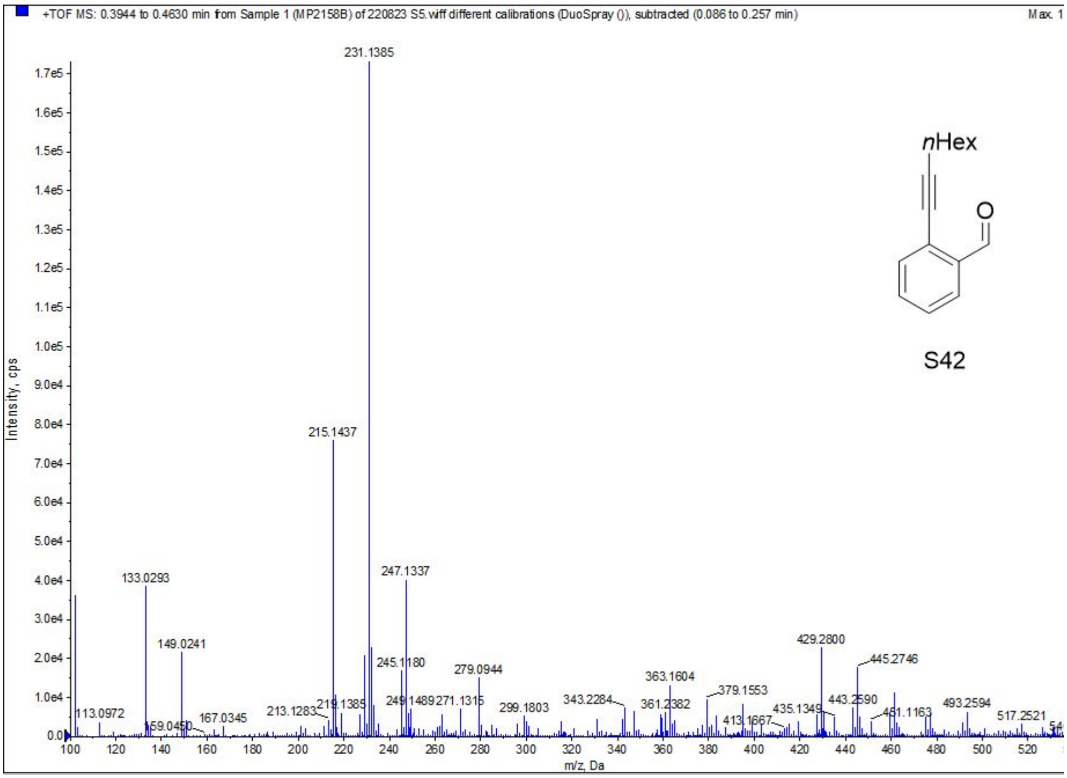

Figure S113. HRMS spectra of **S41** (top) and **S42** (bottom).

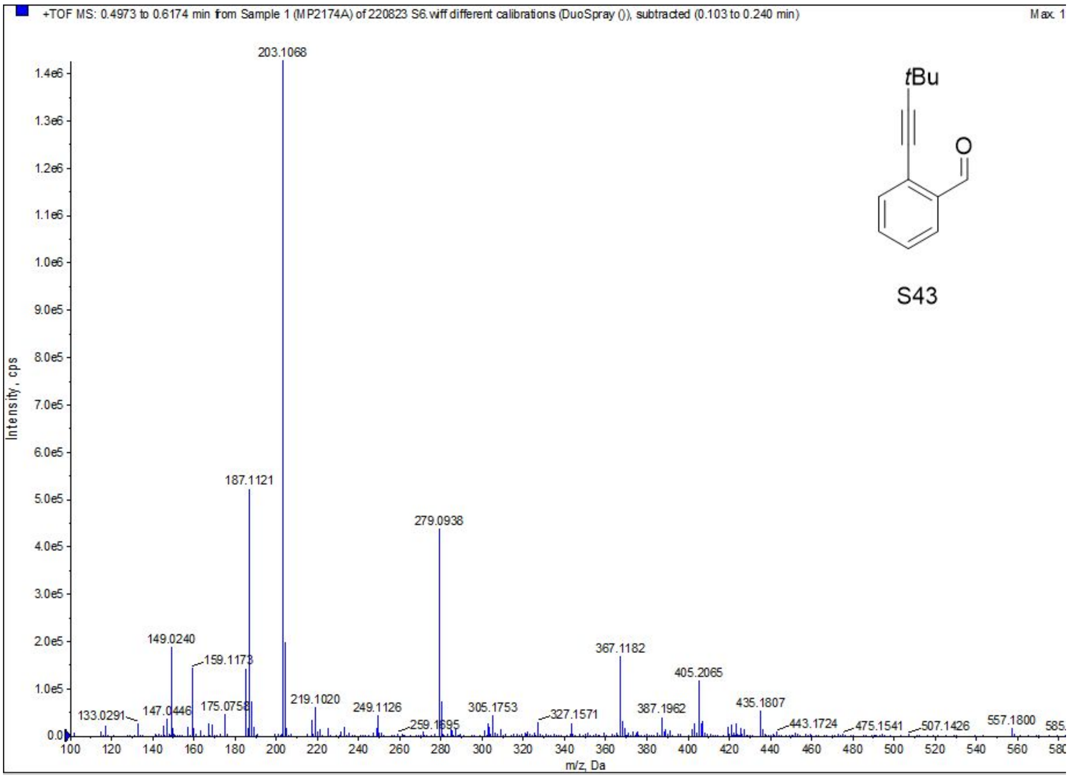

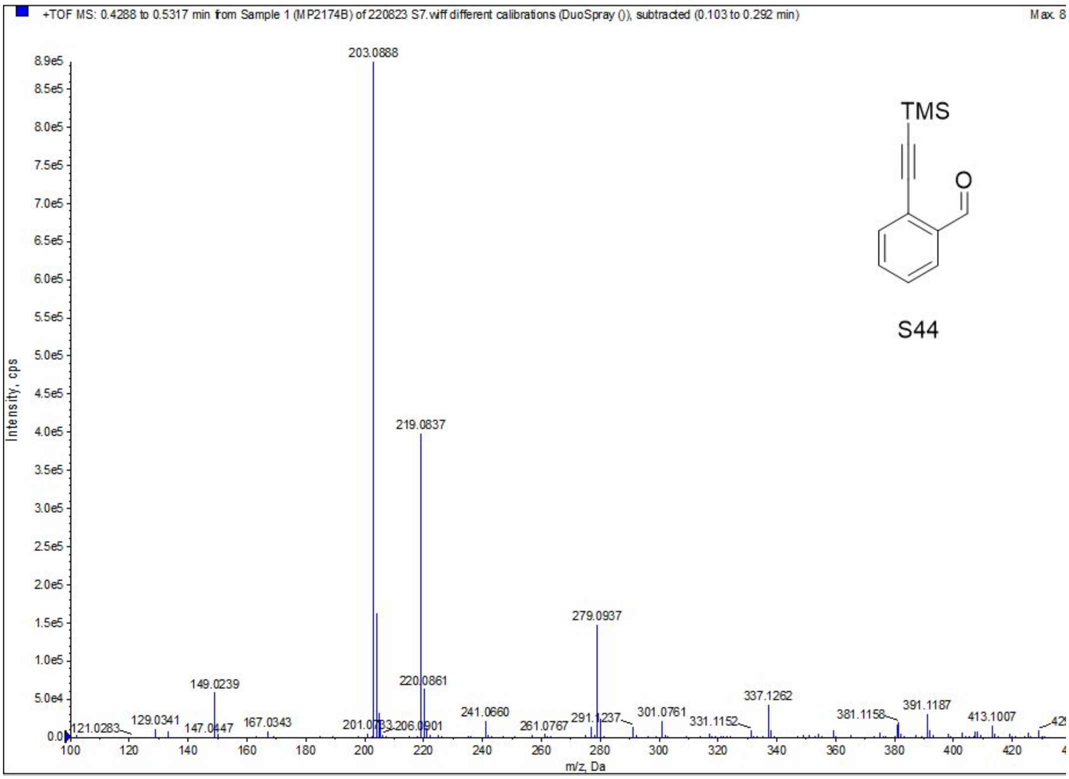

Figure S114. HRMS spectra of **S43** (top) and **S44** (bottom).

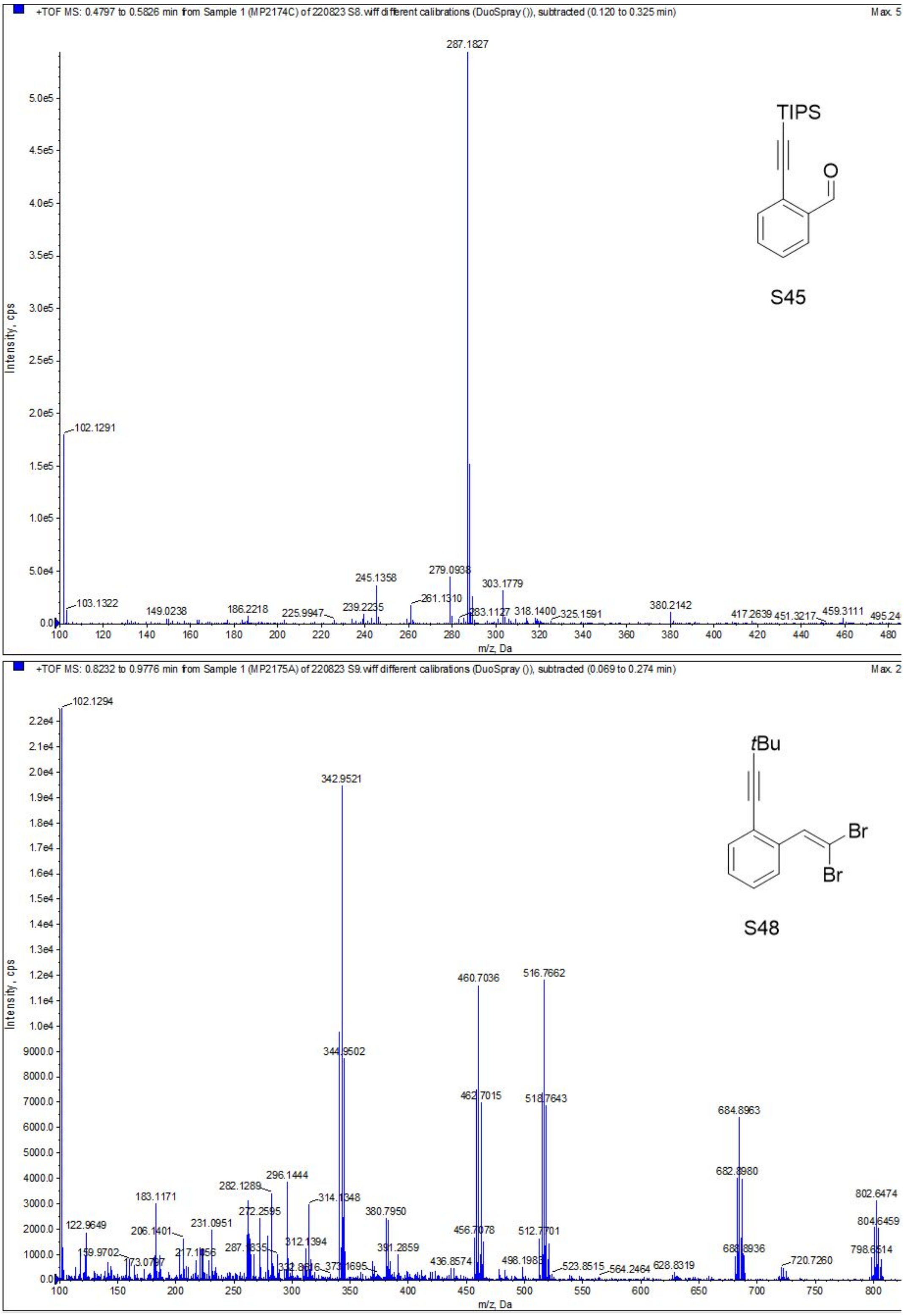

Figure S115. HRMS spectra of **S45** (top) and **S48** (bottom).

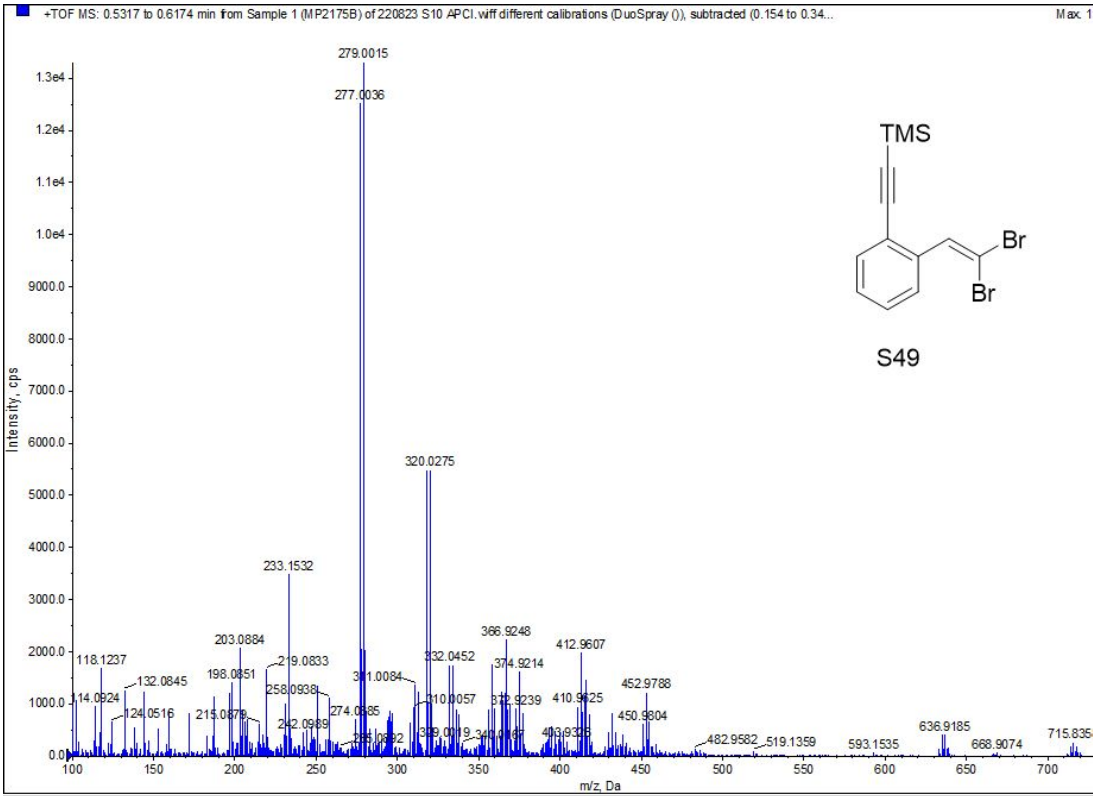

Figure S116. HRMS spectrum of **S49**.

S5 Cartesian coordinates and absolute electronic energies

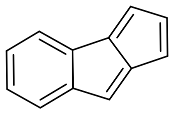

Cyclopenta[a]indene **BP**

E_{el}=-462.150995

	x	y	z
C	-2.82591400	-1.07029900	-0.00000100
C	-3.16249200	0.27659100	0.00000100
C	-2.15834100	1.26327700	0.00000400
C	-0.83353400	0.86685600	0.00000200
C	-0.48679100	-0.51733600	-0.00000200
C	-1.47788200	-1.47978700	-0.00000300
C	0.41269100	1.66678600	-0.00000500
C	1.46745300	0.81563600	-0.00000800
C	0.98129700	-0.56898000	-0.00000400
C	2.92981700	0.76981700	0.00001000
C	3.27616800	-0.54406200	-0.00001000
C	2.05727700	-1.39308000	0.00001300
H	-3.60955700	-1.81948000	-0.00000200
H	-4.20522600	0.57232200	0.00000400
H	-2.42531800	2.31478700	0.00000400
H	-1.23090600	-2.53566200	-0.00000600
H	0.43792300	2.74974100	0.00000100
H	3.60074100	1.61617600	0.00001300
H	4.28469800	-0.93549100	-0.00001700
H	2.06917000	-2.47490300	0.00002300

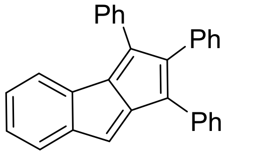

1,2,3-Triphenylcyclopenta[a]indene **TPBP**

E_{el}=-1155.488606

	x	y	z
C	-5.10058700	-1.76286000	-0.07226500
C	-4.84394800	-3.12783600	-0.04649800
C	-3.52067100	-3.60121900	-0.00678900
C	-2.48280400	-2.68539600	0.00964500
C	-2.74384800	-1.28023600	-0.00692200
C	-4.05060100	-0.82528700	-0.04965700
C	-1.02277400	-2.89606100	0.02561800
C	-0.42030200	-1.67954600	0.01159100
C	-1.42741200	-0.62670800	0.01659600
C	0.90547200	-1.05169600	0.00460800
C	0.69223600	0.30631700	-0.00863900
C	-0.79247000	0.58038000	0.00213900
C	-1.41546300	1.91391100	-0.03197800
C	1.72586800	1.36193400	-0.02747700
C	2.17153000	-1.80086800	0.06911600
C	-2.45461200	2.23175700	0.85735100
C	-3.07005100	3.47983300	0.81909100
C	-2.66434300	4.43262600	-0.11468900
C	-1.63371400	4.12997400	-1.00377200
C	-1.00827700	2.88760500	-0.95839100
C	2.74005500	1.35397100	-0.99793900
C	3.72049900	2.34191400	-1.01421500
C	3.70938700	3.35828300	-0.05935400
C	2.70617300	3.38065400	0.90862900
C	1.72107100	2.39666800	0.92122000
C	3.20976400	-1.42296700	0.93628600
C	4.38768600	-2.16024100	1.00222400
C	4.55812200	-3.29167400	0.20436200
C	3.53532200	-3.68203400	-0.65823900
C	2.35390400	-2.94775600	-0.72208300
H	-6.12523700	-1.41015700	-0.11000100
H	-5.66702400	-3.83302900	-0.06070200
H	-3.32113900	-4.66756000	0.00660800
H	-4.27247000	0.23462600	-0.07179500
H	-0.54797100	-3.86877000	0.03822600
H	-2.76293800	1.49810800	1.59330900
H	-3.86387700	3.70986300	1.52121400
H	-3.14501600	5.40389800	-0.14715600
H	-1.31369300	4.86451500	-1.73433300
H	-0.20850200	2.66355500	-1.65343200
H	2.75193100	0.56774900	-1.74363100
H	4.49320800	2.31898500	-1.77477900
H	4.47357200	4.12733200	-0.07137100

H	2.68895400	4.16665000	1.65556000
H	0.94611700	2.42249900	1.67838400
H	3.08355700	-0.55327600	1.56913100
H	5.17346700	-1.85370600	1.68389300
H	5.47718100	-3.86432600	0.25729000
H	3.65797800	-4.55801600	-1.28580600
H	1.57063400	-3.24960600	-1.40801700

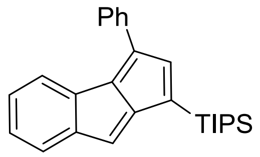

Triisopropyl(3-phenylcyclopenta[a]inden-1-yl)silane **1**

E_{el}=-1337.926874

	x	y	z
C	-4.32077100	3.20978000	0.06069500
C	-3.47096500	4.30751800	0.04068900
C	-2.07705800	4.12399500	0.00619000
C	-1.57134900	2.83621100	-0.01489800
C	-2.44228300	1.70274200	-0.01073000
C	-3.81267600	1.89662100	0.03398700
C	-0.17559500	2.36009000	-0.01716300
C	-0.18484700	1.00229800	-0.01446500
C	-1.56729500	0.52102500	-0.02588400
C	0.72957100	-0.15761000	0.02708700
C	-0.10175700	-1.23987900	0.05915100
C	-1.54060300	-0.84623800	0.01268600
C	-2.63723600	-1.81792700	-0.00775500
C	-3.80876000	-1.58083800	-0.74706000
C	-4.84169800	-2.51180800	-0.76695300
C	-4.73017200	-3.70145400	-0.04674600
C	-3.57073000	-3.95612900	0.68457700
C	-2.53244200	-3.02989300	0.69726400
Si	2.61814300	-0.18096000	0.06158600
C	3.21782600	0.23781600	1.84058000
C	3.24778500	1.18207800	-1.13686300
C	3.18390200	-1.97014300	-0.35287800
C	2.69434600	-0.77000100	2.88226700
C	2.88829000	1.67614700	2.28220500
C	2.68044200	1.09321300	-2.56665400
C	4.78168800	1.32510200	-1.16983300
C	4.66221200	-2.25477100	-0.02140400
C	2.86505100	-2.43125800	-1.78871100
H	-5.39376400	3.36156700	0.09740800
H	-3.88143300	5.31056500	0.05774100
H	-1.41241800	4.98159200	0.00291500
H	-4.49704100	1.05778800	0.05811600
H	0.68514200	3.01637700	-0.00889800
H	0.21264500	-2.27563800	0.07453200
H	-3.88892600	-0.67420900	-1.33417700
H	-5.73215400	-2.31410800	-1.35346700
H	-5.53630200	-4.42611400	-0.06151200
H	-3.47482400	-4.87848000	1.24655300
H	-1.64016800	-3.23520500	1.27739300
H	4.31270600	0.14747900	1.81362200
H	2.85092200	2.10737900	-0.69581600
H	2.58235400	-2.59145000	0.32600200
H	2.99070100	-1.79808900	2.65575900
H	3.08107500	-0.53216400	3.88001600
H	1.60169400	-0.74668300	2.94061000
H	3.32652700	2.42657600	1.61824000
H	3.27676300	1.86821700	3.28914100
H	1.80788900	1.84725600	2.31489500
H	1.59095000	1.01076200	-2.57362300
H	2.94892800	1.98664000	-3.14266400
H	3.08121400	0.23100000	-3.10608800
H	5.21402500	1.43925300	-0.17169100
H	5.07611800	2.20359200	-1.75578500
H	5.25463100	0.45636900	-1.63733300
H	4.91148800	-2.01776600	1.01559600
H	4.89328300	-3.31474400	-0.17889800
H	5.33806900	-1.68121100	-0.66243500
H	1.82653400	-2.24069500	-2.07011300
H	3.04616900	-3.50713400	-1.89631800
H	3.50394100	-1.92540900	-2.51821100

References

1. Mayer, P. J.; El Bakouri, O.; Holczbauer, T.; Samu, G. F.; Janáky, C.; Ottosson, H.; London, G. Structure–Property Relationships in Unsymmetric Bis(antiaromatics): Who Wins the Battle between Pentalene and Benzocyclobutadiene? *J. Org. Chem.* **2020**, *85*, 5158–5172.

2. Gazdag, T.; Mayer, P. J; Kalapos, P. P.; Holczbauer T.; El Bakouri, O.; London G. Unsymmetrical Thienopentalenes: Synthesis, Optoelectronic Properties, and (Anti)aromaticity Analysis. *ACS Omega* **2022**, *7*, 8336–8349.

3 Rivera-Fuentes, P.; von Wantoch Rekowski, M.; Schweizer, W. B.; Gisselbrecht, J.-P.; Boudon, C.; Diederich, F. Cascade Carbopalladation Reaction Between Alkynes and *Gem*-Dibromoolefins: Facile Access to Monoannelated Pentalenes. *Org. Lett.* **2012**, *14*, 4066–4069.

4 London, G.; von Wantoch Rekowski, M.; Dumele, O.; Schweizer, W. B.; Gisselbrecht, J.-P.; Boudon, C.; Diederich, F. Pentalenes with Novel Topologies: Exploiting the Cascade Carbopalladation Reaction Between Alkynes And *gem*-Dibromoolefins. *Chem. Sci.* **2014**, *5*, 965–972.

5 Ye, S.; Yang, X.; Wu, J. Rapid Access to 1-Methyleneindenes via Palladium-Catalyzed Tandem Reactions of 1-(2,2-Dibromovinyl)-2-Alkynylbenzenes with Arylboronic Acids. *Chem. Commun.* **2010**, *46*, 2950–2952.

6 Claus, V.; Molinari, L.; Büllmann, S.; Thusek, J.; Rudolph, M.; Rominger, F.; Hashmi, A. S. K. Gold-Catalyzed Cyclisation by 1,4-Dioxidation. *Chem. Eur. J.* **2019**, *25*, 9385–9389.

7 Yu, B.; Huang, R.; Li, R.; Zhang H.; Huang H. Silver-Catalyzed Chemodivergent Assembly of Aminomethylated Isochromenes and Naphthols. *Chem. Commun.* **2022**, *58*, 3969–3972.

8 Mora-Radó, H.; Bialy, L.; Czechtizky, W.; Méndez, M.; Harrity, J. P. A. An Alkyne Diboration/6π-Electrocyclization Strategy for the Synthesis of Pyridine Boronic Acid Derivatives. *Angew. Chem. Int. Ed.* **2016**, *55*, 5834–5836.

9 Liu, B.; Ouyang, W.; Nie, J.; Gao, Y.; Feng, K.; Huo, Y.; Chen, Q.; Li X. Weak Coordinated Nitrogen Functionality Enabled Regioselective C–H Alkynylation via Pd(II)/Mono-N-Protected Amino Acid Catalysis. *Chem. Commun.* **2020**, *56*, 11255–11258.

10 Hua-Feng, H.; Yang, Y.; Yongwen, J.; Weiliang B. Synthesis of Diynes from 1-(2,2-Dibromovinyl)-2-(Phenylethynyl)Benzene and Imidazole/Benzimidazole Via a CuI/Pd(OAc)$_2$ Catalysed Cascade Reaction. *J. Chem. Res.* **2014**, *38*, 399–403.

11 Hendrich, C. M.; Bongartz, L. M.; Hoffmann, M. T.; Zschieschang, U.; Borchert, J. W.; Sauter, D.; Krämer, P.; Rominger, F.; Mulks, F. F.; Rudolph, M.; Dreuw, A.; Klauk, H.; Hashmi A. S. K. Gold Catalysis Meets Materials Science – A New Approach to π-Extended Indolocarbazoles. *Adv. Synth. Catal.* **2021**, *363*, 549–557.

12 Bucher, G.; Mahajan, A. A.; Schmittel, M. Photochemical C^2−C^6 Cyclization of Enyne−Allenes: Detection of a Fulvene Triplet Diradical in the Laser Flash Photolysis. *J. Org. Chem.* **2008**, *73*, 8815–8828.

13 Yeh, M.-C. P.; Liang, C.-J.; Chen, H.-F.; Weng, Y.-T. Indium(III)-Catalyzed Cyclization of Aromatic 5-Enynamides: Facile Synthesis of 2-Aminonaphthalenes, 2-Amino-1*H*-indenes, and 2,3-Dihydro-1*H*-indeno[2,1-*b*]pyridines. *Adv. Synth. Catal.* **2015**, *357*, 3242–3254.

14 Frisch, M. J.; Trucks, G. W.; Schlegel, H. B.; Scuseria, G. E.; Robb, M. A.; Cheeseman, J. R.; Scalmani, G.; Barone, V.; Mennucci, B.; Petersson, G. A.; Nakatsuji, H.; Caricato, M.; Li, X.; Hratchian, H. P.; Izmaylov, A. F.; Bloino, J.; Zheng, G.; Sonnenberg, J. L.; Hada, M.; Ehara, K.; Toyota, K.; Fukuda, R.; Hasegawa, J.; Ishida, M.; Nakajima, T.; Honda, Y.; Kitao, O.; Nakai, H.; Vreven, T.; Montgomery, J. A.; Peralta, J. E. Jr.; Ogliaro, F.; Bearpark, M.; Heyd, J. J.; Brothers, E.; Kudin, K. N.; Staroverov, V. N.; Keith, T.; Kobayashi, R.; Normand, J.; Raghavachari, K.; Rendell, A.;Burant, J. C.; Iyengar, S. S.; Tomasi, J.; Cossi, M.; Rega, N.; Millam, J. M.; Klene, M.; Knox, J. E.; Cross, J. B.; Bakken, V.; Adamo, C.; Jaramillo, J.; Gomperts, R.; Stratmann, R. E.; Yazyev, O.; Austin, A. J.; Cammi, R.; Pomelli, C.; Ochterski, J. W.; Martin, R. L.; Morokuma, K.; Zakrzewski, V. G.; Voth, G. A.; Salvador, P.; Dannenberg, J. J.; Dapprich, S.; Daniels, A. D.; Farkas, O.; Foresman, J. B.; Ortiz, J. V.; Cioslowski, J.; Fox, D. J. *Gaussian 09*, Revision E.01, Gaussian, Inc., Wallingford CT, 2013.

15 Stephens, P. J.; Devlin, F. J.; Chabalowski, C. F.; Frisch, M. J. Ab Initio Calculation of Vibrational Absorption and Circular Dichroism Spectra Using Density Functional Force Fields. *J. Phys. Chem.* **1994**, *98*, 11623–11627..

16 Krishnan, R.; Binkley, K. S.; Seeger, R.; Pople, J. Self-Consistent Molecular Orbital Methods. XX. A Basis Set for Correlated Wave Functions. *J. Chem. Phys*. **1980**, *72*, 650–654.

17 Herges, R.; Geuenich, D. Delocalization of Electrons in Molecules. *J. Phys. Chem. A* **2001**, *105*, 3214–3220.

18 Geuenich, D.; Hess, K.; Köhler F.; Herges, R. Anisotropy of the Induced Current Density (ACID), a General Method to Quantify and Visualize Electronic Delocalization. *Chem. Rev.* **2005**, *105*, 3758–3772.

19 Gershoni-Poranne, R.; Stanger, A. The NICS-*XY*-Scan: Identification of Local and Global Ring Currents in Multi-Ring Systems. *Chem. Eur. J.* **2014**, *20*, 5673–5688.

20 Stanger, A. Obtaining Relative Induced Ring Currents Quantitatively from NICS. *J. Org. Chem.* **2010**, *75*, 2281–2288.

21 Stanger, A. Nucleus-Independent Chemical Shifts (NICS): Distance Dependence and Revised Criteria for Aromaticity and Antiaromaticity. *J. Org. Chem.* **2006**, *71*, 883–893.

22 Stanger, A.; Rahalkar, A. Aroma, https://chemistry.technion.ac.il/en/team/amnon-stanger/.
